# Regioselective *N*-alkylation of the 1*H*-indazole scaffold; ring substituent and *N*-alkylating reagent effects on regioisomeric distribution

**DOI:** 10.3762/bjoc.17.127

**Published:** 2021-08-02

**Authors:** Ryan M Alam, John J Keating

**Affiliations:** 1 Analytical and Biological Chemistry Research Facility (ABCRF), University College Cork, College Road, Cork, T12 YN60, Ireland; 2 School of Chemistry, Kane Building, University College Cork, T12 YN60, Ireland,; 3 School of Pharmacy, Pharmacy Building, University College Cork, T12 YN60, Ireland

**Keywords:** indazole, *N*-alkylation, regioselective, sodium hydride, tetrahydrofuran

## Abstract

The indazole scaffold represents a promising pharmacophore, commonly incorporated in a variety of therapeutic drugs. Although indazole-containing drugs are frequently marketed as the corresponding *N*-alkyl 1*H*- or 2*H*-indazole derivative, the efficient synthesis and isolation of the desired *N*-1 or *N*-2 alkylindazole regioisomer can often be challenging and adversely affect product yield. Thus, as part of a broader study focusing on the synthesis of bioactive indazole derivatives, we aimed to develop a regioselective protocol for the synthesis of *N*-1 alkylindazoles. Initial screening of various conditions revealed that the combination of sodium hydride (NaH) in tetrahydrofuran (THF) (in the presence of an alkyl bromide), represented a promising system for *N*-1 selective indazole alkylation. For example, among fourteen C-3 substituted indazoles examined, we observed > 99% *N*-1 regioselectivity for 3-carboxymethyl, 3-*tert*-butyl, 3-COMe, and 3-carboxamide indazoles. Further extension of this optimized (NaH in THF) protocol to various C-3, -4, -5, -6, and -7 substituted indazoles has highlighted the impact of steric and electronic effects on *N*-1/*N*-2 regioisomeric distribution. For example, employing C-7 NO_2_ or CO_2_Me substituted indazoles conferred excellent *N*-2 regioselectivity (≥ 96%). Importantly, we show that this optimized *N*-alkylation procedure tolerates a wide structural variety of alkylating reagents, including primary alkyl halide and secondary alkyl tosylate electrophiles, while maintaining a high degree of *N*-1 regioselectivity.

## Introduction

**Figure 1:**
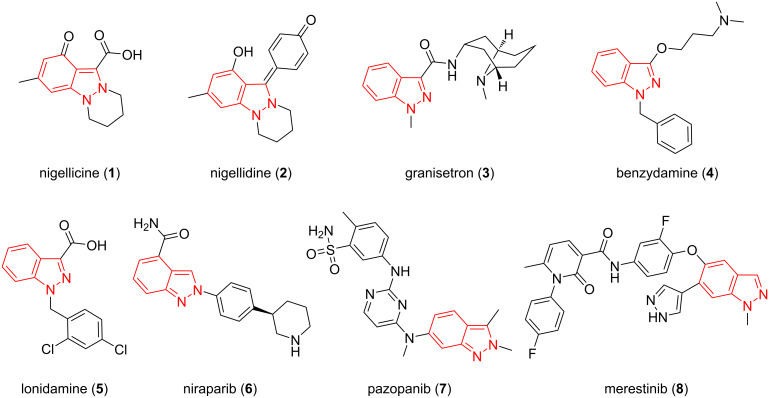
Examples of indazole natural products (**1** and **2**) and synthetic biologically active indazole derivatives (**3**–**8**).

Indazole (benzo[*c*]pyrazole) is an aromatic bicyclic heterocycle and can be viewed as a (bio)isostere of indole [1]. While only a few naturally occurring indazoles have been reported in the literature [2–4], such as the alkaloids nigellicine (**1**) and nigellidine (**2**), there are a myriad of synthetic indazole derivatives known that display a broad range of biological activities. For example, several *N*-1 and *N*-2-substituted indazoles are currently marketed or under clinical investigation for the treatment of nausea and vomiting (granisetron (**3**)) [5]**,** inflammation (benzydamine (**4**)) [6], or certain cancers (lonidamine (**5**), niraparib (**6**), pazopanib (**7**), and merestinib (**8**)) [7–8] (Figure 1). Considering the medicinal significance of *N*-substituted indazole derivatives [9], it would be of great synthetic value to further develop regioselective methods for the preparation of *N*-1 or *N*-2 substituted indazoles.

**Scheme 1:**
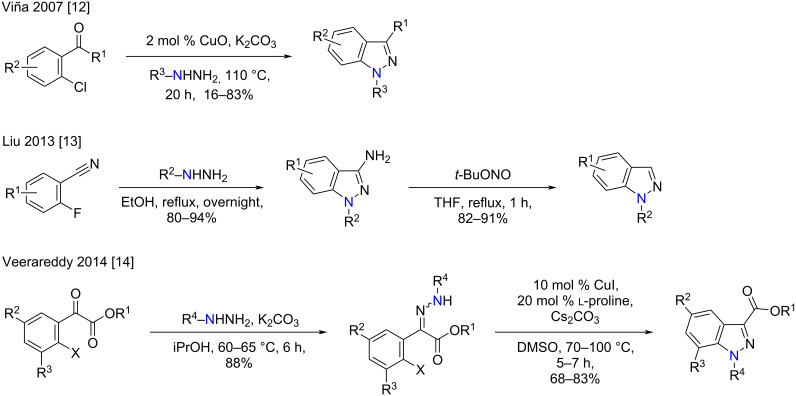
Synthetic approaches to *N*-1 substituted indazole derivatives [12–14].

General approaches to the synthesis of *N*-1 or *N*-2 substituted indazoles involve the incorporation of the *N*-substituent prior to, or following, indazole ring-closure [10–11]. For example, several reports have highlighted the use of *N*-alkyl or *N*-arylhydrazines in the regioselective synthesis of 1*H*-indazoles, from the corresponding *ortho*-haloaryl carbonyl or nitrile, in good to excellent yield (Scheme 1) [12–14].

Alternative strategies to achieve regioselective indazole *N*-alkylation have exploited the noted difference in reactivity between the *N*-1 and *N*-2 atom of the indazole scaffold [15], as the 1*H*-indazole tautomer is typically considered to be more thermodynamically stable than the corresponding 2*H*-tautomer [16]. Using appropriate α-halo carbonyl electrophiles, Hunt et al. have shown that regioselective indazole *N*-alkylation can be achieved through an equilibration process which favours the thermodynamic *N*-1 substituted product [17].

Regioselective indazole *N*-acylation has been suggested to provide the *N*-1 substituted regioisomer, via isomerisation of the corresponding *N*-2 acylindazole to the more stable *N*-1 regioisomer [18]. Similarly, *N*-1 substituted indazoles have been obtained through thermodynamic equilibration, using β-halo ester electrophiles, in the presence of DMF [19]. These latter findings have been utilized to great effect by Conrow et al. to give regioselective access to *N*-1 alkylindazoles on kilogram scale, albeit over two steps from the corresponding *N*-1 acylindazole via reductive acetylation–deacetoxylation [20]. Although electronic and steric factors can influence the regiochemical outcome of indazole *N*-alkylation, varying reaction conditions, such as the choice of base [17,21], acid [22], solvent, and/or *N*-alkylating reagent may also facilitate regioselective indazole *N*-alkylation [23–25]. Bookser et al. have investigated the *N*-alkylation of related bicyclic azolo-fused-ring heterocycles, including 1*H*-indazole, employing NaHMDS in tetrahydrofuran (THF) or dimethyl sulfoxide (DMSO), and observed solvent-dependent regioselectivity [24]. Mechanistic hypotheses, based on elegant experimentation, were proposed to underline the roles that tight and solvent-separated ion pairs played in the observed trend in regioselectivity [24].

Our work sought to further explore the effect of C-3 substitution on *N*-alkylation selectivity control of the 1*H*-indazole scaffold. In view of these antecedents, it was envisioned that the development of a regioselective protocol for indazole *N*-1 alkylation would provide an improved and cost-effective approach to *N*-1 substituted indazole precursors as part of drug discovery and development campaigns.

## Results and Discussion

Working towards the synthesis of a library of novel 1,3-disubstituted indazole derivatives necessitated us to develop a regioselective method that would permit the installation of a wide variety of alkyl sidechains at the *N*-1 position of methyl ester **9** (Table 1). Considering the reported influence of the reaction solvent and/or base on the regiochemical outcome of indazole *N*-alkylation [17,21–22,24], our initial efforts focused on examining the effect of varying these reaction parameters, using *n*-pentyl bromide as the prototypical *N*-alkylating reagent (Table 1).

**Table 1:** Effect of base, solvent, and temperature.^a^

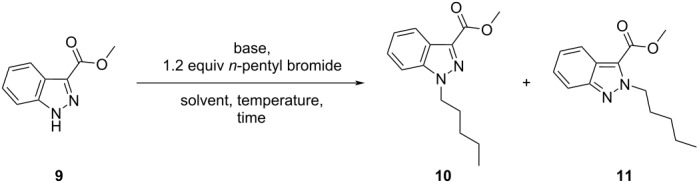									

entry	base	equiv	solvent	*T* (°C)	time (h)	conv.^b^ (%)	ratio^b^ **10**:**11**	yield (%)^c^	

								**10**	**11**

1	Cs_2_CO_3_	3	DMF	rt	16	100	1.4:1	50	39
2	Cs_2_CO_3_	3	DMF	rt	24	100	1.2:1	44	33
3	Cs_2_CO_3_	1.5	DMF	rt	16	100	1.4:1	54	39
4	K_2_CO_3_	1.5	DMF	rt	16	100	1.4:1	60	37
5	Na_2_CO_3_	1.5	DMF	rt	16	34	1.6:1	17	10
6	K_2_CO_3_	0.5	DMF	rt	16	62	1.5:1	33	23
7	K_2_CO_3_	1.5	THF	rt	16	0	–^d^	0	0
8	Na_2_CO_3_	1.5	THF	rt	16	0	–^d^	0	0
9	Cs_2_CO_3_	1.5	MeCN	rt	16	67	1.9:1	29	15
10	Cs_2_CO_3_	1.5	MeCN	rt	30	100	1.9:1	57	29
11	Cs_2_CO_3_	1.5	DMSO	rt	16	100	1.6.1	60	36
12	K_2_CO_3_	1.5	DMSO	rt	16	100	1.6:1	–^d^	–^d^
13	Cs_2_CO_3_	1.5	toluene	rt	16	0	–^d^	0	0
14	Cs_2_CO_3_	1.5	1,4-dioxane	rt	16	0	–^d^	0	0
15	DBU	1.1	CH_2_Cl_2_	rt	16	59	3.2:1	–^d^	–^d^
16	DBU	1.1	THF	rt	16	65	2.7:1	–^d^	–^d^
17	DBU	1.1	DMF	rt	16	72	1.4:1	–^d^	–^d^
18	*t*-BuOK	1.1	THF	^e^	16	30	16:1	–^d^	–^d^
19	*t*-BuOK	1.1	THF	^e^	48	70	16:1	–^d^	–^d^
20	NaH	1.1	THF	^e^	48	57	>99:1	44	0
21	*t*-BuOK	1.1	THF	^f^	5.5	100	13:1	–^d^	–^d^
22	NaH	1.1	THF	^f^	24	100	>99:1	89	0

^a^Reaction scale = 1.4 mmol (**9**); ^b^determined using ^1^H NMR (see Supporting Information File 1); ^c^isolated yield; ^d^not determined; ^e^0 °C → rt; ^f^0 °C → 50 °C; conv. = conversion of **9** to **10** and **11** only; rt ≈ 20 °C.

Early investigations revealed that the combination of cesium carbonate (Cs_2_CO_3_) in dimethylformamide (DMF) at room temperature (≈ 20 °C) afforded a mixture of *N*-1 and *N*-2 regioisomers (**10** and **11**, respectively), with only partial preference for the desired *N*-1 regioisomer **10** (Table 1, entry 1). Furthermore, increasing the reaction time or decreasing the number of equivalents of Cs_2_CO_3_ did not appear to influence the regiochemical outcome of the reaction (Table 1, entries 2 and 3, respectively). Substituting potassium carbonate (K_2_CO_3_) for Cs_2_CO_3_ did not show any improvement in the regioisomeric distribution of **10** and **11** (ratio *N*-1 (**10**)/*N*-2 (**11**) = 1.4:1) (Table 1, entry 4). Similarly, the use of sodium carbonate under identical conditions gave a notably lower combined yield of **10** and **11** (27%), due to poor conversion (34%) (Table 1, entry 5). Attempts to reduce the amount of K_2_CO_3_ base to 0.5 equivalents, with respect to indazole **9**, (Table 1, entry 6) resulted in incomplete conversion (62%) and provided no significant change in *N*-1 regioselectivity (ratio *N*-1:*N*-2 = 1.5:1). Importantly, using THF as the reaction solvent with potassium or sodium carbonate bases failed to give the *N*-alkylated products **10** or **11** (Table 1, entries 7 and 8, respectively).

Further variation of the reaction solvent revealed no significant improvement in *N*-1 regioselectivity, when using acetonitrile (MeCN) (Table 1, entries 9 and 10) or DMSO (Table 1, entries 11 and 12) (ratio *N*-1:*N*-2 = 1.9:1 and 1.6:1, respectively). Furthermore, employing toluene or 1,4-dioxane as the reaction solvent failed to provide regioisomer **10** or **11** (Table 1, entries 13 and 14). The latter observation may be due to the restricted solubility of Cs_2_CO_3_ in toluene and 1,4-dioxane [24]**.** Using K_2_CO_3_ in MeCN, Longworth et al. have obtained **10** with a similar degree of *N*-1 regioselectivity (ratio *N*-1:*N*-2 = 2.8:1) [26]. However, altering solvent polarity when employing 1,8-diazabicyclo[5.4.0]undec-7-ene (DBU) as a base (Table 1, entries 15–17) positively influenced the regioselectivity, albeit with poor conversion (59–72%) when compared with other inorganic carbonate bases (vide supra).

**Scheme 2:**
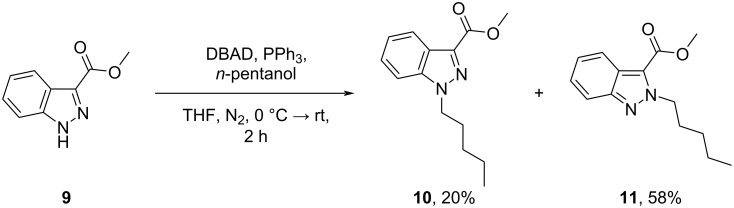
*N*-Alkylation of indazole **9** under Mitsunobu conditions shows a strong preference (ratio *N*-1 (**10**):*N*-2 (**11**) = 1:2.5) for the formation of *N*-2 regioisomer **11** (58%, isolated yield) over the corresponding *N*-1 regioisomer **10** (20% isolated yield).

Despite poor conversion (30%), the use of the strong alkoxide base, potassium *tert*-butoxide, in tetrahydrofuran (THF) gave an appreciably higher degree of *N*-1 regioselectivity (94%, *N*-1 regioselectivity) (Table 1, entry 18) than the previously investigated reaction conditions (55–76%, *N*-1 regioselectivity) (Table 1, entries 1–17). To improve conversion to the desired *N*-1 regioisomer **10**, the reaction time was extended (48 h) (Table 1, entry 19) and the reaction temperature increased from room temperature (≈ 20 °C) to 50 °C (Table 1, entry 21). It was found that the latter variation facilitated the complete consumption of **9** with negligible effect on the regiochemical outcome of the reaction. Gratifyingly, sodium hydride (NaH) demonstrated excellent *N*-1 regioselectivity (ratio *N*-1:*N*-2 > 99:1), albeit with poor conversion (57%) (Table 1, entry 20). Again, increasing the preceding reaction temperature from room temperature to 50 °C not only facilitated the complete conversion of methyl ester **9** to desired *N*-1 regioisomer **10**, but also maintained an excellent degree of regiocontrol (ratio *N*-1:*N*-2 > 99:1) (Table 1, entry 22). The *N*-alkylation of methyl ester **9** was also investigated under Mitsunobu conditions (see Supporting Information File 1), in the presence of *n*-pentanol (Scheme 2). Notably, the latter conditions demonstrated a strong (ca. three-fold) preference for the formation of the corresponding *N*-2 regioisomer **11** (ratio *N*-1:*N*-2 = 1:2.5) rather than the desired *N*-1 regioisomer.

**Figure 2:**
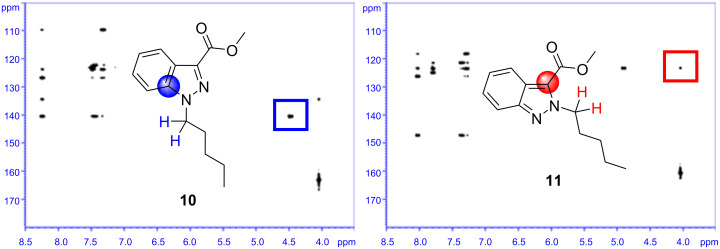
Observation of a ^1^H–^13^C correlation between the C-7a (blue circle) or C-3 (red circle) atom of the indazole ring and the *N*-*n*-pentyl CH_2_ proton pair proximal to N atoms of the indazole nucleus, using (^1^H–^13^C) HMBC NMR experiments, permitted the regiochemical assignment of *N*-1 and *N*-2 substituted regioisomers **10** and **11**, respectively.

To assign the regiochemistry of isolated *N*-1 and *N*-2 substituted indazole isomers, a combination of one and two-dimensional NMR experiments (particularly, heteronuclear multiple bond correlation (HMBC)) was employed [27]. For example, (^1^H–^13^C) HMBC analysis of *N*-1 regioisomer **10** shows a ^1^H–^13^C correlation between the C-7a carbon of the indazole ring and the *n*-pentyl CH_2_ proton pair proximal to the indazole *N*-1 atom (Figure 2). No evident ^1^H–^13^C correlation was observed between the *n*-alkyl CH_2_ proton pair proximal to the indazole *N*-1 atom and the indazole C-3 atom, for *N*-1 substituted indazole regioisomer **10**. Conversely, (^1^H–^13^C) HMBC analysis of *N*-2 substituted regioisomer **11**, revealed a ^1^H–^13^C correlation between the alkyl CH_2_ proton pair (proximal to the indazole *N*-2 atom) and the C-3 carbon of the indazole heterocycle, while no ^1^H–^13^C correlation was observed between the alkyl CH_2_ proton pair and the C-7a carbon atom of the indazole ring.

**Figure 3:**
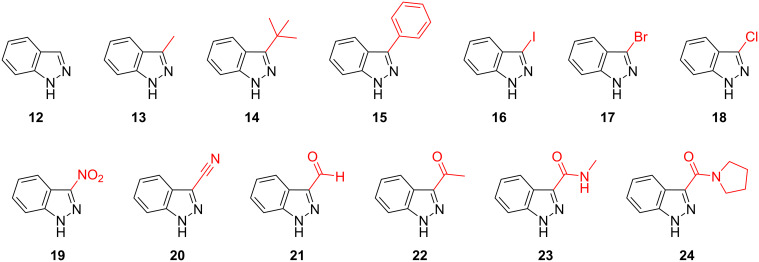
C-3 substituted indazole derivatives (**12**–**24**) employed to investigate C-3 substituent effects on indazole *N*-alkylation regioselectivity.

With a set of optimal conditions for the regioselective *N*-1 alkylation of methyl ester **9** in hand (Table 1, entry 22), our attention turned to probing the influence of a variety of indazole C-3 substituents on the regiochemical *N*-alkylation outcome. A series of C-3 substituted indazoles (**12**–**24**) (Figure 3) were thus assembled [28–33] to investigate the effects of electronic and steric factors on indazole *N*-alkylation (Table 2) using our optimized conditions from Table 1 (entry 22) (referred to as “conditions A” in Table 2).

As literature precedence shows, the combination of Cs_2_CO_3_ in DMF has been commonly employed to achieve indazole *N*-alkylation [17,34–35]. For comparison with our previously optimized *N*-alkylation protocol (Table 1, entry 22; conditions A), conditions that provided less favorable *N*-1 regioselectivity (Table 1, entry 3, henceforth referred to as “conditions B” in Table 2) were also included as a part of this investigation.

**Table 2:** Indazole C-3 substituent effects.

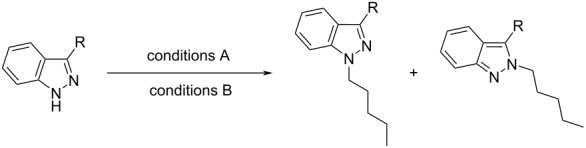									

entry	R	product		cond. A^a,c^	yield (%)^d^		cond. B^b,c^	yield (%)^d^	
					
		*N*-1	*N*-2	ratio *N*-1:*N*-2^e^	*N*-1	*N*-2	ratio *N*-1:*N*-2^e^	*N*-1	*N*-2

1	H	**25**	**26**	1:1.3	33	46	1.7:1	43	28
2	Me	**27**	**28**	2.3:1	54	22	4.6:1	73	12
3	*t*-Bu	**29**	**30**	>99:1	85	0	>99:1	79	0
4	Ph	**31**	**32**	7.8:1	54	11	14:1	90	6
5	I	**33**	**34**	3.8:1	60	14	4.4:1	53	16
6	Br	**35**	**36**	5.9:1	83	12	7.5:1	85	12
7	Cl	**37**	**38**	5.6:1	65	7	8.0:1	75	9
8	NO_2_	**39**	**40**	83:1	80	0	6.4:1	57	9
9	CN	**41**	**42**	3.4:1	–^f^	– ^f^	5.2:1	– ^f^	– ^f^
10	CHO	**43**	**44**	16:1	– ^f^	– ^f^	9.8:1	– ^f^	– ^f^
11	COMe	**45**	**46**	>99:1	97^g^	0^g^	61:1	66^g^	0^g^
12	CONHMe	**47**	**48**	>99:1	88	0	>99:1	93	0
13	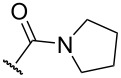	**49**	**50**	>99:1	53	0	>99:1	93	0

^a^1.1 equiv NaH, 1.2 equiv *n*-pentyl bromide, THF, N_2_, 0 °C → 50 °C, 24 h; ^b^1.5 equiv Cs_2_CO_3_, 1.2 equiv *n*-pentyl bromide, DMF, rt, 16 h; ^c^reaction scale = 1 mmol (appropriate indazole); ^d^isolated yield; ^e^determined using ^1^H NMR (see Supporting Information File 1); ^f^not determined, due to the formation of an inseparable mixture (using wet flash column chromatography) of the corresponding *N*-1 and *N*-2 regioisomers; ^g^reaction scale = 0.5 mmol (**22**); cond. = conditions.

Increasing alkyl and aryl steric bulk at the indazolic C-3 position seems to favor *N*-1 regioisomer formation in the order *t*-Bu > Ph > Me > H, for both conditions A and B (Table 2, entries 1–4). Furthermore, having a sterically demanding *t*-Bu group at the indazole C-3 position (**14**) gave the *N*-1 substituted regioisomer **29** exclusively under both conditions A and B, respectively (Table 2, entry 3). Apart from the 1*H*-indazole scaffold [36–37], the steric influence of adjacent substituent(s) on *N*-alkylation regioselectivity has previously been described for other nitrogen-containing heterocycles, such as pyrazole [38], purine, and related 1,3-azoles [39]. Although the *N*-alkylation of indazole **12,** using conditions A (NaH in THF), proceeded with poor regioselectivity (ratio *N*-1 (**25**):*N*-2 (**26**) = 1:1.3), Bookser et al. have obtained a similar regioselective outcome using a combination of NaHMDS and MeI instead of NaH and *n*-pentyl bromide (Table 2, entry 1), respectively [24].

The presence of a halogen atom (I, Br, or Cl) at the C-3 position of the indazole scaffold (**16**–**18**) revealed no significant trend in regioselectivity (Table 2, entries 5–7). The latter observed preference for the formation of the corresponding *N*-1 regioisomer (**33**, **35**, and **37**, respectively), under both conditions A and B, highlights the steric influence of the halogen C-3 substituent. Furthermore, Bookser et al. have noted comparable regioselectivity with related C-3 bromo substituted N-containing heterocycles, under similar reaction conditions [24]. Marked *N*-1 regioselectivity was achieved with 3-nitro substituted indazole **19**, when using NaH in THF (conditions A) (ratio *N*-1 (**39**):*N*-2 (**40**) = 83:1) (Table 2, entry 8). However, the same degree of *N*-1 regioselectivity was not observed for indazole **19**, when using Cs_2_CO_3_ in DMF (conditions B) (ratio *N*-1 (**39**):*N*-2 (**40**) = 6.4:1) (Table 2, entry 8). Interestingly, the presence of an electronegative nitrile group at the indazole C-3 position (**20**) provided only modest *N*-1 regioselectivity, under both conditions A and B (*N*-1 (**41**):*N*-2 (**42**) = 3.4:1 and 5.2:1, respectively) (Table 2, entry 9).

Remarkably, unlike methyl ester **9** (vide supra), indazoles **12**, **13**, **15**–**18**, and **20** all demonstrated a higher preference for *N*-1 indazole alkylation under conditions B, when compared with conditions A (Table 2, entries 1, 2, 4–7, and 9). Furthermore, C-3 ketone (**22**) and amide (**23** and **24**) substituted indazoles also gave the corresponding *N*-1 regioisomers with a high degree of *N*-1 regioselectivity (ratio *N*-1:*N*-2 = 61:1 for indazole **22** and > 99:1 for indazoles **23** and **24**), when employing Cs_2_CO_3_ in DMF (Table 2, entries 11–13). The latter exclusive formation of the corresponding *N*-1 regioisomer observed for indazoles **22** and **23** under conditions B may arise from the steric repulsion of the electrophile from the *N*-2 position to the *N*-1 atom by the indazole C-3 substituent (vide infra).

While the corresponding *N*-1 and *N*-2 regioisomers arising from the *N*-alkylation of C-3 substituted indazoles **12**–**24** were generally amenable to separation using wet flash column chromatography, the corresponding *N*-1- and *N*-2-*n*-pentylindazole derivatives of both indazoles **20** and **21** were largely inseparable (Table 2, entries 9 and 10, respectively). However, preparative thin-layer chromatography (PTLC) did permit the isolation of an enriched sample of the *N*-1-*n*-pentyl substituted derivative of indazole **20** for (^1^H–^13^C) HMBC NMR analysis and confirmatory regiochemical assignment. Similarly, while the regioisomeric products arising from the *N*-alkylation of indazole **21** could not be separated using wet flash column chromatography or PTLC, a significantly enriched sample of the corresponding *N*-1 regioisomer (ratio *N*-1 (**43**):*N*-2 (**44**) = 16:1; Table 2, entry 10) was obtained under conditions A.

Notably, under conditions A (NaH in THF), C-3 substituted indazoles **19** (-NO_2_), **21** (-CHO), **22** (-COMe), **23** (-CONHMe), and tertiary amide **24** all demonstrated a high degree of *N*-1 regioselectivity (ratio *N*-1:*N*-2 = 16:1 (**21**), 83:1 (**19**), > 99:1 (**22**, **23**, and **24**), respectively) (Table 2, entries 8 and 10–13). We postulate that these observed preferences for the generation of the *N*-1 regioisomer, under conditions A, may be due to the formation of a tight ion pair involving the indazole *N*-2 atom and C-3 substituents which are capable of cation chelation via the *N*-2 atom electron lone pair and the C-3 substituent X=O functionality, respectively [24]. Tight ion pair formation with the sodium cation and both the *N*-2 atom and C-3 substituents of the indazole scaffold likely hinders the approach of the electrophile to *N*-2 and directs alkylation to the *N*-1 position. Furthermore, this effect is not observed for indazoles bearing C-3 substituents that cannot participate in the formation of tight ion pairs (such as, **12**–**18** and **20**), under conditions A (NaH in THF). The latter *N*-1 regioselectivity conferred through tight ion pair formation is augmented by the steric effect that the C-3 substituent enforces. The high degree of *N*-1 regioselectivity obtained for indazoles bearing bulky substituents at the C-3 position that are not capable of engaging in tight ion pair formation, such as **14** and **15**, further highlights the influence of steric effects on regioselectivity.

To determine if the high degree of *N*-1 regioselectivity (ratio *N*-1:*N*-2 > 99:1) observed when employing conditions A (see Table 1 and Table 2) was due to base (NaH) or solvent (THF) effects, Cs_2_CO_3_ was substituted for NaH (Table 3, entries 1 and 2). Although the *N*-alkylation of indazole **9** was hindered by poor conversion (9%) when carried out at room temperature, the complete conversion of **9** to regioisomers **10** and **11** was observed and *N*-1 regioselectivity maintained upon increasing the reaction temperature to 50 °C (Table 3, entries 1 and 2).

**Table 3:** Effect of NaH and THF on *N*-1 regioselectivity.^a^

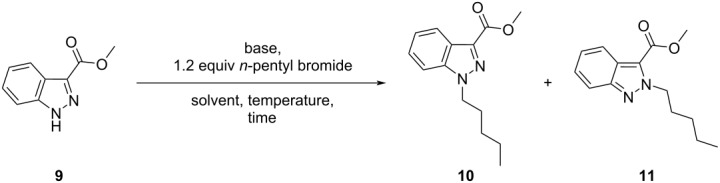							

entry	base	equiv	solvent	*T* (°C)	time (h)	conv.^b^ (%)	ratio^b^ **10**:**11**

1	Cs_2_CO_3_	1.5	THF	rt	16	9	5.8:1
2	Cs_2_CO_3_	1.5	THF	^c^	24	100	6.8:1
3	NaH	1.1	DMF	rt	16	100	1.9:1
4	NaH	1.1	DMF	^c^	24	100	1.8:1
5	LiH	1.1	THF	^c^	24	18	28:1
6	KH	1.1	THF	^c^	24	100	11:1
7	NaHMDS	1.1	THF	^c^	24	91	>99:1
8	NaNH_2_	1.1	THF	^c^	24	69	62:1
9	LDA	1.1	THF	^c^	24	26	>99:1

^a^Reaction scale = 1 mmol (**9**); **^b^**determined using ^1^H NMR (see Supporting Information File 1); ^c^0 °C → 50 °C; conv. = conversion of **9** to **10** and **11** only; rt ≈ 20 °C.

Employing a combination of NaH and DMF (Table 3, entries 3 and 4) caused a significant drop-off in *N*-1 regioselectivity (> 30%), when compared with NaH/THF (conditions A; Table 1, entry 22). Bookser et al. have reported a similar decline in *N*-1 regioselectivity for the alkylation of 1*H*-indazole with MeI when using a combination of NaHMDS and DMSO instead of THF as the reaction solvent [24]. It is likely that DMF similarly facilitates the formation of solvent-separated ion pairs which serve to diminish the high *N*-1 regioselectivity previously achieved when using THF (Table 3, entry 2), where tight ion pair formation between the cesium cation, *N*-2 atom, and chelating X=O C-3 group of the indazole ring predominates. Although the C-3 methyl carboxylate group of **9** may contribute to selective *N*-1 alkylation through steric effects, the use of DMF as the reaction solvent does not support tight ion pair formation and diminishes *N*-1 regioselectivity.

Further variation of the alkali metal cation, through the use of strong hydride bases, such as LiH or KH in THF, to examine *N*-1 regioselectivity (Table 3, entries 5 and 6, respectively) revealed a high preference for the formation of *N*-1 regioisomer **10** (ratio *N*-1 (**10**):*N*:2 (**11**) = 28:1 and 11:1, respectively). Similarly, strong amide bases, including NaHMDS, NaNH_2_, and LDA, also furnished *N*-1-alkylindazole **10** with excellent regioselectivity (up to > 98%, *N*-regioisomer), when using THF as the reaction solvent (Table 3, entries 7–9). These latter results indicate that the reaction solvent may play an important role in determining the regiochemical outcome of the *N*-alkylation. For example, the *N*-alkylation of methyl ester **9** under conditions B (Cs_2_CO_3_/DMF) showed no notable preference for the formation of *N*-1 regioisomer **10** (ratio *N*-1 (**10**):*N*-2 (**11**) = 1.4:1) (Table 1, entry 3), however, using a combination of Cs_2_CO_3_ and THF demonstrated marked *N*-1 regioselectivity (ratio *N*-1 (**10**):*N*-2 (**11**) = 5.8:1) (Table 3, entry 1).

To investigate the effect of the position of the indazole C-3 substituent on regiochemical outcome, several C-7 substituted indazoles (Me-, Br-, NO_2_-, and CO_2_Me) were alkylated using both conditions A (NaH in THF) and B (Cs_2_CO_3_ in DMF) (Table 4, entries 1–4). A significant reversal in regioselectivity was observed under conditions A for C-7 Me and Br-substituted indazoles, when compared with their analogous C-3 substituted counterparts (Table 2, entries 2 and 6), favouring the formation of the corresponding *N*-2 regioisomer (Table 4, entries 1 and 2). This latter preference for *N*-2 alkylation is likely due to the proximal steric bulk of both the C-7 Me and Br substituents, respectively, to the *N*-1 position.

**Table 4:** Effect of indazole benzenoid ring substituents on *N*-1:*N*-2 regioselectivity.^a^

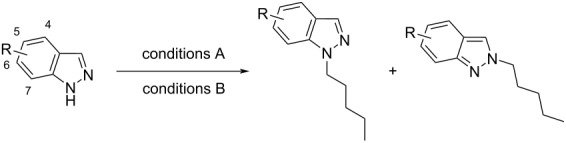									

entry	R	product		cond. A^b,d^	yield (%)^e^		cond. B^c,d^	yield (%)^e^	
					
		*N*-1	*N*-2	ratio *N*-1:*N*-2^e^	*N*-1	*N*-2	ratio *N*-1:*N*-2^e^	*N*-1	*N*-2

1	7-Me	**51**	**52**	1:3.0	19	71	1:1.2	46	47
2	7-Br	**53**	**54**	1:7.3	13	76	1:1.3	56	35
3	7-NO_2_	**55**	**56**	1:>99	0	81	1.1:1	50	44
4	7-CO_2_Me	**57**	**58**	1:25	0	77	2.6:1	69	25
5	6-CO_2_Me	**59**	**60**	1:2.2	22	55	1.7:1	61	35
6	5-CO_2_Me	**61**	**62**	1:1.3	31	35	1.9:1	60	32
7	4-CO_2_Me	**63**	**64**	1:1.3	28	34	1.3:1	55	26

^a^Reaction scale = 1 mmol (appropriate indazole); ^b^1.1 equiv NaH, 1.2 equiv *n*-pentyl bromide, THF, N_2_, 0 °C → 50 °C, 24 h; ^c^1.5 equiv Cs_2_CO_3_, 1.2 equiv *n*-pentyl bromide, DMF, rt, 16 h; ^d^determined using ^1^H NMR (see Supporting Information File 1); ^e^isolated yield; cond. = conditions.

The presence of a nitro or methyl carboxylate group at the C-7 position of the indazole core facilitated excellent *N*-2 regioselectivity under conditions A (Table 4, entries 3 and 4). To further examine the positional effect of benzenoid ring substitution on *N*-alkylation regioselectivity, 6-, 5-, and 4-CO_2_Me substituted indazole derivatives were alkylated, under conditions A and B (Table 4, entries 5–7). While C-4 and C-5 substituted indazole methyl esters showed no apparent *N*-1 or *N*-2 regioselectivity under conditions A (ratio *N*-1:*N*-2 of both **61**:**62** and **63**:**64** = 1:1.3) (Table 4, entries 6 and 7), the corresponding C-6 substituted indazole methyl ester demonstrated a notable preference for *N*-2 alkylation (ratio *N*-1 (**59**):*N*-2 (**60**) = 1:2.2) (Table 4, entry 5). Importantly, the remarkable *N*-2 regioselectivity observed for C-7 NO_2_ and CO_2_Me substituted indazoles (≥ 96%) under conditions A provides further support for the role that tight ion pair formation may play in achieving regioselective *N*-alkylation (vide supra).

*N*-Alkylation of C-7 Me, Br, or NO_2_ substituted indazoles, using conditions B (Cs_2_CO_3_ in DMF), showed an overall loss of *N*-2 regioselectivity (Table 4, entries 1–3), when compared with the corresponding regiochemical outcomes obtained under conditions A (NaH in THF) (Table 4, entries 1–3). However, the *N*-alkylation of C-4, -5, -6, and -7 CO_2_Me substituted indazoles, in the presence of Cs_2_CO_3_ in DMF, all showed a preference for the formation of the corresponding *N*-1 regioisomer (Table 4, entries 4–7).

**Scheme 3:**
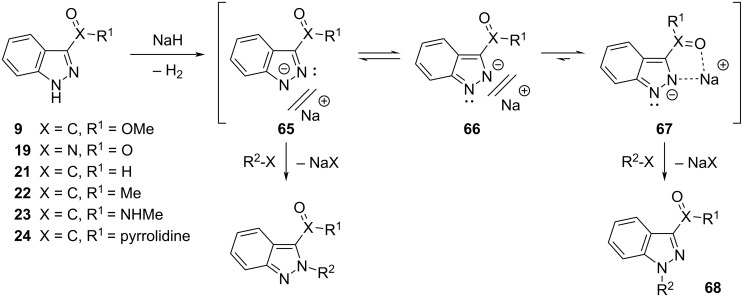
Proposed mechanism for the regioselective *N*-1 alkylation of indazoles **9**, **19**, and **21**–**24** in the presence of NaH in THF (conditions A).

Mechanistically, we postulate that our optimized regioselective *N*-1 alkylation of the exemplar methyl ester **9** and other appropriately C-3 substituted indazoles (**19**, or **21**–**24**) (under conditions A, Table 1**,** entry 22) involves the initial irreversible deprotonation of the indazole in the presence of NaH to initially give indazolyl salt **65** which is in equilibrium with its alternate anionic form **66** (Scheme 3). Through tautomerization, salt **66** may then form a tight ion pair with a sodium cation, via the *N*-2 atom and X=O containing C-3 substituent of the indazole nucleus, affording species **67** whose existence dominates in THF as solvent. The formation of **67** is then followed by the nucleophilic substitution of alkylating reagent R^2^–X to selectively give the desired *N*-1 regioisomer **68**. It is likely that a mixture of the solvent-separated ion pairs **65** and **66** predominate, when using polar solvents such as DMF [24]. Furthermore, precluding tight ion pair formation through the use of DMF, may prompt indazole *N*-alkylation to fall predominantly under steric control, resulting in diminished *N*-alkylation regioselectivity.

To further probe the potential influence of the alkali metal cation on the regioselective *N*-1 alkylation of indazole methyl ester **9**, a control experiment was carried out, using 1 equivalent of the ether 15-crown-5 (with respect to NaH) (Table 5). Chelation of the sodium cation with the crown ether should disrupt the formation of tight ion pairs (Scheme 2) and attenuate *N*-1 regioselectivity. The presence of 15-crown-5 caused a notable reduction in *N*-1 regioselectivity, when compared with results obtained in the absence of the crown ether (ratio *N*-1 (**10**):*N*-2 (**11**) = 9.6:1 (Table 5, entry 1) versus > 99:1 (Table 1, entry 22)). Similarly, to disqualify any potential inherent effect of the crown ether on the regioselective outcome, increasing the number of equivalents of 15-crown-5 from one to five equivalents (with respect to NaH) gave a similar regioisomeric distribution of *N*-substituted indazoles **10** and **11** (Table 5, entry 2). These control experiments show that 15-crown-5 disrupts tight ion pair formation, providing further support for our mechanistic proposal that under conditions A (NaH/THF), tight ion pair formation directs regioselective *N*-1 alkylation.

**Table 5:** Effect of 15-crown-5 on the regioselective *N*-alkylation of indazole **9**, in the presence of NaH in THF.^a^

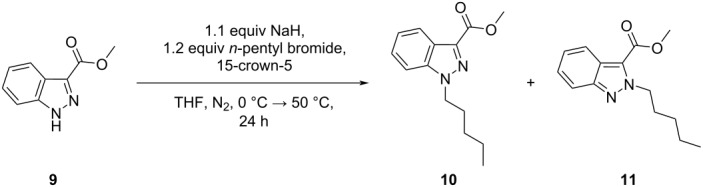					

entry	equiv 15-C-5	conv.^b^ (%)	ratio **10**:**11**^b^	yield (%)^c^	

				**10**	**11**

1	1.1	100	9.6:1	77	6
2	5.5	100	6.0:1	71	10

^a^Reaction scale = 1 mmol (**9**); ^b^determined using ^1^H NMR (see Supporting Information File 1); ^c^isolated yield; conv. = conversion of **9** to **10** and **11** only.

To demonstrate the scope of our optimized *N*-1 regioselective *N*-alkylation protocol (conditions A), methyl ester-substituted indazole **9** was subjected to a series of alkylating reagents under both conditions A and B (Table 6). The high selectivity observed for *N*-1 alkylation using NaH in THF (conditions A) was mainly effective using primary halide and tosylate compounds as electrophiles. Similar to the regiospecificity observed when employing *n*-pentyl bromide (ratio *N*-1 (**10**):*N*-2 (**11**) > 99:1, Table 1, entry 22), its tosylate counterpart gave the corresponding *N*-1 regioisomer **10** with a high degree of *N*-1 regioselectivity (ratio *N*-1 (**10**):*N*-2 (**11**) = 76:1) under conditions A (Table 6, entry 1). Furthermore, conditions A could be successfully applied to the synthesis of benzyl and alicyclic indazole derivatives **69**–**74** (Table 6, entries 2–6), affording the *N*-1 regioisomer almost exclusively. Notwithstanding excellent *N*-1 regioselectivity when using conditions A (ratio *N*-1 (**73**):*N*-2 (**74**) > 99:1), the yield of the corresponding *N*-1 substituted cyclohexylmethylindazole **73** was significantly reduced, due to poor conversion (13%, combined *N*-1 and *N*-2 (see Supporting Information)) (Table 5, entry 5). However, employing the corresponding tosylate under identical conditions (NaH in THF (conditions A)) permitted improved conversion to the desired *N*-1 substituted alicyclic indazole **73** (78%, combined *N*-1 and *N*-2 (see Supporting Information File 1)), whilst maintaining excellent *N*-1 regioselectivity (ratio *N*-1 (**73**):*N*-2 (**74**) = 70:1) (Table 6, entry 6).

**Table 6:** Alkylating reagent effects on *N*-1/*N*-2 regioselectivity.^a^

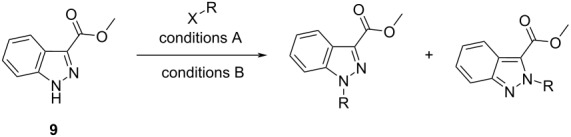										

entry	R	X	product		cond. A^b,c^	yield (%)^d^		cond. B^c,e^	yield (%)^d^	
					
			*N*-1	*N*-2	ratio *N*-1:*N*-2^c^	*N*-1	*N*-2	ratio *N*-1:*N*-2^c^	*N*-1	*N*-2

1	*n*-pentyl	OTs	**10**	**11**	76:1	88	0	1.6:1	58	37
2	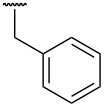	Br	**69**	**70**	>99:1	90	0	2.1:1	60	22
3	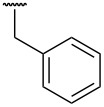	OTs	**69**	**70**	>62:1	85	3	2.1:1	52	25
4	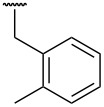	Br	**71**	**72**	>99:1	82	0	3.8:1	77	21
5	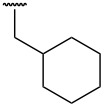	Br	**73**	**74**	>99:1	7^f^	0	1.5:1	35^f^	23^f^
6	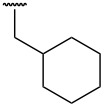	OTs	**73**	**74**	70:1	63^f^	0	2.0:1	40^f^	16^f^
7	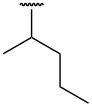	Br	**75**	**76**	>99:1	3^f^	0	1:1.1	49	44
8	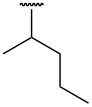	OTs	**75**	**76**	25:1	81	2	1.1:1	43	38
9	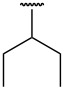	Br	**77**	**78**	>99:1	4^f^	0	1.0:1	48	46

^a^Reaction scale = 1 mmol (**9**); ^b^1.1 equiv NaH, 1.2 equiv R–X, THF, N_2_, 0 °C → 50 °C, 24 h; ^c^determined using ^1^H NMR (see Supporting Information File 1); ^d^isolated yield; cond. = conditions; ^e^1.5 equiv Cs_2_CO_3_, 1.2 equiv R–X, DMF, rt, 16 h; ^f^incomplete conversion of **9** to the corresponding *N*-1 and/or *N*-2 regioisomer.

In the presence of NaH in THF (conditions A), secondary alkyl bromides, such as 2- and 3-bromopentanes, both gave their corresponding *N*-1 alkylindazoles **75**–**78** in only trace amounts (< 5% isolated yield, Table 6, entries 7 and 9), due to poor conversion (< 5%, combined *N*-1 and *N*-2 (see Supporting Information File 1)). While the latter observation may be due to competing elimination of the alkyl halide under strongly basic conditions [40], the use of a secondary tosylate electrophile under conditions A (Table 6, entry 8) furnished the desired *N*-1 regioisomer **75** in very good isolated yield (81%). These latter results would suggest that secondary alkyl tosylates are more suitable than their corresponding halide counterparts, for *N*-1 regioselective alkylation, under these investigated conditions. Conversely, the use of Cs_2_CO_3_ in DMF (conditions B) afforded approximately equal amounts of the corresponding *N*-1 and *N*-2 regioisomers **75**–**78** when using the aforementioned secondary alkyl bromides, with complete consumption of indazole **9** observed (Table 6, entries 7 and 9). However, *N*-1 regioselectivity is absent (ratio **75**:**76** and **77**:**78** ≈ 1:1, Table 6, entries 7–9) under conditions B, most likely due to solvent-separated ion pair formation.

## Conclusion

Both 1*H*- and 2*H*-indazoles represent a core heterocyclic motif in many therapeutic small molecule drugs. Thus, from a synthetic perspective, the regioselective *N*-alkylation of the indazole scaffold would be of great value to the pharmaceutical industry. Focusing on 3-substituted indazoles, highly selective *N*-1 alkylations can be achieved using NaH in THF (conditions A) and Cs_2_CO_3_ in DMF (conditions B) as applied to primary and secondary alkyl electrophiles. When compared with conditions A, the use of Cs_2_CO_3_ in DMF (conditions B) demonstrated improved regioselectivity for the corresponding *N*-1 regioisomers of unsubstituted (**12**) and C-3 methyl (**13**), phenyl (**15**), halo (**16**–**18**), and cyano (**20**) substituted indazoles. Investigating the effect on the regioisomeric *N*-1/*N*-2 distribution indicated that steric bulk likely plays a significant role in determining *N*-1 regioselectivity. For example, the ratio of *N*-1/*N*-2 increases in the order of increasing steric bulk at the indazole C-3 position (H < Me < Ph < *t*-Bu). However, when the former protocol (conditions A) was extended to indazoles bearing a nitro (**19**) or carbonyl (**21**–**24**) functional group, the desired *N*-1 regioisomer was obtained with a very high degree of regioselectivity. In the case of C-3 substituted indazoles **9**, **19** and **21**–**24** (those bearing a X=O α to the indazole C-3 position), we postulate that in the presence of NaH, the corresponding indazole salt may form a tight ion pair (**67**) which serves to attenuate *N*-2 alkylation*,* and affords the desired *N*-1 regioisomer exclusively. Furthermore, the excellent *N*-2 regioselectivity (≥ 96%) observed for the *N*-alkylation of C-7 NO_2_ or CO_2_Me indazoles (Table 4, entries 3 and 4) provides further support for the important role that tight ion pair formation plays in directing *N*-alkylation of the indazole scaffold.

## Supporting Information

File 1 Compound synthesis, characterisation, and copies of spectral data pertaining to regioisomeric distribution (*N*-1:*N*-2) determination.
