# Supplementary material for: Regioselective N-alkylation of the 1H-indazole scaffold; ring substituent and N-alkylating reagent effects on regioisomeric distribution
Source: Beilstein J Org Chem. 2021 Aug 2;17:1939–51. doi: 10.3762/bjoc.17.127 (PMC8353588; doi:10.3762/bjoc.17.127)
Supplement: File 1 — Compound synthesis, characterisation, and copies of spectral data pertaining to regioisomeric distribution (N-1:N-2) determination. [file Beilstein_J_Org_Chem-17-1939-s001.pdf]

BEILSTEIN JOURNAL OF ORGANIC CHEMISTRY

Supporting Information

for

Regioselective *N*-alkylation of the 1*H*-indazole scaffold; ring substituent and *N*-alkylating reagent effects on regioisomeric distribution

Ryan M. Alam and John J. Keating

Beilstein J. Org. Chem. **2021,** *17,* 1939–1951. doi:10.3762/bjoc.17.127

Compound synthesis, characterisation, and copies of spectral data pertaining to regioisomeric distribution (*N*-1:*N*-2) determination

License and Terms: This is a supporting information file under the terms of the Creative Commons Attribution License (https://creativecommons.org/licenses/by/4.0). Please note that the reuse, redistribution and reproduction in particular requires that the author(s) and source are credited and that individual graphics may be subject to special legal provisions.
The license is subject to the *Beilstein Journal of Organic Chemistry* terms and conditions: (https://www.beilstein-journals.org/bjoc/terms)

Table of Contents

Experimental Note S6

Synthesis of C-3 substituted 1*H*-indazoles S7

1*H*-Indazole-3-carboxylic acid S7

Methyl 1*H*-indazole-3-carboxylate (**9**) S9

1*H*-indazole (**12**) S11

3-(*o*-Tolyldiazenyl)-1*H*-indazole S13

3-*tert*-Butyl-1*H*-indazole (**14**) S15

3-Phenyl-1*H*-indazole (**15**) S17

3-Iodo-1*H*-indazole (**16**) S19

3-Bromo-1*H*-indazole (**17**) S21

3-Nitro-1*H*-indazole (**19**) S23

1*H*-Indazole-3-carboxaldehyde (**21**) S25

N-Methyl-1*H*-indazole-3-carboxamide (**23**) S27

(1*H*-Indazol-3-yl)(pyrrolidin-1-yl)methanone (**24**) S29

1*H*-indazole *N*-alkylation S31

General Procedure (A): S31

General Procedure (B): S31

Note: Order of Elution of *N*-1 and *N*-2 Regioisomers using Wet Flash Column Chromatography S32

Methyl 1-*n*-pentyl-1*H*-indazole-3-carboxylate (**10**) S32

Methyl 2-*n*-pentyl-2*H*-indazole-3-carboxylate (**11**) S34

N-Alkylation of Methyl Ester 9 under Mitsunobu Conditions S36

1-*n*-Pentyl-1*H*-indazole (**25**) S37

2-*n*-Pentyl-2*H*-indazole (**26**) S39

3-Methyl-1-*n*-pentyl-1*H*-indazole (**27**) S41

3-Methyl-2-*n*-pentyl-2*H*-indazole (**28**) S43

3-*tert*-Butyl-1-*n*-pentyl-1*H*-indazole (**29**) S45

3-Phenyl-1-*n*-pentyl-1*H*-indazole (**31**) S47

3-Phenyl-2-*n*-pentyl-2*H*-indazole (**32**) S49

3-Iodo-1-*n*-pentyl-1*H*-indazole (**33**) S51

3-Iodo-2-*n*-pentyl-2*H*-indazole (**34**) S53

3-Bromo-1-*n*-pentyl-1*H*-indazole (**35**) S55

3-Bromo-2-*n*-pentyl-2*H*-indazole (**36**) S57

3-Chloro-1-*n*-pentyl-1*H*-indazole (**37**) ... S59
3-Chloro-2-*n*-pentyl-2*H*-indazole (**38**) ... S61
3-Nitro-1-*n*-pentyl-1*H*-indazole (**39**) ... S63
3-Nitro-2-*n*-pentyl-2*H*-indazole (**40**) ... S65
1-*n*-Pentyl-1*H*-indazole-3-carbonitrile (**41**) ... S67
1-*n*-Pentyl-1*H*-indazole-3-carboxaldehyde (**43**) ... S69
1-(1-*n*-Pentyl-1*H*-indazol-3-yl)ethan-1-one (**45**) ... S71
N-Methyl-1-*n*-pentyl-1*H*-indazole-3-carboxamide (**47**) ... S73
(1-*n*-Pentyl-1*H*-indazol-3-yl)(pyrrolidin-1-yl)methanone (**49**) ... S75
7-Methyl-1-*n*-pentyl-1*H*-indazole (**51**) ... S77
7-Methyl-2-*n*-pentyl-2*H*-indazole (**52**) ... S79
7-Bromo-1-*n*-pentyl-1*H*-indazole (**53**) ... S81
7-Bromo-2-*n*-pentyl-2*H*-indazole (**54**) ... S83
7-Nitro-1-*n*-pentyl-1*H*-indazole (**55**) ... S85
7-Nitro-2-*n*-pentyl-2*H*-indazole (**56**) ... S87
Methyl 1-*n*-pentyl-1*H*-indazole-7-carboxylate (**57**) ... S89
Methyl 2-*n*-pentyl-2*H*-indazole-7-carboxylate (**58**) ... S91
Methyl 1-*n*-pentyl-1*H*-indazole-6-carboxylate (**59**) ... S93
Methyl 2-*n*-pentyl-2*H*-indazole-6-carboxylate (**60**) ... S95
Methyl 1-*n*-pentyl-1*H*-indazole-5-carboxylate (**61**) ... S97
Methyl 2-*n*-pentyl-2*H*-indazole-5-carboxylate (**62**) ... S99
Methyl 1-*n*-pentyl-1*H*-indazole-4-carboxylate (**63**) ... S101
Methyl 2-*n*-pentyl-2*H*-indazole-4-carboxylate (**64**) ... S103
Methyl 1-benzyl-1*H*-indazole-3-carboxylate (**69**) ... S105
Methyl 2-benzyl-2*H*-indazole-3-carboxylate (**70**) ... S107
Methyl 1-(2-methylbenzyl)-1*H*-indazole-3-carboxylate (**71**) ... S109
Methyl 2-(2-methylbenzyl)-2*H*-indazole-3-carboxylate (**72**) ... S111
Methyl 1-(cyclohexylmethyl)-1*H*-indazole-3-carboxylate (**73**) ... S113
Methyl 2-(cyclohexylmethyl)-2*H*-indazole-3-carboxylate (**74**) ... S115
Methyl 1-(pentan-2-yl)-1*H*-indazole-3-carboxylate (**75**) ... S117
Methyl 2-(pentan-2-yl)-2*H*-indazole-3-carboxylate (**76**) ... S119
Methyl 1-(pentan-3-yl)-1*H*-indazole-3-carboxylate (**77**) ... S121
Methyl 2-(pentan-3-yl)-2*H*-indazole-3-carboxylate (**78**) ... S123
Synthesis of Tosylates ... S125
General Procedure (C): ... S125

n-Pentyl 4-methylbenzenesulfonate S125
Benzyl 4-methylbenzenesulfonate S127
Cyclohexylmethyl 4-methylbenzenesulfonate S129
Pentan-2-yl 4-methylbenzenesulfonate S131
Regioisomeric distribution (*N*-1:*N*-2) determination; Crude ^{1}H NMR spectra S133
Table 1, Entry 3 .... S133
Table 1, Entry 18 .... S134
Scheme 1 .... S135
Table 2, Entry 1 (Conditions A) S136
Table 2, Entry 1 (Conditions B) S137
Table 2, Entry 2 (Conditions A) S138
Table 2, Entry 2 (Conditions B) S139
Table 2, Entry 3 (Conditions A) S140
Table 2, Entry 3 (Conditions B) S141
Table 2, Entry 4 (Conditions A) S142
Table 2, Entry 4 (Conditions B) S143
Table 2, Entry 5 (Conditions A) S144
Table 2, Entry 5 (Conditions B) S145
Table 2, Entry 6 (Conditions A) S146
Table 2, Entry 6 (Conditions B) S147
Table 2, Entry 7 (Conditions A) S148
Table 2, Entry 7 (Conditions B) S149
Table 2, Entry 8 (Conditions A) S150
Table 2, Entry 8 (Conditions B) S151
Table 2, Entry 9 (Conditions A) S152
Table 2, Entry 9 (Conditions B) S153
Table 2, Entry 10 (Conditions A) S154
Table 2, Entry 10 (Conditions B) S155
Table 2, Entry 11 (Conditions A) S156
Table 2, Entry 11 (Conditions B) S157
Table 2, Entry 12 (Conditions A) S158
Table 2, Entry 12 (Conditions B) S159
Table 2, Entry 13 (Conditions A) S160
Table 2, Entry 13 (Conditions B) S161
Table 3, Entry 1 .... S162

Table 3, Entry 2.. S163
Table 3, Entry 3.. S164
Table 3, Entry 4.. S165
Table 3, Entry 5.. S166
Table 3, Entry 6.. S167
Table 3, Entry 7.. S168
Table 3, Entry 8.. S169
Table 3, Entry 9.. S170
Table 4, Entry 1 (Conditions A) S171
Table 4, Entry 1 (Conditions B) S172
Table 4, Entry 2 (Conditions A) S173
Table 4, Entry 2 (Conditions B) S174
Table 4, Entry 3 (Conditions A) S175
Table 4, Entry 3 (Conditions B) S176
Table 4, Entry 4 (Conditions A) S177
Table 4, Entry 4 (Conditions B) S178
Table 4, Entry 5 (Conditions A) S179
Table 4, Entry 5 (Conditions B) S180
Table 4, Entry 6 (Conditions A) S181
Table 4, Entry 6 (Conditions B) S182
Table 4, Entry 7 (Conditions A) S183
Table 4, Entry 7 (Conditions B) S184
Table 5, Entry 1.. S185
Table 5, Entry 2.. S186
Table 6, Entry 1 (Conditions A) S187
Table 6, Entry 1 (Conditions B) S188
Table 6, Entry 2 (Conditions A) S189
Table 6, Entry 2 (Conditions B) S190
Table 6, Entry 3 (Conditions A) S191
Table 6, Entry 3 (Conditions B) S192
Table 6, Entry 4 (Conditions A) S193
Table 6, Entry 4 (Conditions B) S194
Table 6, Entry 5 (Conditions A) S195
Table 6, Entry 5 (Conditions B) S196
Table 6, Entry 6 (Conditions A) S197

Table 6, Entry 6 (Conditions B) S198
Table 6, Entry 7 (Conditions A) S199
Table 6, Entry 7 (Conditions B) S200
Table 6, Entry 8 (Conditions A) S201
Table 6, Entry 8 (Conditions B) S202
Table 6, Entry 9 (Conditions A) S203
Table 6, Entry 9 (Conditions B) S204
References S205

Experimental Note

All reagents were obtained from commercial sources and were used without further purification, unless otherwise stated. The following solvents were distilled prior to use, according to the following methods; THF was freshly distilled from sodium benzophenone ketyl, CH_2Cl_2 was distilled from phosphorus pentoxide and stored over 4 Å molecular sieves, MeOH was distilled from Mg/I_2 onto 3 Å molecular sieves. Room temperature (rt) ranged between 16.5–24 °C with an average value of 20 °C. Thin layer chromatography (TLC) and preparative TLC (PTLC) was carried out on pre-coated Merck silica gel GF254 plates, using $UV_{254\ nm}$ light detection and/or basic aq. $KMnO_4$ staining. Molecular sieves (3 Å and 4 Å) were dried prior to use, by heating to 170 °C for 48 h. Wet flash column chromatography was performed using Merck Kieselgel 60 (particle size 0.040–0.063 mm, density 0.8 g/cm^3).

Nuclear magnetic resonance (NMR) spectroscopy was performed on a Bruker Avance 400 MHz NMR spectrometer or Bruker Avance 300 MHz NMR spectrometer at 20 °C, using $CDCl_3$ (with tetramethylsilane [TMS] as internal standard, δ_H 0.00 ppm) or DMSO-d_6 as sample solvent. Chemical shift values (δ_H and δ_C) are reported in parts per million (ppm) relative to TMS ($CDCl_3$) and/or residual solvent (δ_H 2.50 [DMSO-d_6] or δ_H 7.26 [$CDCl_3$]) and coupling constants (J) are expressed in Hertz (Hz), in the following format; chemical shift value (integration, multiplicity, coupling constant). ^{1}H NMR spectral data are described, using the following abbreviations; s (singlet), brs (broad singlet), d (doublet), t (triplet), tq (triplet of quartets), q (quartet), quint (quintet), ddd (doublet of doublets of doublets), appsext (apparent sextet), and m (multiplet). ^{13}C NMR spectral data was calibrated using residual solvent signals for $CDCl_3$ (δ_C 77.0, t) or DMSO-d_6 (δ_C 39.5, septet).

Melting point (m.p.) datum was obtained (uncorrected), using a Stuart® Analogue Melting Point Apparatus SMP11, for non-recrystallized solids (unless otherwise stated). Infrared (IR) spectra were obtained using a Perkin Elmer Spectrum Two FT-IR Spectrometer (Shelton, CT, USA). High-resolution mass spectrometry (HRMS) experiments were performed on a Waters Micromass LCT Premier® time-of-flight (TOF) mass spectrometer or a Waters Vion IMS QTOF mass spectrometer using electrospray ionization (ESI). The eluent system employed for HRMS analysis consisted of MeCN/H_2O (1:1) and contained 0.1% v/v formic acid. HRMS experiments were performed using leucine enkephalin as an internal calibrant.

Synthesis of C-3 substituted 1*H*-indazoles

1*H*-Indazole-3-carboxylic acid

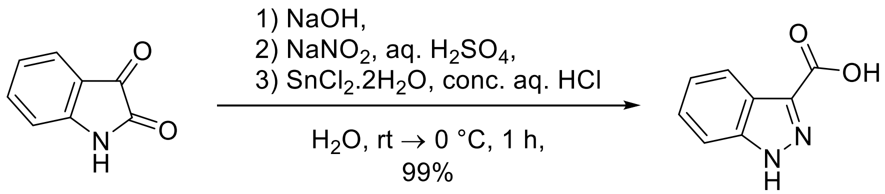

A chilled solution of isatin (12.098 g, 82.23 mmol) in aq. NaOH (3.618 g, 90.45 mmol, 25 mL H_2O) was stirred at room temperature for 10 min (complete consumption of isatin was observed by TLC [EtOAc, R_f = 0.80]). The resulting pale orange solution was cooled to 0 °C and slowly charged with aq. $NaNO_2$ (5.674 g, 82.23 mmol, 25 mL H_2O) at 0 °C. The combined aq. solution was then slowly transferred (temperature < 4 °C) via cannula to a stirred solution of aq. H_2SO_4 (8.74 mL, 164.46 mmol, in 150 mL H_2O) at 0 °C. Upon completion of addition, the resulting mixture was stirred at 0 °C for a further 15 min to afford a tan brown mixture. To control foaming, Et_2O was occasionally added dropwise to the reaction mixture. The diazonium salt solution was then slowly introduced via cannula (temperature < 4 °C) to a vigorously stirred solution of $SnCl_2.2H_2O$ (46.385 g, 205.58 mmol) in conc. HCl (75 mL, 12.1 M) at 0 °C. Upon complete addition of the diazonium salt solution, the reaction mixture was left to stir for 1 h at 0 °C. Filtration of the resulting pale-yellow suspension under vacuum furnished a bright yellow solid wet cake which was further dried under vacuum in the presence of P_2O_5 overnight to give 1*H*-indazole-3-carboxylic acid as a fine bright yellow solid (13.334 g, 99%). A portion of the title compound was further purified by recrystallization from DMF/CH_2Cl_2 for spectral characterization: m.p. 268 °C (DMF/CH_2Cl_2) (lit. m.p. 265–265.5 °C [AcOH])[1]; IR (ATR, cm^{-1}) ν_{max} 3187, 2939, 1687, 1626, 1474, 1241, 738; ^1H NMR (400 MHz, DMSO-d_6) δ 13.79 (1H, brs), 12.97 (1H, brs), 8.08 (1H, d, J = 8.2 Hz), 7.64 (1H, d, J = 8.4 Hz), 7.43 (1H, ddd, J = 7.9, 7.1, 0.8 Hz), 7.28 (1H, t, J = 7.5 Hz); ^{13}C NMR (100 MHz, DMSO-d_6) δ 163.9, 141.1, 136.0, 126.6, 122.6, 122.4, 121.3, 111.1; HRMS (ESI) m/z $[M–H]^-$ Calcd for $C_8H_6N_2O_2$ 161.0357, found 161.0349 (− 5.0 ppm). Spectral data were in agreement with literature values [2].

^{1}H NMR (400 MHz, DMSO-d_6)

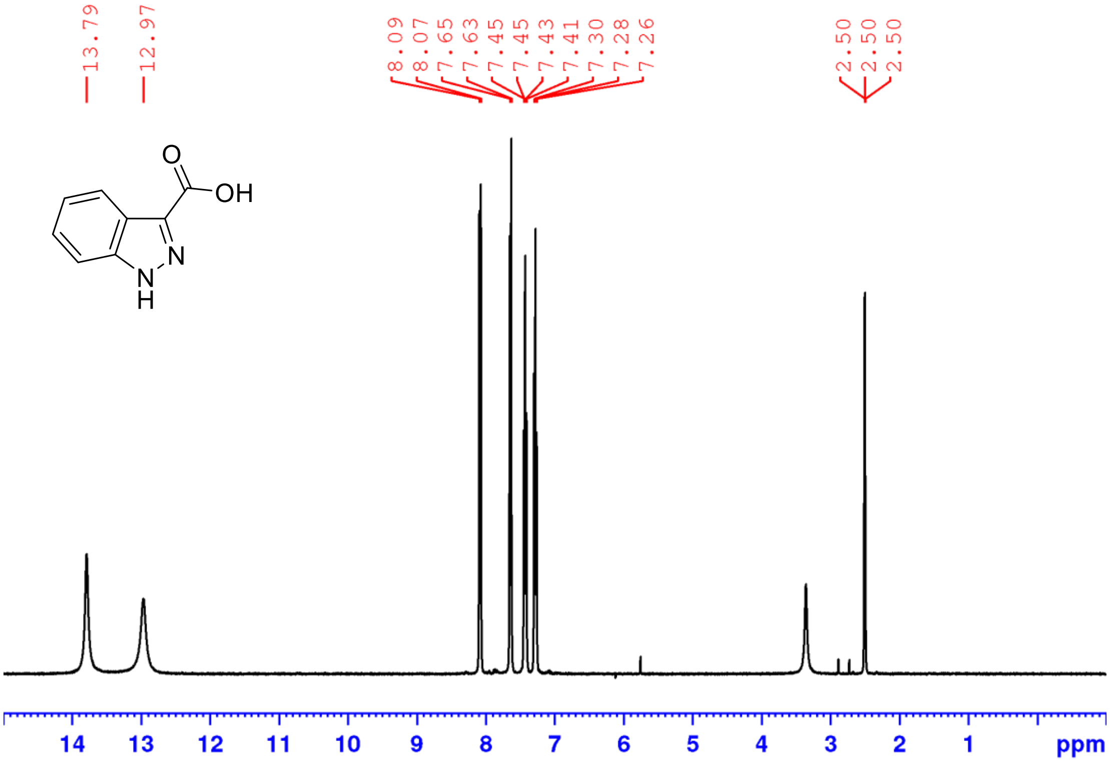

^{13}C NMR (100 MHz, DMSO-d_6)

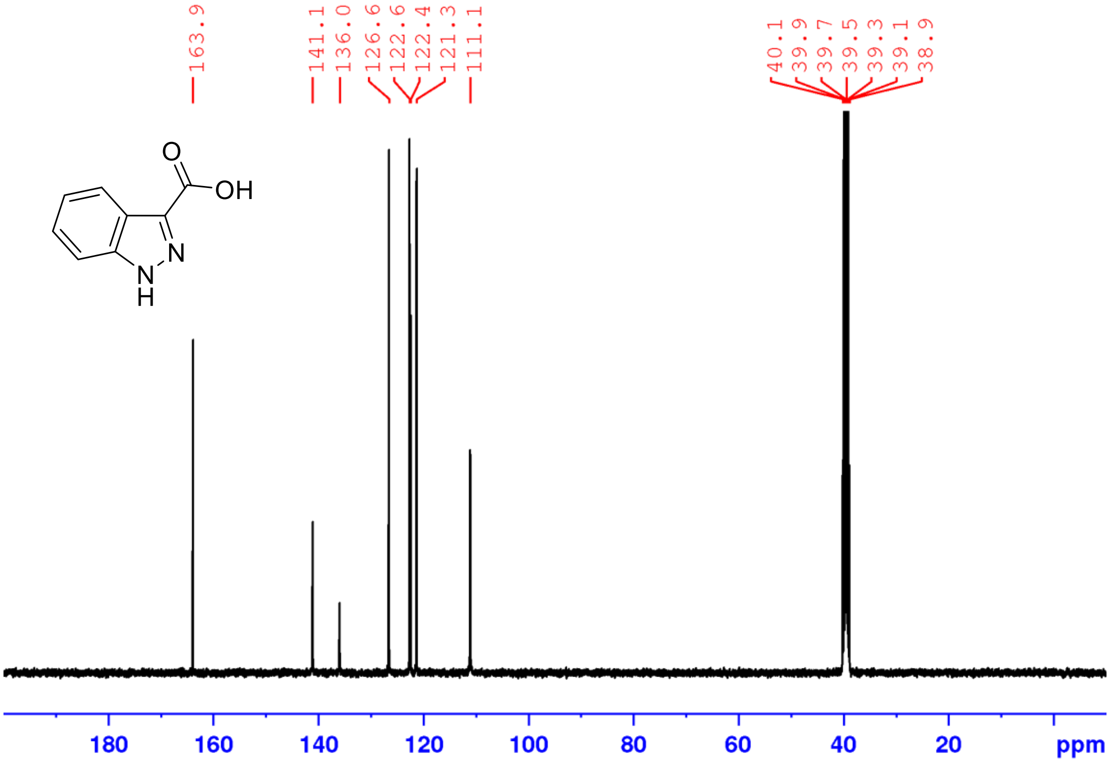

Methyl 1*H*-indazole-3-carboxylate (9)

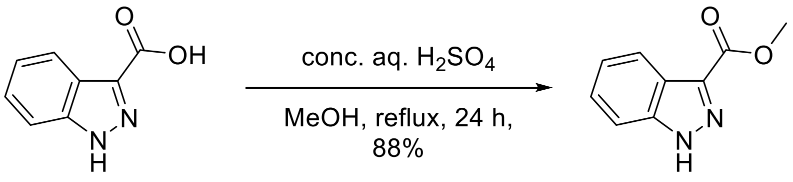

9

1*H*-Indazole-3-carboxylic acid (9 g, 55.5 mmol), in MeOH (277.5 mL, 0.2 M) was treated with conc. H_2SO_4 (1 mL, 18.4 mmol) and heated to reflux for 24 h. The resulting orange solution was concentrated in vacuo. The crude product was then reconstituted in EtOAc (150 mL) and washed with sat. aq. $NaHCO_3$ (50 mL), water (50 mL), and brine (50 mL). The resulting organic layer was then dried over $MgSO_4$ and concentrated under reduced pressure to afford title compound **9** as a bright yellow solid. Recrystallization from EtOAc/hexane (1:3) furnished the ester as a beige solid (8.585 g, 88%): m.p. 165–167.9 °C (EtOAc/hexane) (lit. m.p. 168–170 °C [EtOAc/hexane])[3]; IR (ATR, cm^{-1}) ν_{max} 3216, 3184, 3088, 3007, 2951, 1732, 1479, 1232, 1149, 746; ^1H NMR (400 MHz, $CDCl_3$) δ 13.05 (1H, brs), 8.22 (1H, d, J = 8.2 Hz), 7.77 (1H, d, J = 8.4 Hz), 7.47 (1H, t, J = 7.5 Hz), 7.34 (1H, t, J = 7.5 Hz), 4.08 (3H, s); ^{13}C NMR (100 MHz, $CDCl_3$) δ 163.5, 141.4, 136.3, 127.4, 123.3, 122.4, 121.7, 111.2, 52.1; HRMS (ESI) m/z $[M+H]^+$ Calcd for $C_9H_9N_2O_2$ 177.0659, found 177.0661 (1.1 ppm). Spectral data were in agreement with literature values [4].

^1H NMR (400 MHz, $CDCl_3$) **9**

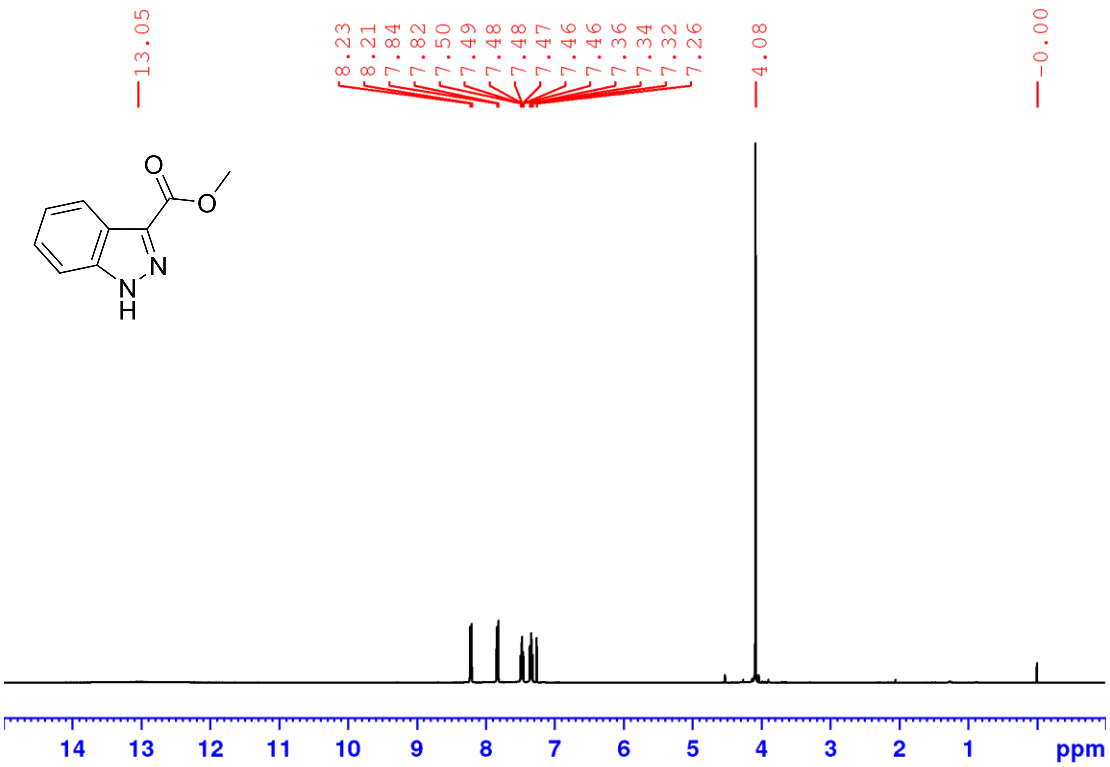

^{13}C NMR (100 MHz, $CDCl_3$) **9**

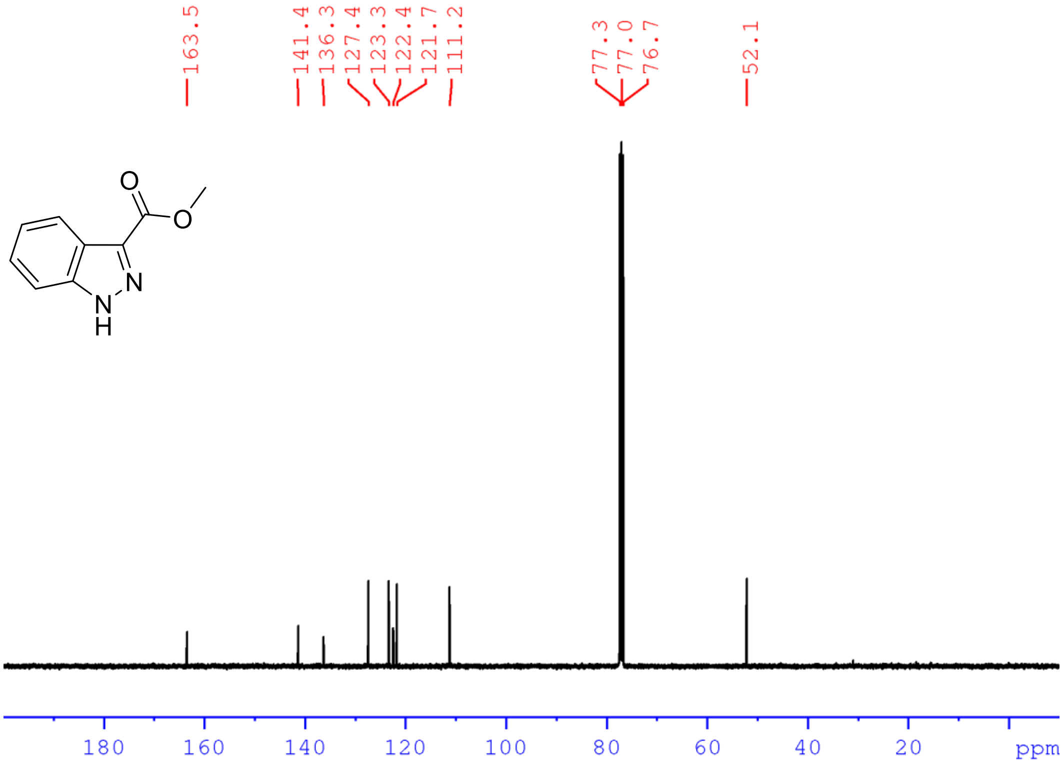

1*H*-indazole (12)

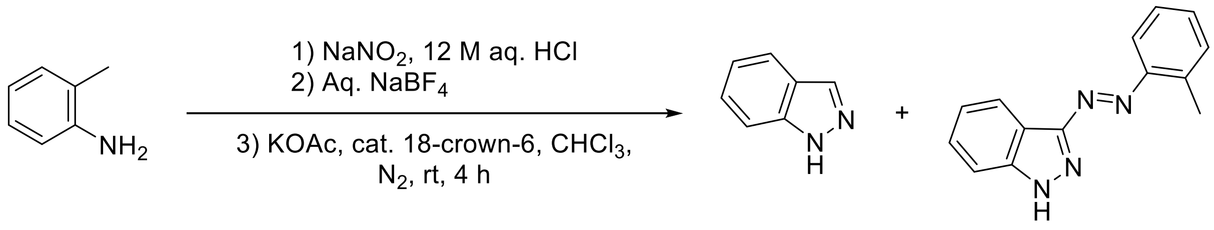

12, 30% (over two steps) 3%

To a 500 mL round bottom flask containing a cooled (−5 °C) solution of *o*-toluidine (22.01 mL, 209 mmol) in 3.2 M aq. HCl (52.25 mL conc. HCl [628 mmol] in 198.55 mL H_2O) was added $NaNO_2$ (14.421 g, 209 mmol) in H_2O (33.44 mL) dropwise (reaction mass temperature was maintained between –4 °C and 2 °C). The mixture was stirred for a further 30 min (temperature < 0 °C) and then filtered. The filtrate was then charged with aq. $NaBF_4$ (25.252 g, 230 mmol in 94.1 mL H_2O) and stirred at 0 °C for a further 40 min. The resulting solid was then isolated, using vacuum filtration, to afford a solid wet cake which was subsequently washed with cold EtOH (50 mL × 3) and cold Et_2O (50 mL × 3). The colorless solid was then added portion-wise to a stirred solution of KOAc (32.473 g, 331 mmol) and 18-crown-6 (2.036 g, 8 mmol) in $CHCl_3$ (600 mL, 0.35 M) in a 1 L vessel (fitted with a Liebig condenser) at room temperature. After 2 h, the crimson suspension was filtered under reduced pressure. The solids were further washed with $CHCl_3$ (250 mL × 3) and the resulting filtrate was dried over Na_2SO_4 prior to solvent removal under reduced pressure to give crude **12** as a brown gum (18.964 g). Recrystallization from H_2O gave **12** as a beige crystalline solid (7.4 g, 30%): m.p. 143–143.5 °C (H_2O) (lit. m.p. 143–144 °C [H_2O])[5]; IR (ATR, cm^{-1}) ν_{max} 3178, 3156, 1621, 1504, 1356, 952, 845, 739; ^1H NMR (400 MHz, $CDCl_3$) δ 10.76 (1H, brs), 8.13 (1H, s), 7.78 (1H, d , J = 8.1 Hz), 7.52 (1H, d, J = 8.4 Hz), 7.40 (1H, t, J = 7.6 Hz), 7.18 (1H, t, J = 7.5 Hz); ^{13}C NMR (100 MHz, $CDCl_3$) δ 140.0, 134.8, 126.8, 123.2, 121.0, 120.9, 109.7; HRMS (ESI) *m/z* $[M+H]^+$ Calcd for $C_7H_7N_2$ 119.0604, found 119.0606 (1.7 ppm). Spectral data were in agreement with literature values [2].

^{1}H NMR (400 MHz, $CDCl_3$) **12**

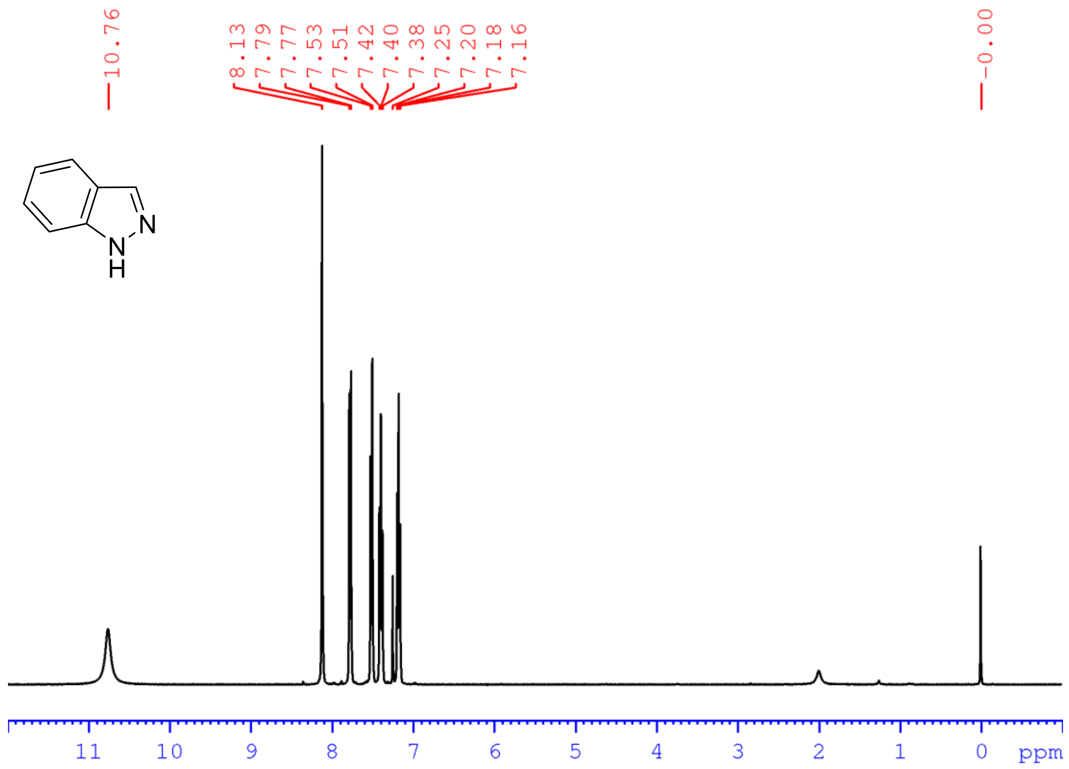

^{13}C NMR (100 MHz, $CDCl_3$) **12**

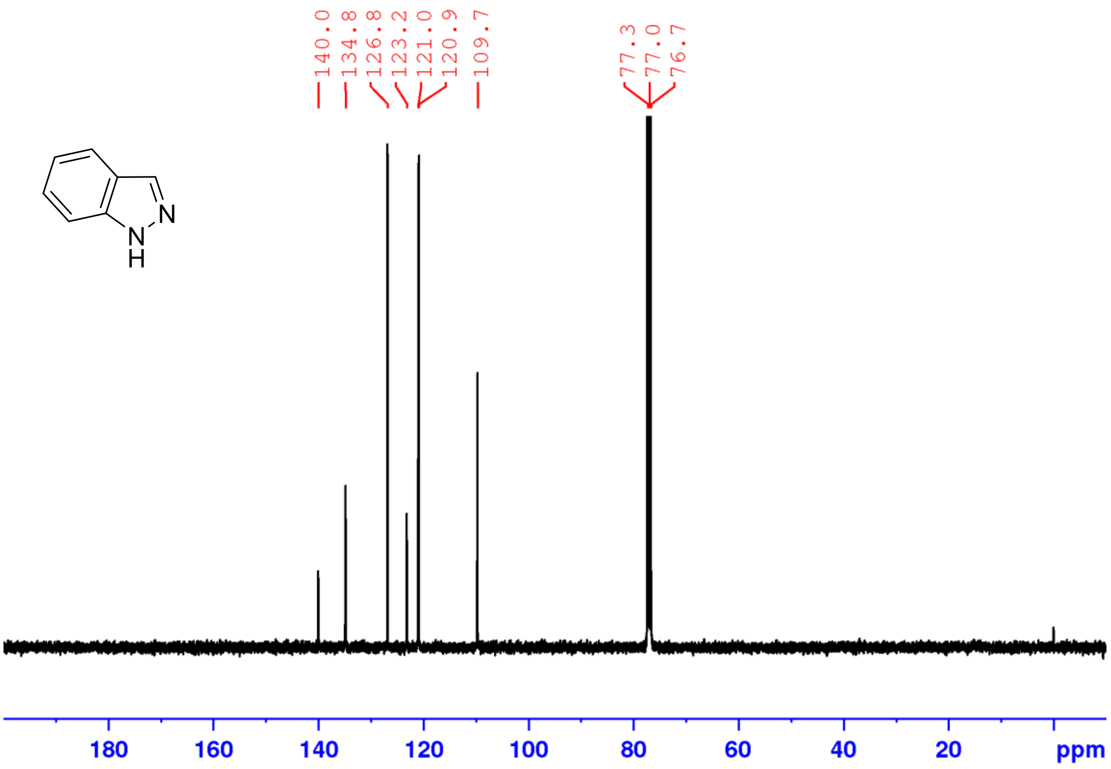

3-(*o*-Tolyldiazenyl)-1*H*-indazole

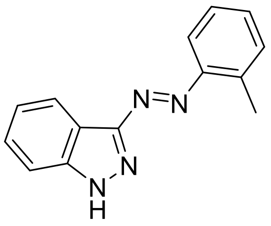

3%

Using wet flash column chromatography (EtOAc/hexane, 1:4), the title compound (R_f = 0.27) was isolated from the crude reaction mixture of indazole **12** as a brilliant yellow solid (1.481 g, 3%): m.p. 207–208.5 °C (xylene) (lit. m.p. 211–211.5 °C [xylene])[6]; IR (ATR, cm^{-1}) ν_{max} 3152, 3122, 3070, 3048, 2980, 2921, 2885, 2851, 1629, 1486 (N=N), 1374, 739, 713; ^{1}H NMR (400 MHz, DMSO-d_6) δ 13.94 (1H, brs), 8.28 (1H, d, J = 8.1 Hz), 7.70 (1H, d, J = 7.9 Hz), 7.65 (1H, d, J = 8.4 Hz), 7.50 (1H, t, J = 7.6 Hz), 7.48–7.42 (2H, m), 7.42–7.34 (2H, m), 2.75 (3H, s); ^{13}C NMR (100 MHz, DMSO-d_6) δ 155.9, 150.6, 141.4, 137.4, 131.4, 131.2, 127.5, 126.7, 124.2, 122.4, 114.3, 112.8, 110.7, 17.6; HRMS (ESI) *m/z* $[M+H]^+$ Calcd for $C_{14}H_{13}N_4$ 237.1140, found 237.1137 (– 1.3 ppm).

^{1}H NMR (400 MHz, DMSO-d_6)

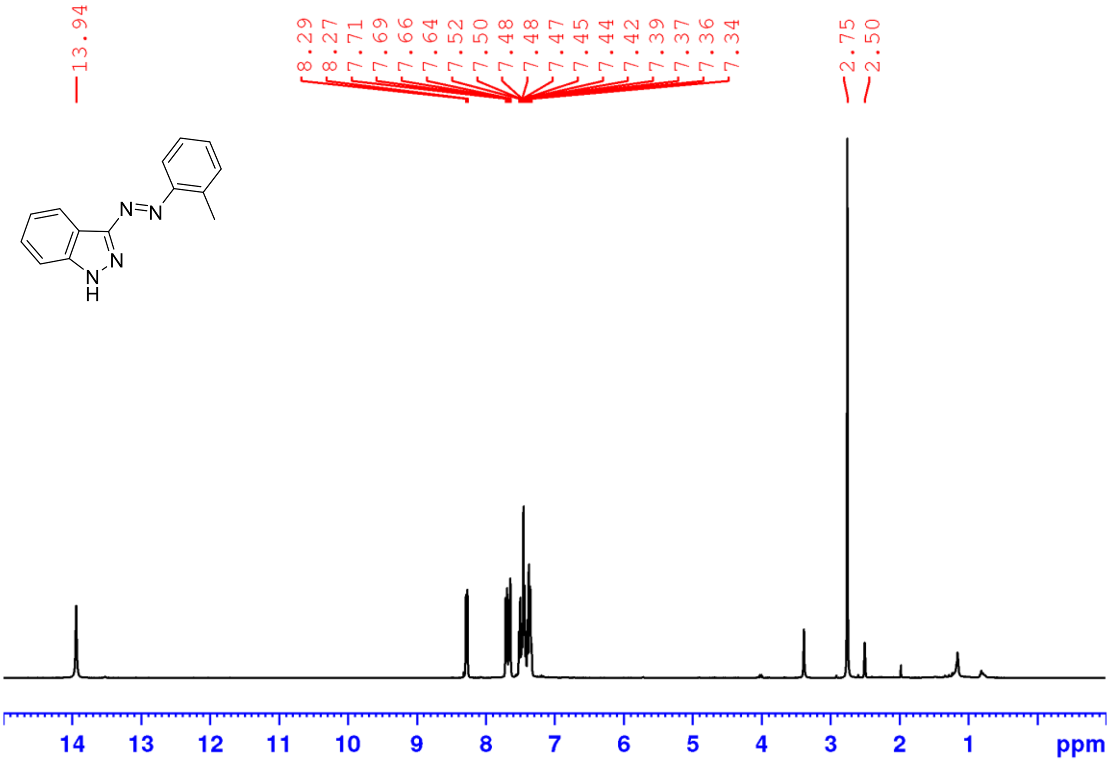

^{13}C NMR (100 MHz, DMSO-d_6)

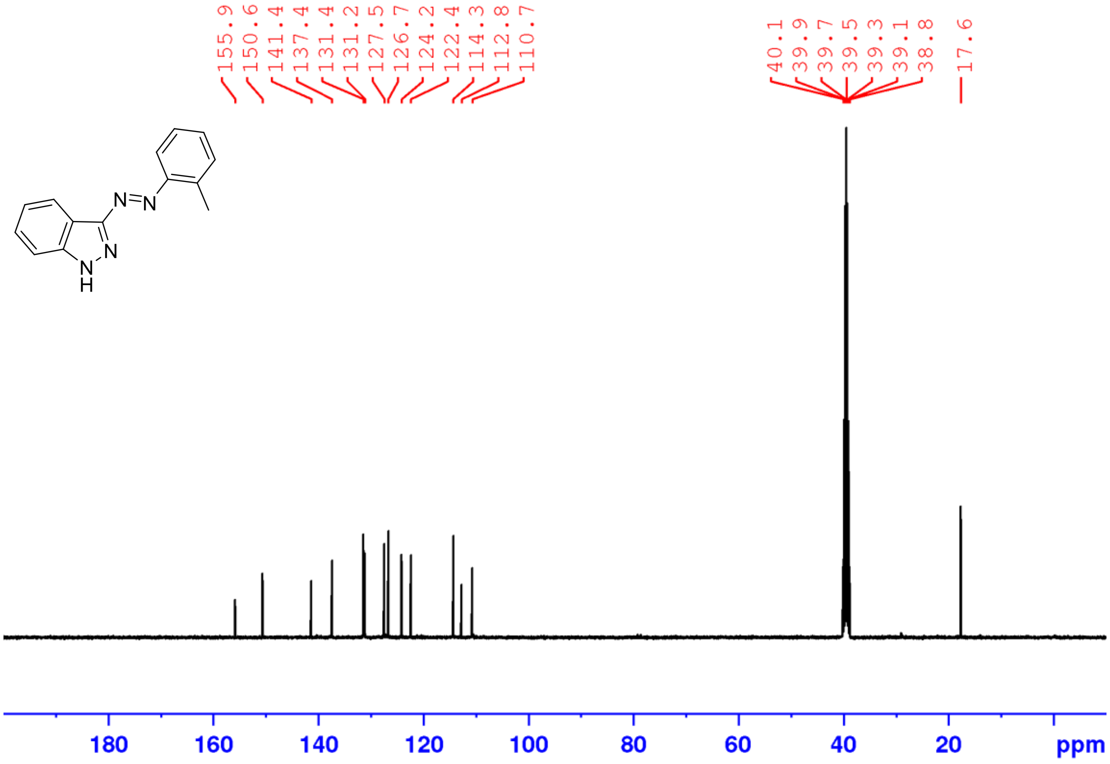

3-*tert*-Butyl-1*H*-indazole (14)

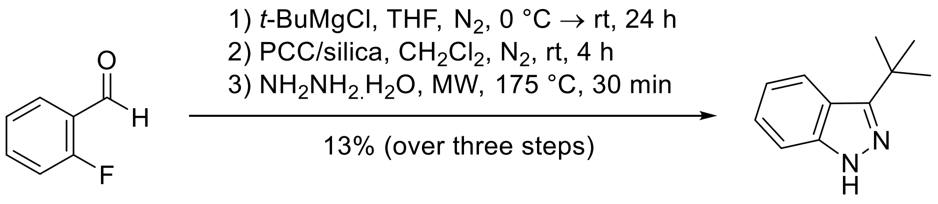

14

To an oven dried two neck 100 mL round bottom flask flush with N_2 was added 2 M *t*-BuMgCl in Et_2O (11 mL, 22 mmol) and THF (33 mL). The mixture was then treated dropwise with a solution of 2-fluorobenzaldehyde (2.11 mL, 20 mmol). The resulting solution was allowed to stir under an inert atmosphere at rt for a further 24 h. The reaction was then cooled to 0 °C and treated dropwise with sat. aq. NH_4Cl (1 mL). After stirring for 30 min, the reaction mass was concentrated in vacuo. The resulting residue was taken up in CH_2Cl_2 (50 mL) and washed with H_2O (25 mL × 2) and brine (25 mL). The organic layer was then dried over $MgSO_4$ and concentrated under reduced pressure. The resulting yellow oil was dissolved in CH_2Cl_2 (6 mL) and then slowly added to a stirring solution of PCC (3.880 g, 18 mmol) and silica (3.880 g) in CH_2Cl_2 (24 mL) and allowed to stir under an inert atmosphere for 4 h. The resulting black suspension was then filtered through a short pad of silica and eluted with CH_2Cl_2. The resulting solution was concentrated under vacuum to give an amber oil which was then transferred to a microwave vessel and containing a stir bar and hydrazine hydrate (5 mL [80% in H_2O, ≈51% hydrazine]) and allowed to stir at 175 °C for 30 min in a Discover SP® microwave reactor (CEM). The resulting mixture was then poured onto ice (15 mL) and allowed to stir for a further 30 min. The aqueous layer was then extracted with CH_2Cl_2 (10 mL × 3). The organic phases were combined, dried over $MgSO_4$, and concentrated in vacuo to give crude **14** a beige solid. Iterative recrystallization from EtOAc/hexane (1:10) subsequently furnished title compound **14** as a colorless solid (467 mg, 13%): m.p. 190–191 °C (EtOAc/hexane) (lit. m.p. 152–154 °C)[7]; IR (ATR, cm^{-1}) ν_{max} 3144, 3110, 2963, 2927, 2898, 1341, 1052, 774, 737, 429; ^{1}H NMR (300 MHz, $CDCl_3$) δ 9.10 (1H, brs), 7.90 (1H, ddd, *J* = 8.2, 1.9, 0.9 Hz), 7.43 (1H, ddd, *J* = 8.4, 1.9, 0.9 Hz), 7.34 (1H, ddd, *J* = 7.8, 6.8, 1.0 Hz), 7.11 (1H, ddd, *J* = 8.2, 6.9, 1.1 Hz), 1.54 (9H, s); ^{13}C NMR (75 MHz, $CDCl_3$) δ 154.6, 141.9, 126.2, 122.1, 120.5, 119.8, 110.0, 33.8, 30.0; HRMS (ESI) *m/z* $[M+H]^+$ Calcd for $C_{11}H_{15}N_2$ 175.1230, found 175.1228 (– 1.1 ppm). Spectral data were in agreement with literature values [7].

^{1}H NMR (300 MHz, $CDCl_3$) **14**

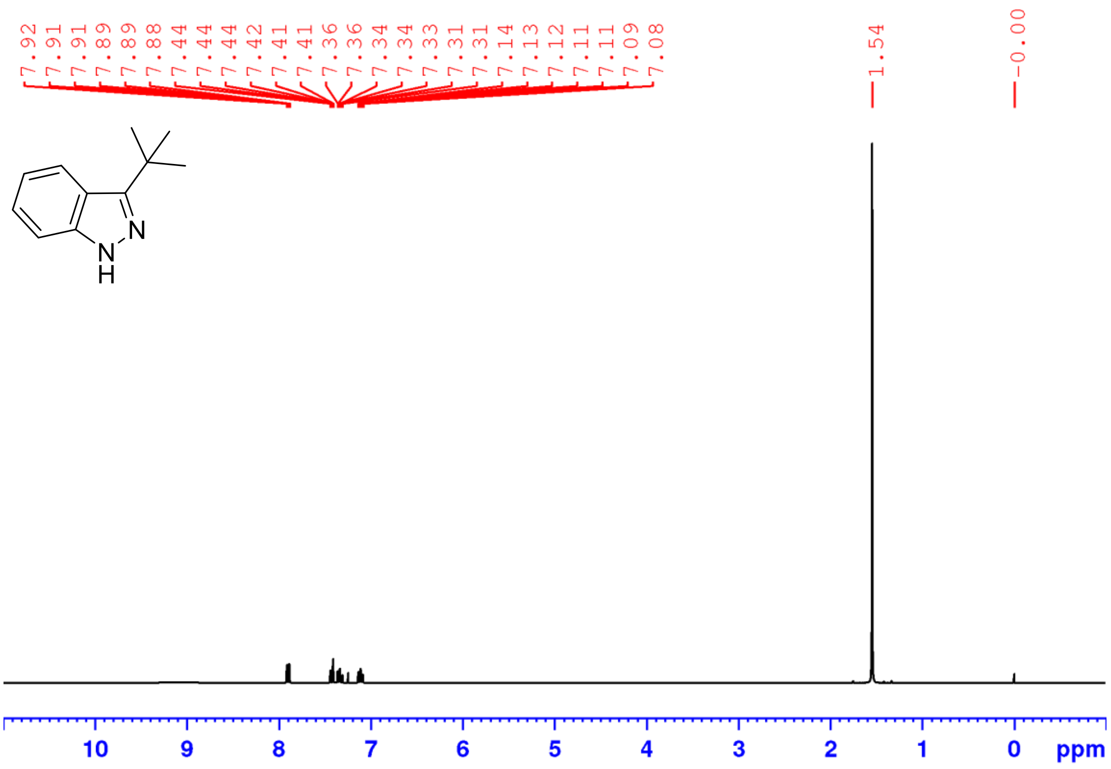

^{13}C NMR (75 MHz, $CDCl_3$) **14**

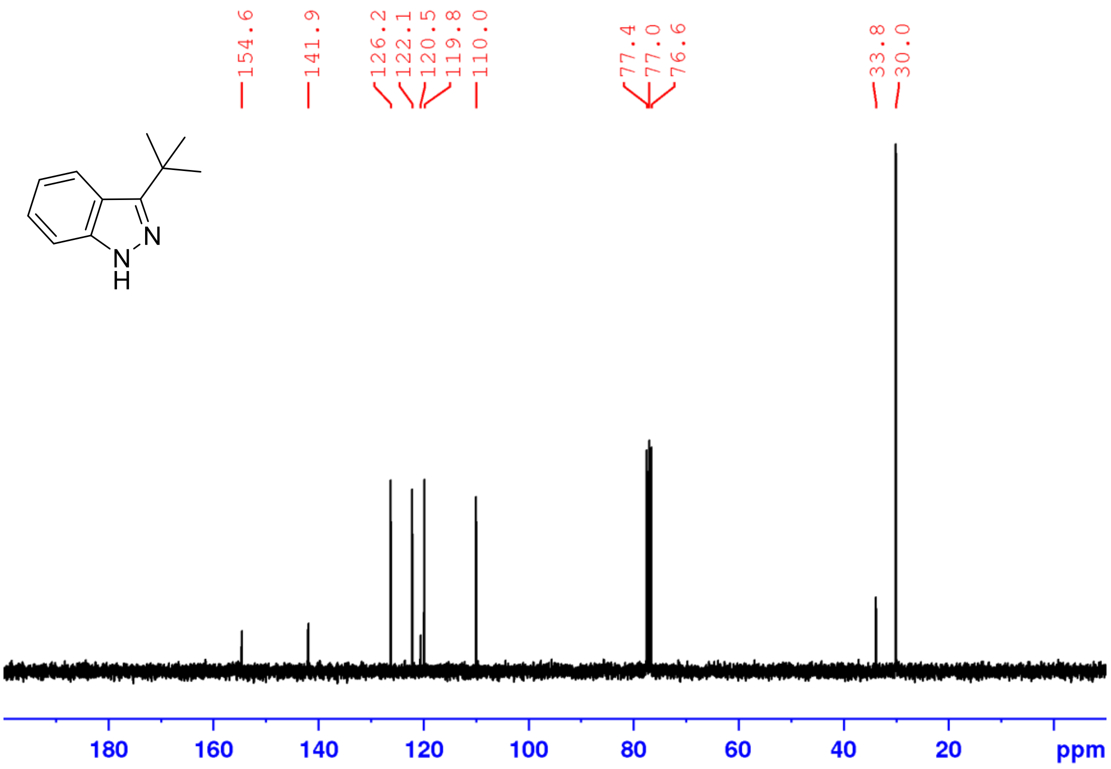

3-Phenyl-1*H*-indazole (15)

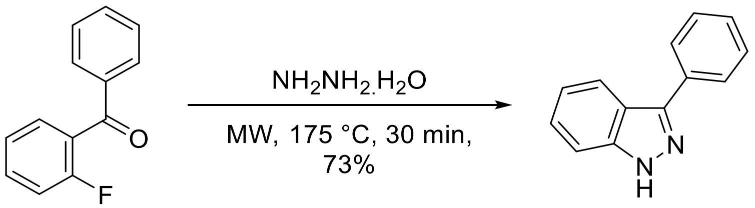

15

To a 20 mL microwave reaction vial was added 2-fluorobenzaldehyde (0.42 mL, 4 mmol) and 80% aq. hydrazine hydrate (1.6 mL, 25 mmol [50–60% hydrazine], 0.3 M). The vessel was then placed in a Discover SP® microwave reactor (CEM) and heated to 175 °C for 30 min. Upon cooling to room temperature, the reaction mass was then diluted with water (30 mL) and extracted with CH_2Cl_2 (10 mL × 3). The combined organic phases were then washed with brine (10 mL), dried over $MgSO_4$, and concentrated in vacuo to give a colorless oil which was allowed to stand at room temperature overnight. The resulting orange solid was then further purified using wet flash column chromatography (EtOAc/hexane, 1:9) to furnish title compound **15** (R_f = 0.14) as a colorless oil (567 mg, 73%) which solidified on standing at room temperature overnight to give a beige solid: m.p. 110 °C (lit. m.p. 108–111 °C)[8]; IR (ATR, cm^{-1}) ν_{max} 3150, 2933, 1344, 774, 736, 694, 427; ^1H NMR (400 MHz, $CDCl_3$) δ 11.73 (1H, brs), 8.04–7.98 (3H, m), 7.55–7.51 (2H, m), 7.47–7.43 (1H, m), 7.27 (1H, ddd, J = 8.1, 6.9, 0.8 Hz), 7.17 (1H, ddd, J = 8.0, 6.9, 0.8 Hz), 7.04 (1H, d, J = 8.5 Hz); ^{13}C NMR (75 MHz, $CDCl_3$) δ 145.7, 141.7, 133.6, 129.0, 128.2, 127.8, 126.8, 121.4, 121.1, 121.0, 110.3; HRMS (ESI) *m/z*: $[M+H]^+$ Calcd for $C_{13}H_{11}N_2$ 195.0917, found 195.0918 (0.5 ppm). Spectral data were in agreement with literature values [8].

^{1}H NMR (400 MHz, $CDCl_3$) **15**

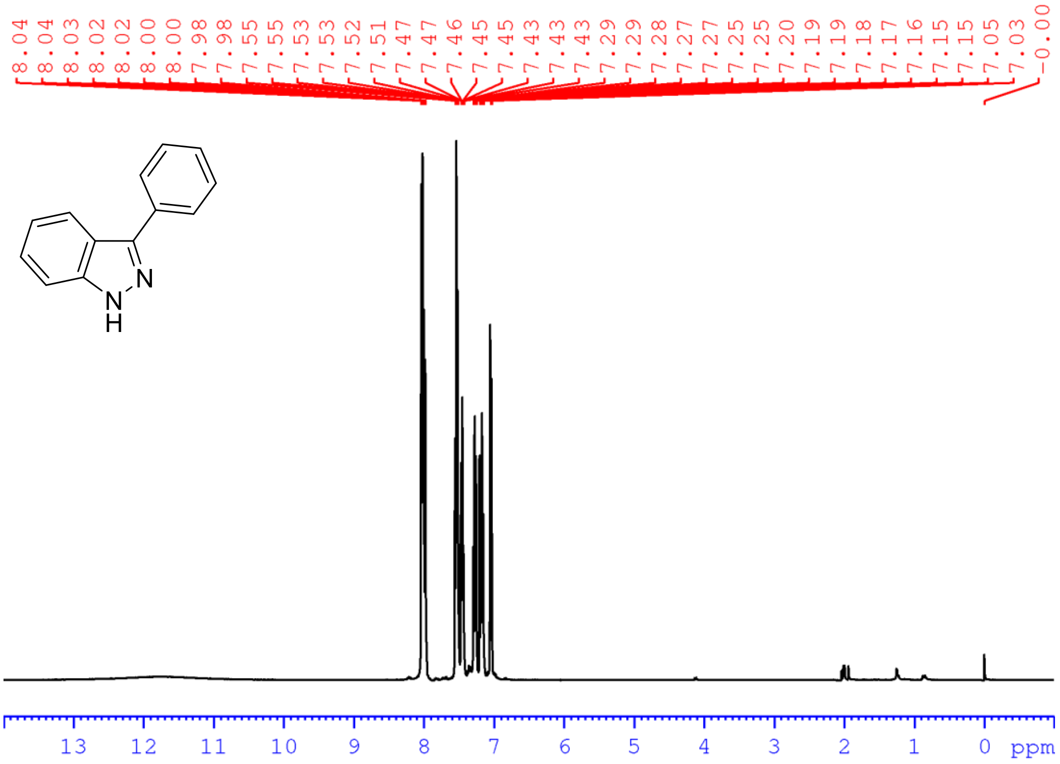

^{13}C NMR (100 MHz, $CDCl_3$) **15**

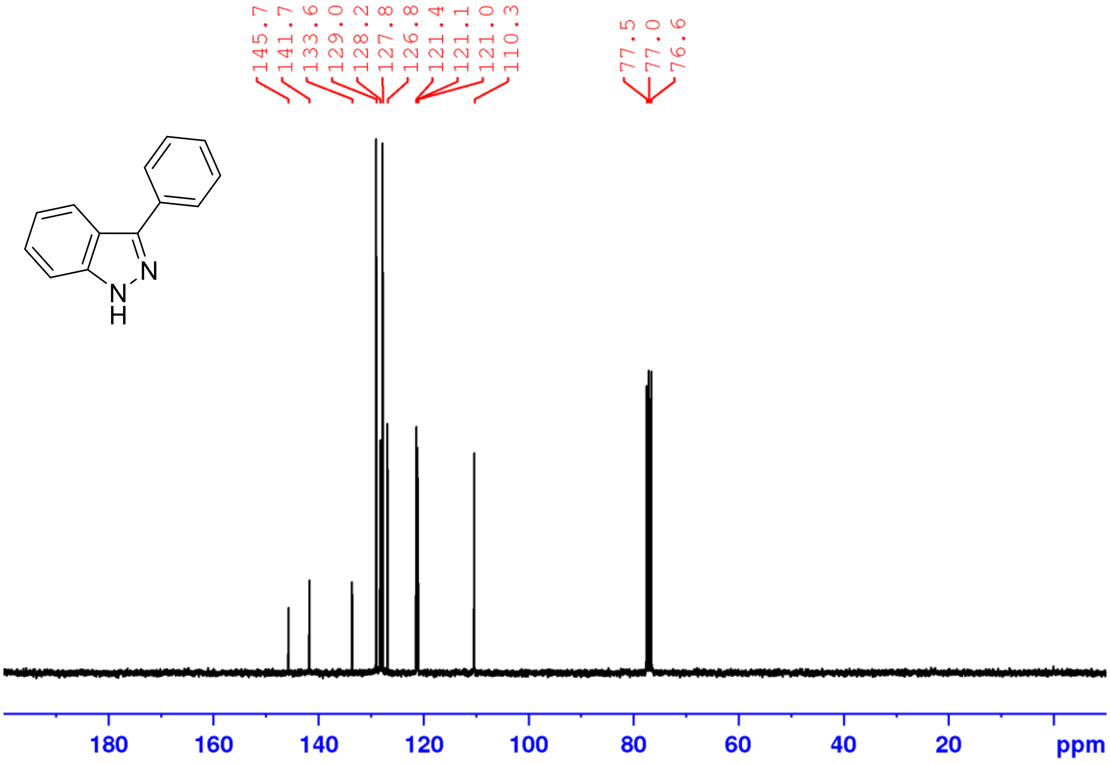

3-Iodo-1*H*-indazole (16)

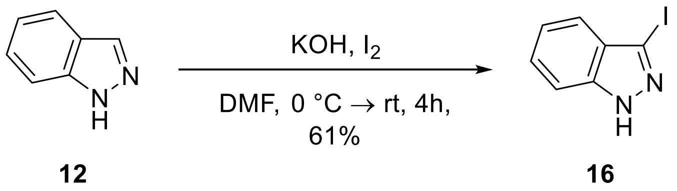

To a 500 mL round bottom flask containing 1*H*-indazole (**12**, 7.4 g, 63 mmol) and I_2 (15.989 g, 126 mmol) in DMF (190 mL), at 0 °C, was added portion wise KOH (7.071 g, 126 mmoL). The mixture was allowed to warm to room temperature and stirred for a further 4 h. The reaction mass was then treated with sat. aq. $Na_2S_2O_3$ (500 mL), diluted with H_2O (250 mL), and extracted with EtOAc (100 mL × 5). The combined organic layers were then washed with brine (250 mL × 3), dried over anhydrous Na_2SO_4, and concentrated in vacuo to give a crude brown solid residue which was further purified using wet flash column chromatography (EtOAc/hexane, 1:4) to afford the title compound **16** (R_f = 0.46) as a colorless amorphous solid (9.309 g, 61%): m.p. 142 °C (lit. m.p. 142–143 °C)[9]; IR (ATR, cm^{-1}) ν_{max} 3156, 2936, 1621, 1473, 1345, 1239, 1014, 900, 770, 738, 634; ^{1}H NMR (400 MHz, $CDCl_3$) δ 12.42 (1H, brs), 7.72 (1H, d, *J* = 8.6 Hz), 7.52 (1H, d, *J* = 8.2 Hz), 7.47 (1H, ddd, *J* = 8.0, 7.2, 0.8 Hz), 7.23 (1H, t, *J* = 7.4 Hz); ^{13}C NMR (100 MHz, $CDCl_3$) δ 140.6, 128.1, 127.4, 121.8, 121.3, 110.6, 93.5; HRMS (ESI) *m/z* $[M+H]^+$ Calcd for $C_7H_6IN_2$ 244.9570, found 244.9574 (1.6 ppm). Spectral data were in agreement with literature values [9].

^{1}H NMR (400 MHz, $CDCl_3$) **16**

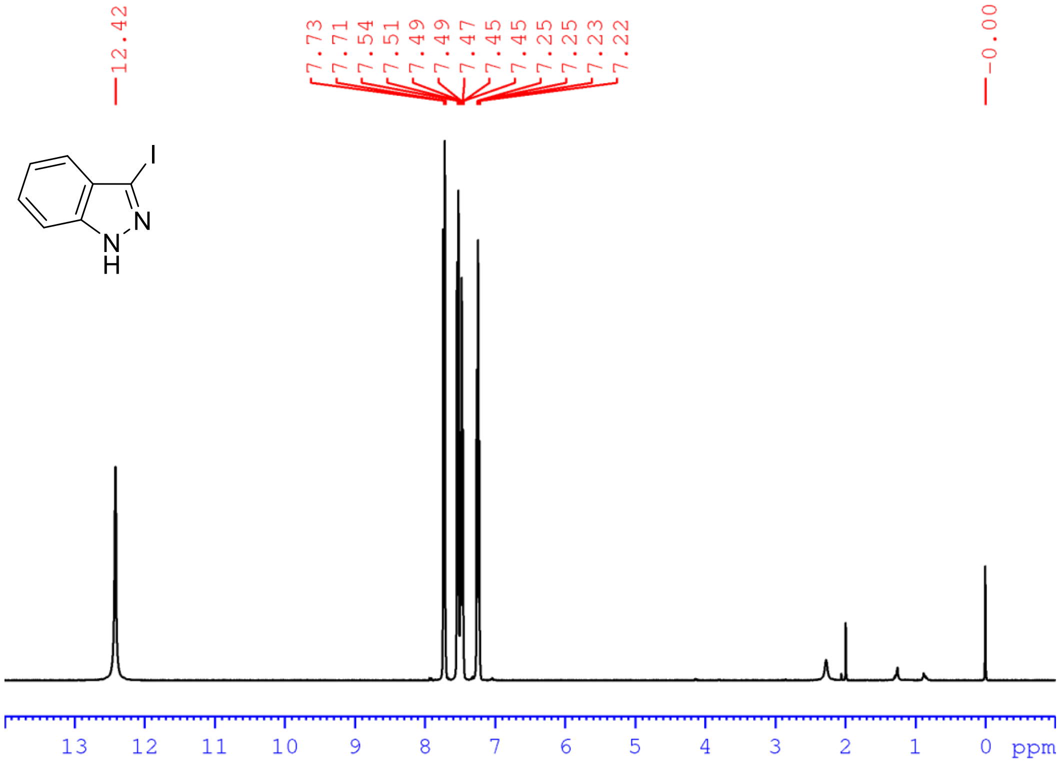

^{13}C NMR (100 MHz, $CDCl_3$) **16**

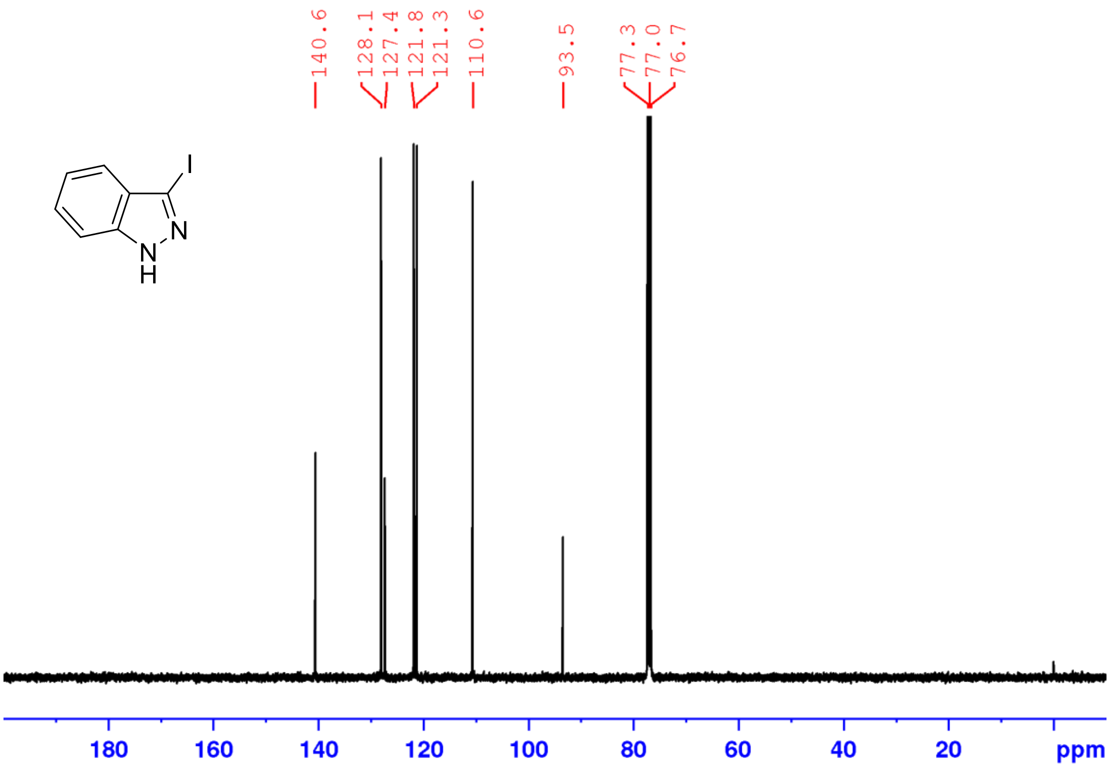

3-Bromo-1*H*-indazole (17)

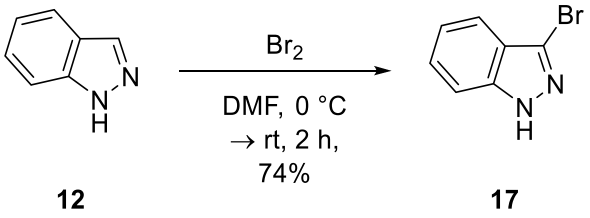

To a 50 mL round bottom flask was added 1*H*-indazole (**12**, 591 mg, 5 mmol) and DMF (10 mL). The resulting solution was then cooled to 0 °C and subsequently treated dropwise with bromine (0.38 mL, 7.41 mmol). The reaction mass was then warmed to room temperature and allowed to stir for a further 2 h. The resulting deep brown solution was then diluted with EtOAc (30 mL) and washed with brine (60 mL), sat. aq. $Na_2S_2O_3$ (60 mL), and brine (60 mL × 2). The resulting organic layer was dried over $MgSO_4$ and concentrated in vacuo to afford crude **17** as a beige solid. Wet flash column chromatography (EtOAc/hexane, 1:3) furnished title compound **17** (R_f = 0.43) as colorless solid (729 mg, 74%): m.p. 119 °C (lit. m.p. 120–122 °C)[10]; IR (ATR, cm^{-1}) ν_{max} 3156, 3129, 2945, 1625, 1480, 1331, 1243, 1026, 902, 771, 734, 640; ^1H NMR (400 MHz, $CDCl_3$) δ 11.19 (1H, brs), 7.67 (1H, d, J = 8.2 Hz), 7.59 (1H, d, J = 8.5 Hz), 7.47 (1H, ddd, J = 8.2, 7.1, 1.0 Hz), 7.25 (1H, ddd, J = 7.7, 7.0, 0.7 Hz); ^{13}C NMR (100 MHz, $CDCl_3$) δ 141.2, 128.2, 123.1, 122.9, 121.9, 120.2, 110.4; HRMS (ESI) m/z $[M+H]^+$ Calcd for $C_7H_6{}^{79}BrN_2$ 196.9709, found 196.9712 (0.9 ppm). Spectral data were in agreement with literature values [10].

^{1}H NMR (400 MHz, $CDCl_3$) **17**

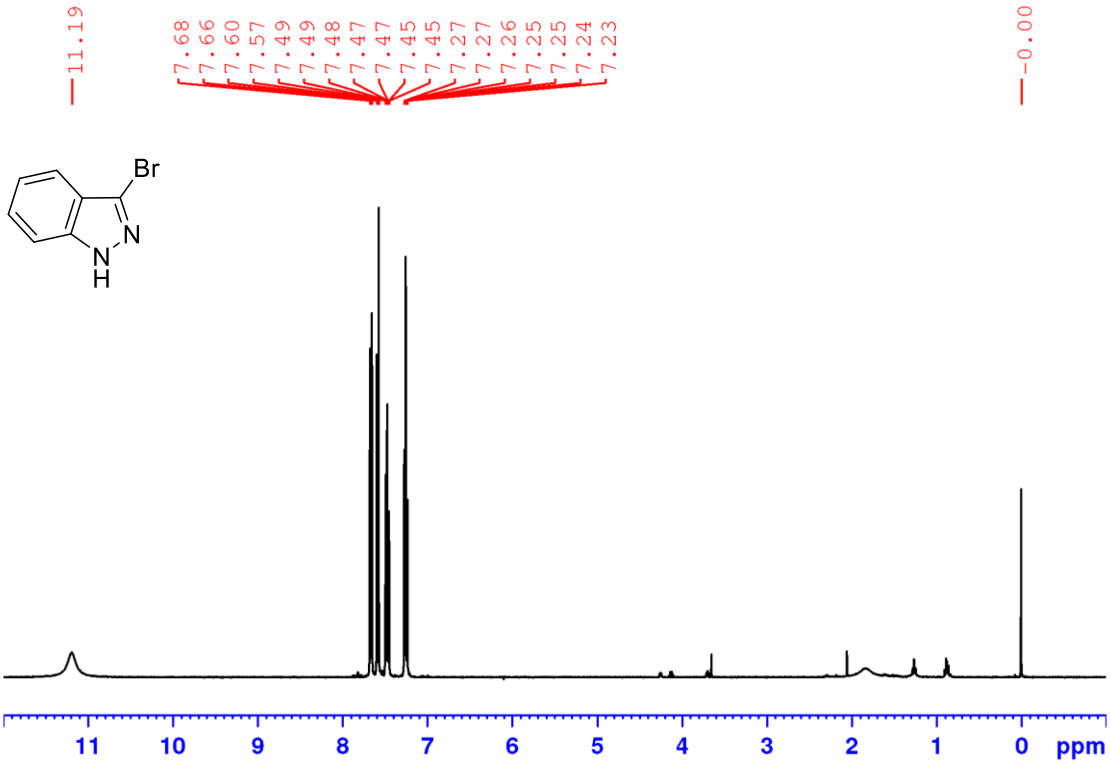

^{13}C NMR (100 MHz, $CDCl_3$) **17**

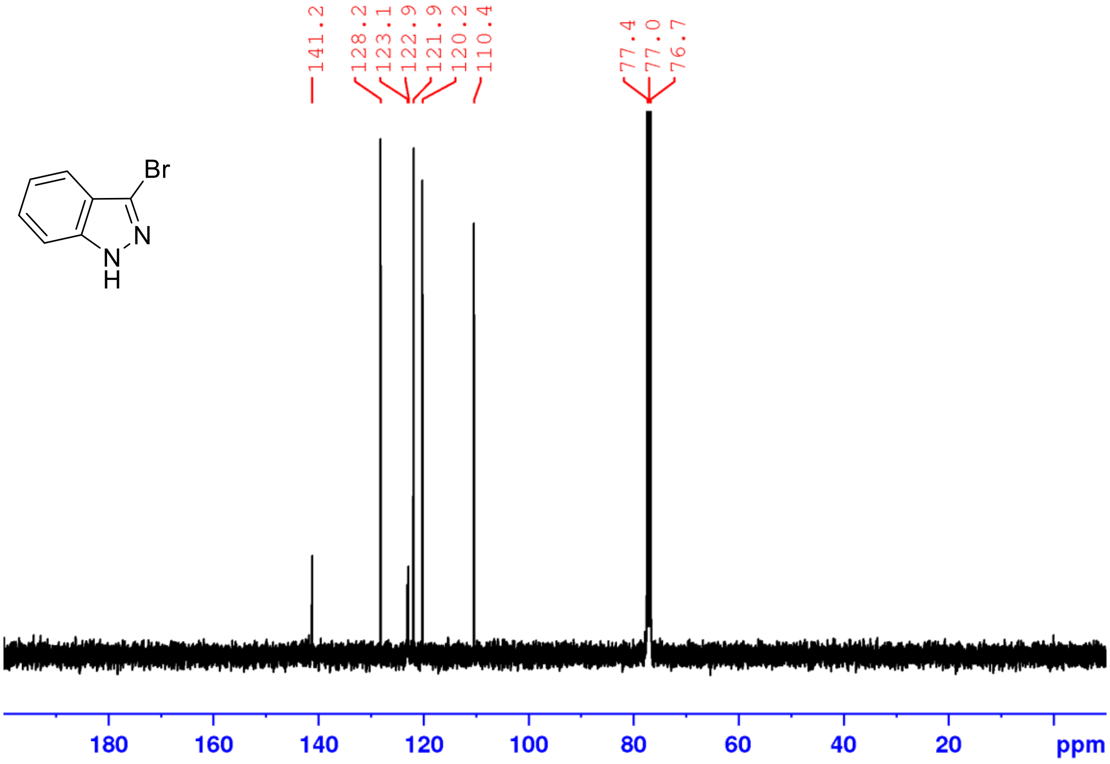

3-Nitro-1*H*-indazole (19)

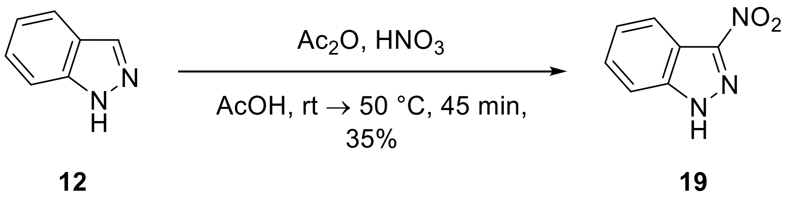

To a 50 mL round bottom flask was added 1*H*-indazole (**12**, 2.362 g, 20 mmol) and AcOH (16.6 mL). The mixture was allowed to stir for a further 15 min at room temperature, prior to the addition of conc. HNO_3 (1.02 mL, 22.8 mmol). The resulting beige suspension was then heated to 40 °C for 15 min. The reaction mixture was then treated with Ac_2O (2.74 mL, 24.84 mmol) and heated to 50 °C for 30 min. Upon cooling to room temperature, the reaction mass was treated with ice cold H_2O (60 mL) and allowed to stir for a further 1 h. The suspension was then filtered under vacuum to afford the crude product as a solid wet cake which was further purified using wet flash column chromatography (EtOAc/$CHCl_3$, 1:3) to afford title compound **19** (R_f = 0.58) as beige solid (1.139 g, 35%): m.p. 204–205 °C (lit. m.p. 205 °C)[4]; IR (ATR, cm^{-1}) ν_{max} 3196, 3170, 3058. 2945, 2907, 1531, 1381, 1322, 1249, 831, 777, 744, 427; ^{1}H NMR (300 MHz, DMSO-d_6) δ 14.45 (1H, brs), 8.11 (1H, d, J = 8.2 Hz), 7.75 (1H, d, J = 8.4 Hz), 7.57 (1H, ddd, J = 8.1, 6.9, 1.1 Hz), 7.48 (1H, ddd, J = 7.9, 7.0, 0.8, Hz); ^{13}C NMR (75 MHz, DMSO-d_6) δ 148.2, 141.4, 128.1, 125.4, 120.0, 115.2, 112.0; HRMS (ESI) m/z $[M–H]^-$ Calcd for $C_7H_4N_3O_2$ 162.0309, found 162.0301 (− 4.9 ppm). Spectral data were in agreement with literature values [4].

^{1}H NMR (300 MHz, DMSO-d_6) **19**

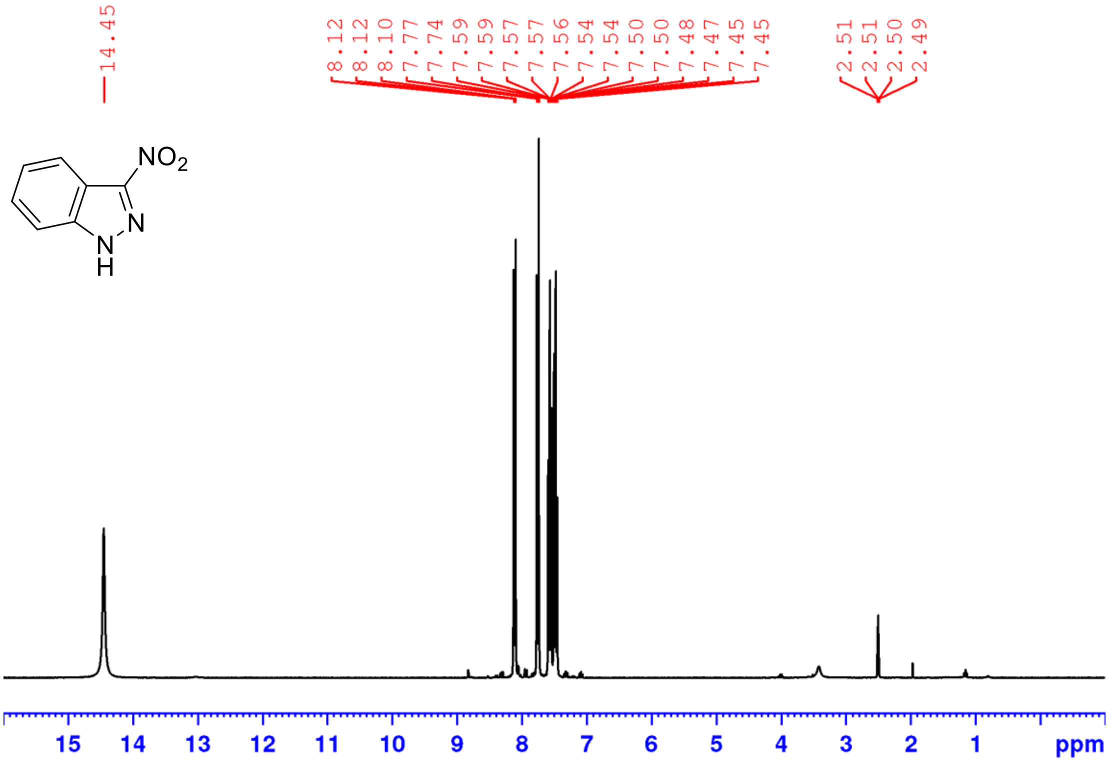

^{13}C NMR (75 MHz, DMSO-d_6) **19**

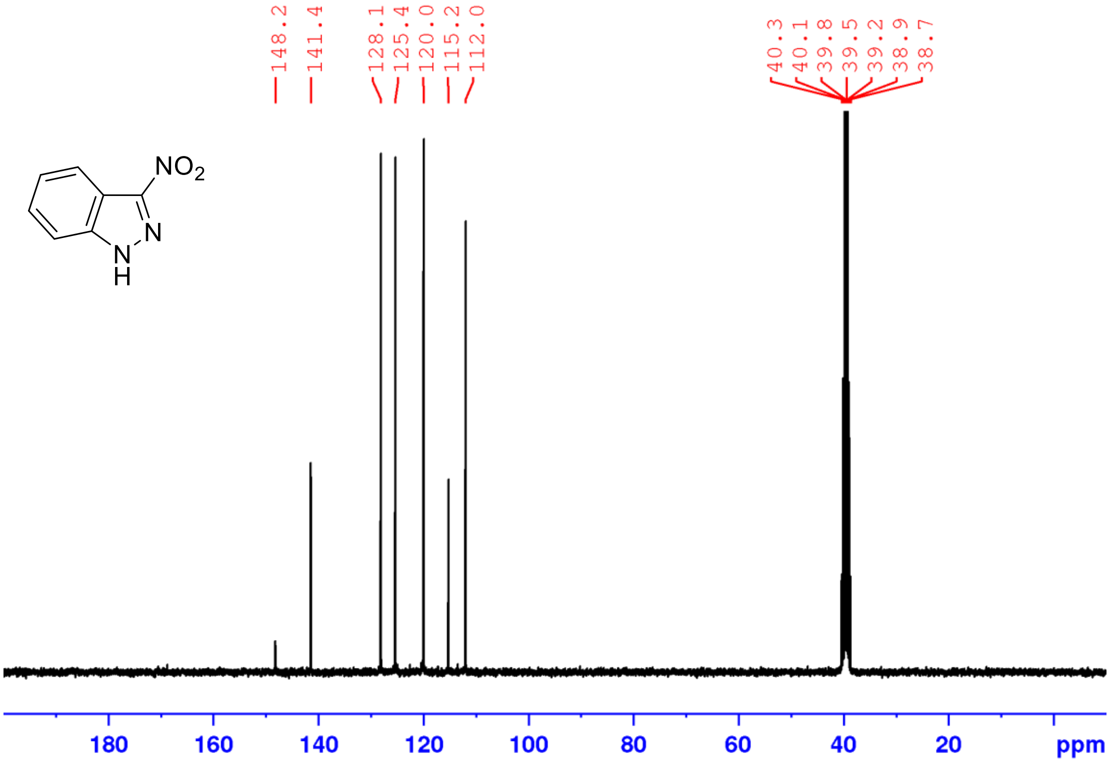

1*H*-Indazole-3-carboxaldehyde (21)

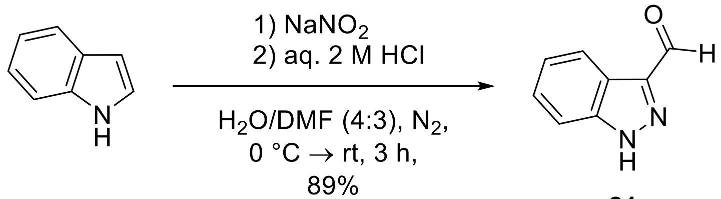

21

Aqueous HCl (4 mL, 2 M, 8.1 mmol) was slowly added (0.3 mL/min) to a cold (0 °C) solution of $NaNO_2$ (1.656 g, 24 mmol) in H_2O/DMF (12 mL H_2O, 9 mL DMF; 4:3) under a nitrogen atmosphere and left to stir for a further 10 min at 0 °C. 1*H*-Indole (351 mg, 3 mmol) in DMF (9 mL) was then slowly added (75 µL/min) to the flask over 2 h at 0 °C. The resulting mixture was stirred at room temperature for 3 h and then treated with toluene and the solvent removed under reduced pressure to afford a crude dark orange solid (2.279 g). The solid was then dissolved with CH_2Cl_2 (35 mL) and repeatedly washed with H_2O (50 mL × 3). The resulting organic layer was dried over $MgSO_4$ and concentrated in vacuo to give the title compound **21** as a brown solid (388 mg, 89%): m.p. 138 °C (lit. m.p. 141 °C)[11]; IR (ATR, cm^{-1}) ν_{max} 3184, 3161, 2953, 1668, 1087, 794, 738; ^1H NMR (400 MHz, $CDCl_3$) δ 11.51 (1H, brs), 10.34 (1H, s), 8.32 (1H, d = 8.1 Hz), 7.62 (1H, d, *J* = 8.4 Hz), 7.50 (1H, t, *J* = 7.6 Hz), 7.37 (1H, t, *J* = 7.5 Hz); ^{13}C NMR (100 MHz, $CDCl_3$) δ 187.5, 144.8, 141.2, 128.1, 124.3, 122.0, 121.0, 110.1; HRMS (ESI) *m/z* $[M+H]^+$ Calcd for $C_8H_7N_2O$ 147.0553, found 147.0552 (– 0.5 ppm). Spectral data were in agreement with literature values [11].

^{1}H NMR (400 MHz, $CDCl_3$) **21**

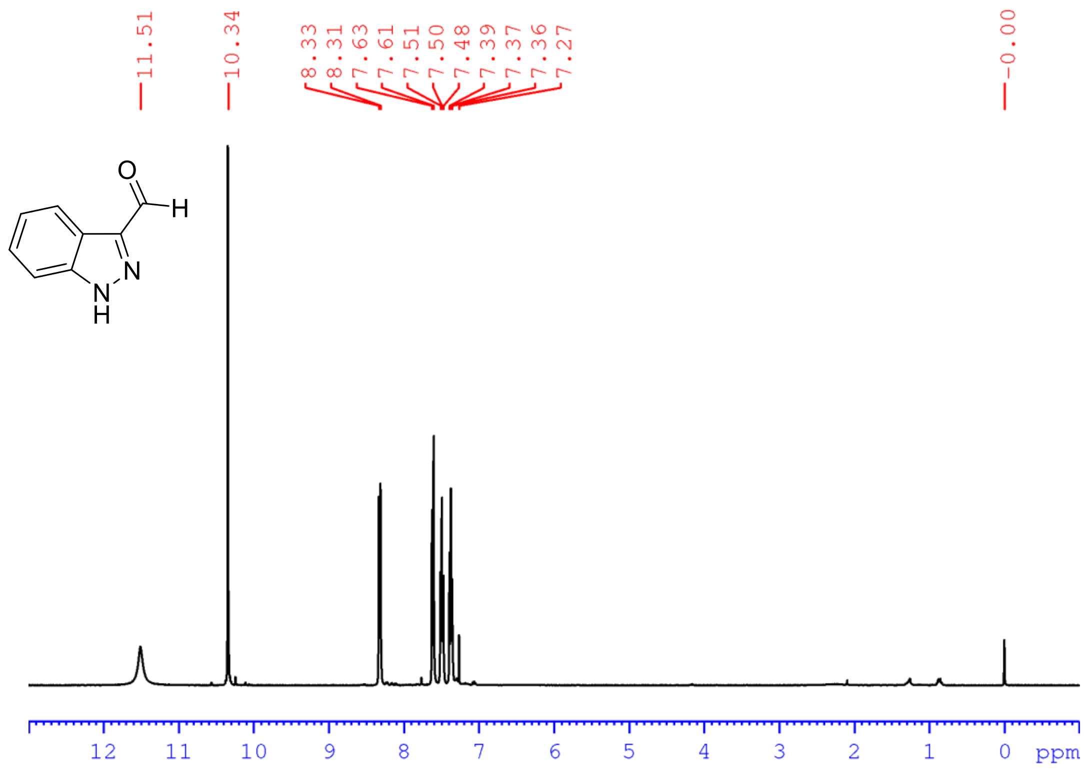

^{13}C NMR (100 MHz, $CDCl_3$) **21**

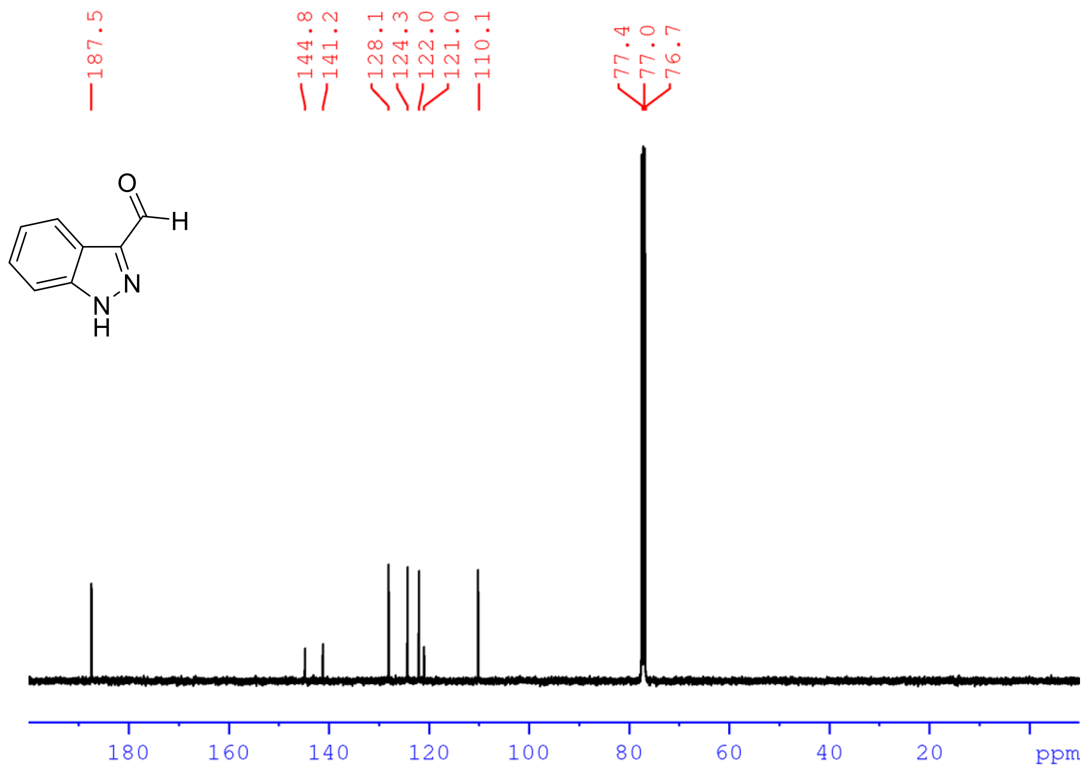

_N_-Methyl-1_H_-indazole-3-carboxamide (23)

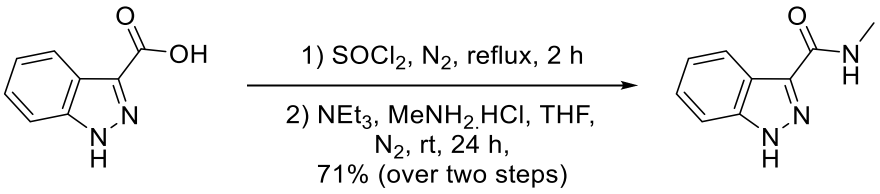

23

To an oven-dried 50 mL round bottom flask flushed with N_2 was added 1*H*-indazole-3-carboxylic acid (500 mg, 3.08 mmol) and $SOCl_2$ (5 mL, 68.9 mmol). The mixture was then heated to reflux and stirred for 2 h. The resulting mixture was then allowed to cool to room temperature and concentrated in vacuo to give an oily residue which was dissolved in THF (15.4 mL, 0.2 M) and subsequently treated dropwise with NEt_3 (0.86 mL, 6.17 mmol) and methylamine HCl (1.04 g, 15.4 mmol). The resulting dark brown suspension was allowed to stir under a nitrogen atmosphere at room temperature for a further 24 h. The yellow reaction mixture was then concentrated under reduced pressure to afford an oily residue which was taken up in $CHCl_3$ (30 mL) and washed with aq. 0.2 M aq. HCl (10 mL). The aqueous layer was subsequently extracted with $CHCl_3$ (5 mL × 2). The combined organic phases were then washed with brine (10 mL), dried over $MgSO_4$, and concentrated in vacuo to give a pale orange solid which was triturated with CH_2Cl_2 (5 mL) and filtered to give title compound **23** as fine beige solid (381 mg, 71%, over two steps): m.p. 174–175 °C; IR (ATR, cm^{-1}) ν_{max} 3346, 3307, 3149, 3117, 3072, 3048, 2777, 1634, 1548, 1466, 1347, 1237, 1157, 984, 783, 673, 427; ^1H NMR (400 MHz, DMSO-d_6) δ 13.55 (1H, brs), 8.36 (1H, d, J = 4.4, Hz), 8.18 (1H, d, J = 8.1 Hz), 7.60 (1H, d, J = 8.4 Hz), 7.40 (1H, t, J = 7.6 Hz), 7.23 (1H, t, J = 7.5 Hz), 2.82 (3H, d, J = 4.7 Hz); ^{13}C NMR (100 MHz, DMSO-d_6) δ 162.7, 141.0, 138.4, 126.4, 121.9, 121.6, 121.4, 110.6, 25.5; HRMS (ESI) *m/z* $[M+H]^+$ Calcd for $C_9H_{10}N_3O$ 176.0818, found 176.0822 (2.3 ppm).

^{1}H NMR (400 MHz, DMSO-d_6) **23**

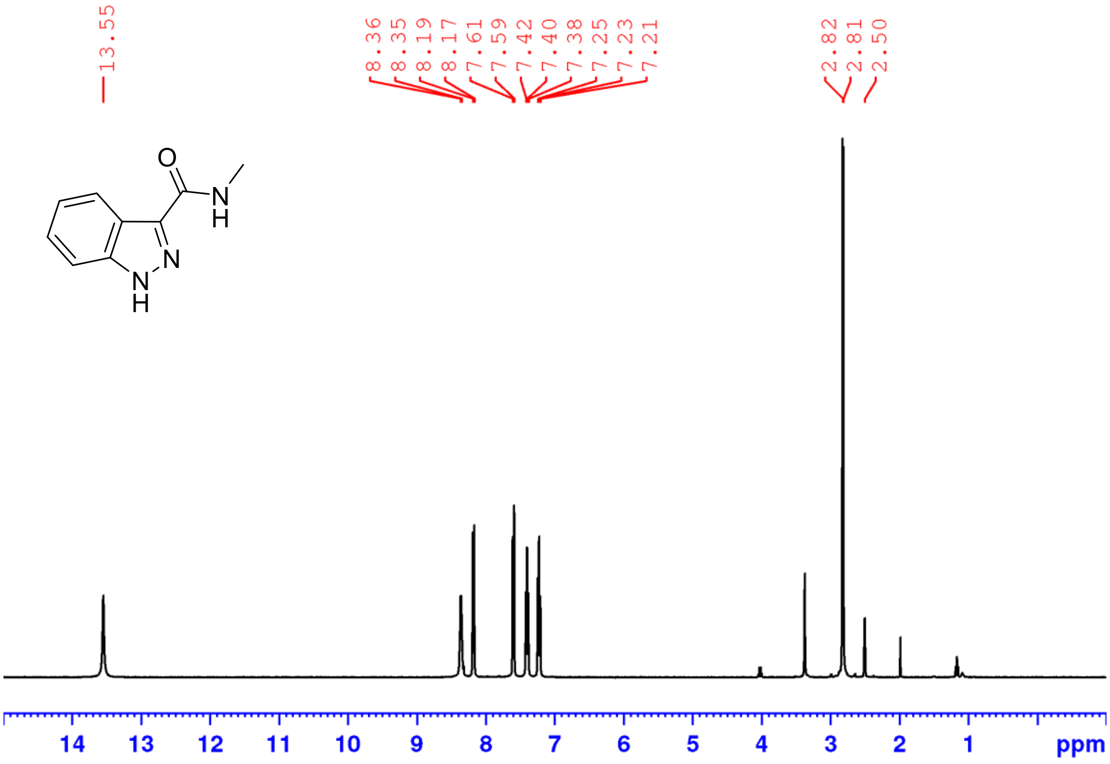

^{13}C NMR (100 MHz, DMSO-d_6) **23**

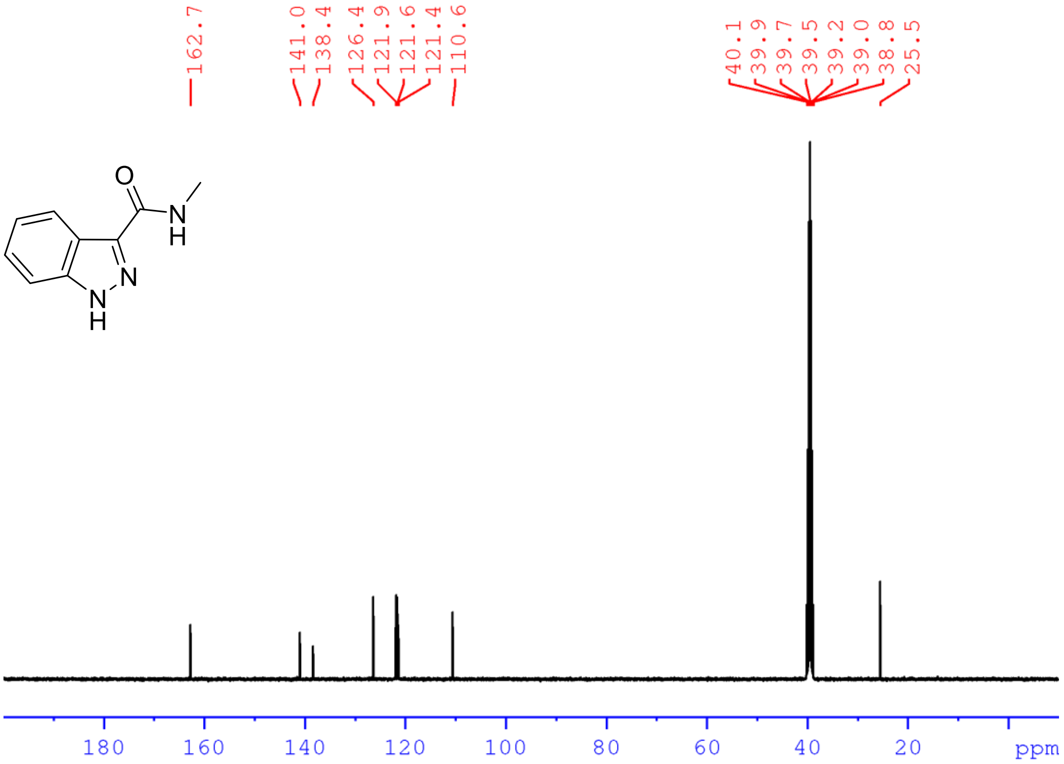

(1*H*-Indazol-3-yl)(pyrrolidin-1-yl)methanone (24)

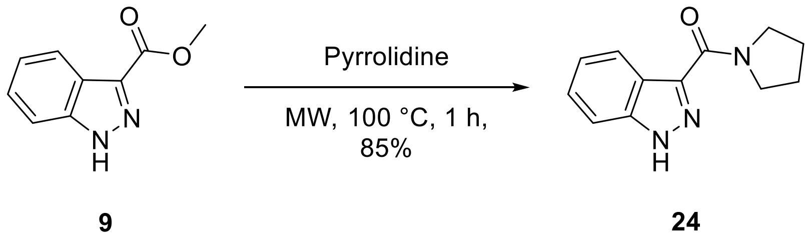

To a 20 mL microwave reaction vial was added methyl ester **9** (1 g, 5.68 mmol) and pyrrolidine (4 mL, 48.7 mmol). The vessel was then placed in a Discover SP® microwave reactor (CEM) and heated to 100 °C and stirred for 1 h. Upon cooling to room temperature, the resulting solution was concentrated in vacuo to give an orange oily solid. The crude product was then triturated with CH_2Cl_2 (10 mL) and the resulting solids filtered under vacuum to afford title compound **24** as a fine beige solid (1.043 g, 85%): m.p. 193–194 °C (EtOH); IR (ATR, cm^{-1}) ν_{max} 3151, 3118, 3047, 2969, 2925, 2887, 1589, 1566, 1488, 1466, 1450, 1341, 1320, 1147, 764, 735, 433; ^{1}H NMR (300 MHz, DMSO-d_6) δ 13.42 (1H, brs), 8.15 (1H, d, J = 8.2 Hz), 7.60 (1H, d, J = 8.4 Hz), 7.40 (1H, ddd, J = 8.3, 7.0, 1.1 Hz), 7.21 (1H, ddd, J = 7.9, 7.1, 0.8 Hz), 3.94 (2H, t, J = 6.5 Hz), 3.57 (2H, t, J = 6.6 Hz), 1.96–1.79 (4H, m); ^{13}C NMR (75 MHz, DMSO-d_6) δ 161.5, 140.3, 139.5, 126.4, 122.9, 122.0, 121.6, 110.3, 48.4, 46.4, 26.1, 23.4; HRMS (ESI) m/z [M–H]$^+$ Calcd for $C_{12}H_{14}N_3O$ 216.1131, found 216.1122 (− 4.2 ppm).

^{1}H NMR (300 MHz, DMSO-d_6) **24**

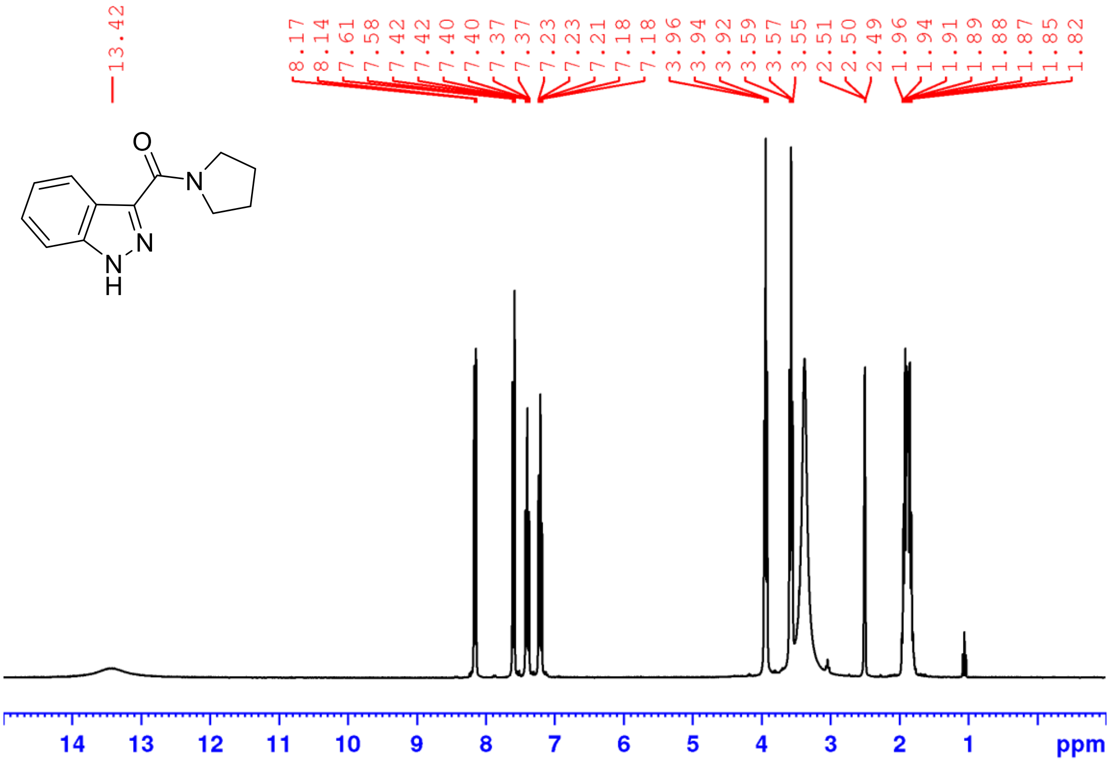

^{13}C NMR (75 MHz, DMSO-d_6) **24**

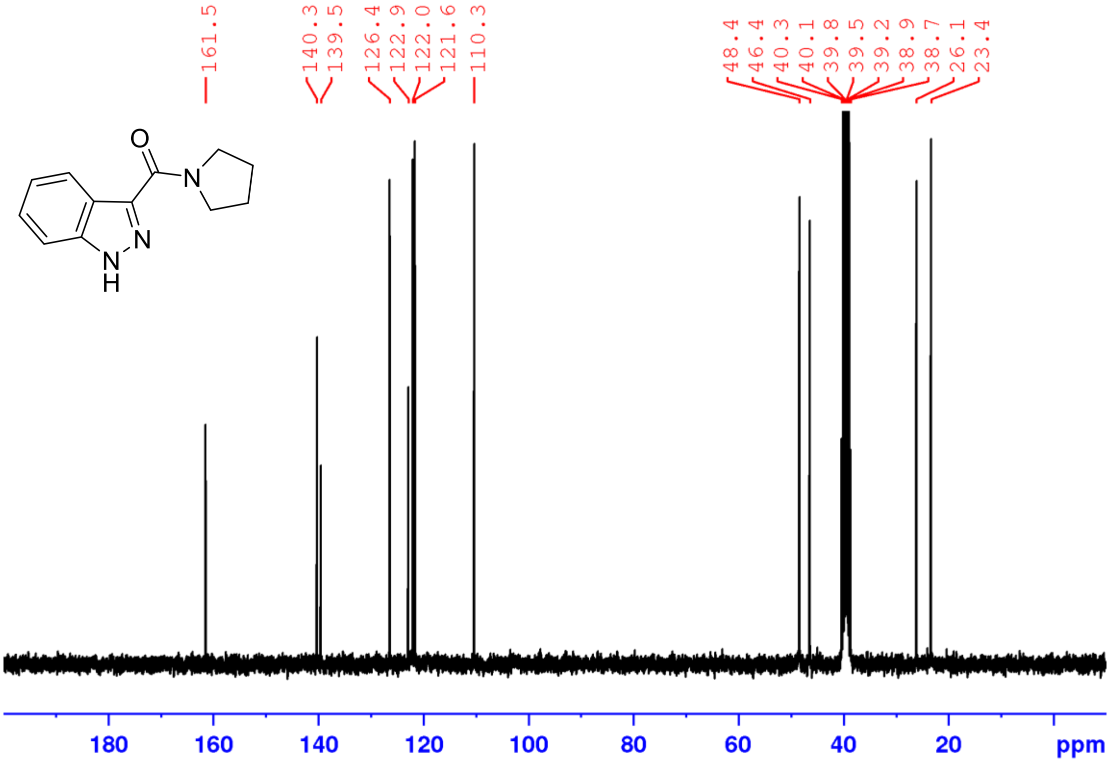

1*H*-indazole *N*-alkylation

General Procedure (A):

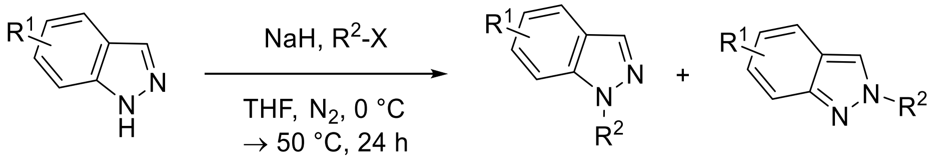

To an oven-dried 50 mL round bottom flask was added the appropriately substituted indazole (1 mmol) and THF (5 mL). The resulting solution was cooled to 0 °C and treated with NaH (26 mg, 1.1 mmol) and allowed to stir at 0 °C for a further 1 h. To the cooled suspension was added alkylating reagent, R^2-X (1.2 mmol). The mixture was heated to 50 °C for a further 24 h. The reaction mass was treated with MeOH (2.5 mL) and concentrated under reduced pressure. The resulting crude residue was dissolved in EtOAc (25 mL), washed with sat. aq. $Na_2S_2O_3$ (10 mL), H_2O (10 mL), and brine (10 mL). The organic layer was dried over $MgSO_4$ and concentrated in vacuo to give the crude product which was further purified using wet flash column chromatography to furnish the corresponding *N*-alkylated indazole(s).

General Procedure (B):

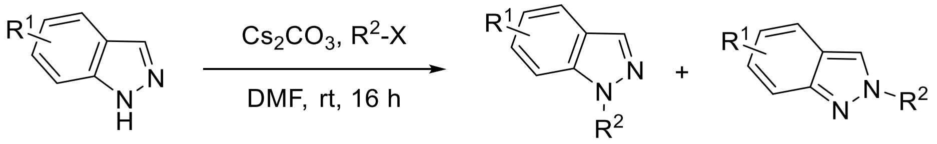

To a 50 mL round bottom flask was added the appropriately substituted indazole (1 mmol) and DMF (5 mL). The resulting solution was treated with Cs_2CO_3 (489 mg, 1.5 mmol) and allowed to stir at room temperature for a further 30 min. To the suspension was added alkylating reagent, R^2-X (1.2 mmol), and the mixture was stirred at room temperature for a further 16 h. The reaction mass was diluted with EtOAc (20 mL) and washed with brine (40 mL), sat. aq. $Na_2S_2O_3$ (10 mL) and brine (40 mL × 2). The organic layer was dried over $MgSO_4$ and concentrated under reduced pressure to afford crude product which was further purified using wet flash column chromatography to yield the corresponding *N*-alkylated indazole(s).

Note: Order of Elution of *N*-1 and *N*-2 Regioisomers using Wet Flash Column Chromatography

Following General Procedure A or B, the *N*-1 regioisomer typically eluted before the corresponding *N*-2 regioisomer, using wet flash column chromatography. However, the order of elution was *reversed* for 1*H*-indazole and analogous derivatives bearing a nitro or carboxymethyl substituent at the C-3 position, where the *N*-2 regioisomer eluted first and was then followed by the corresponding *N*-1 regioisomer.

Methyl 1-*n*-pentyl-1*H*-indazole-3-carboxylate (10)

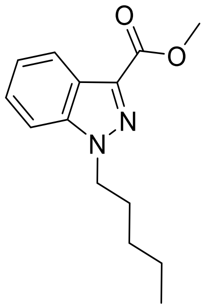

10

Following **General Procedure A** and/or **B**, wet flash column chromatography (EtOAc/hexane, 1:4) furnished title compound **10** (R_f = 0.40) as a yellow oil: IR (ATR, cm^{-1}) v_{max} 2955, 2932, 2861, 1709, 1616, 1477, 1215, 1158, 1117, 750, 643, 432; ^{1}H NMR (400 MHz, $CDCl_3$) δ 8.24 (1H, d, *J* = 8.2 Hz), 7.48 (1H, d, *J* = 8.4 Hz), 7.44 (1H, ddd, *J* = 7.3, 6.5, 0.8 Hz), 7.32 (1H, ddd, *J* = 7.9 Hz, 6.8, 1.1 Hz), 4.47 (2H, t, *J* = 7.4 Hz), 4.04 (3H, s), 1.97 (2H, quint, *J* = 7.4 Hz), 1.40–1.25 (4H, m), 0.88 (3H, t, *J* = 7.0 Hz); ^{13}C NMR (100 MHz, $CDCl_3$) δ 163.1, 140.4, 134.4, 126.7, 123.7, 123.0, 122.2, 109.6, 52.0, 50.0, 29.6, 28.9, 22.2, 13.9; HRMS (ESI) *m/z* $[M+H]^+$ Calcd for $C_{14}H_{19}N_2O_2$ 247.1441, found 247.1442 (0.4 ppm). Spectral data were in agreement with literature values [3].

^{1}H NMR (400 MHz, $CDCl_3$) **10**

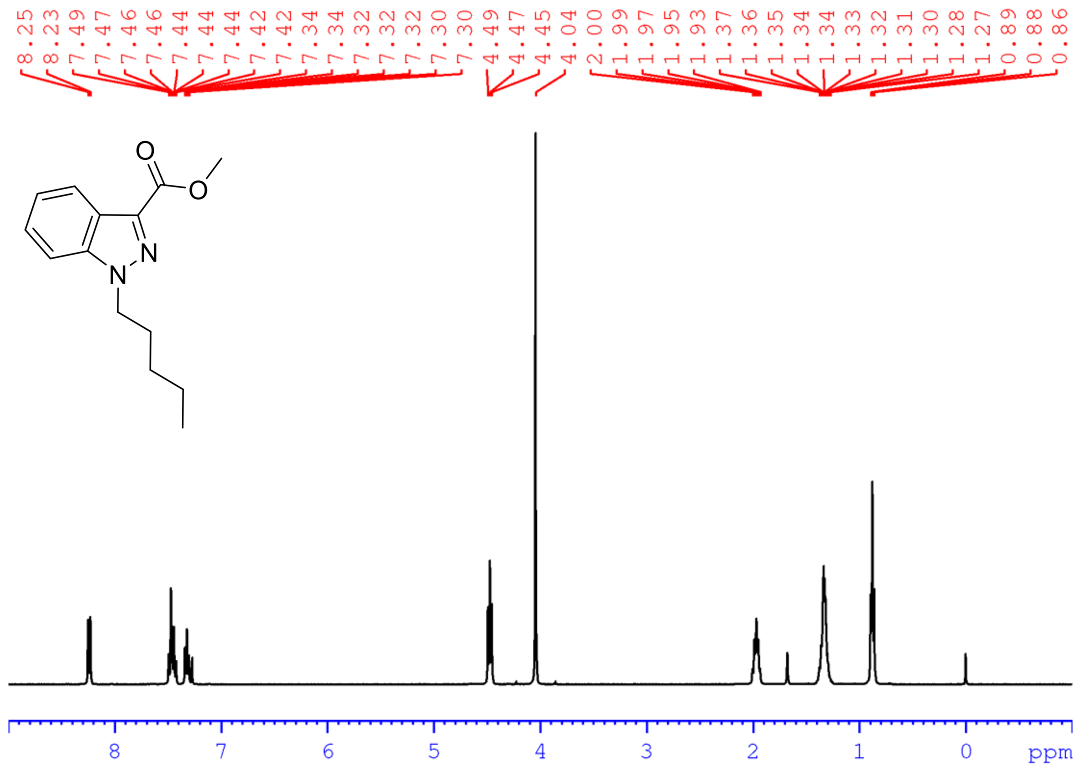

^{13}C NMR (100 MHz, $CDCl_3$) **10**

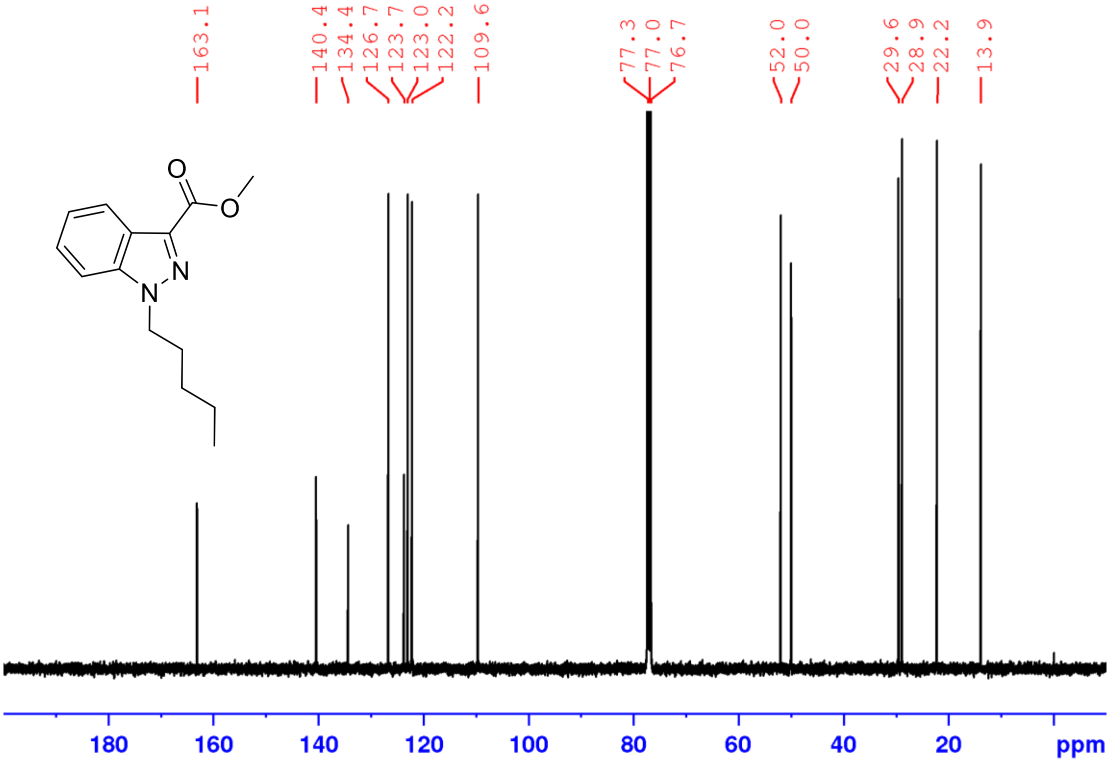

Methyl 2-*n*-pentyl-2*H*-indazole-3-carboxylate (11)

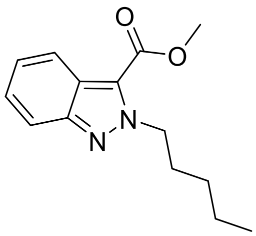

11

Following **General Procedure A** and/or **B**, wet flash column chromatography (EtOAc/hexane, 1:4) furnished title compound **11** (R_f = 0.63) as a yellow oil: IR (ATR, cm^{-1}) v_{max} 2955, 2931, 2861, 1710, 1464, 1278, 1205, 1077, 758, 434; ^{1}H NMR (400 MHz, $CDCl_3$) δ 8.02 (1H, d, J = 8.3 Hz), 7.79 (1H, d, J = 8.6 Hz), 7.35 (1H, t, J = 7.6 Hz), 7.28 (1H, t, J = 7.9 Hz), 4.90 (2H, t, J = 7.5 Hz), 1.97 (2H, quint, J = 7.4 Hz), 1.38–1.37 (4H, m), 0.90 (3H, t, J = 6.4 Hz); ^{13}C NMR (100 MHz, $CDCl_3$) δ 160.7, 147.3, 126.2, 124.9, 123.5, 123.3, 121.4, 118.2, 53.8, 51.9, 30.7, 28.8, 22.3, 14.0; HRMS (ESI) *m/z* $[M+H]^+$ Calcd for $C_{14}H_{19}N_2O_2$ 247.1441, found 247.1445 (1.6 ppm). Spectral data were in agreement with literature values [12].

^{1}H NMR (400 MHz, $CDCl_3$) **11**

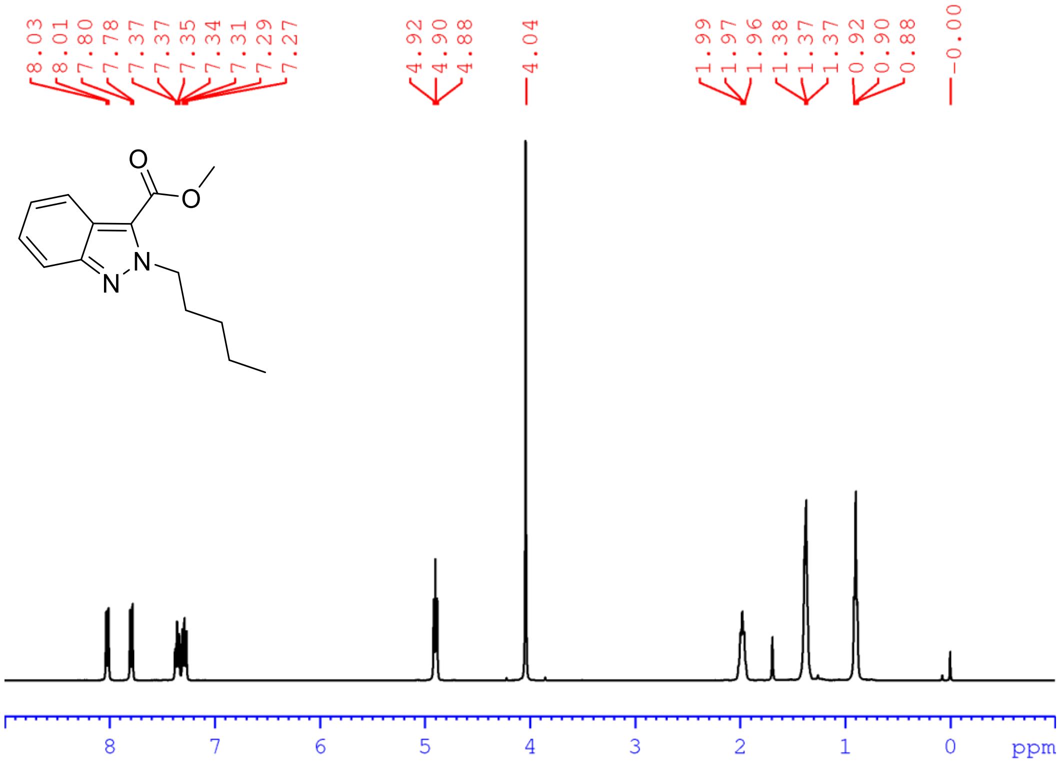

^{13}C NMR (100 MHz, $CDCl_3$) **11**

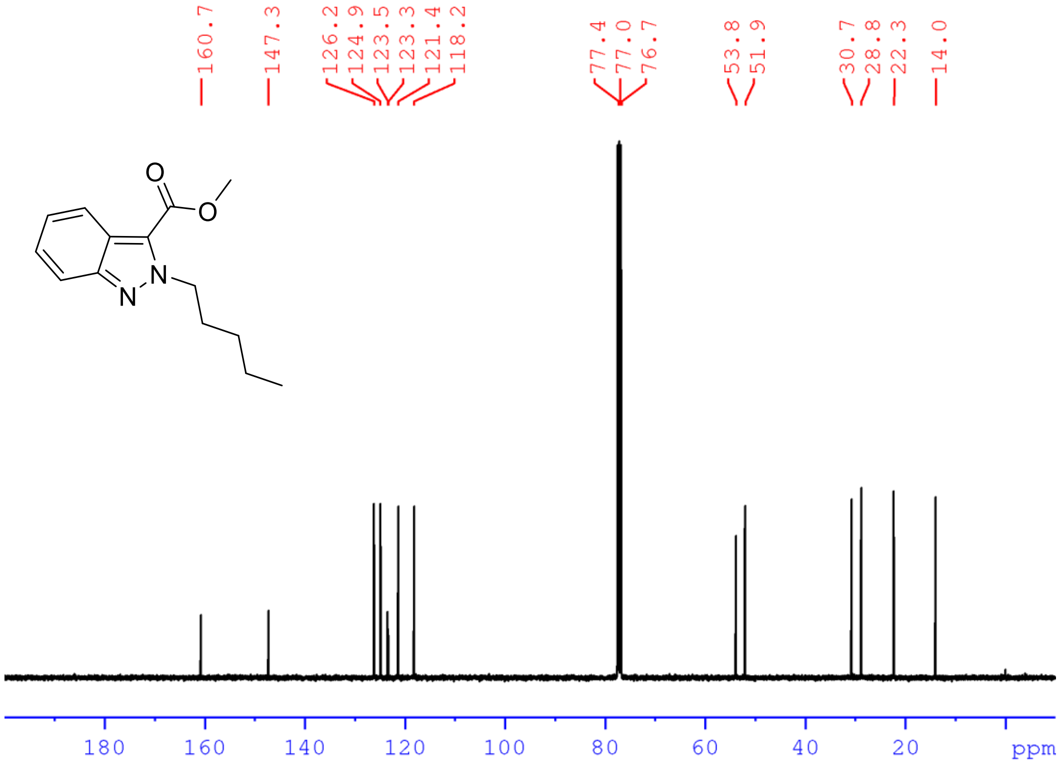

N-Alkylation of Methyl Ester 9 under Mitsunobu Conditions

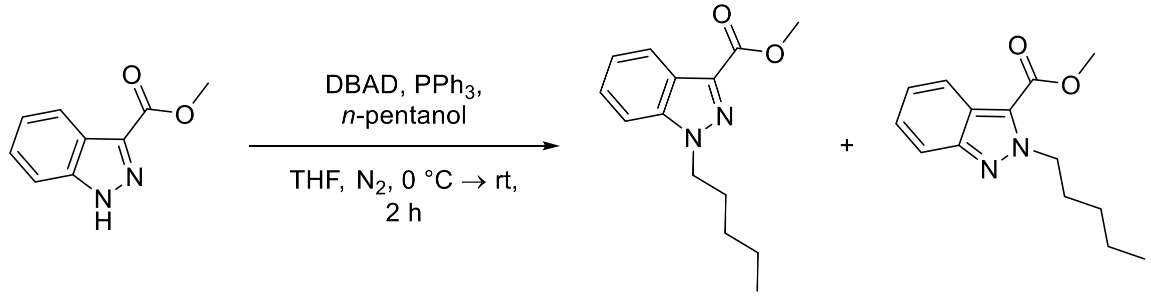

9 **10** **11**

To an oven-dried 50 mL round bottom flask was added DBAD (261 mg, 1.5 mmol) and THF (10 mL). The resulting bright yellow solution was cooled to 0 °C and then treated with PPh_3 (393 mg, 1.5 mmol) and allowed to stir for a further 30 min at 0 °C. The resulting colorless mixture was treated with *n*-pentanol (0.11 mL, 1 mmol) and ester **9** (264 mg, 1.5 mmol). The reaction mixture was warmed to room temperature and allowed to stir for a further 2 h. The resulting solution was concentrated under reduced pressure to afford a viscous amber oil. The crude residue was dissolved in $CHCl_3$ (5 mL) and treated dropwise with CF_3CO_2H (1.5 mL, 15 mmol) and stirred for 18 h at room temperature. The resulting solution was concentrated under reduced pressure and redissolved in EtOAc (25 mL). The organic phase was then washed with sat. aq. $NaHCO_3$ (10 mL × 2), H_2O (10 mL), brine (10 mL), dried over $MgSO_4$, and concentrated under reduced pressure to afford crude product which was further purified using wet flash column chromatography (EtOAc/hexane, 1:4) to give *N*-1 regioisomer **10** (R_f = 0.40) as a colorless oil (49 mg, 20%) and the corresponding *N*-2 regioisomer **11** (R_f = 0.63) as a colorless oil (143 mg, 58%).

1-*n*-Pentyl-1*H*-indazole (25)

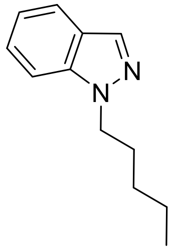

Following **General Procedure A** and/or **B**, wet flash column chromatography (EtOAc/hexane, 1:19) gave the title compound **25** (R_f = 0.10) as an amber oil: IR (ATR, cm^{-1}) ν_{max} 3061, 2956, 2930, 2871, 2859, 1616, 1475, 751, 737, 635, 428; ^1H NMR (400 MHz, $CDCl_3$) δ 7.99 (1H, d, J = 0.8 Hz), 7.73 (1H, ddd, J = 8.1, 1.9, 1.0 Hz), 7.42–7.35 (2H, m), 7.14 (1H, ddd, J = 7.8, 6.6, 1.2 Hz), 4.38 (2H, t, J = 7.2 Hz), 1.93 (2H, quint, J = 7.3 Hz), 1.40–1.26 (4H, m), 0.86 (3H, t, J = 7.0 Hz); ^{13}C NMR (100 MHz, $CDCl_3$) δ 139.4, 132.7, 126.0, 124.0, 121.1, 120.3, 109.0, 48.9, 29.6, 29.0, 22.3, 14.0; HRMS (ESI) *m/z*: $[M+H]^+$ Calcd for $C_{12}H_{17}N_2$ 189.1386; found 138.1382 (− 2.4 ppm). Spectral data were in agreement with literature values [13].

^{1}H NMR (400 MHz, $CDCl_3$) **25**

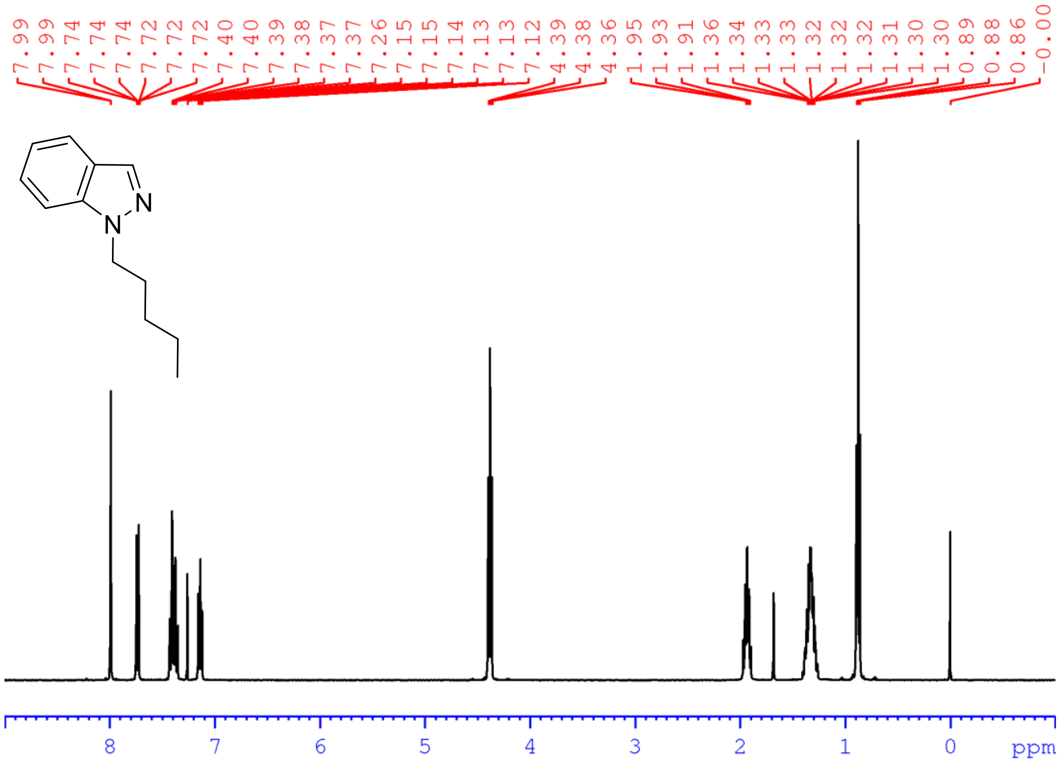

^{13}C NMR (100 MHz, $CDCl_3$) **25**

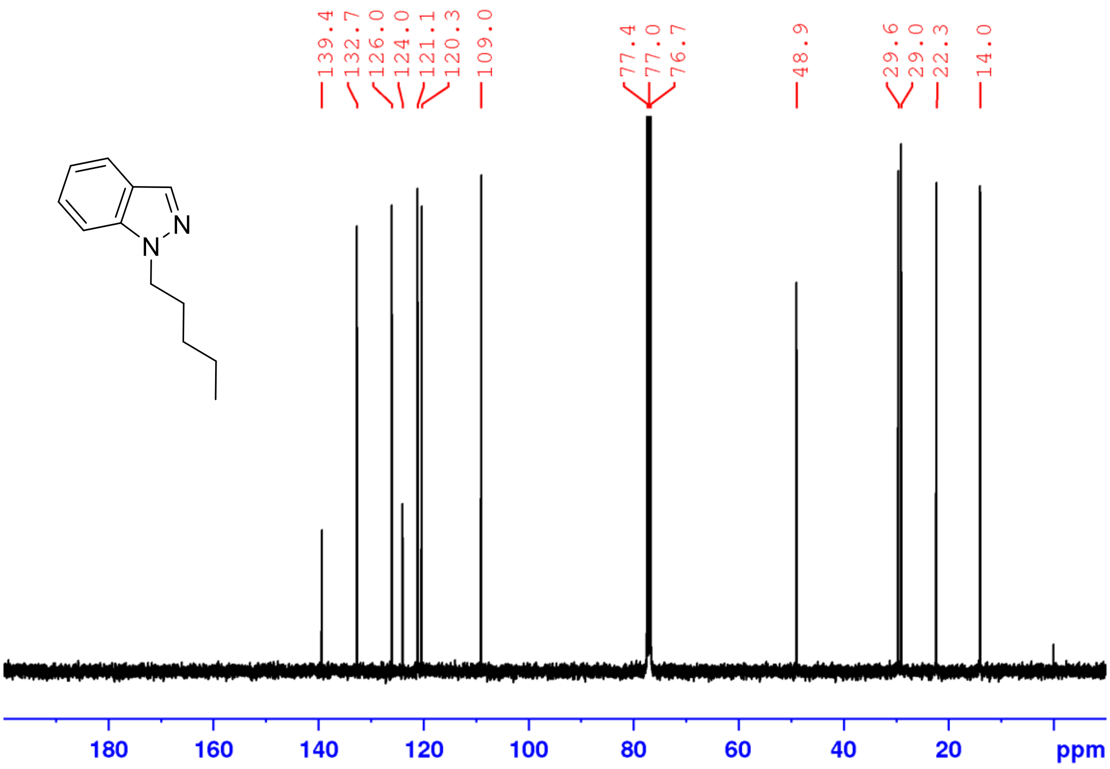

2-*n*-Pentyl-2*H*-indazole (26)

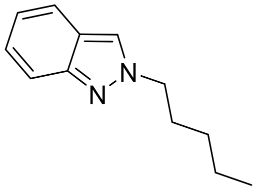

Following **General Procedure A** and/or **B**, wet flash column chromatography (EtOAc/hexane, 1:19) gave the title compound **26** (R_f = 0.23) as colorless oil: IR (ATR, cm^{-1}) ν_{max} 3099, 3060, 2955, 2931, 2860, 1628, 1156, 1140, 781, 754, 732, 432; ^{1}H NMR (400 MHz, $CDCl_3$) δ 7.88 (1H, s), 7.72 (1H, dd, *J* = 8.7, 0.8 Hz), 7.64 (1H, d, *J* = 8.4 Hz), 7.27 (1H, ddd, *J* = 8.7, 6.7, 1.1 Hz), 7.07 (1H, ddd, *J* = 8.4, 6.6, 0.9 Hz), 4.38 (2H, t, *J* = 7.2 Hz), 2.00 (2H, quint, *J* = 7.4 Hz), 1.40–1.25 (4H, m), 0.88 (3H, t, *J* = 7.1 Hz); ^{13}C NMR (100 MHz, $CDCl_3$) δ 148.7, 125.6, 122.4, 121.6, 121.4, 120.0, 117.3, 53.7, 30.3, 28.7, 22.1, 13.8; HRMS (ESI) *m/z*: $[M+H]^+$ Calcd for $C_{12}H_{17}N_2$ 189.1386; found 189.1379 (− 3.4 ppm). Spectral data were in agreement with literature values [13].

^{1}H NMR (400 MHz, $CDCl_3$) **26**

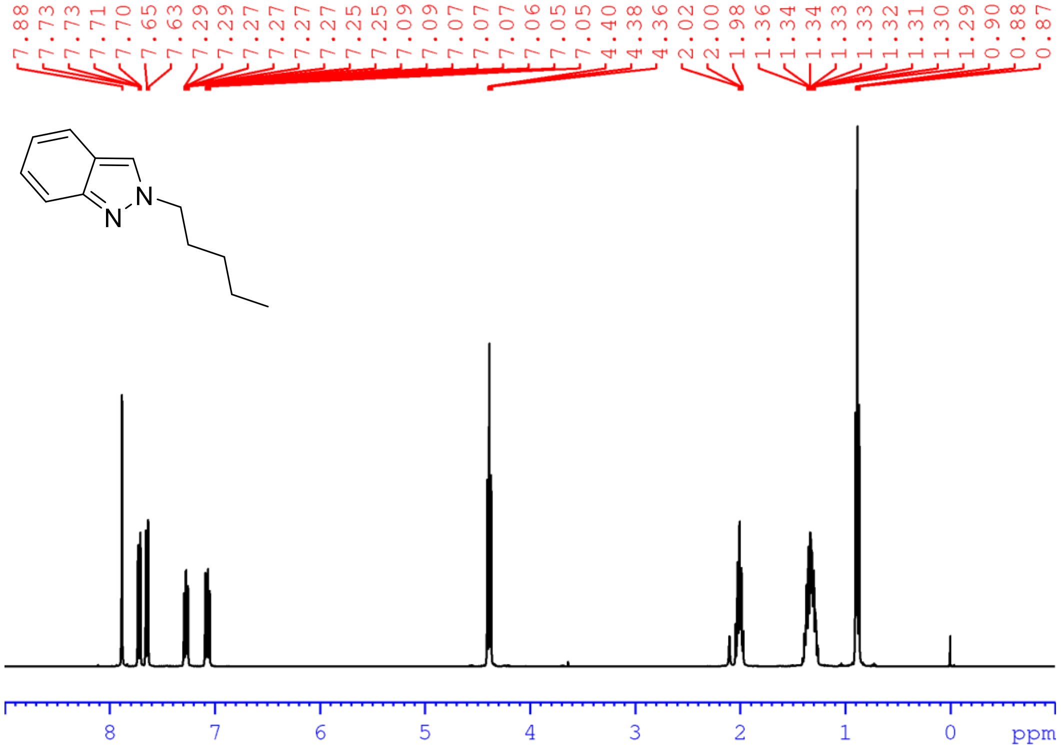

^{13}C NMR (100 MHz, $CDCl_3$) **26**

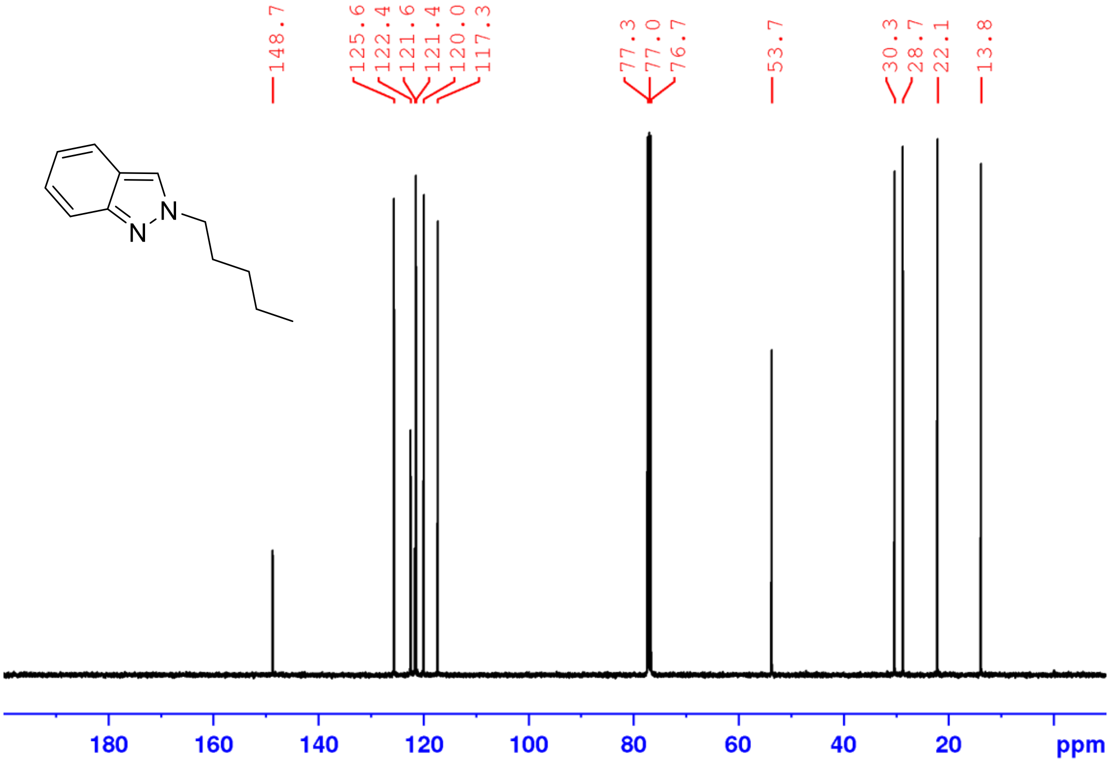

3-Methyl-1-*n*-pentyl-1*H*-indazole (27)

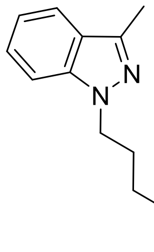

Following **General Procedure A** and/or **B**, wet flash column chromatography (EtOAc/hexane, 1:4) gave the title compound **27** (R_f = 0.67) as a colorless oil: IR (ATR, cm^{-1}) ν_{max} 3058, 2956, 2928, 2859, 1615, 1506, 1454, 1349, 1184, 1135, 1078, 1009, 739, 430; ^{1}H NMR (400 MHz, $CDCl_3$) δ 7.64 (1H, d, J = 8.1 Hz), 7.37–7.32 (2H, m), 7.09 (1H, ddd, J = 7.1, 5.4, 2.3 Hz), 4.29 (2H, t, J = 7.2 Hz), 2.57 (3H, s), 1.89 (2H, quint, J = 7.4 Hz), 1.37–1.26 (4H, m), 0.87 (3H, t, J = 7.0 Hz); ^{13}C NMR (100 MHz, $CDCl_3$) δ 141.0, 140.2, 125.9, 123.1, 120.3, 119.4, 108.8, 48.6, 29.7, 29.0, 22.3, 13.9, 11.9; HRMS (ESI) *m/z*: $[M+H]^+$ Calcd for $C_{13}H_{19}N_2$ 203.1543, found 203.1545 (1.1 ppm).

^{1}H NMR (400 MHz, $CDCl_3$) **27**

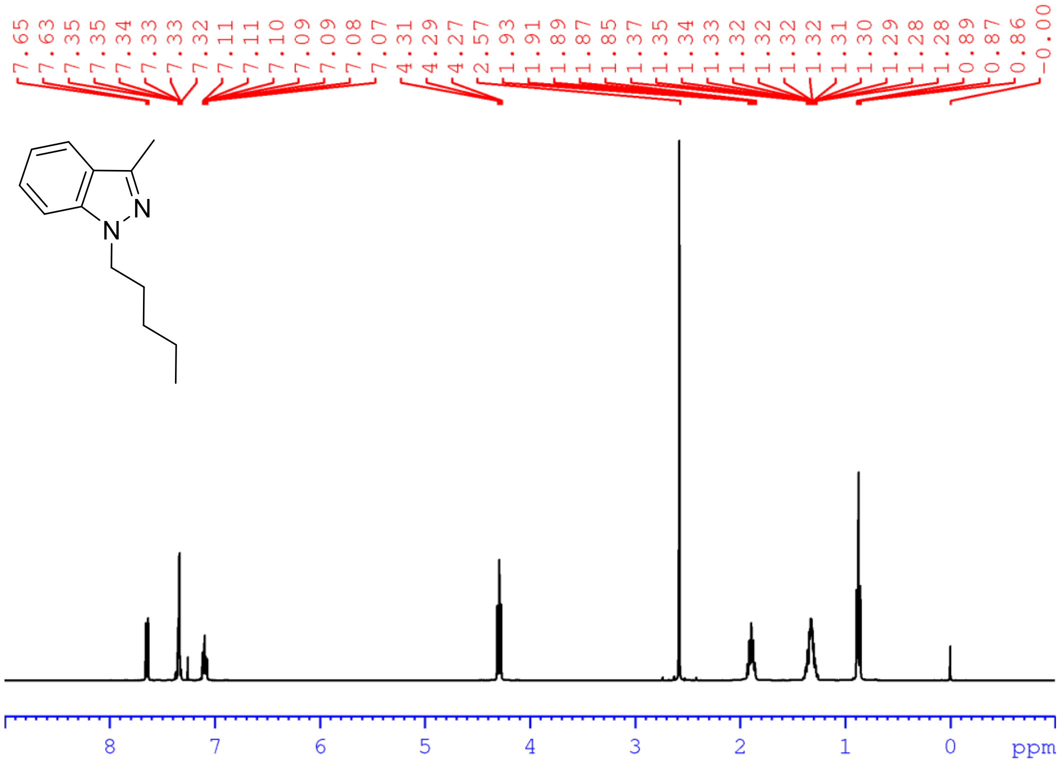

^{13}C NMR (100 MHz, $CDCl_3$) **27**

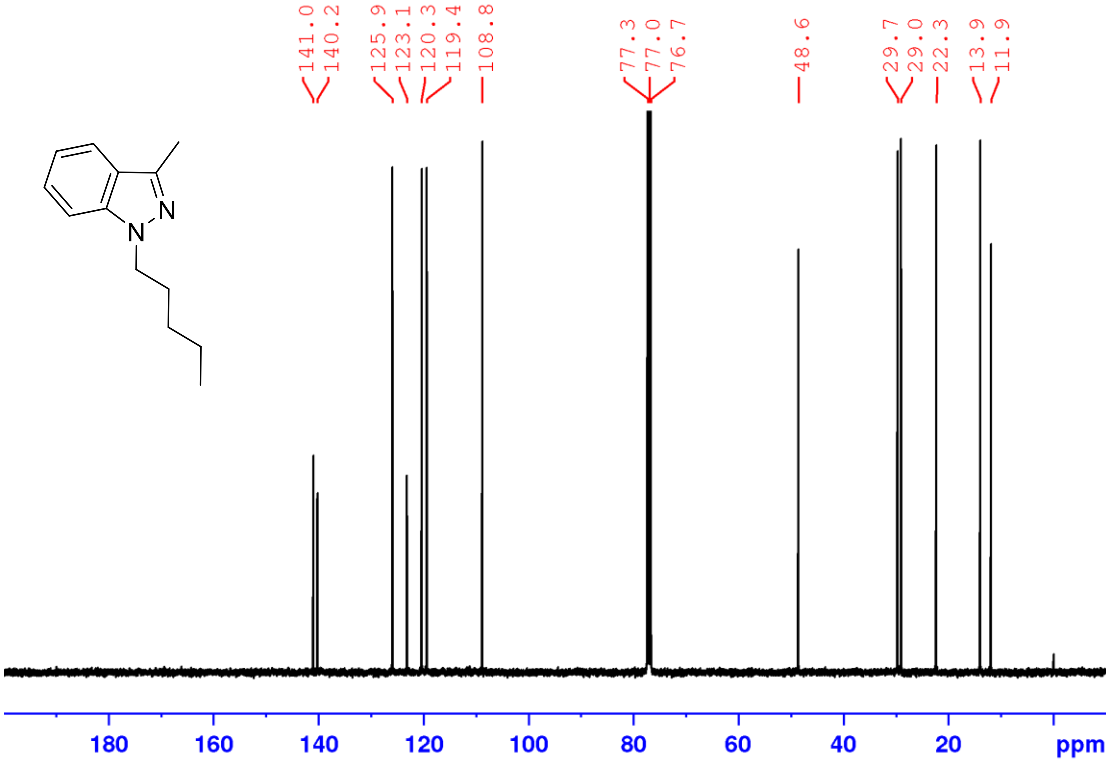

3-Methyl-2-*n*-pentyl-2*H*-indazole (28)

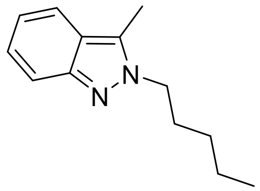

Following **General Procedure A** and/or **B**, wet flash column chromatography (EtOAc/hexane, 1:4) gave the title compound **28** (R_f = 0.43) as an amber oil: IR (ATR, cm^{-1}) ν_{max} 3058, 3019, 2955, 2929, 2860, 1630, 1455, 1366, 740, 433; ^{1}H NMR (400 MHz, $CDCl_3$) δ 7.64 (1H, d, J = 8.7 Hz), 8.40 (1H, d, J = 8.4 Hz), 7.25 (1H, ddd, J = 7.8, 6.7, 0.8 Hz), 7.03–6.99 (1H, m), 4.31 (2H, t, J = 7.4 Hz), 2.59 (3H, s), 1.94 (2H, quint, J = 7.4 Hz), 1.40–1.25 (4H, m), 0.89 (3H, t, J = 6.9 Hz); ^{13}C NMR (100 MHz, $CDCl_3$) δ 147.6, 130.6, 125.8, 120.9, 120.1, 119.5, 117.0, 50.2, 30.1, 28.8, 22.2, 13.9, 9.8; HRMS (ESI) *m/z*: $[M+H]^+$ Calcd for $C_{13}H_{19}N_2$ 203.1543, found 203.1542 (– 0.3 ppm).

^{1}H NMR (400 MHz, $CDCl_3$) **28**

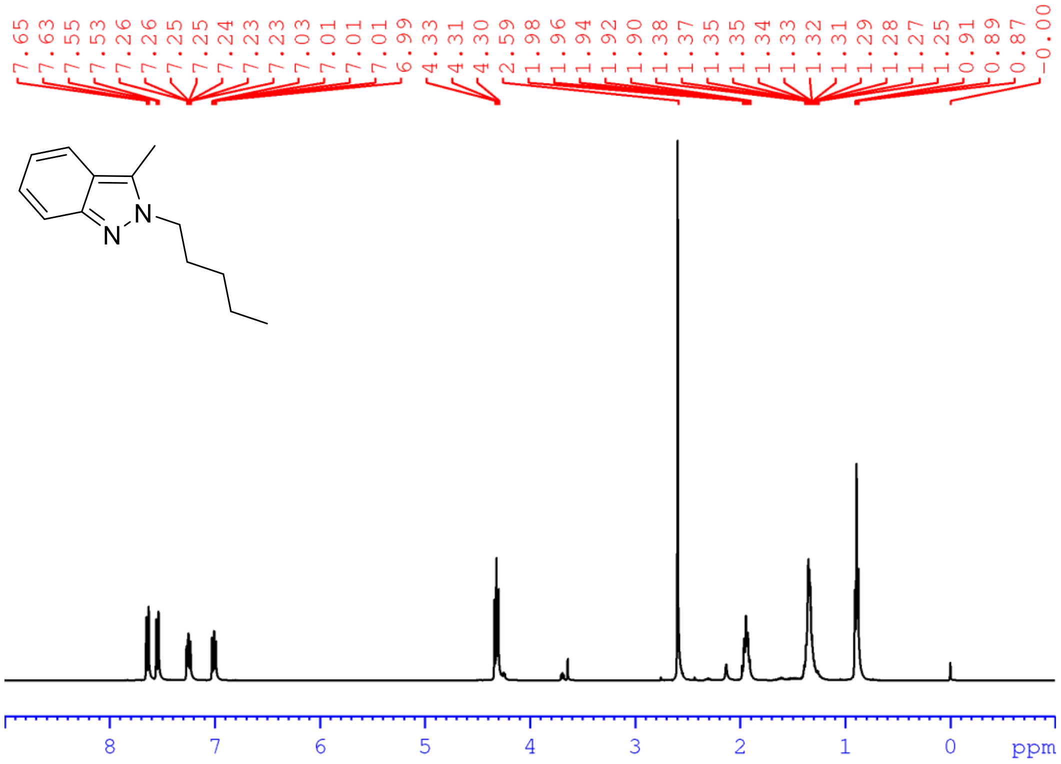

^{13}C NMR (100 MHz, $CDCl_3$) **28**

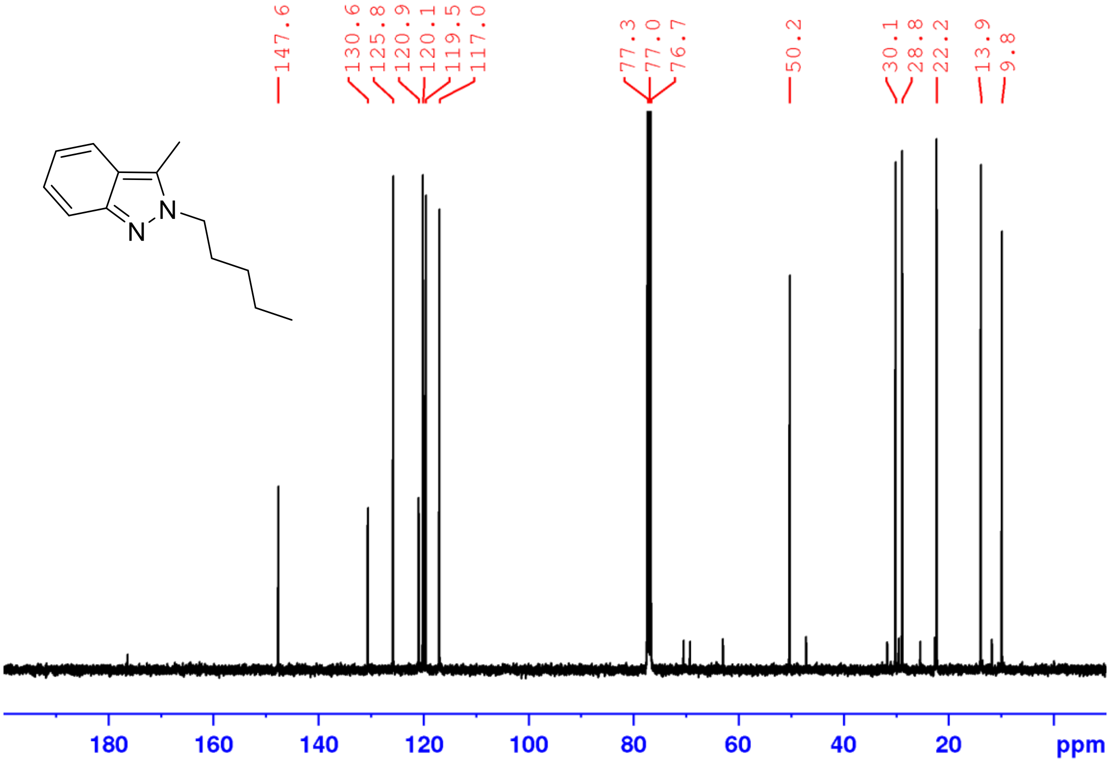

3-*tert*-Butyl-1-*n*-pentyl-1*H*-indazole (29)

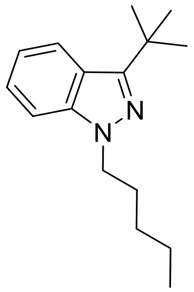

Following **General Procedure A** and/or **B**, wet flash column chromatography (EtOAc/hexane, 1:9) gave the title compound **29** (R_f = 0.53) as a colorless oil: IR (ATR, cm^{-1}) ν_{max} 3054, 2958, 2928, 2870, 1612, 1500, 738, 430; ^{1}H NMR (300 MHz, $CDCl_3$) δ 7.86 (1H, d, J = 8.2 Hz), 7.35–7.24 (2H, m), 7.05 (1H, ddd, J = 8.0, 5.9, 1.9 Hz), 4.29 (2H, t, J = 7.3 Hz), 1.87 (2H, quint, J = 7.3 Hz), 1.52 (9H, s), 1.40–1.24 (4H, m), 0.88 (3H, t, J = 6.9 Hz); ^{13}C NMR (75 MHz, $CDCl_3$) δ 152.3, 140.9, 125.3, 122.3, 121.1, 119.9, 109.1, 48.5, 30.3, 29.5, 29.1, 22.3, 14.0; HRMS (ESI) *m/z*: $[M+H]^+$ Calcd for $C_{16}H_{25}N_2$ 245.2012, found 245.2012 (0.0 ppm).

^{1}H NMR (300 MHz, $CDCl_3$) **29**

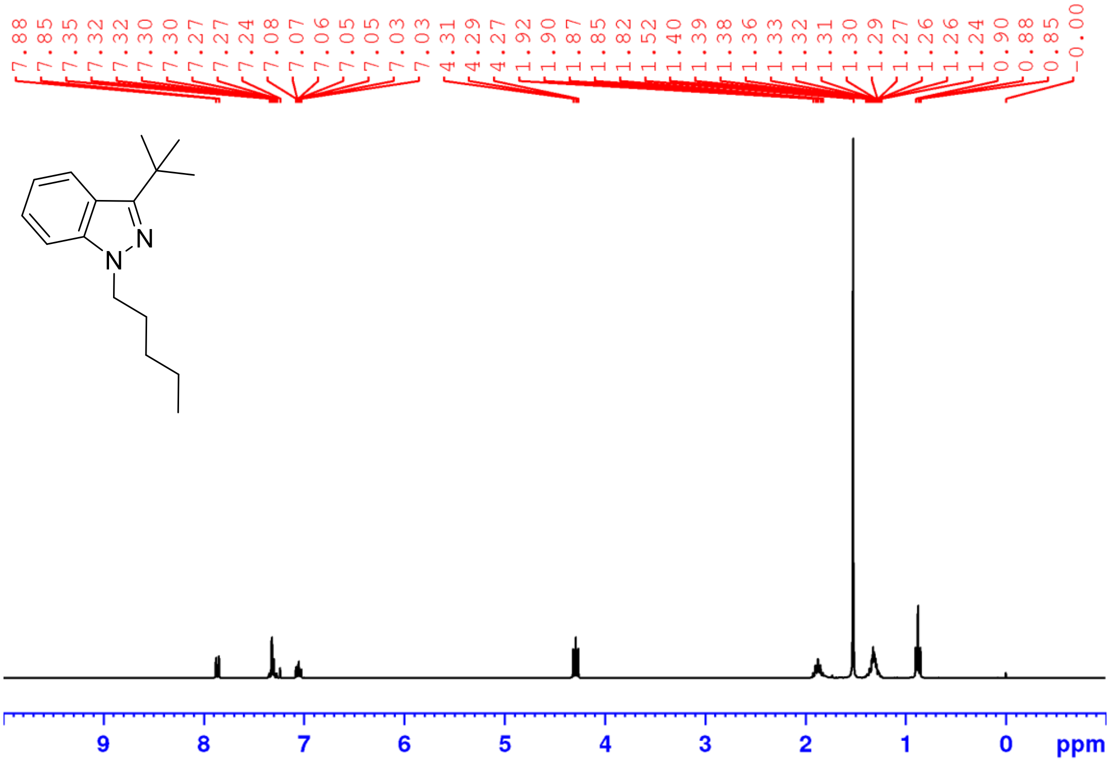

^{13}C NMR (75 MHz, $CDCl_3$) **29**

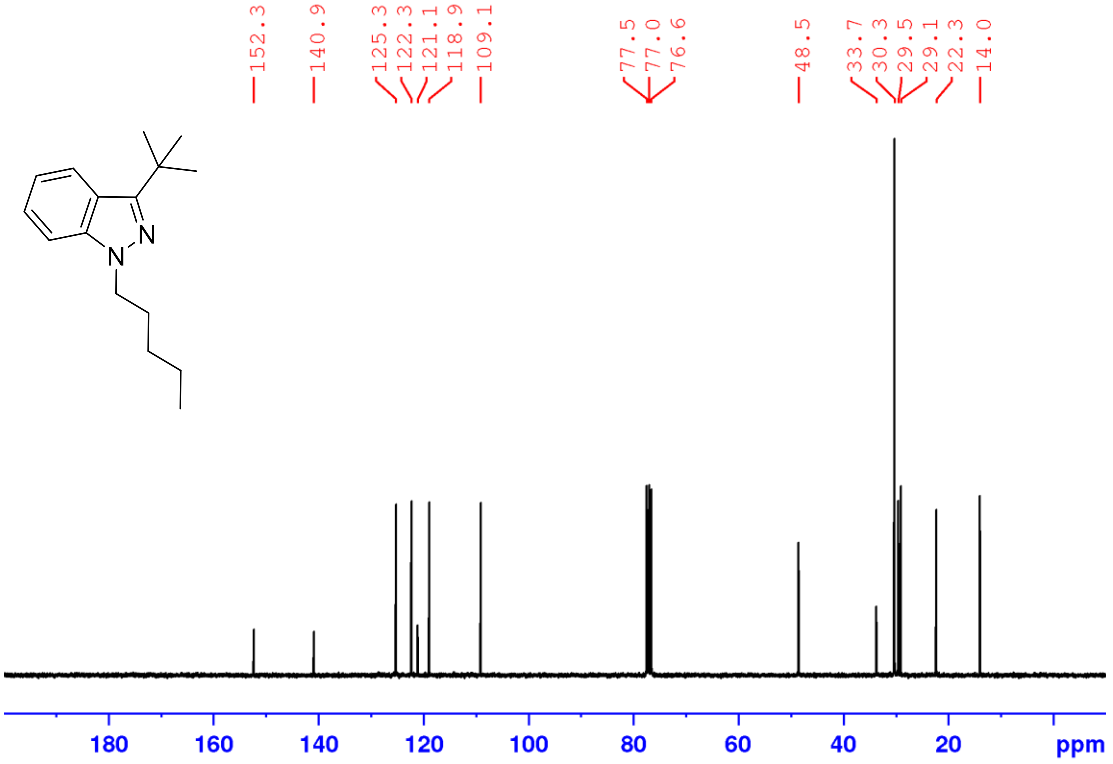

3-Phenyl-1-*n*-pentyl-1*H*-indazole (31)

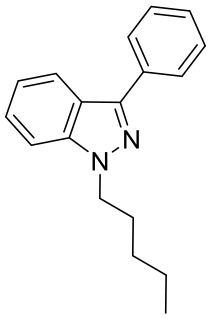

Following **General Procedure A** and/or **B**, wet flash column chromatography (EtOAc/hexane, 1:9) furnished the title compound **31** (R_f = 0.62) as a yellow oil: IR (ATR, cm^{-1}) ν_{max} 3057, 2956, 2930, 2870, 2858, 1613, 1490, 1304, 1150, 777, 741, 695, 666, 429; ^{1}H NMR (300 MHz, $CDCl_3$) δ 8.01–7.94 (3H, m), 7.50–7.45 (2H, m), 7.41–7.34 (3H, m), 7.17 (1H, ddd, *J* = 10.0, 8.0, 1.8 Hz), 4.39 (2H, t, *J* = 7.2 Hz), 1.96 (2H, quint, *J* = 7.3 Hz), 1.42–1.23 (4H, m), 0.88 (3H, t, *J* = 6.9 Hz); ^{13}C NMR (75 MHz, $CDCl_3$) δ 143.5, 140.9, 133.8, 128.7, 127.6, 127.4, 126.0, 121.6, 121.3, 120.7, 109.2, 48.9, 29.6, 29.0, 22.3, 13.9; HRMS (ESI) *m/z*: $[M+H]^+$ Calcd for $C_{18}H_{21}N_2$ 265.1699, found 265.1702 (1.1 ppm).

^{1}H NMR (300 MHz, $CDCl_3$) **31**

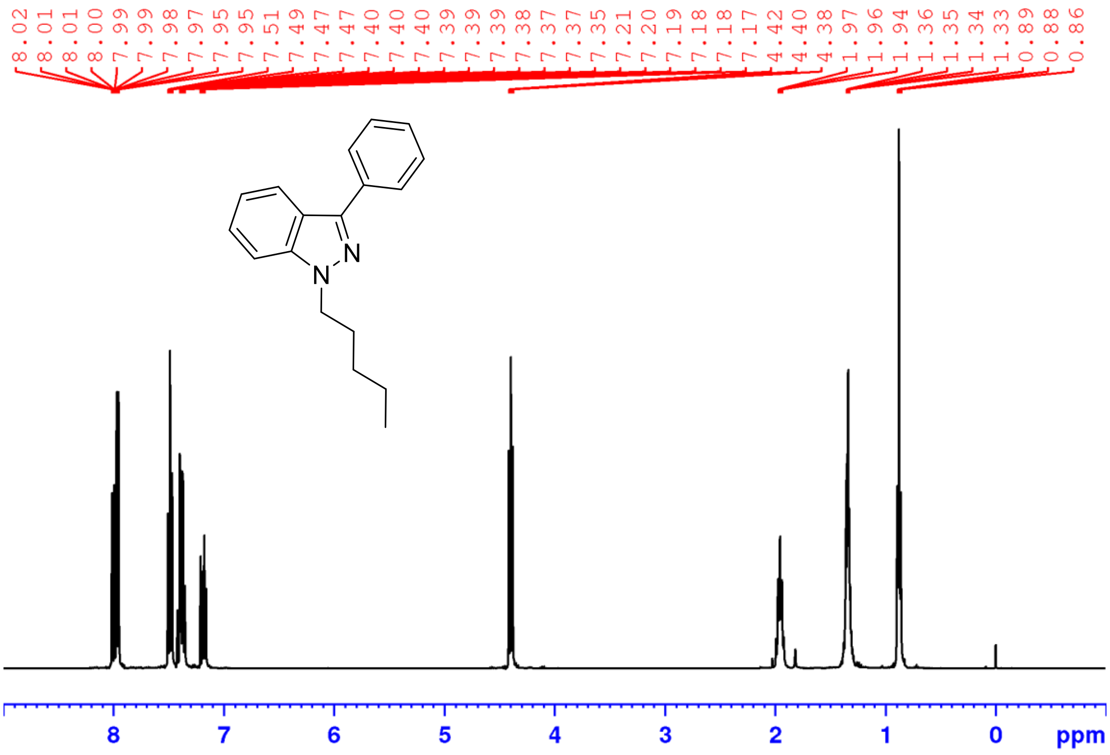

^{13}C NMR (75 MHz, $CDCl_3$) **31**

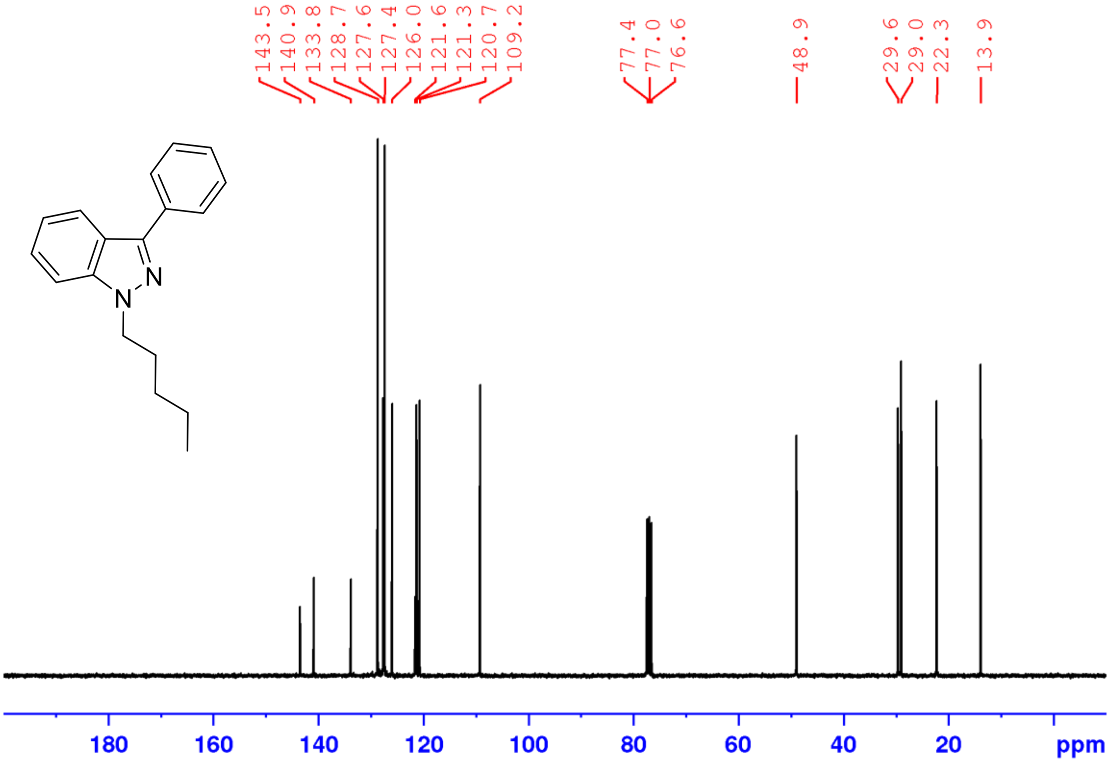

3-Phenyl-2-*n*-pentyl-2*H*-indazole (32)

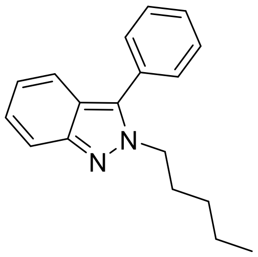

Following **General Procedure A** and/or **B**, wet flash column chromatography (EtOAc/hexane, 1:9) gave the title compound **32** (R_f = 0.30) as a colorless oil: IR (ATR, cm^{-1}) ν_{max} 3057, 2955, 2928, 2858, 2870, 1627, 1602, 1497, 1466, 1365, 1273, 1010, 755, 744, 699, 434; ^{1}H NMR (300 MHz, $CDCl_3$) δ 7.73 (1H, d, J = 8.7 Hz), 7.59 – 7.46 (6H, m), 7.31 (1H, ddd, J = 7.7, 6.6, 1.1 Hz), 7.06 (1H, ddd, J = 7.4, 6.6, 0.8 Hz), 4.41 (2H, t, J = 7.5 Hz), 1.96 (2H, quint, 7.4 Hz), 1.34–1.17 (4H, m), 0.83 (3H, t, J = 6.9 Hz); ^{13}C NMR (75 MHz, $CDCl_3$) δ 148.0, 135.8, 130.0, 129.7, 129.0, 128.7, 128.3, 126.2, 121.6, 121.2, 120.2, 117.1, 50.7, 30.5, 28.8, 22.1, 13.8; HRMS (ESI) *m/z*: $[M+H]^+$ Calcd for $C_{18}H_{21}N_2$ 265.1699, found 265.1704 (1.9 ppm).

^{1}H NMR (400 MHz, $CDCl_3$) **32**

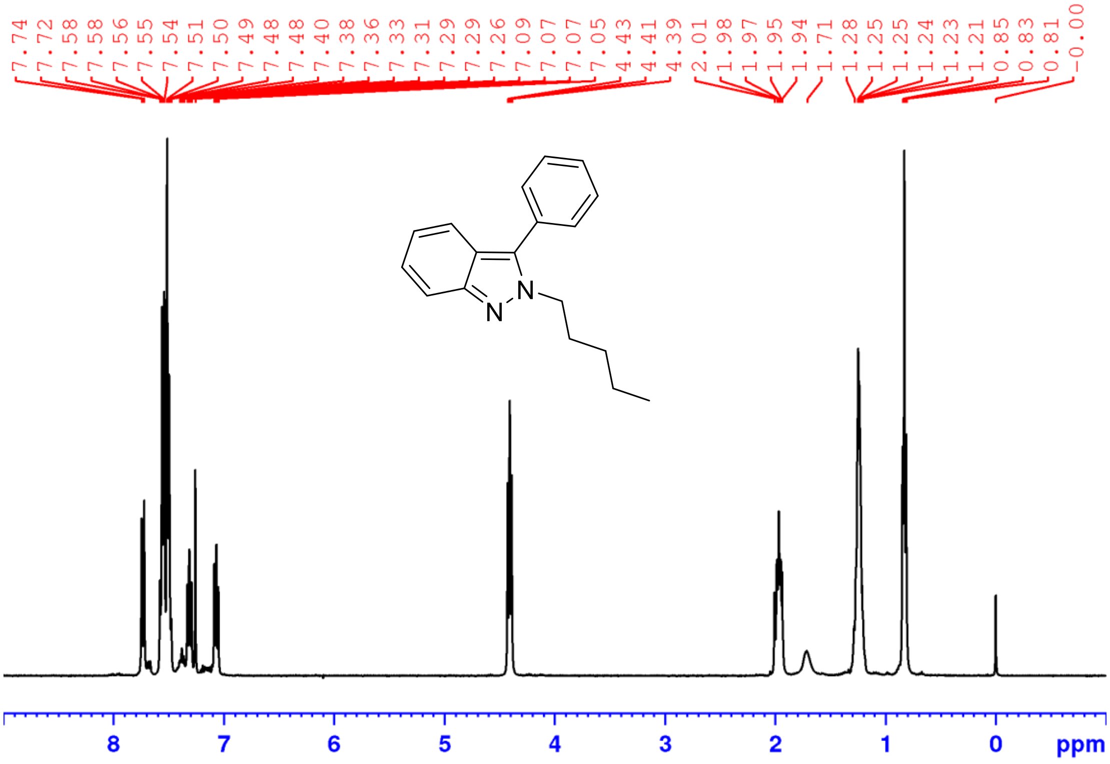

^{13}C NMR (100 MHz, $CDCl_3$) **32**

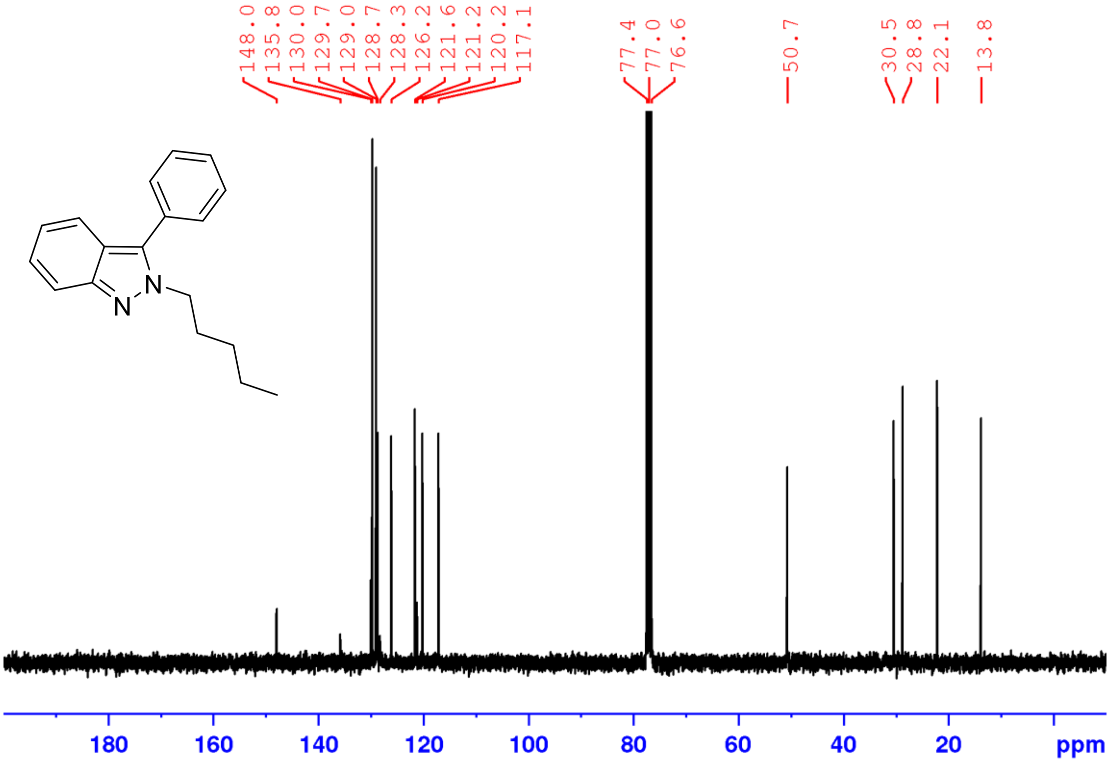

3-Iodo-1-*n*-pentyl-1*H*-indazole (33)

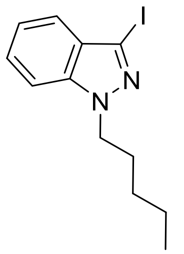

Following **General Procedure A** and/or **B**, wet flash column chromatography (EtOAc/hexane, 1:19) furnished the title compound **33** (R_f = 0.50) as a yellow oil: IR (ATR, cm^{-1}) ν_{max} 3059, 2955, 2929, 2860, 2858, 1614, 1459, 1319, 1173, 764, 740; ^{1}H NMR (400 MHz, $CDCl_3$) δ 7.48 (1H, d, J = 8.2 Hz), 7.43 (1H, ddd, J = 7.7 Hz, 6.7 Hz, 1.0 Hz), 7.37 (1H, d, J = 8.5 Hz), 7.20 (1H, ddd, J = 8.1, 6.7, 1.0 Hz), 4.38 (2H, t, J = 7.3 Hz), 1.92 (2H, quint, J = 7.3 Hz), 1.40–1.26 (4H, m), 0.87 (3H, t, J = 7.1 Hz); ^{13}C NMR (100 MHz, $CDCl_3$) δ 140.1, 128.2, 127.2, 121.7, 121.2, 109.2, 90.6, 49.6, 29.8, 29.0, 22.3, 14.0; HRMS (ESI) m/z $[M+H]^+$ Calcd for $C_{12}H_{16}IN_2$ 315.0358, found 315.0362 (1.3 ppm).

^{1}H NMR (400 MHz, $CDCl_3$) **33**

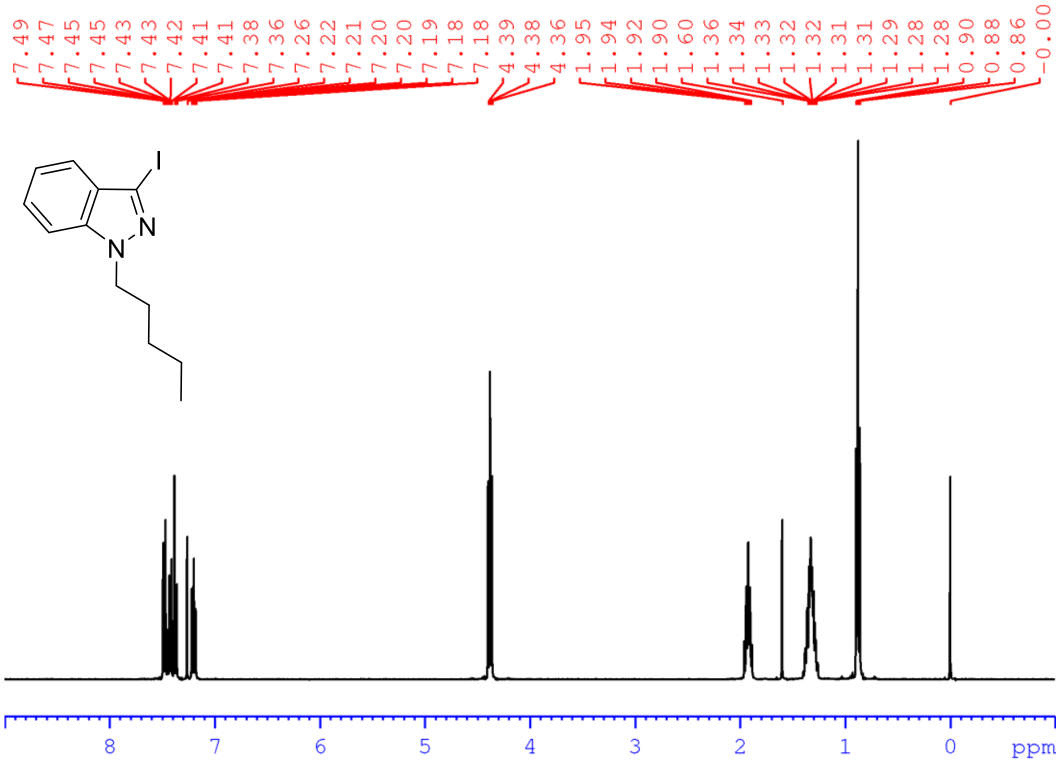

^{13}C NMR (100 MHz, $CDCl_3$) **33**

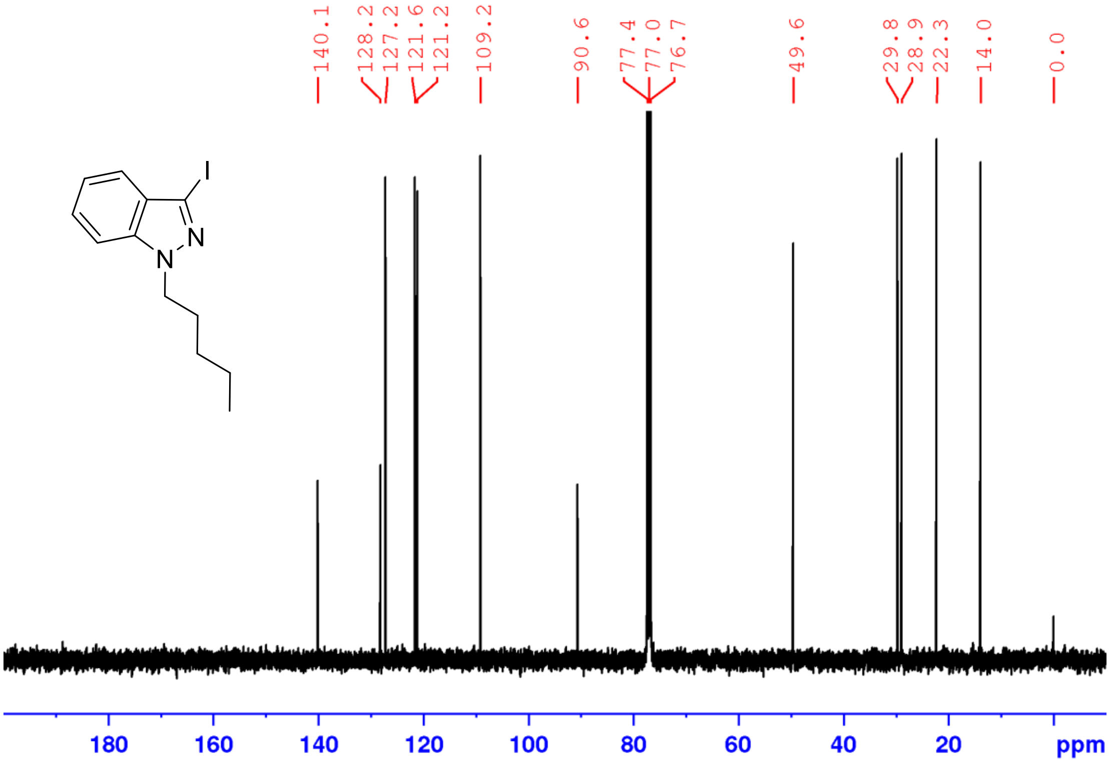

3-Iodo-2-*n*-pentyl-2*H*-indazole (34)

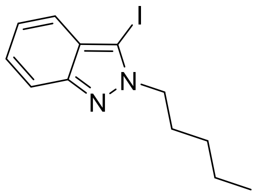

Following **General Procedure A** and/or **B**, wet flash column chromatography (EtOAc/hexane, 1:19) furnished the title compound **34** (R_f = 0.30) as a yellow crystalline solid: m.p. 58–60 °C; IR (ATR, cm^{-1}) ν_{max} 3056, 2955, 2930, 1623, 1456, 1352, 1036, 757, 741, 435; ^{1}H NMR (400 MHz, $CDCl_3$) δ 7.67 (1H, dd, J = 8.7, 0.6 Hz), 7.40 (1H, d, J = 8.4 Hz), 7.31 (1H, ddd, J = 8.7, 6.6, 1.0 Hz), 7.12 (1H, ddd, J = 8.4, 6.6, 0.7 Hz), 4.50 (2H, t, J = 7.4 Hz), 1.99 (2H, quint, J = 7.7 Hz), 1.43–1.31 (4H, m), 0.91 (3H, t, J = 6.8 Hz); ^{13}C NMR (100 MHz, $CDCl_3$) δ 149.4, 127.2, 126.8, 122.5, 120.6, 117.9, 75.1, 53.6, 30.3, 28.7, 22.3, 13.9; HRMS (ESI) m/z $[M+H]^+$ Calcd for $C_{12}H_{16}IN_2$ 315.0358, found 315.0357 (– 0.3 ppm).

^{1}H NMR (400 MHz, $CDCl_3$) **34**

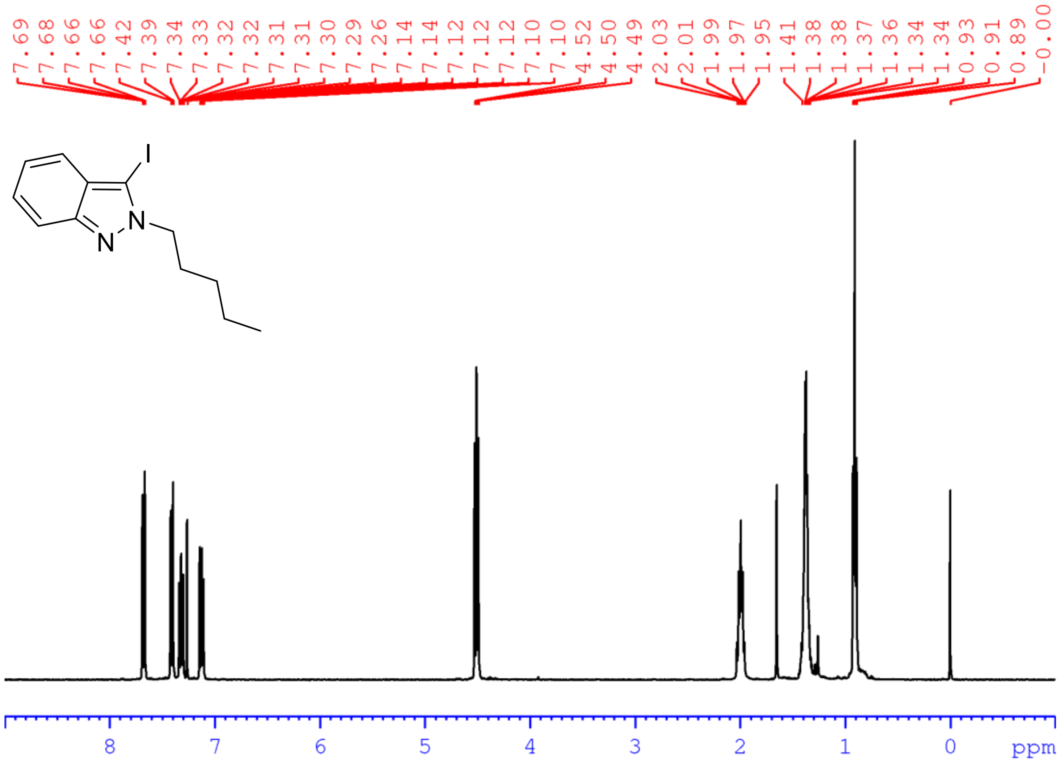

^{13}C NMR (100 MHz, $CDCl_3$) **34**

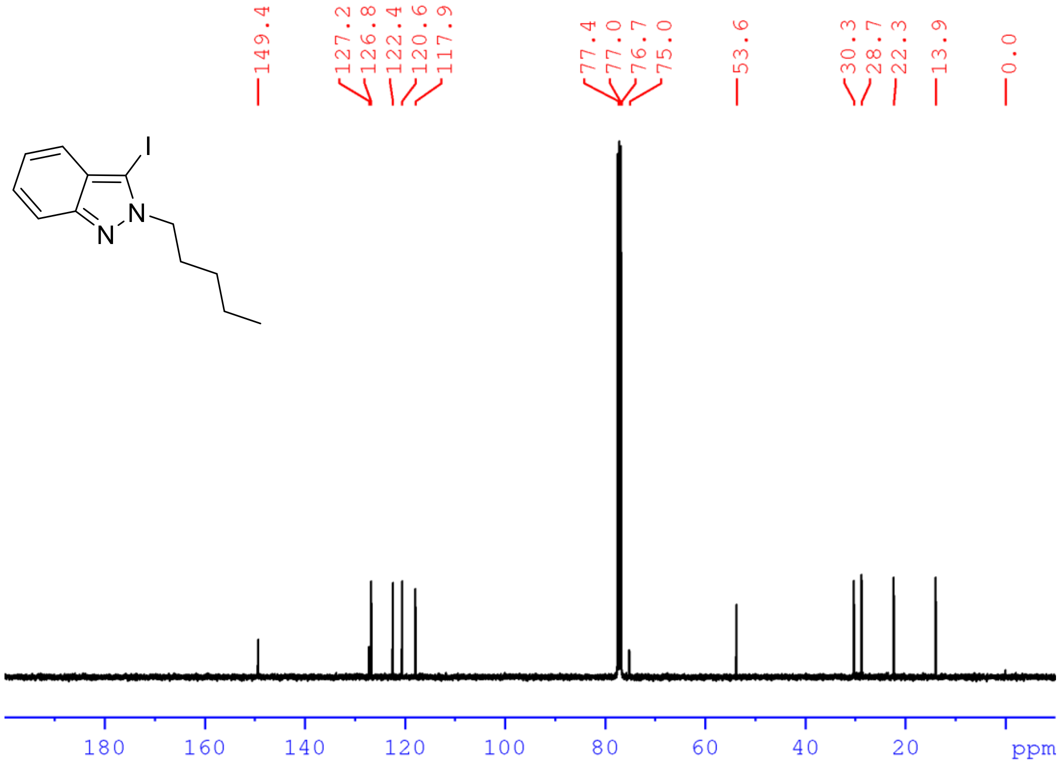

3-Bromo-1-*n*-pentyl-1*H*-indazole (35)

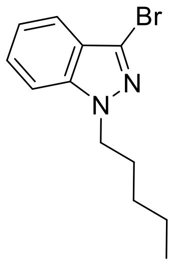

Following **General Procedure A** and/or **B**, wet flash column chromatography (Et_2O/hexane, 1:19) furnished the title compound **35** (R_f = 0.33) as a colorless oil: IR (ATR, cm^{-1}) ν_{max} 3061, 2956, 2930, 2871, 2859, 1616, 1494, 1462, 1328, 1175, 765, 739, 428; ^{1}H NMR (400 MHz, $CDCl_3$) δ 7.57 (1H, d, J = 8.2 Hz), 7.37 (1H, ddd, J = 8.6, 7.5, 1.0 Hz), 7.33 (1H, d, J = 8.5 Hz), 7.15 (1H, ddd, J = 7.9, 7.9, 1.2 Hz), 4.29 (2H, t, J = 7.2 Hz), 1.88 (2H, quint, J = 7.3 Hz), 1.36–1.21 (4H, m), 0.85 (3H, t, J = 7.0 Hz); ^{13}C NMR (100 MHz, $CDCl_3$) δ 140.4, 127.1, 123.4, 121.0, 120.1, 119.7, 109.1, 49.2, 29.4, 28.7, 22.1, 13.8; HRMS (ESI) *m/z* $[M+H]^+$ Calcd for $C_{12}H_{16}{}^{79}BrN_2$ 267.0497, found 267.0491 (– 2.2 ppm).

^{1}H NMR (400 MHz, $CDCl_3$) **35**

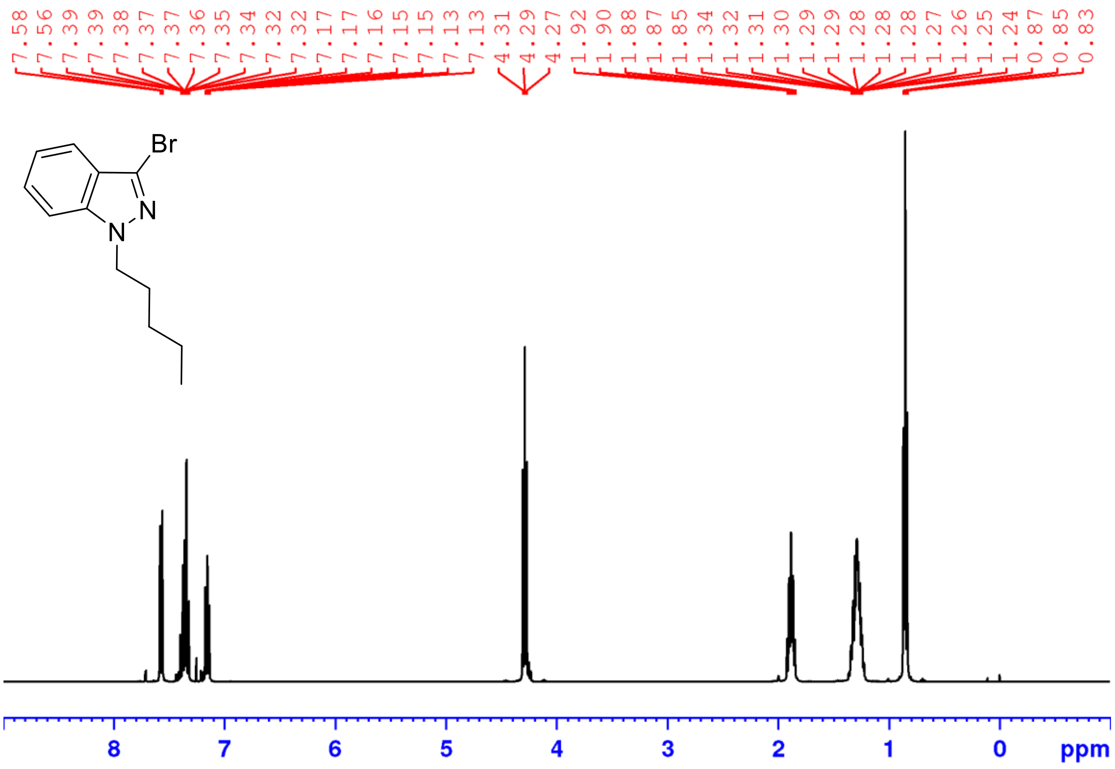

^{13}C NMR (100 MHz, $CDCl_3$) **35**

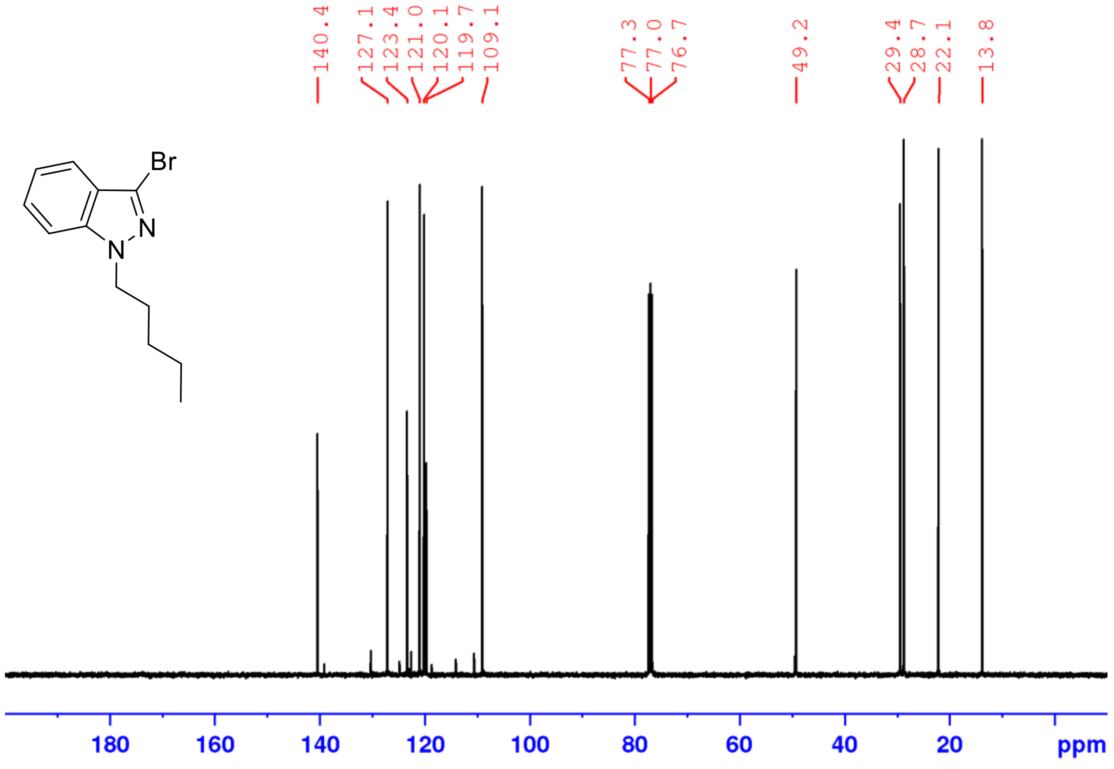

3-Bromo-2-*n*-pentyl-2*H*-indazole (36)

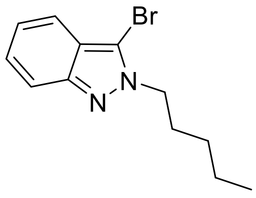

Following **General Procedure A** and/or **B**, wet flash column chromatography (Et_2O/hexane, 1:19) furnished the title compound **36** (R_f = 0.17) as a colorless oil: IR (ATR, cm^{-1}) ν_{max} 3059, 2955, 2930, 2871, 2860, 1627, 1461, 1360, 1044, 758, 741; ^{1}H NMR (400 MHz, $CDCl_3$) δ 7.67 (1H, d, *J* = 8.8 Hz, H4), 7.51 (1H, d, *J* = 8.4 Hz, H7), 7.30 (1H, ddd, *J* = 8.4, 6.6, 0.6 Hz, H6), 7.12 (1H, t, *J* = 7.5 Hz, H5), 4.47 (2H, t, *J* = 7.4 Hz, H1'), 1.98 (2H, quint, *J* = 7.3 Hz, H2'), 1.43–1.30 (4H, m, H3'/H4'), 0.90 (3H, t, *J* = 6.8 Hz, H5'); ^{13}C NMR (100 MHz, $CDCl_3$) δ 148.4, 126.7, 122.2, 121.8, 119.3, 117.8, 105.6, 51.7, 29.9, 28.7, 22.2, 13.9; HRMS (ESI) *m/z*: $[M+H]^+$ Calcd for $C_{12}H_{16}{}^{79}BrN_2$ 267.0497, found 267.0495 (− 0.7 ppm).

^{1}H NMR (400 MHz, $CDCl_3$) **36**

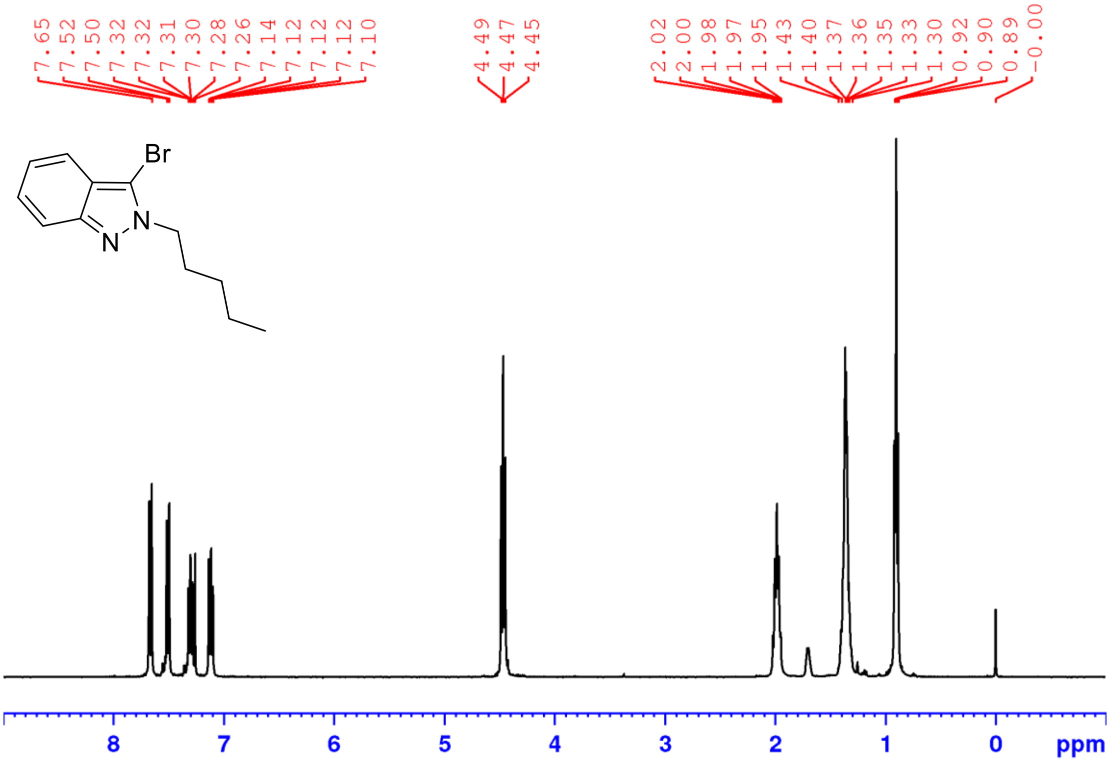

^{13}C NMR (100 MHz, $CDCl_3$) **36**

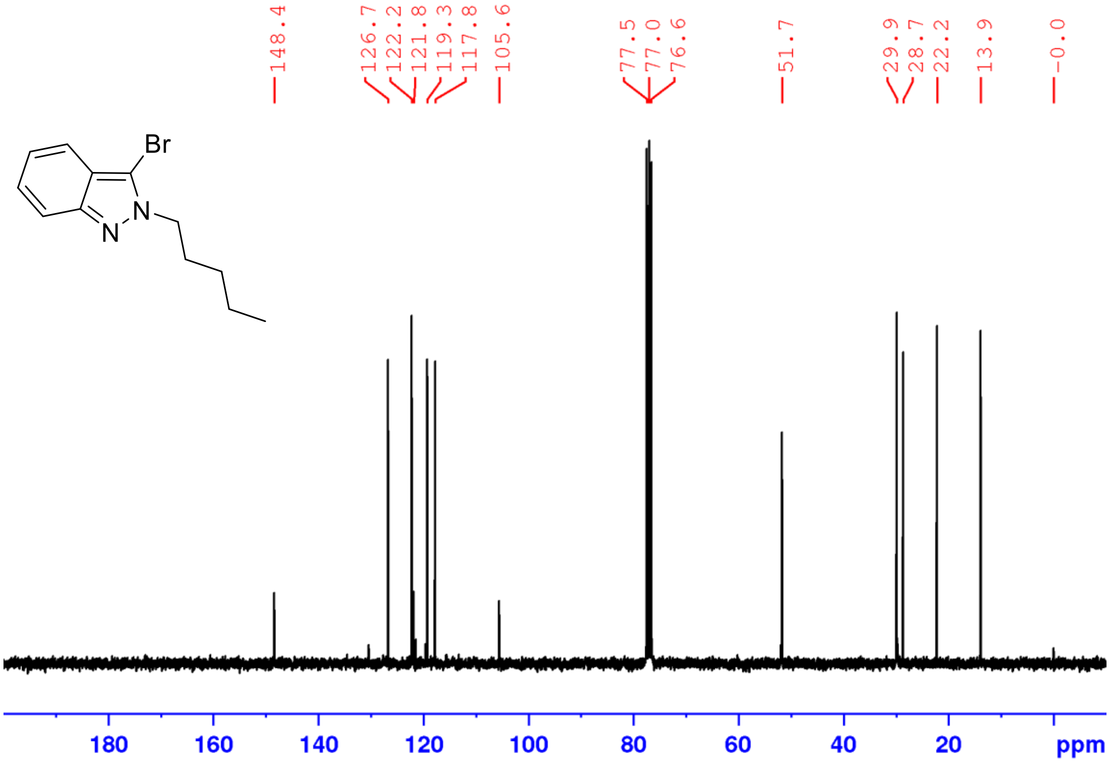

3-Chloro-1-*n*-pentyl-1*H*-indazole (37)

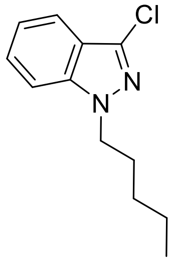

Following **General Procedure A** and/or **B**, wet flash column chromatography (EtOAc/hexane, 1:9) furnished the title compound **37** (R_f = 0.57) as an colorless oil; IR (ATR, cm^{-1}) ν_{max} 3062, 2957, 2931, 2872, 2860, 1617, 1466, 1337, 1178, 739, 428; ^{1}H NMR (400 MHz, $CDCl_3$) δ 7.64 (1H, dd, J = 8.2, 1.7 Hz), 7.41–7.33 (2H, m), 7.16 (1H, ddd, J = 7.9, 6.6, 1.1 Hz), 4.28 (2H, t, J 7.2 Hz), 1.89 (2H, quint, J = 7.3 Hz), 1.38–1.23 (4H, m), 0.86 (3H, t, J = 7.0 Hz); ^{13}C NMR (100 MHz, $CDCl_3$) δ 140.6, 132.2, 127.2, 120.9, 120.8, 119.6, 109.2, 49.1, 29.4, 28.8, 22.2, 13.8; HRMS (ESI) *m/z* $[M+H]^+$ Calcd for $C_{12}H_{16}{}^{35}ClN_2$ 223.0997, found 223.1000 (1.3 ppm).

^{1}H NMR (400 MHz, $CDCl_3$) **37**

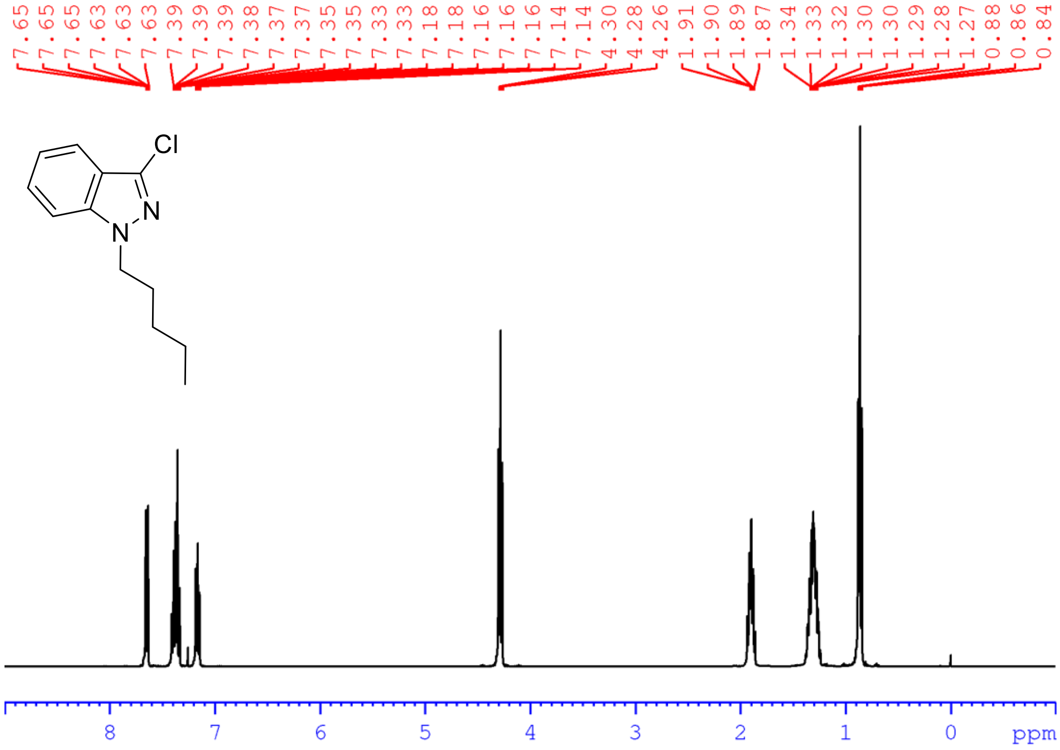

^{13}C NMR (100 MHz, $CDCl_3$) **37**

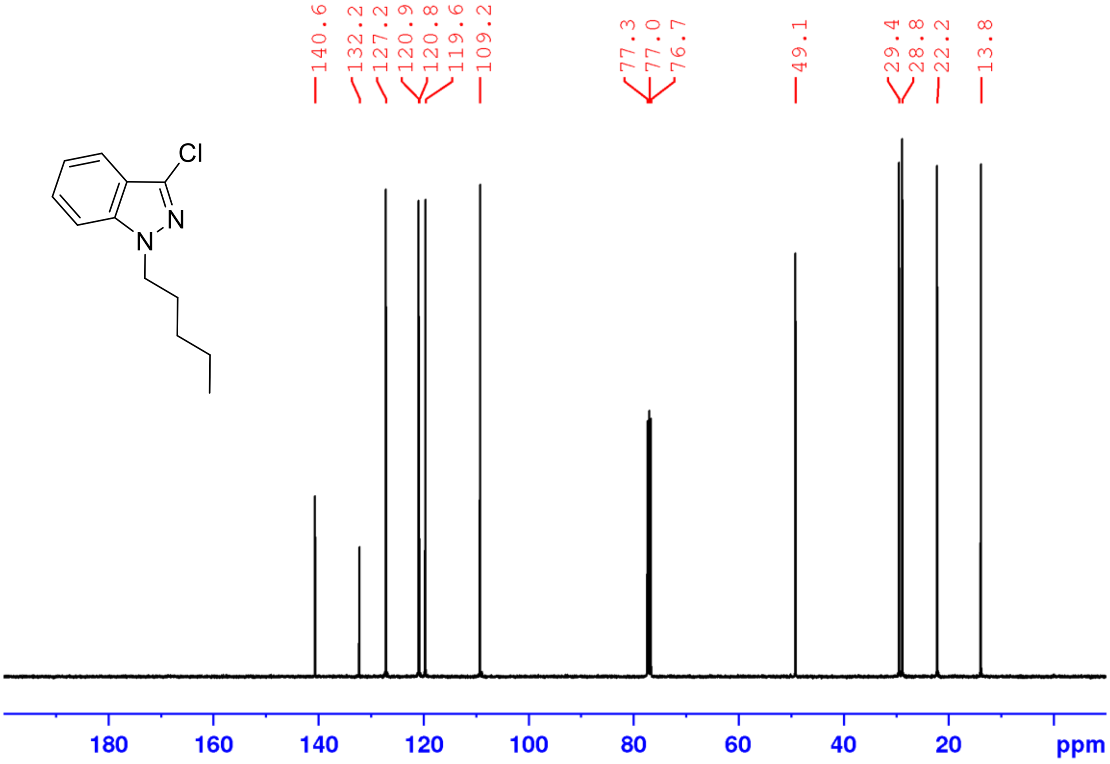

3-Chloro-2-*n*-pentyl-2*H*-indazole (38)

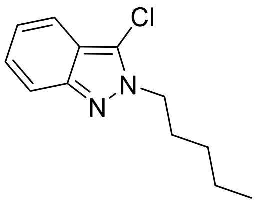

Following **General Procedure A** and/or **B**, wet flash column chromatography (EtOAc/hexane, 1:9) furnished the title compound **38** (R_f = 0.43) as an colorless oil: IR (ATR, cm^{-1}) v_{max} 3062, 2957, 2931, 2872, 2860, 1617, 1495, 1466, 1336, 1178, 1005, 766, 739, 428; ^{1}H NMR (400 MHz, $CDCl_3$) δ 7.28 (1H, d, J = 8.8 Hz), 7.55 (1H, d, J = 8.4 Hz), 7.30 (1H, ddd, J = 8.7, 6.6, 1.1 Hz), 7.11 (1H, ddd, J = 8.5, 6.7, 0.7 Hz), 4.43 (2H, t, J = 7.2 Hz), 1.98 (2H, quint, J = 7.3 Hz), 1.42–1.28 (4H, m), 0.90 (3H, t, J = 6.9 Hz);^{13}C NMR (100 MHz, $CDCl_3$) δ 147.8, 126.7, 122.0, 119.0, 118.9, 118.6, 117.8, 50.5, 29.7, 28.7, 22.2, 13.9; HRMS (ESI) *m/z*: [M+H]$^+$ Calcd for $C_{12}N_{16}{}^{35}ClN_2$ 223.0997, found 223.0996 (– 0.4 ppm).

^{1}H NMR (400 MHz, $CDCl_3$) **38**

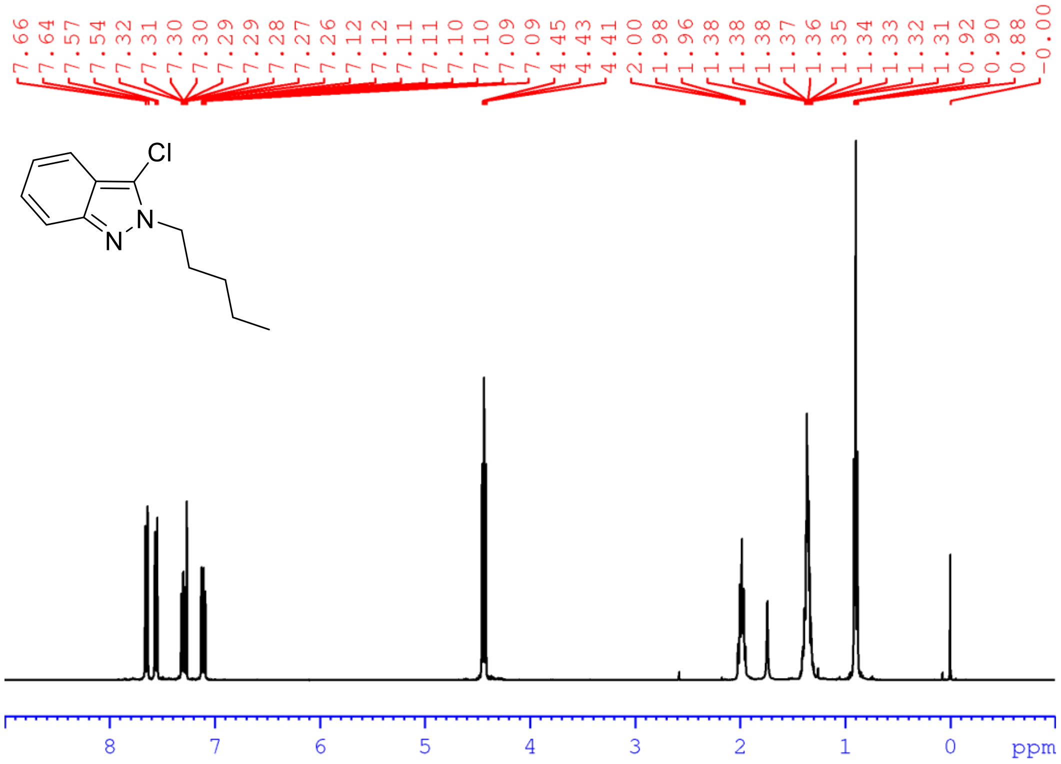

^{13}C NMR (100 MHz, $CDCl_3$) **38**

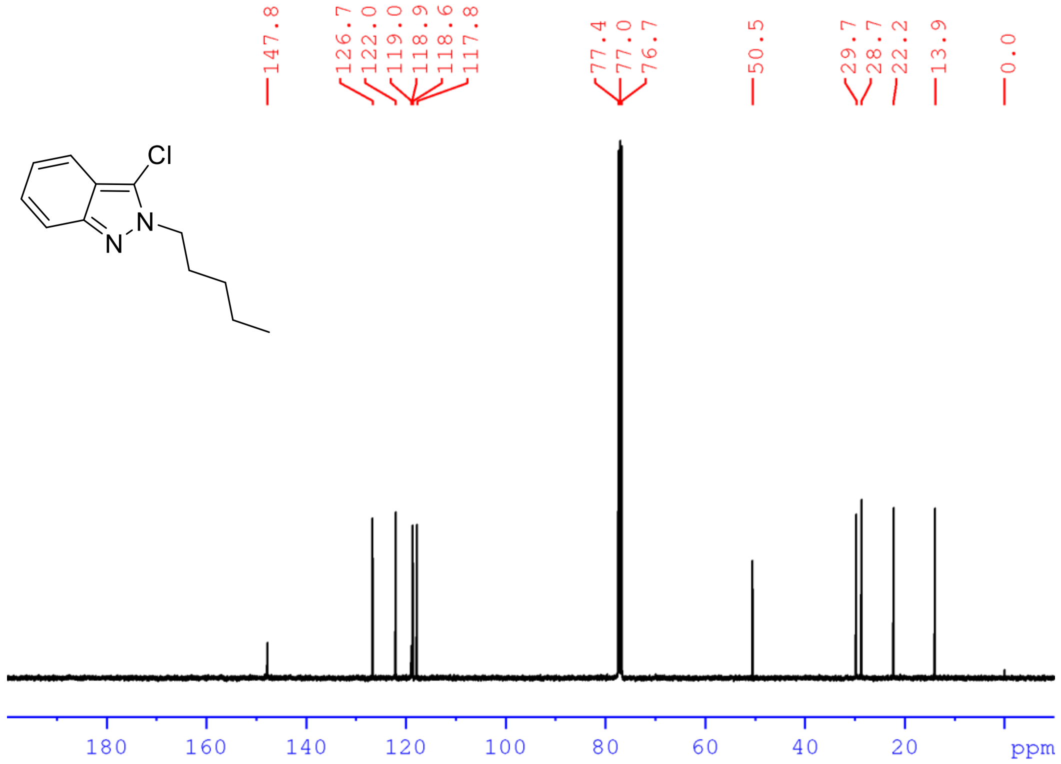

3-Nitro-1-*n*-pentyl-1*H*-indazole (39)

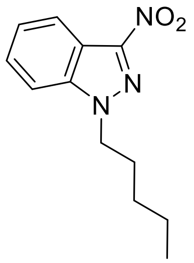

Following **General Procedure A** and/or **B**, wet flash column chromatography (EtOAc/hexane, 1:19) gave the title compound **39** (R_f = 0.20) as an amber oil: IR (ATR, cm^{-1}) ν_{max} 3061, 2957, 2931, 2861, 1525, 1497, 1457, 1390, 1316, 1188, 831, 748, 431; ^1H NMR (400 MHz, $CDCl_3$) δ 8.29 (1H, dd, J = 8.2, 1.0 Hz), 7.58–7.53 (2H, m), 7.51–7.44 (1H, m), 4.49 (2H, t, J = 7.4 Hz), 2.01 (2H, quint, J = 7.3 Hz), 1.42–1.29 (4H, m), 0.89 (3H, t, J = 6.9 Hz); ^{13}C NMR (100 MHz, $CDCl_3$) δ 147.3, 141.2, 128.2, 125.5, 121.5, 116.8, 110.2, 50.6, 29.4, 28.8, 22.2, 13.9; HRMS (ESI) *m/z* $[M+H]^+$ Calcd for $C_{12}H_{16}N_3O_2$ 234.1237, found 234.1236 (–0.4 ppm).

^{1}H NMR (400 MHz, $CDCl_3$) **39**

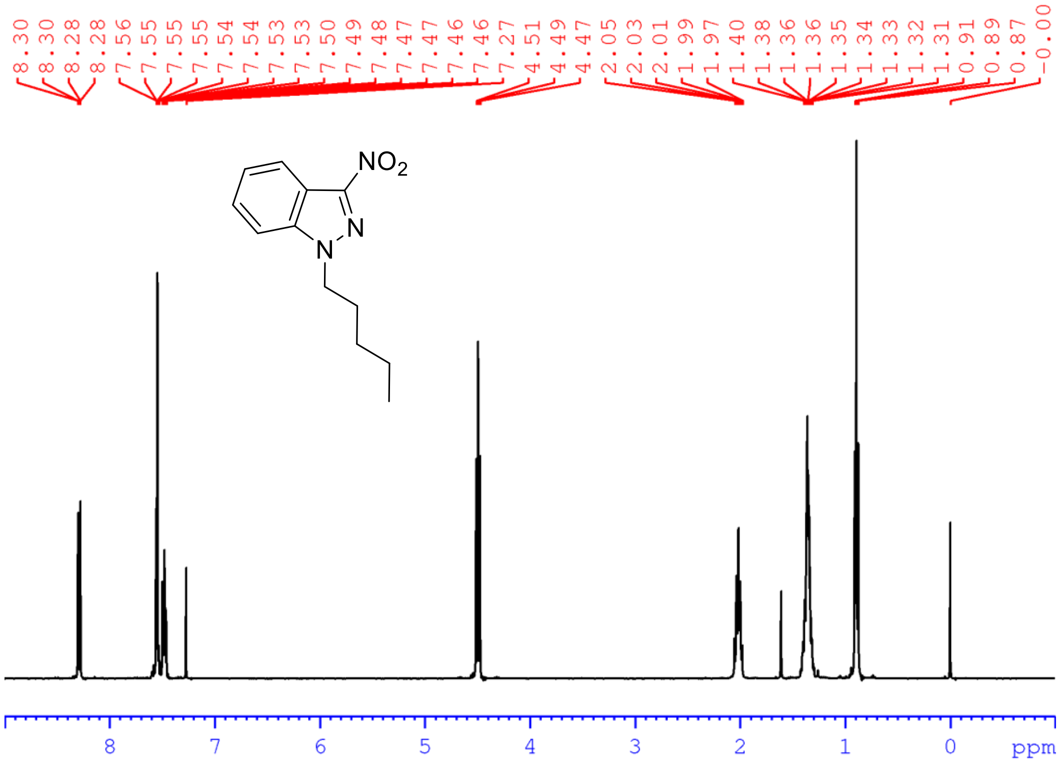

^{13}C NMR (100 MHz, $CDCl_3$) **39**

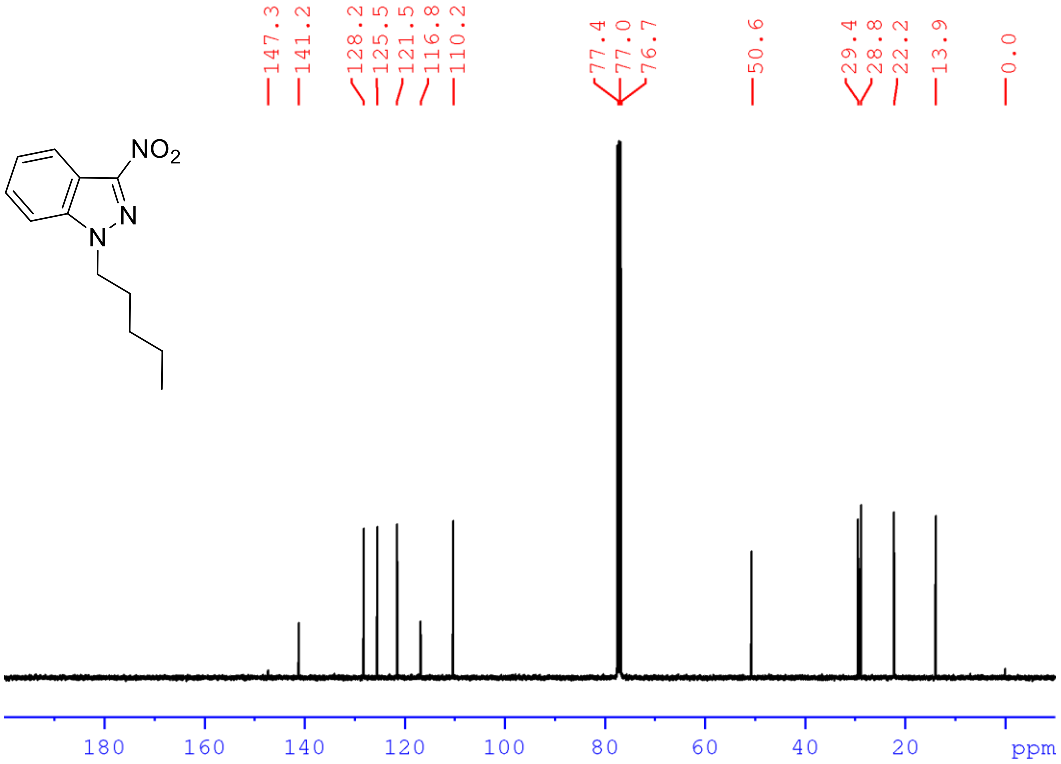

3-Nitro-2-*n*-pentyl-2*H*-indazole (40)

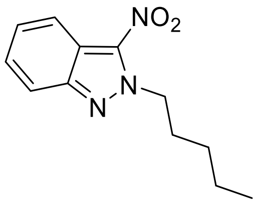

Following **General Procedure B**, wet flash column chromatography (EtOAc/hexane, 1:19) furnished the title compound **40** (R_f = 0.30) as a yellow oil: IR (ATR, cm^{-1}) v_{max} 3068, 2958, 2930, 2861, 1494, 1440, 1336, 1287, 1088, 822, 751, 433; ^{1}H NMR (400 MHz, $CDCl_3$) δ 8.17 (1H, ddd, J = 8.3, 1.8, 0.9 Hz), 7.82 (1H, app. d, J = 8.4 Hz), 7.51–7.43 (2H, m), 4.94 (2H, t, J = 7.5 Hz), 2.01 (2H, quint, J = 7.5 Hz), 1.42–1.37 (4H, m), 0.91 (3H, t, J = 7.1 Hz); ^{13}C NMR (100 MHz, $CDCl_3$) δ 146.2, 137.2, 129.5, 128.5, 120.4, 119.0, 118.2, 55.4, 30.0, 28.6, 22.2, 13.9; HRMS (ESI) *m/z*: $[M+H]^+$ Calcd for $C_{12}H_{16}N_3O_2$ 234.1237, found 234.1238 (0.4 ppm).

^{1}H NMR (400 MHz, $CDCl_3$) **40**

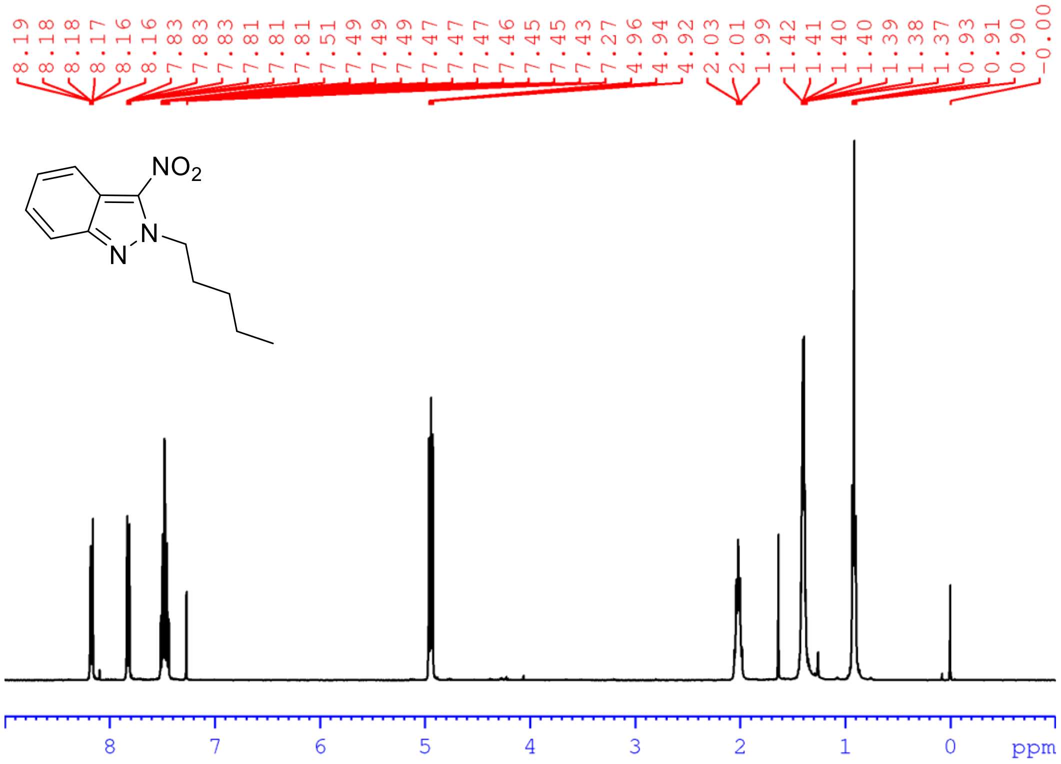

^{13}C NMR (100 MHz, $CDCl_3$) **40**

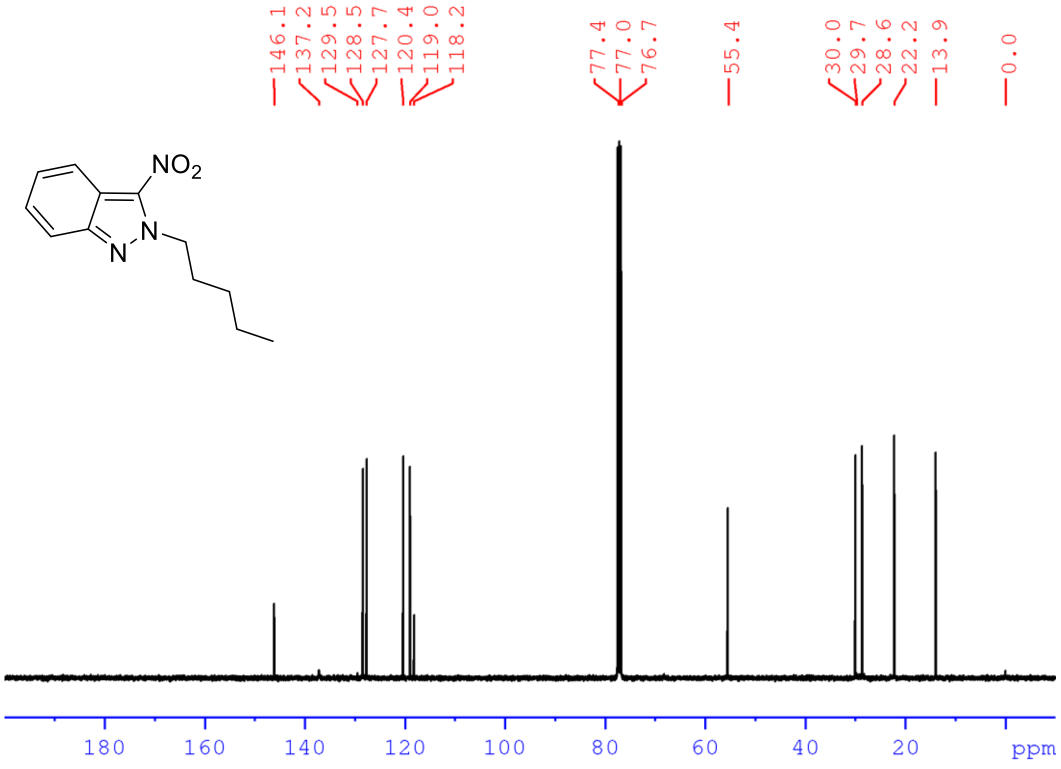

1-*n*-Pentyl-1*H*-indazole-3-carbonitrile (41)

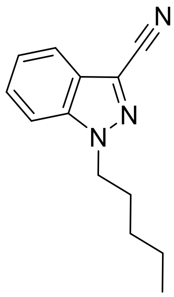

Following **General Procedure A** and/or **B**, preparative thin layer chromatography (EtOAc/hexane, 1:4) gave the title compound **41** (R_f = 0.65) as a colorless oil: IR (ATR, cm^{-1}) ν_{max} 2957, 2932, 2861, 2233, 1467, 1350, 770, 744, 429; ^1H NMR (300 MHz, $CDCl_3$) δ 7.85 (1H, d, J = 8.2 Hz), 7.53–7.48 (2H, m), 7.35 (1H, ddd, J = 7.9, 6.4, 1.3 Hz), 4.45 (2H, t, J = 7.2 Hz), 1.97 (2H, quint, J = 7.2 Hz), 1.39–1.26 (4H, m), 0.89 (3H, t, J = 7.1 Hz); ^{13}C NMR (100 MHz, $CDCl_3$) δ 139.5, 127.6, 125.4, 123.5, 119.7, 117.4, 113.8, 110.1, 50.2, 29.3, 28.8, 22.2, 13.9; HRMS (ESI) *m/z*: $[M+H]^+$ Calcd for $C_{13}H_{16}N_3$ 214.1339; found 214.1337 (– 0.9 ppm).

^{1}H NMR (300 MHz, $CDCl_3$) **41**

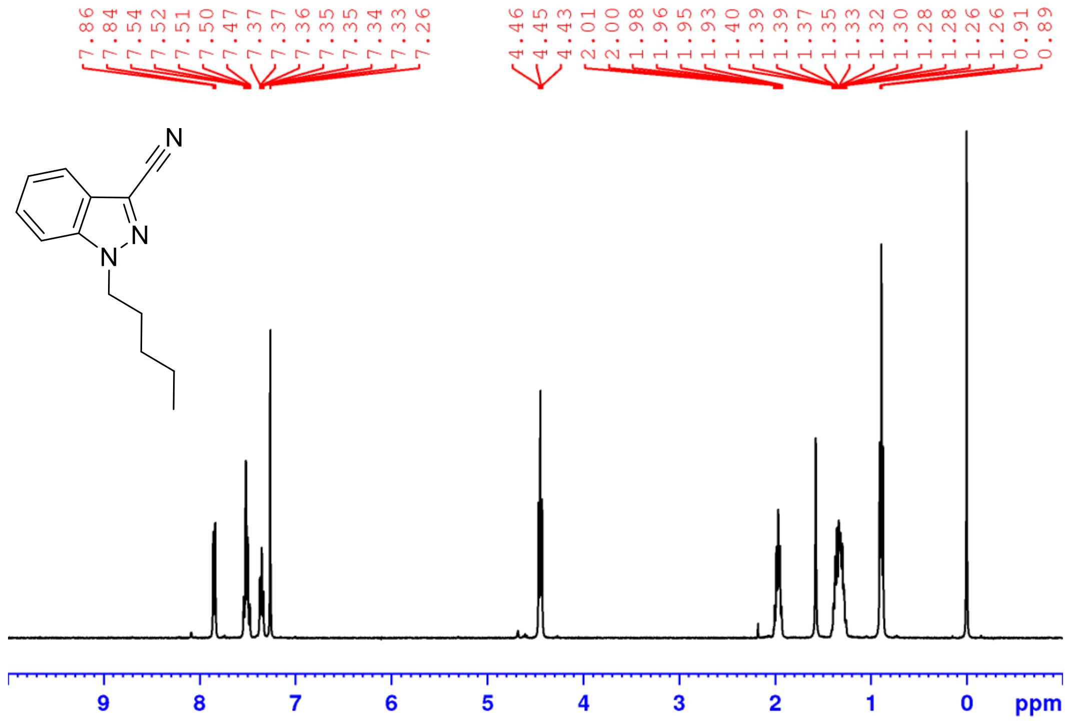

^{13}C NMR (100 MHz, $CDCl_3$) **41**

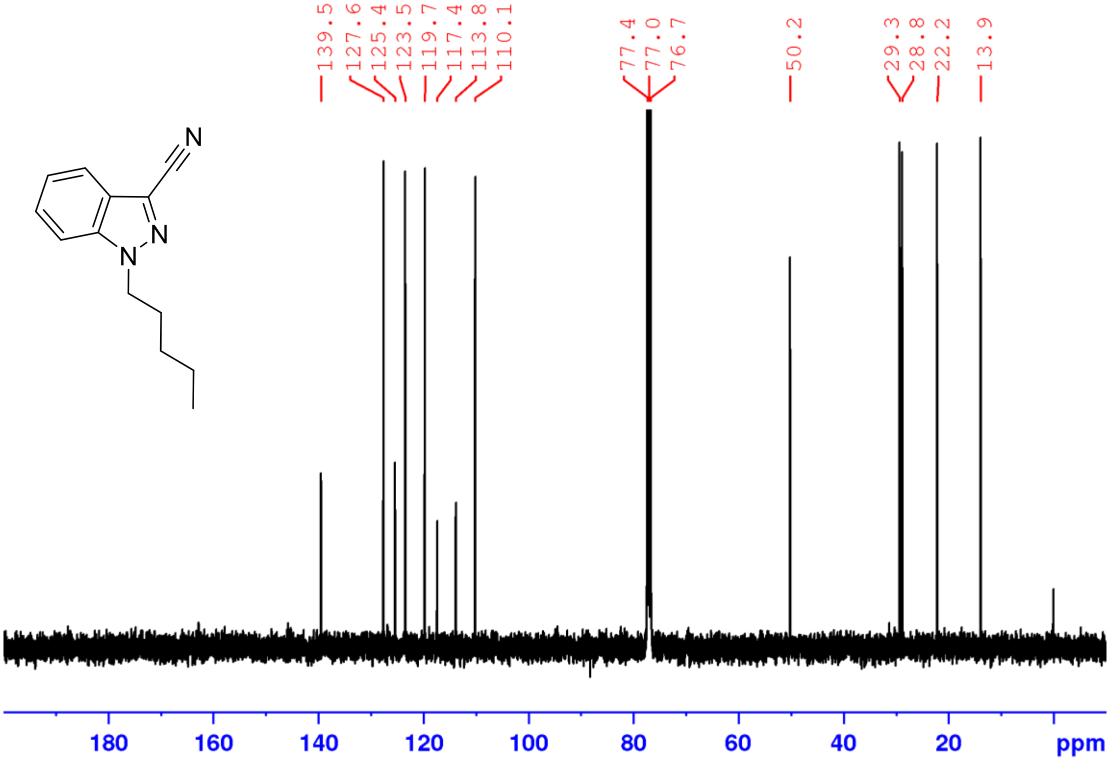

1-*n*-Pentyl-1*H*-indazole-3-carboxaldehyde (43)

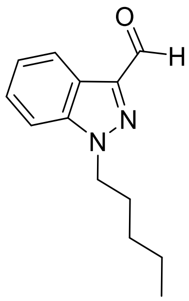

Following **General Procedure A** and/or **B**, wet flash column chromatography (EtOAc/hexane, 1:4) gave the title compound **43** (R_f = 0.83) as an amber oil: IR (ATR, cm^{-1}) ν_{max} 2956, 2932, 2859, 1679, 1472, 1139, 1092, 787, 744, 514, 432; ^{1}H NMR (300 MHz, $CDCl_3$) δ 10.24 (1H, s), 8.30 (1H, ddd, J = 8.1, 1.0, 1.0 Hz), 7.48–7.45 (2H, m,), 7.34 (1H, ddd, J = 8.0, 5.4, 2.5 Hz), 4.46 (2H, t, J = 7.2 Hz), 1.99 (2H, quint, J = 7.3 Hz), 1.44–1.26 (4H, m), 0.89 (3H, t, J = 6.9 Hz); ^{13}C NMR (75 MHz, $CDCl_3$) δ 186.8, 142.8, 140.8, 127.2, 123.9, 122.2, 122.0, 109.5, 49.9, 29.2, 28.8, 22.1, 13.8; HRMS (ESI) *m/z*: $[M+H]^+$ Calcd for $C_{13}H_{17}N_2O$ 217.1335, found 217.1340 (2.3 ppm).

^{1}H NMR (300 MHz, $CDCl_3$) **43**

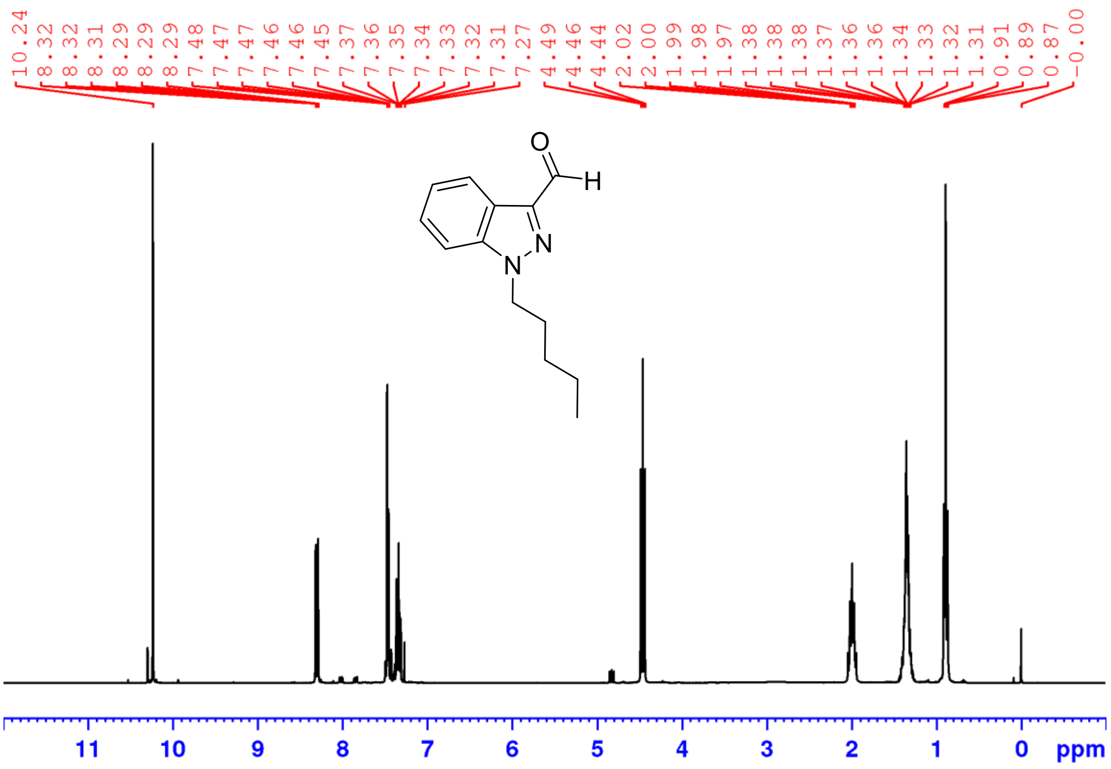

^{13}C NMR (75 MHz, $CDCl_3$) **43**

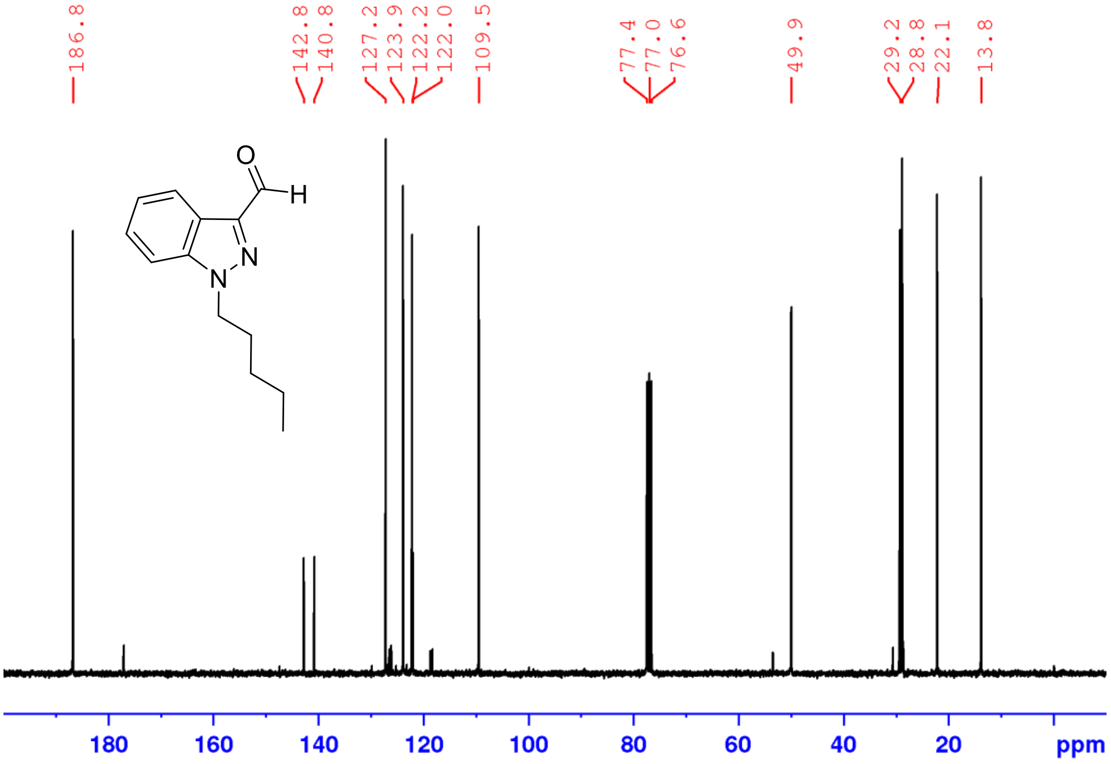

1-(1-*n*-Pentyl-1*H*-indazol-3-yl)ethan-1-one (45)

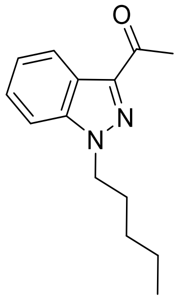

Following **General Procedure A** and/or **B**, wet flash column chromatography (EtOAc/hexane, 1:4) gave the title compound **45** (R_f = 0.55) as a colorless oil: IR (ATR, cm^{-1}) ν_{max} 3057, 2956, 2932, 2872, 2860, 1670, 1470, 1172, 1155, 939, 746, 569, 432; ^1H NMR (300 MHz, $CDCl_3$) δ 8.36 (1H, ddd, *J* = 8.1, 1.0, 1.0 Hz), 7.46–7.40 (2H, m), 7.30 (1H, ddd, *J* = 8.0, 5.9, 2.1 Hz), 4.41 (2H, t, *J* = 7.2 Hz), 2.71 (3H, s), 1.97 (2H, quint, *J* = 7.3 Hz), 1.44–1.27 (4H, m), 0.89 (3H, t, *J* = 6.9 Hz); ^{13}C NMR (75 MHz, $CDCl_3$) δ 194.7, 142.1, 140.7, 126.6, 123.4, 122.9, 122.7, 109.2, 49.6, 29.3, 28.8, 26.7, 22.2, 13.8; HRMS (ESI) *m/z*: $[M+H]^+$ Calcd for $C_{14}H_{19}N_2O$ 231.1492, found 231.1492 (0.0 ppm).

^{1}H NMR (300 MHz, $CDCl_3$) **45**

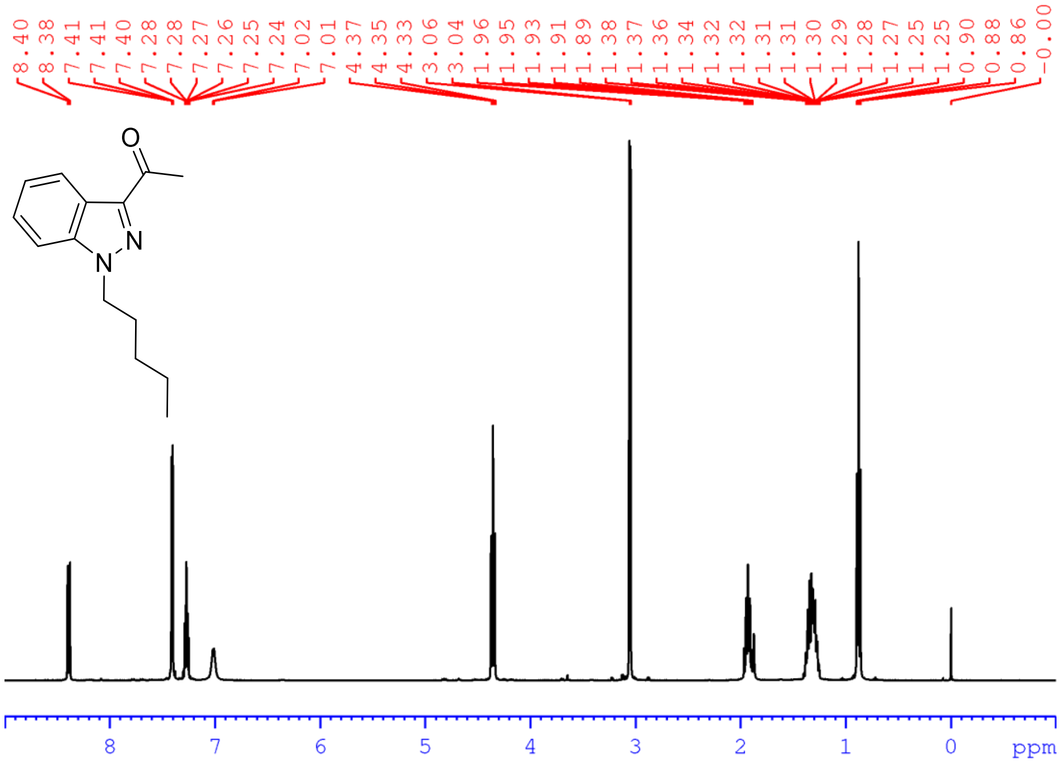

^{13}C NMR (75 MHz, $CDCl_3$) **45**

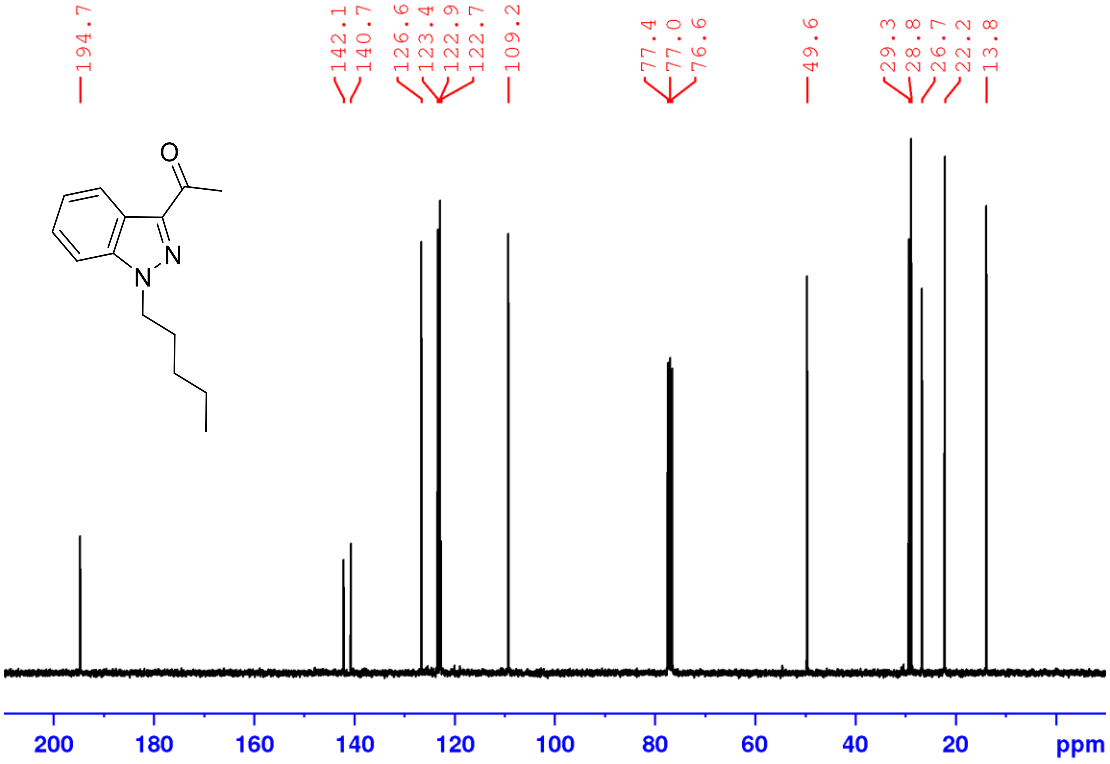

***N*-Methyl-1-*n*-pentyl-1*H*-indazole-3-carboxamide (47)**

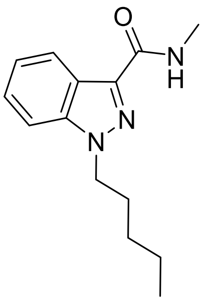

Following **General Procedure A** and/or **B**, the title compound **47** was isolated upon work-up as an amber oil: IR (ATR, cm^{-1}) ν_{max} 3333, 2955, 2931, 2871, 1651, 1539, 1182, 772, 749, 547; ^{1}H NMR (400 MHz, $CDCl_3$) δ 8.39 (1H, d, J = 8.2 Hz), 7.41–7.40 (2H, m), 7.30–7.23 (1H, m), 7.02, (1H, d, J = 3.2 Hz), 4.35 (2H, t, J = 7.2 Hz), 3.05 (3H, d, J = 5.0 Hz), 1.93 (2H, quint, J = 7.3 Hz), 1.40–1.25 (4H, m), 0.88 (3H, t, J = 7.0 Hz); ^{13}C NMR (100 MHz, $CDCl_3$) δ 163.3, 140.7, 137.2, 126.6, 122.9, 122.7, 122.4, 109.1, 49.3, 29.4, 28.8, 25.6, 22.2, 13.9; HRMS (ESI) *m/z*: $[M+H]^+$ Calcd for $C_{14}H_{20}N_3O$ 246.1601, found 246.1599 (– 0.8 ppm).

^{1}H NMR (400 MHz, $CDCl_3$) **47**

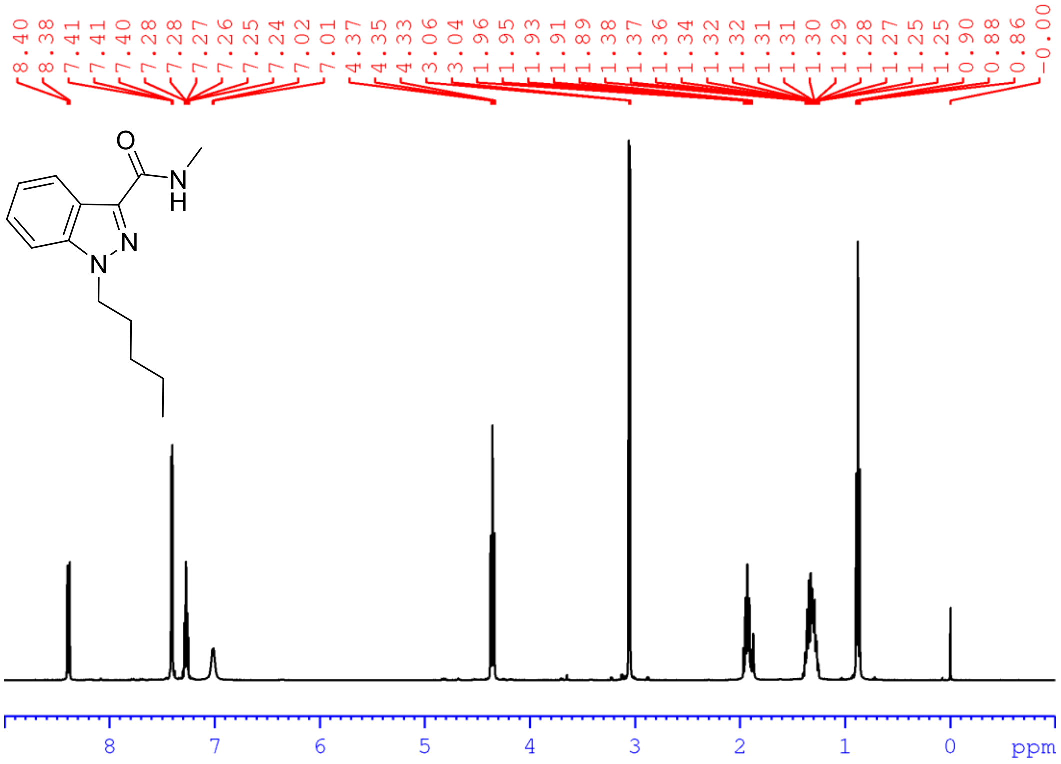

^{13}C NMR (100 MHz, $CDCl_3$) **47**

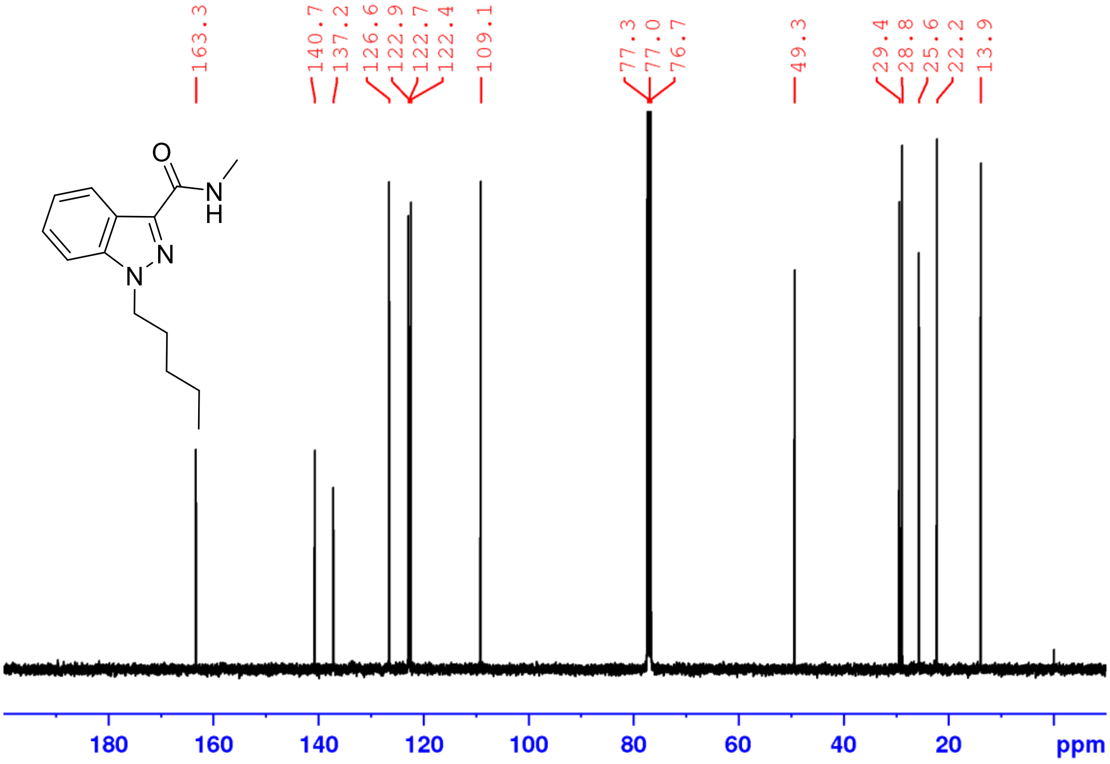

(1-*n*-Pentyl-1*H*-indazol-3-yl)(pyrrolidin-1-yl)methanone (49)

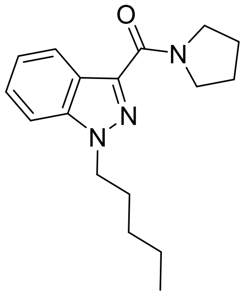

Following **General Procedure A** and/or **B**, wet flash column chromatography (EtOAc/hexane, 3:7) gave the title compound **49** (R_f = 0.30) as a colorless oil: IR (ATR, cm^{-1}) ν_{max} 3055, 2955, 2931, 2871, 1610, 1479, 1443, 1345, 1183, 1169, 748, 432; ^{1}H NMR (300 MHz, $CDCl_3$) δ 8.36 (1H, ddd, J = 8.2, 1.0, 1.0 Hz), 7.41–7.37 (2H, m), 7.25–7.20 (1H, m), 4.37 (2H, t, J = 7.0 Hz), 4.03 (2H, t, J = 6.6 Hz), 3.75 (2H, t, J = 6.6 Hz), 2.03–1.87 (6H, m), 1.41–1.22 (4H, m), 0.87 (3H, t, J = 7.0 Hz); ^{13}C NMR (75 MHz, $CDCl_3$) δ 162.3, 139.8, 138.6, 126.2, 124.3, 123.1, 121.8, 108.7, 49.0, 48.8, 46.6, 29.2, 28.7, 26.5, 23.8, 22.1, 13.8; HRMS (ESI) *m/z*: $[M+H]^+$ Calcd for $C_{17}H_{24}N_3O$ 286.1914, found 286.1910 (– 1.4 ppm).

^{1}H NMR (300 MHz, $CDCl_3$) **49**

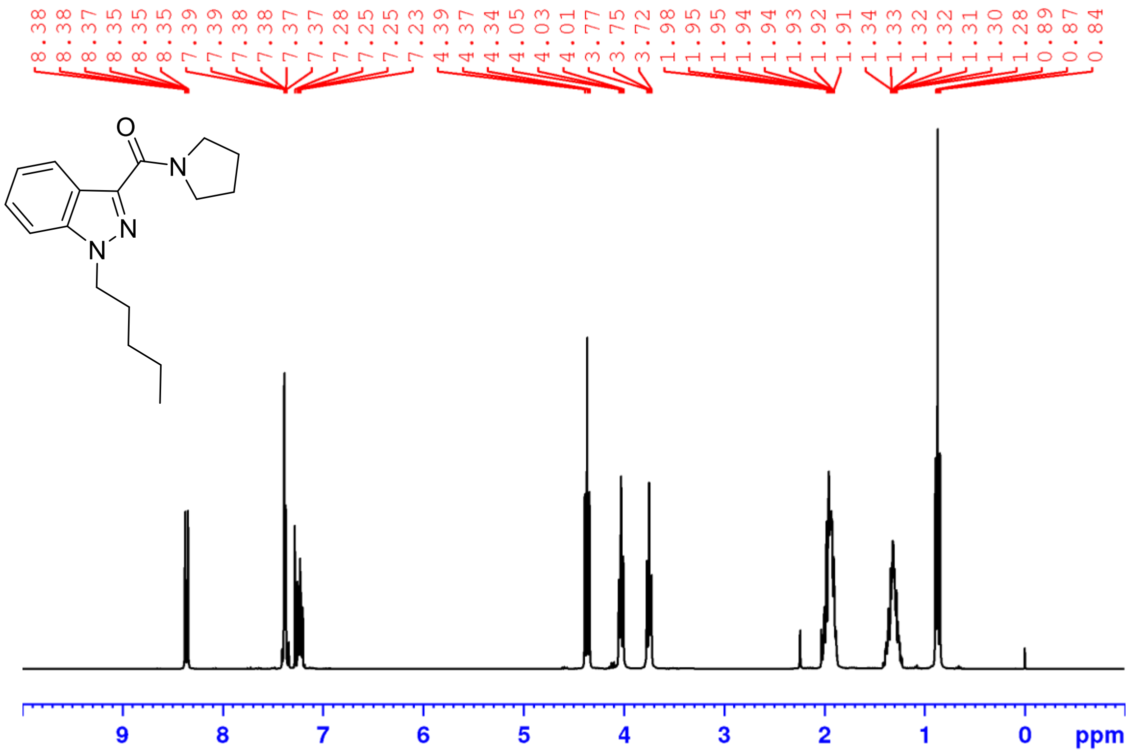

^{13}C NMR (75 MHz, $CDCl_3$) **49**

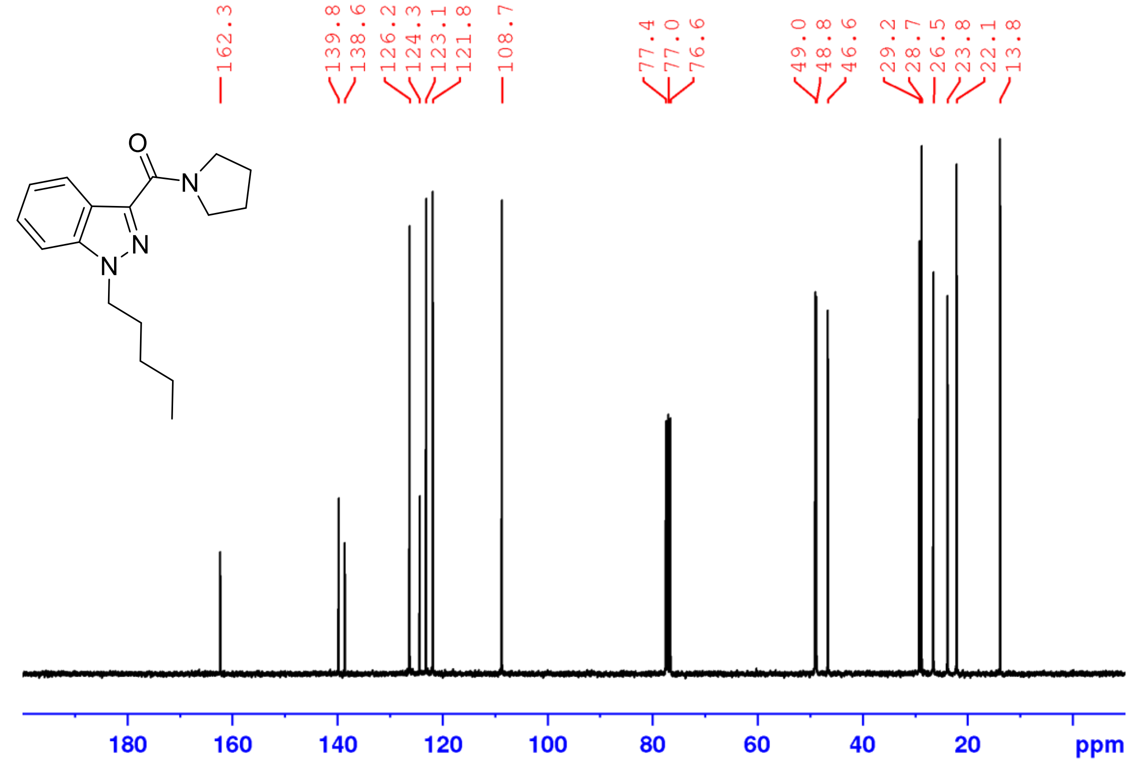

7-Methyl-1-*n*-pentyl-1*H*-indazole (51)

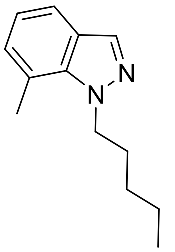

Following **General Procedure A** and/or **B**, wet flash column chromatography (Et_2O/hexane, 1:4) gave the title compound **51** (R_f = 0.40) as a colorless oil: IR (ATR, cm^{-1}) ν_{max} 3059, 3033, 2955, 2930, 2861, 1607, 1465, 1409, 1053, 859, 832, 773, 743, 637; ^1H NMR (300 MHz, $CDCl_3$) δ 7.94 (1H, s), 7.53 (1H, dd, *J* = 7.9, 0.5 Hz), 7.07 (1H, ddd, *J* = 6.9, 1.0, 1.0 Hz), 6.99 (1H, dd, *J* = 7.9, 7.0 Hz), 4.55 (1H, t, *J* = 7.5 Hz), 2.70 (3H, s), 1.89 (2H, quint, *J* = 7.4 Hz), 1.42–1.26 (4H, m), 0.89 (3H, t, *J* = 7.0 Hz); ^{13}C NMR (75 MHz, $CDCl_3$) δ 138.7, 132.9, 128.1, 124.8, 120.5, 119.8, 118.9, 51.3, 31.5, 28.8, 22.3, 19.4, 13.9; HRMS (ESI) *m/z*: $[M+H]^+$ Calcd for $C_{13}H_{19}N_2$ 203.1543, found 203.1541 (– 1.0 ppm).

^{1}H NMR (300 MHz, $CDCl_3$) **51**

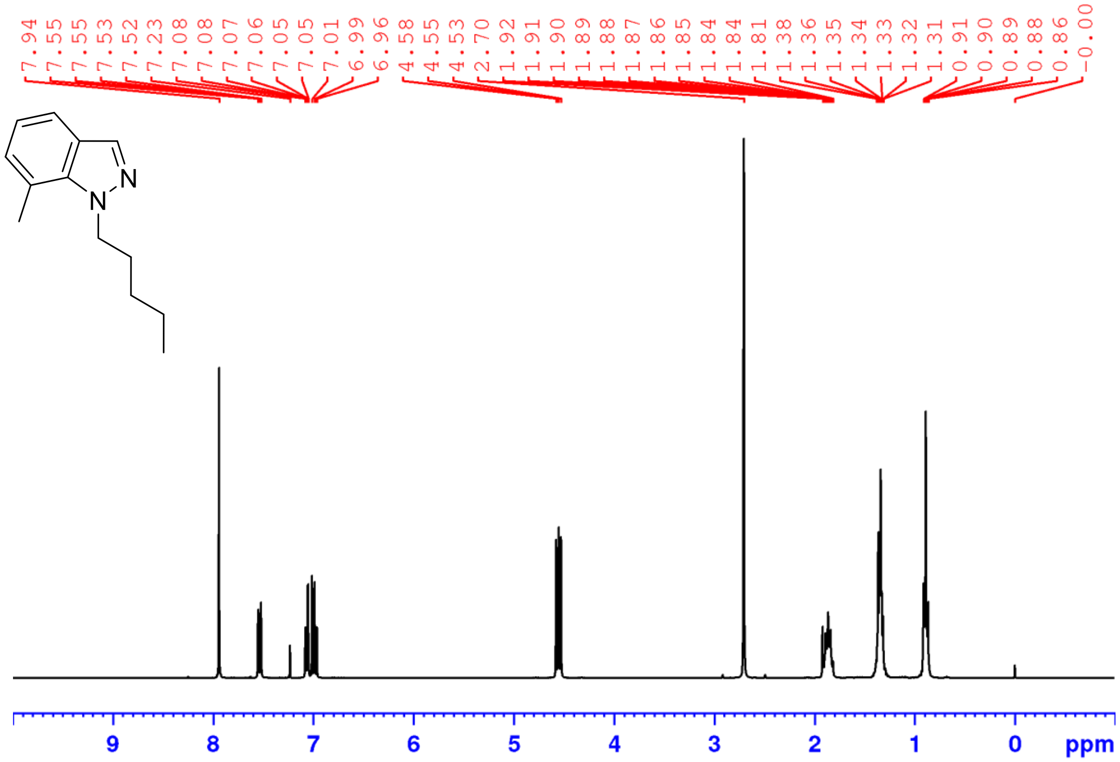

^{13}C NMR (75 MHz, $CDCl_3$) **51**

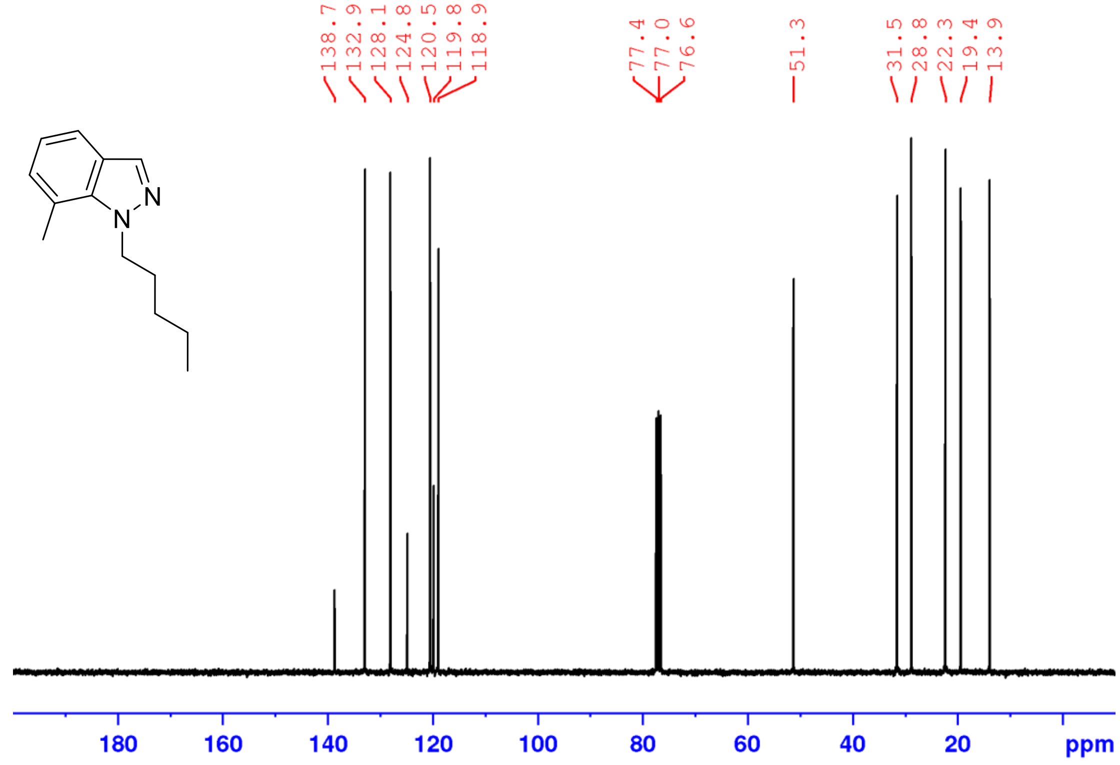

7-Methyl-2-*n*-pentyl-2*H*-indazole (52)

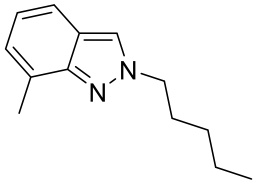

Following **General Procedure A** and/or **B**, wet flash column chromatography (Et_2O /hexane, 1:4) gave the title compound **52** (R_f = 0.27) as a colorless oil: IR (ATR, cm^{-1}) ν_{max} 2955, 2930, 2871, 2859, 1531, 1467, 1152, 1001, 873, 793, 750; ^1H NMR (300 MHz, $CDCl_3$) δ 7.86 (1H, s), 7.49–7.45 (1H, m), 7.14–6.94 (2H, m), 4.40 (2H, t, J = 7.3 Hz), 2.63 (3H, s), 2.00 (2H, quint, J = 7.4 Hz), 1.43–1.25 (4H, m), 0.89 (3H, t, J = 6.9 Hz); ^{13}C NMR (75 MHz, $CDCl_3$) δ 149.0, 127.4, 124.7, 122.5, 121.7, 121.5, 117.4, 53.7, 30.5, 28.8, 22.2, 17.2, 13.8; HRMS (ESI) *m/z*: $[M+H]^+$ Calcd for $C_{13}H_{19}N_2$ 203.1543, found 203.1542 (– 0.5 ppm).

^{1}H NMR (300 MHz, $CDCl_3$) **52**

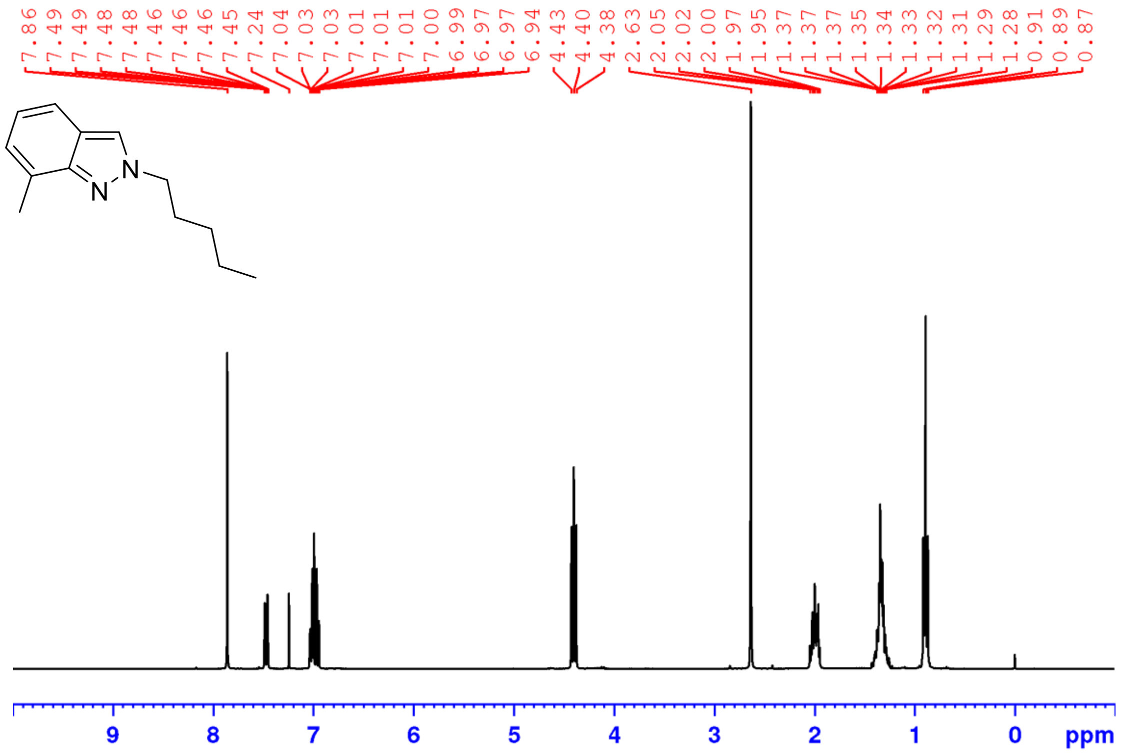

^{13}C NMR (75 MHz, $CDCl_3$) **52**

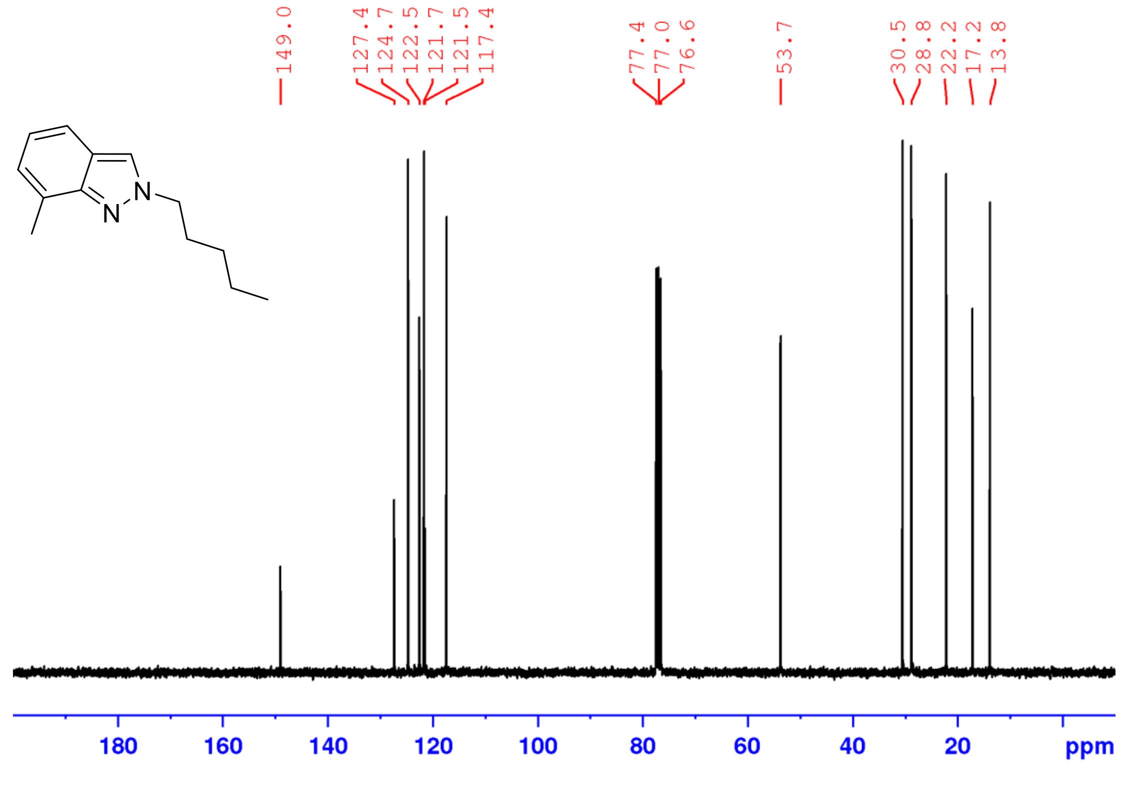

7-Bromo-1-*n*-pentyl-1*H*-indazole (53)

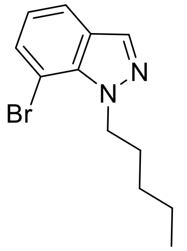

Following **General Procedure A** and/or **B**, wet flash column chromatography (Et_2O/hexane, 1:4) gave the title compound **53** (R_f = 0.50) as a colorless oil: IR (ATR, cm^{-1}) ν_{max} 3063, 2955, 2930, 2859, 1608, 1556, 1493, 1447, 1313, 1109, 938, 820, 772, 731, 567; ^1H NMR (300 MHz, $CDCl_3$) δ 7.97 (1H, s), 7.64 (1H, dd, J = 8.0, 0.9 Hz), 7.52 (1H, dd, J = 7.4, 0.9 Hz), 6.94 (1H, dd, J = 8.0, 7.5 Hz), 4.77 (2H, t, J = 7.5 Hz), 1.91 (2H, quint, J = 7.4 Hz), 1.43–1.28 (4H, m), 0.89 (3H, t, J = 6.9 Hz); ^{13}C NMR (75 MHz, $CDCl_3$) δ 136.5, 132.8, 130.9, 126.7, 121.4, 120.4, 102.8, 50.8, 31.3, 28.7, 22.3, 13.9; HRMS (ESI) *m/z*: $[M+H]^+$ Calcd for $C_{12}H_{16}{}^{79}BrN_2$ 267.0491, found 267.0486 (–1.9 ppm).

^{1}H NMR (300 MHz, $CDCl_3$) **53**

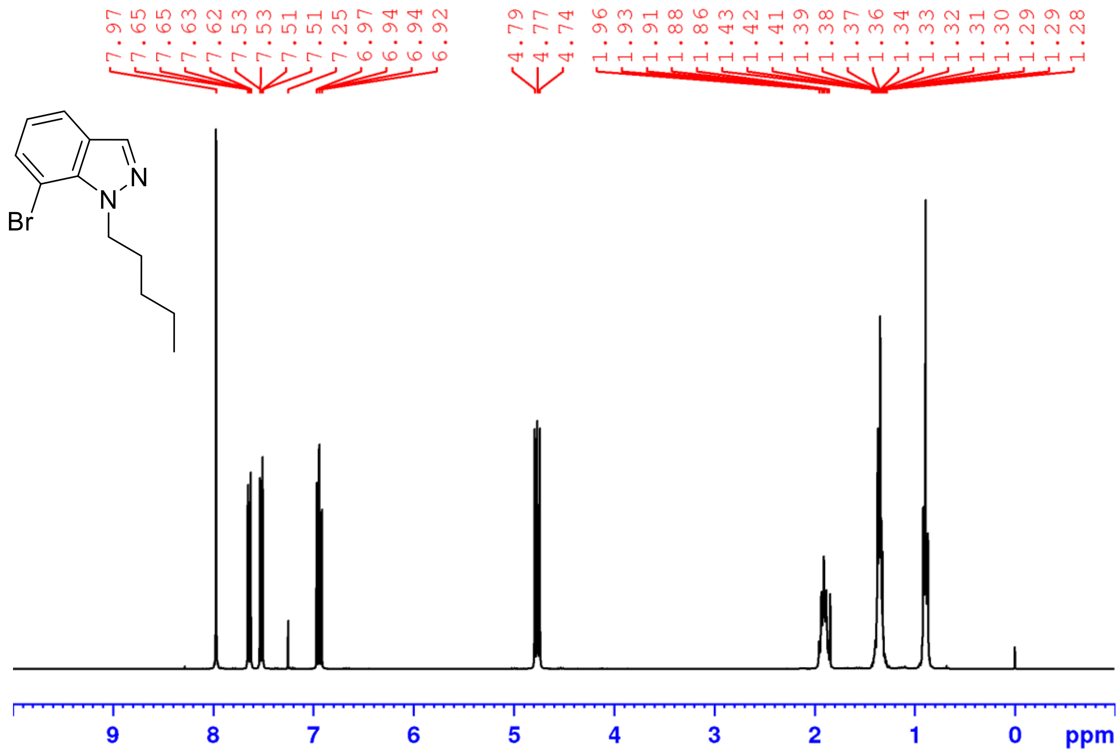

^{13}C NMR (75 MHz, $CDCl_3$) **53**

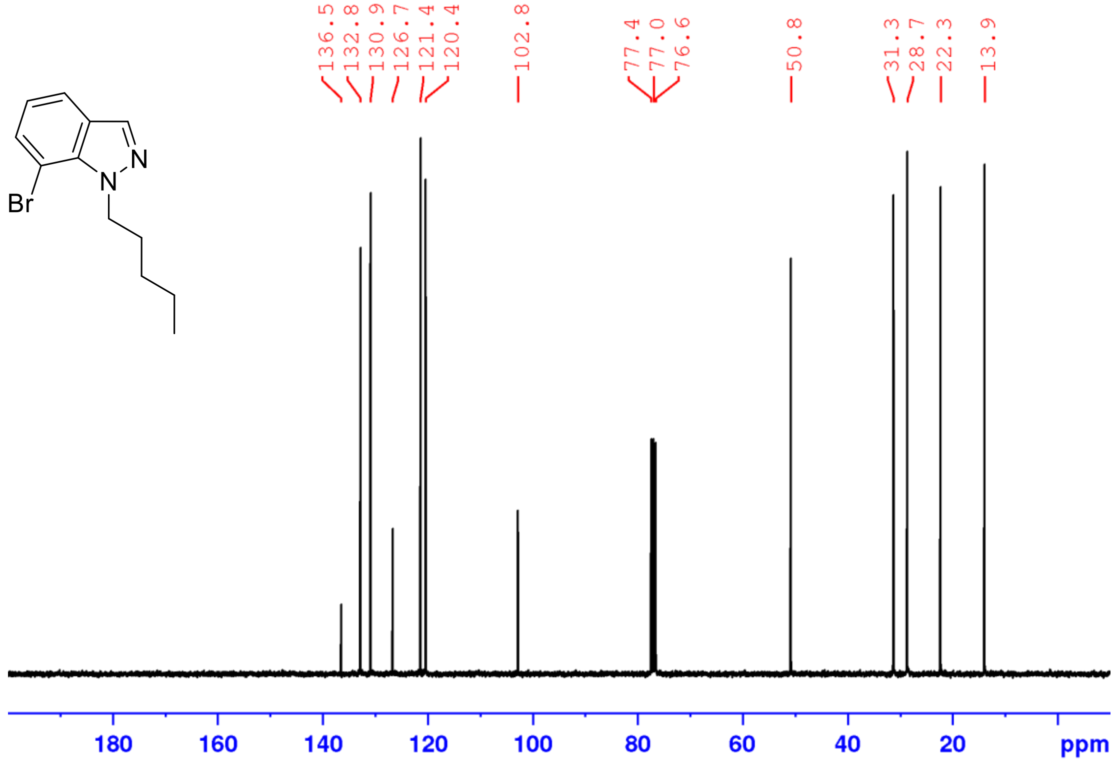

7-Bromo-2-*n*-pentyl-2*H*-indazole (54)

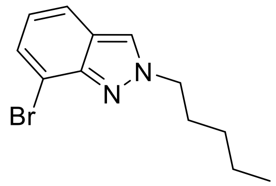

Following **General Procedure A** and/or **B**, wet flash column chromatography (Et_2O/hexane, 1:4) gave the title compound **54** (R_f = 0.17) as a colorless oil: IR (ATR, cm^{-1}) ν_{max} 2955, 2930, 2870, 2859, 1622, 1509, 1464, 1376, 1152, 1139, 937, 796, 738; ^{1}H NMR (300 MHz, $CDCl_3$) δ 7.97 (1H, s), 7.58 (1H, dd, J = 8.3, 0.8 Hz), 7.47 (1H, dd, J = 7.2, 0.9 Hz), 6.91 (1H, dd, J = 8.3, 7.2 Hz), 4.42 (2H, t, J = 7.4 Hz), 2.00 (2H, quint, J = 7.4 Hz), 1.40–1.24 (4H, m), 0.88 (3H, t, J = 6.9 Hz); ^{13}C NMR (75 MHz, $CDCl_3$) δ 147.1, 128.4, 123.7, 122.5, 122.0, 119.5, 110.8, 53.9, 30.3, 28.6, 22.2, 13.7; HRMS (ESI) *m/z*: $[M+H]^+$ Calcd for $C_{12}H_{16}{}^{79}BrN_2$ 267.0491, found 267.0488 (– 1.1 ppm).

^{1}H NMR (300 MHz, $CDCl_3$) **54**

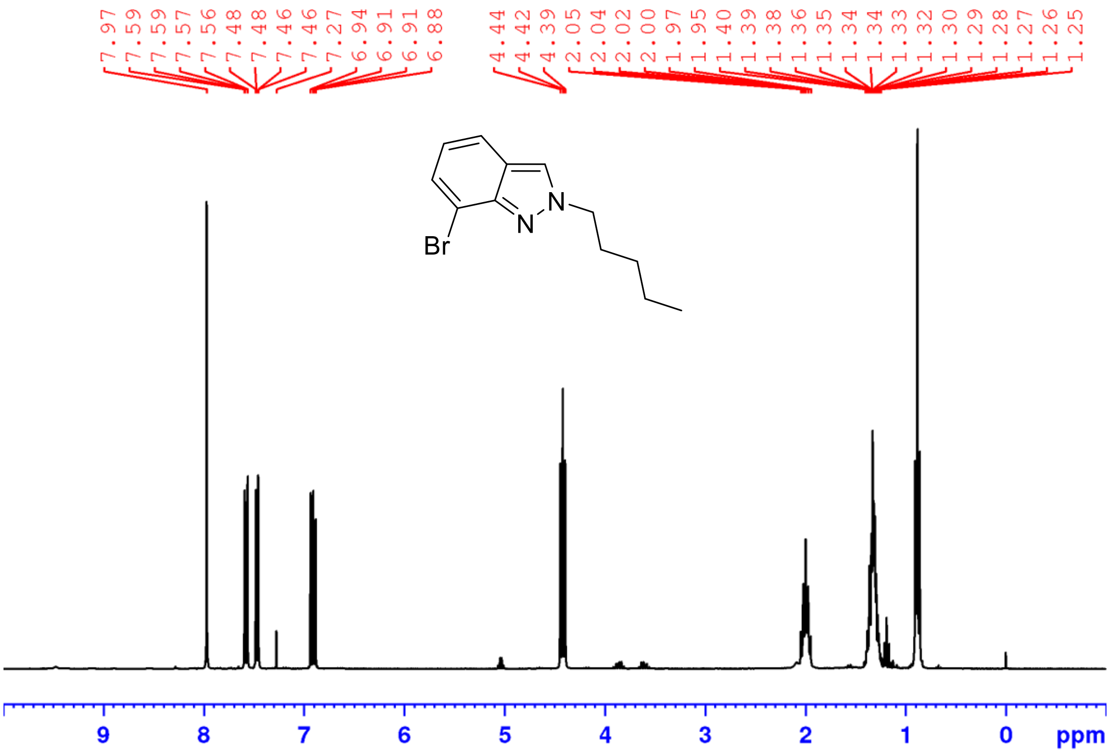

^{13}C NMR (75 MHz, $CDCl_3$) **54**

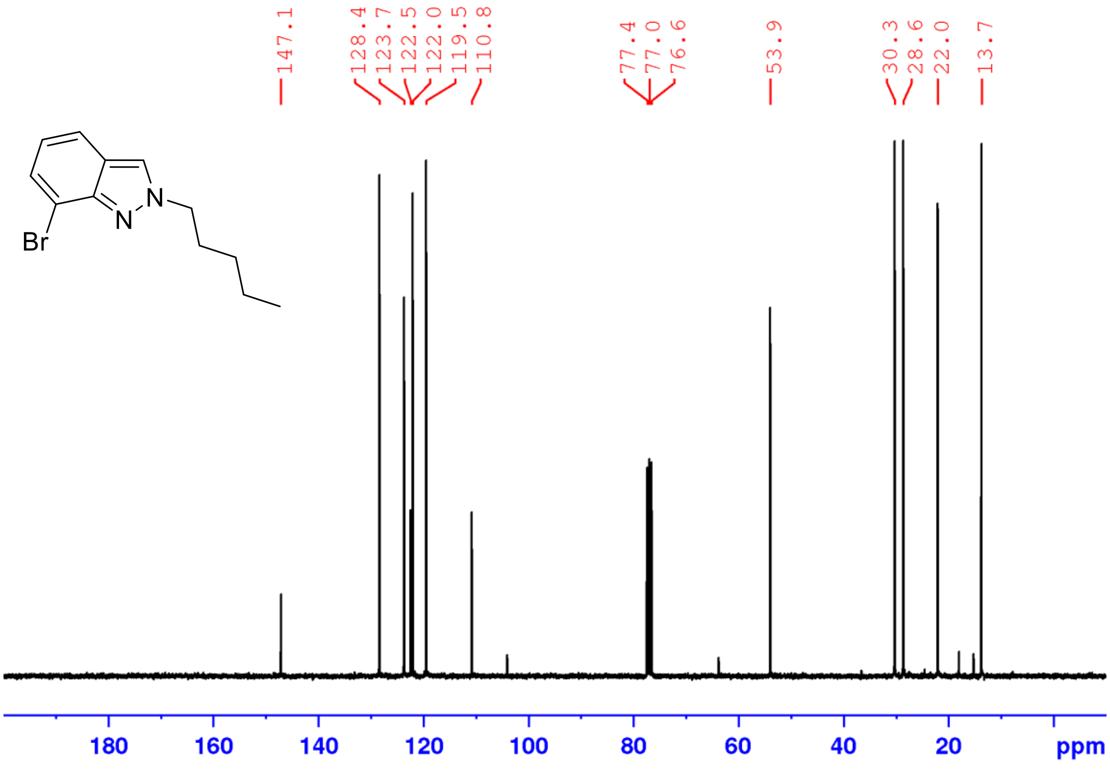

7-Nitro-1-*n*-pentyl-1*H*-indazole (55)

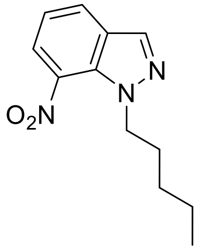

Following **General Procedure B**, wet flash column chromatography (EtOAc/hexane, 1:4) gave the title compound **55** (R_f = 0.66) as a yellow oil: IR (ATR, cm^{-1}) ν_{max} 2957, 2930, 2860, 1622, 1520, 1374, 1321, 996, 872, 842, 791, 729, 618; ^{1}H NMR (300 MHz, $CDCl_3$) δ 8.17 (1H, s), 8.09 (1H, dd, *J* = 7.7, 1.0 Hz), 8.02 (1H, dd, *J* = 8.0, 1.0 Hz), 7.21 (1H, t, *J* = 7.8 Hz), 4.61 (2H, t, *J* = 7.5 Hz), 1.76 (2H, quint, *J* = 7.4 Hz), 1.38–1.18 (4H, m), 0.86 (3H, t, *J* = 7.1 Hz); ^{13}C NMR (75 MHz, $CDCl_3$) δ 135.4, 134.1, 130.1, 129.0, 127.8, 124.5, 119.5, 53.2, 30.1, 28.6, 22.1, 13.8; HRMS (ESI) *m/z*: $[M+H]^+$ Calcd for $C_{12}H_{16}N_3O_2$ 234.1237, found 234.1230 (– 3.0 ppm).

^1H NMR (300 MHz, $CDCl_3$) **55**

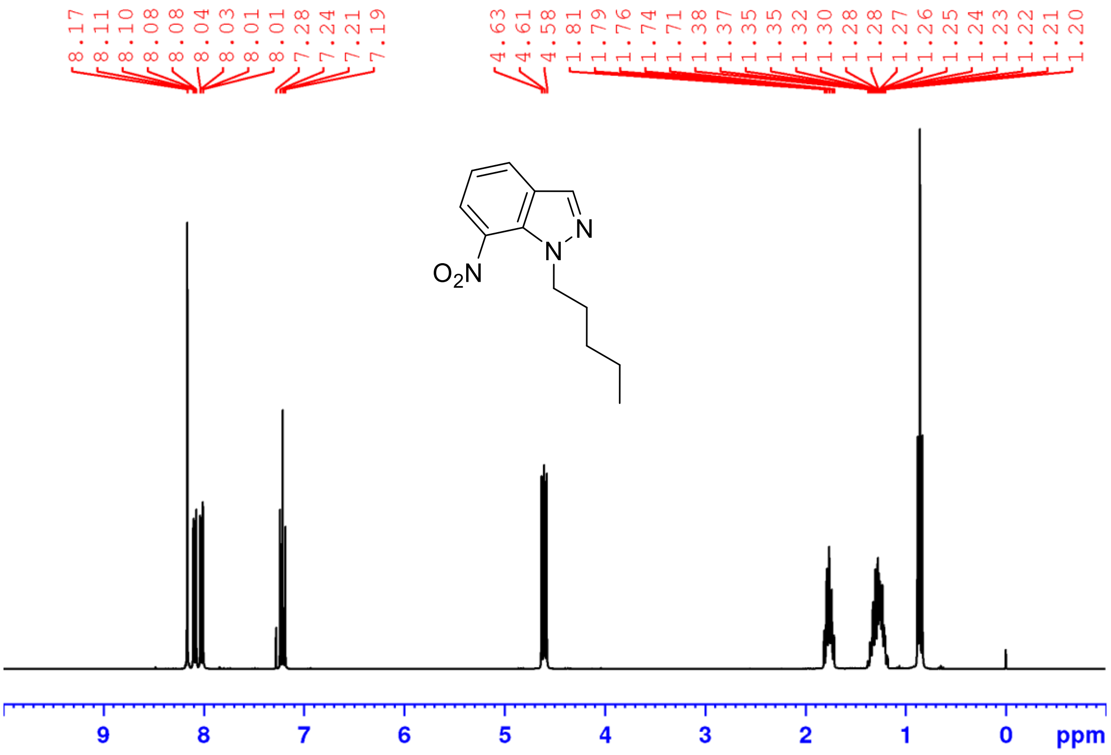

^{13}C NMR (75 MHz, $CDCl_3$) **55**

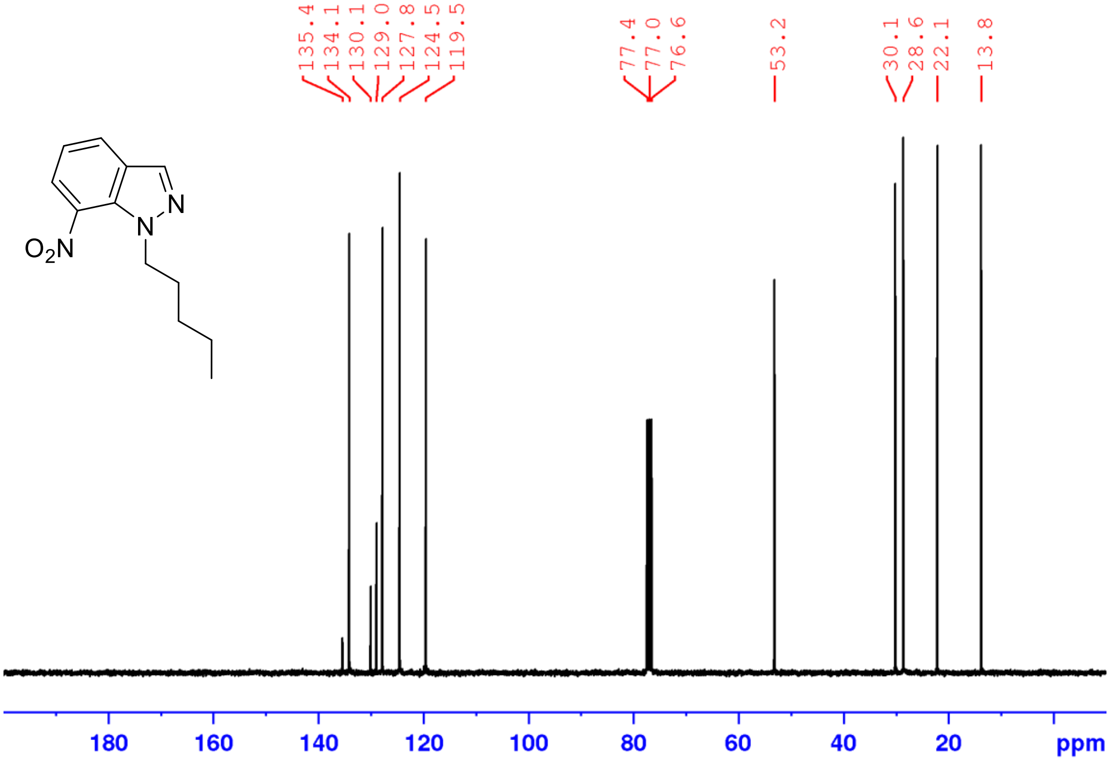

7-Nitro-2-*n*-pentyl-2*H*-indazole (56)

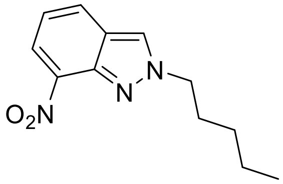

Following **General Procedure A** and/or **B**, wet flash column chromatography (EtOAc/hexane, 1:4) gave the title compound **56** (R_f = 0.17) as a yellow oil: IR (ATR, cm^{-1}) ν_{max} 3123, 1956, 2931, 2861, 1634, 1512, 1331, 1298, 1151, 992, 818, 740; ^{1}H NMR (300 MHz, $CDCl_3$) δ 8.33 (1H, dd, *J* = 7.6, 1.0 Hz), 8.20 (1H, s), 8.05 (1H, dd, *J* = 8.2, 1.0 Hz), 7.18 (1H, dd, *J* = 8.2, 7.6 Hz), 4.55 (2H, t, *J* = 7.4 Hz), 2.07 (2H, quint, *J* = 7.4 Hz), 1.45–1.29 (4H, m), 0.90 (3H, t, *J* = 6.9 Hz); ^{13}C NMR (75 MHz, $CDCl_3$) δ 140.6, 137.6, 128.7, 125.5, 125.0, 124.9, 120.0, 54.5, 30.3, 28.7, 22.2, 13.9; HRMS (ESI) *m/z*: [M+H]$^+$ Calcd for $C_{12}H_{16}N_3O_2$ 234.1237, found 234.1231 (– 2.6 ppm).

^{1}H NMR (300 MHz, $CDCl_3$) **56**

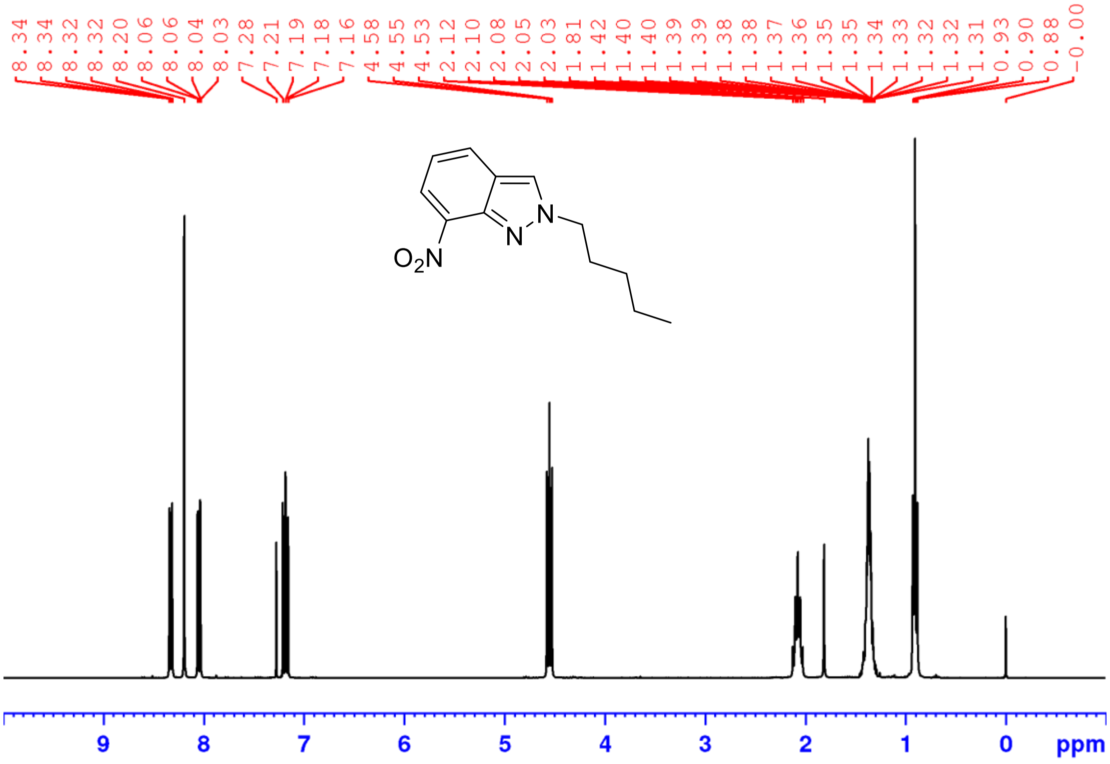

^{13}C NMR (75 MHz, $CDCl_3$) **56**

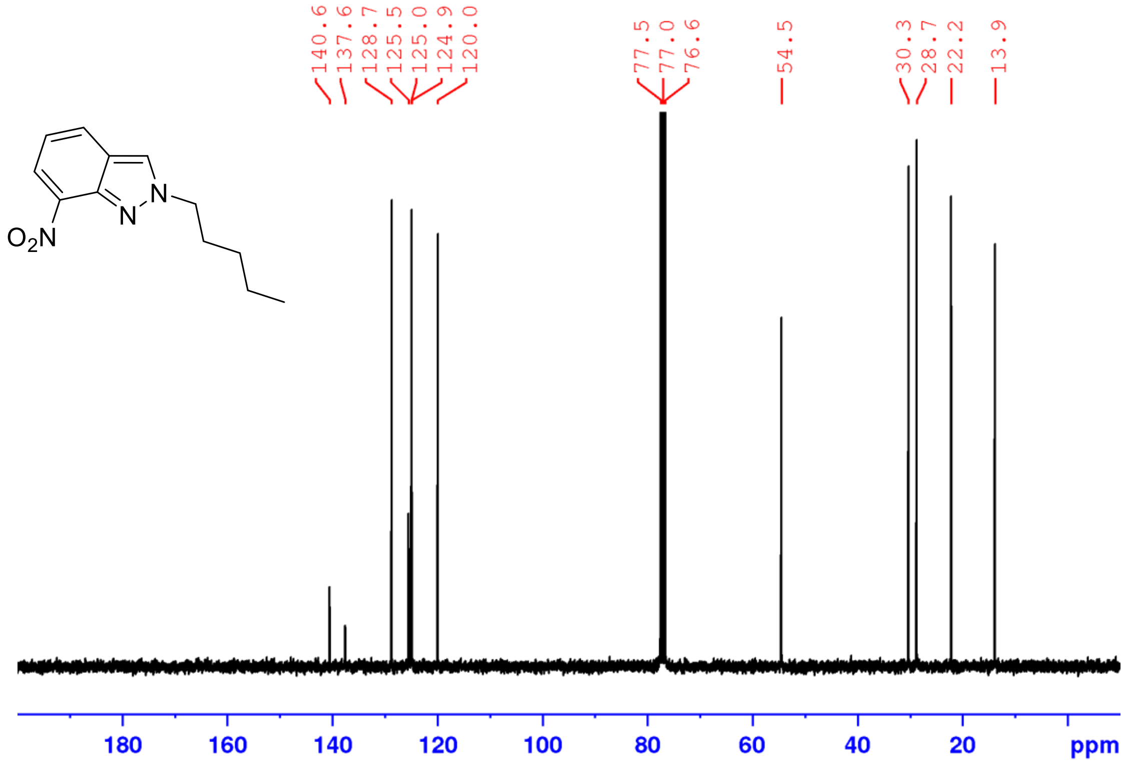

Methyl 1-*n*-pentyl-1*H*-indazole-7-carboxylate (57)

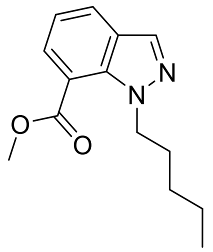

Following **General Procedure B**, wet flash column chromatography (Et_2O/hexane, 1:4) gave the title compound **57** (R_f = 0.67) as a colorless oil: IR (ATR, cm^{-1}) ν_{max} 2954, 2931, 2860, 1720, 1454, 1435, 1263, 1212, 1136, 1115, 856, 839, 751, 742, 642; ^1H NMR (300 MHz, $CDCl_3$) δ 8.06 (1H, s), 7.92–7.78 (2H, m), 7.13 (1H, dd, J = 8.0, 7.4 Hz), 4.71 (2H, t, J = 7.4 Hz), 3.97 (3H, s), 1.77 (2H, quint, J = 7.4 Hz), 1.38–1.18 (4H, m), 0.86 (3H, t, J = 7.0 Hz); ^{13}C NMR (75 MHz, $CDCl_3$) δ 166.8, 136.0, 133.5, 129.9, 126.6, 125.8, 119.4, 115.4, 52.3, 52.2, 29.9, 28.7, 22.2, 13.8; HRMS (ESI) *m/z*: $[M+H]^+$ Calcd for $C_{14}H_{19}N_2O_2$ 247.1441, found 247.1440 (– 0.4 ppm).

^{1}H NMR (300 MHz, $CDCl_3$) **57**

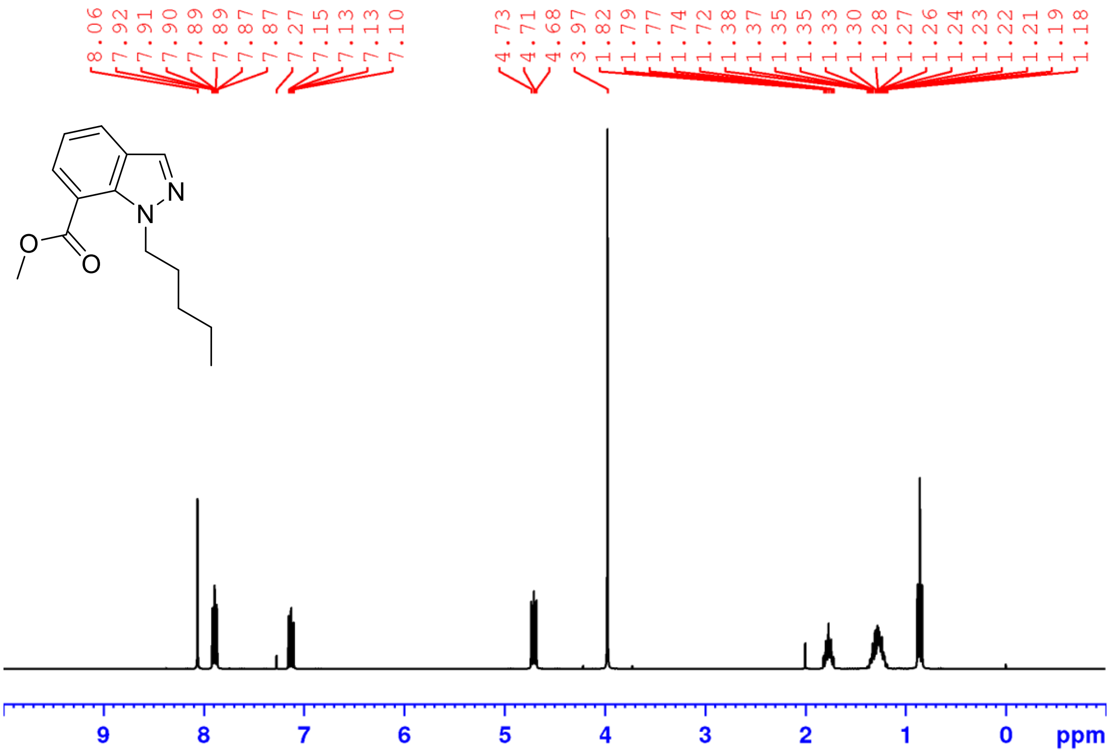

^{13}C NMR (75 MHz, $CDCl_3$) **57**

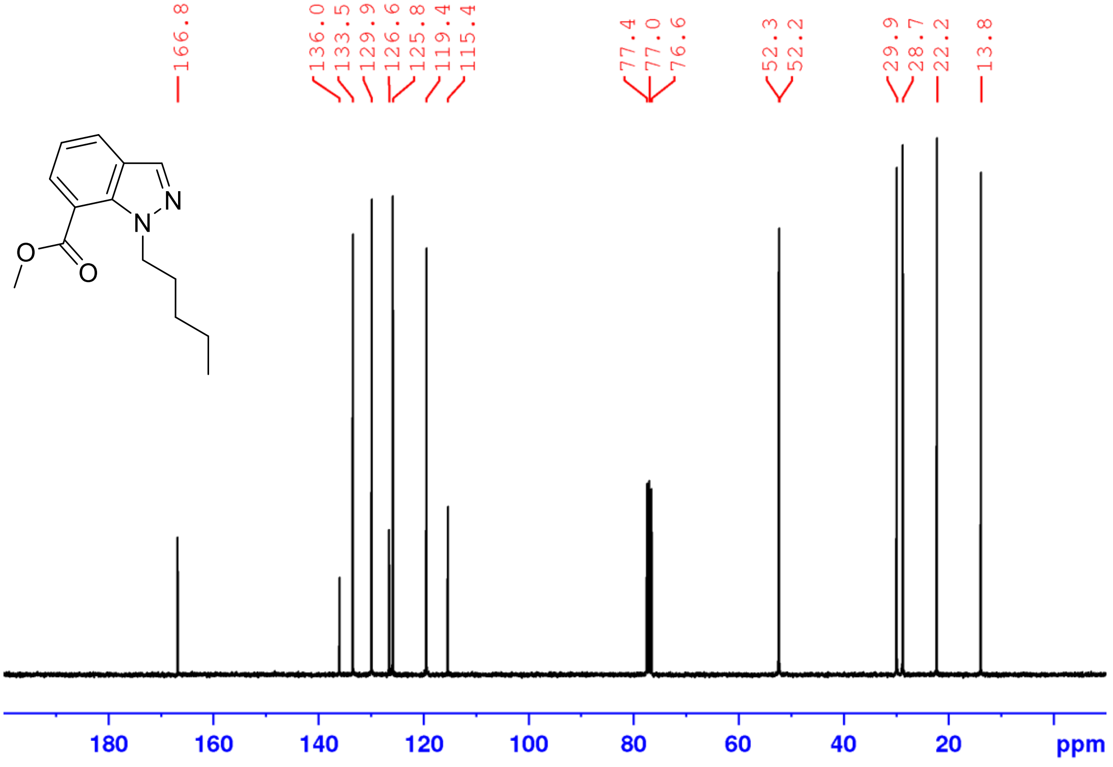

Methyl 2-*n*-pentyl-2*H*-indazole-7-carboxylate (58)

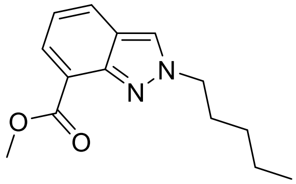

Following **General Procedure A** and/or **B**, wet flash column chromatography (Et_2O/hexane, 1:4) gave the title compound **58** (R_f = 0.10) as a colorless oil: IR (ATR, cm^{-1}) v_{max} 3113, 2952, 2932, 2871, 1706, 1277, 1265, 1200, 1136, 1038, 755; ^1H NMR (300 MHz, $CDCl_3$) δ 8.08 (1H, dd, J = 7.1, 1.1 Hz), 8.03 (1H, s), 7.88 (1H, dd, J = 8.3, 1.1 Hz), 7.12 (1H, dd, J = 8.3, 7.1 Hz), 4.49 (2H, t, J = 4.5 Hz), 4.01 (3H, s), 2.05 (2H, quint, J = 7.4 Hz), 1.44–1.29 (4H, m), 0.90 (3H, t, J = 6.9 Hz); ^{13}C NMR (75 MHz, $CDCl_3$) δ 166.5, 145.9, 130.4, 125.9, 123.4, 123.2, 120.4, 119.0, 54.0, 52.0, 30.2, 28.7, 22.1, 13.8; HRMS (ESI) *m/z*: $[M+H]^+$ Calcd for $C_{14}H_{19}N_2O_2$ 247.1441, found 247.1440 (– 0.4 ppm).

^{1}H NMR (300 MHz, $CDCl_3$) **58**

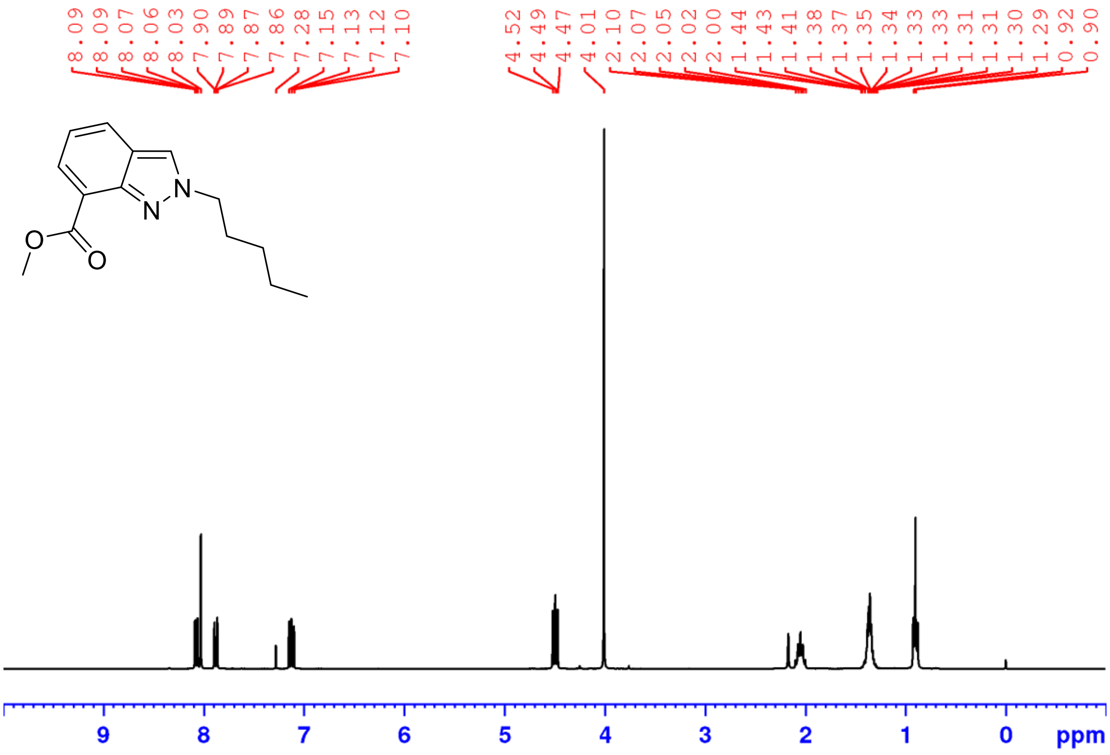

^{13}C NMR (75 MHz, $CDCl_3$) **58**

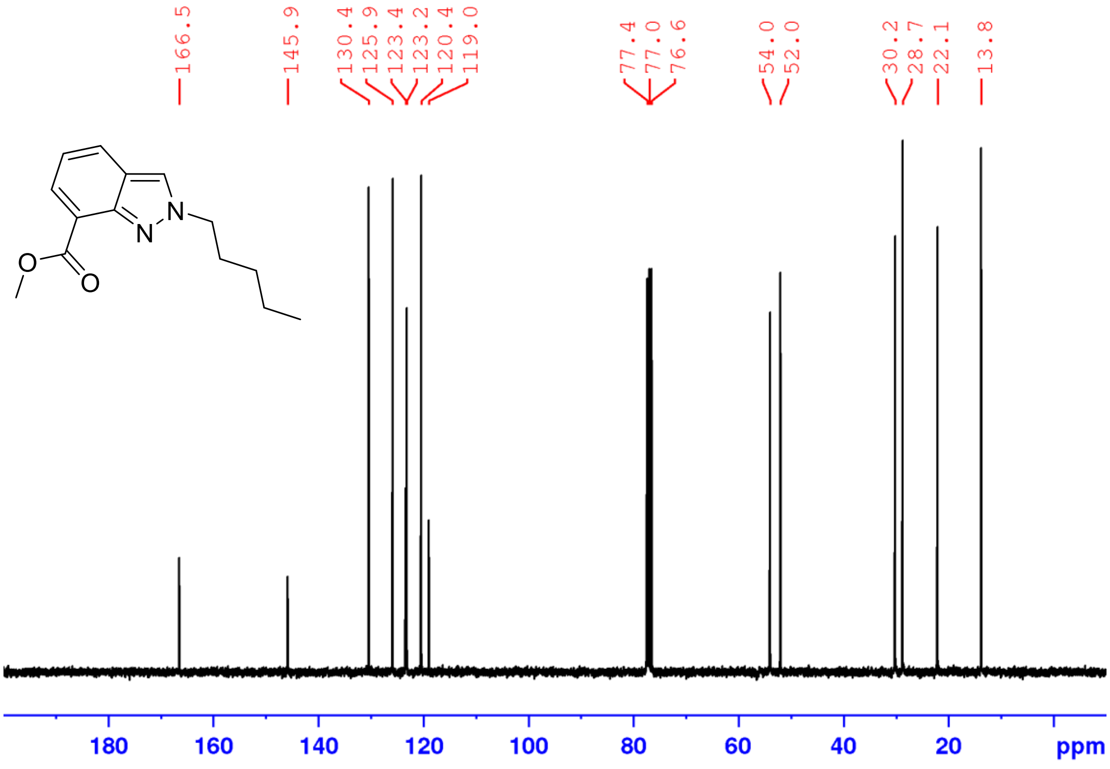

Methyl 1-*n*-pentyl-1*H*-indazole-6-carboxylate (59)

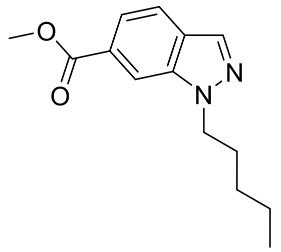

Following **General Procedure A** and/or **B**, wet flash column chromatography (Et_2O/hexane, 1:4) gave the title compound **59** (R_f = 0.27) as a colorless oil: IR (ATR, cm^{-1}) ν_{max} 2954, 2932, 2860, 1717, 1434, 1275, 1252, 1234, 1084, 761, 741, 630, 429; ^{1}H NMR (300 MHz, $CDCl_3$) δ 8.12 (1H, dd, *J* = 2.0, 1.0 Hz), 8.03 (1H, d, *J* = 1.0 Hz), 7.79 (1H, dd, *J* = 8.5, 1.3 Hz), 7.74 (1H, dd, *J* = 8.5, 1.3 Hz), 4.42 (2H, t, *J* = 7.2 Hz), 3.97 (3H,s), 1.94 (2H, quint, *J* = 7.3 Hz), 1.43–1.24 (4H, m), 0.88 (3H, t, *J* = 6.9 Hz); ^{13}C NMR (75 MHz, $CDCl_3$) δ 167.3, 138.8, 132.6, 127.7, 126.4, 120.8, 120.7, 111.4, 52.2, 49.1, 29.5, 28.8, 22.2, 13.8; HRMS (ESI) *m/z*: $[M+H]^+$ Calcd for $C_{14}H_{19}N_2O_2$ 247.1441, found 247.1436 (– 2.0 ppm).

^{1}H NMR (300 MHz, $CDCl_3$) **59**

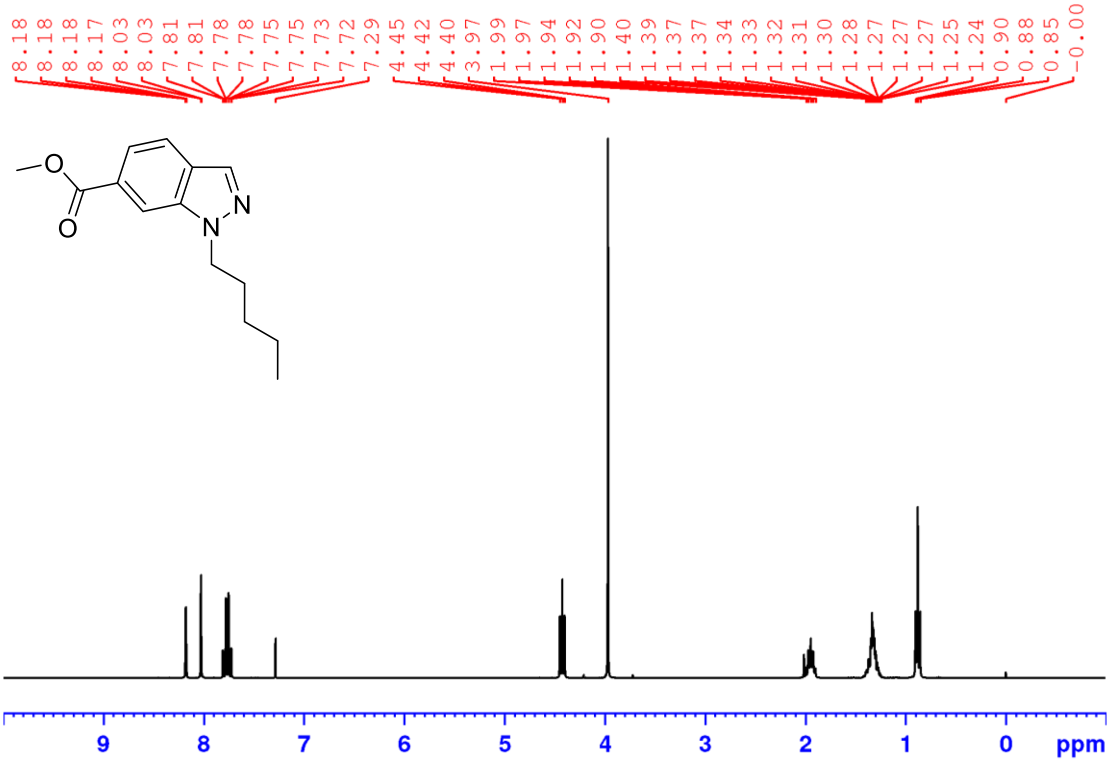

^{13}C NMR (75 MHz, $CDCl_3$) **59**

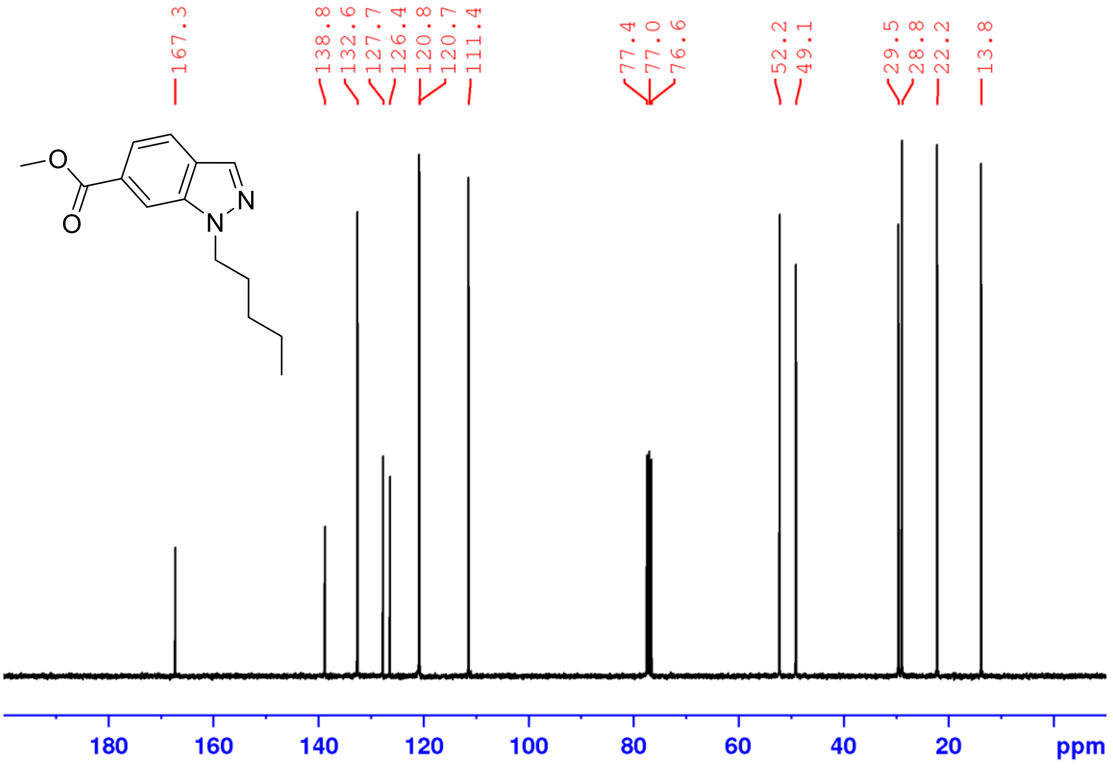

Methyl 2-*n*-pentyl-2*H*-indazole-6-carboxylate (60)

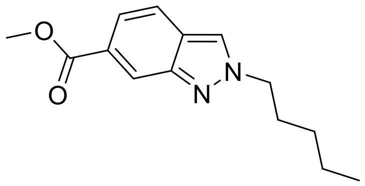

Following **General Procedure A** and/or **B**, wet flash column chromatography (Et_2O/hexane, 1:4) gave the title compound **60** (R_f = 0.07) as a colorless oil: IR (ATR, cm^{-1}) ν_{max} 2953, 2932, 2871, 1713, 1434, 1326, 1244, 1221, 1083, 747, 436; ^1H NMR (300 MHz, $CDCl_3$) δ 8.51 (1H, dd, J = 2.1, 1.0 Hz), 7.93 (1H, d, J = 0.8 Hz), 7.71 (1H, dd, J = 8.8, 1.3 Hz), 7.66 (1H, dd, J = 8.8, 0.9 Hz), 4.42 (2H, t, J = 7.2 Hz), 3.94 (3H, s), 2.02 (2H, quint, J = 7.3 Hz), 1.43–1.25 (4H, m), 0.89 (3H, t, J = 7.0 Hz); ^{13}C NMR (75 MHz, $CDCl_3$) δ 167.5, 147.9, 127.5, 123.8, 122.7, 121.13, 121.12, 120.0, 54.1, 52.0, 30.2, 28.6, 22.1, 13.7; HRMS (ESI) *m/z*: $[M+H]^+$ Calcd for $C_{14}H_{19}N_2O_2$ 247.1441, found 247.1436 (– 2.0 ppm).

^{1}H NMR (300 MHz, $CDCl_3$) **60**

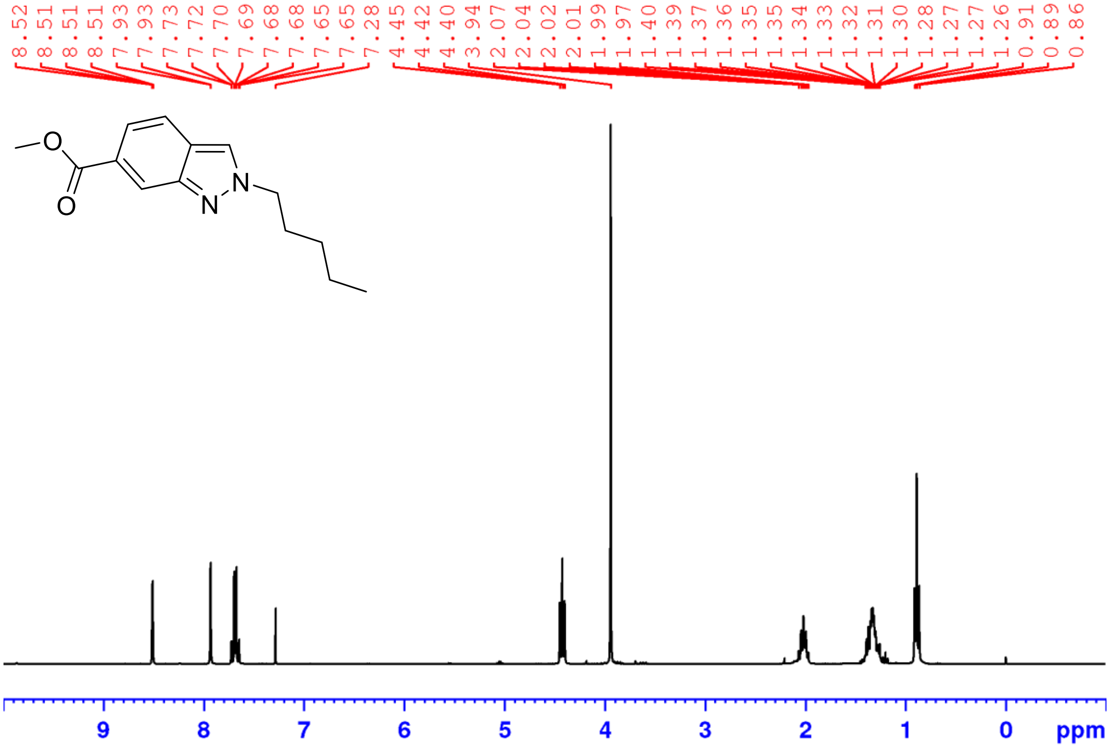

^{13}C NMR (75 MHz, $CDCl_3$) **60**

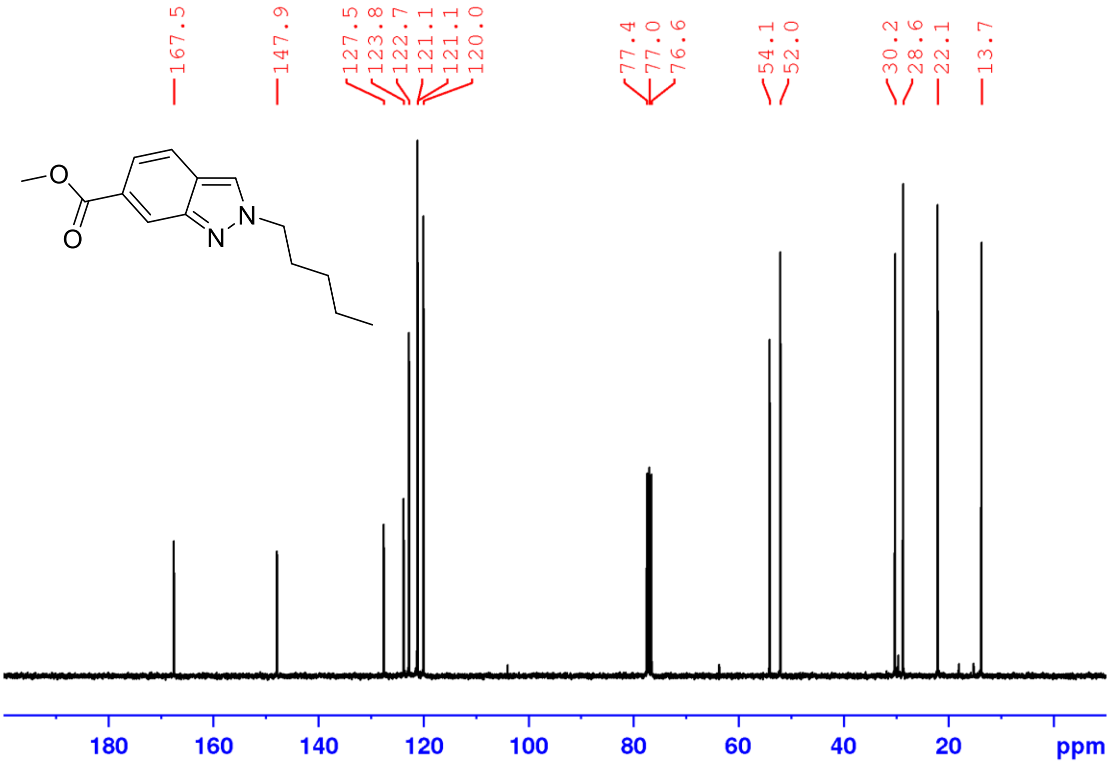

Methyl 1-*n*-pentyl-1*H*-indazole-5-carboxylate (61)

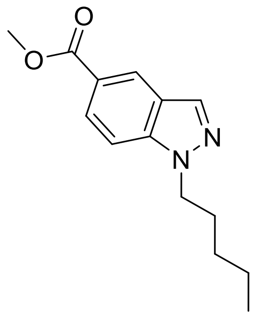

Following **General Procedure A** and/or **B**, wet flash column chromatography (EtOAc/hexane, 1:4) gave the title compound **61** (R_f = 0.43) as an amber oil: IR (ATR, cm^{-1}) ν_{max} 2954, 2932, 2871, 1713, 1619, 1435, 1313, 1250, 1178, 1088, 767; ^{1}H NMR (300 MHz, $CDCl_3$) δ 8.51 (1H, dd, *J* = 1.5, 0.8 Hz), 8.09 (1H, d, *J* = 0.9 Hz), 8.05 (1H, dd, *J* = 8.9, 1.5 Hz), 7.41 (1H, ddd, *J* = 8.9, 0.8, 0.8 Hz), 4.38 (2H, t, *J* = 7.1 Hz), 3.94 (3H, s), 1.94 (2H, quint, *J* = 7.3 Hz), 1.42–1.23 (4H, m), 0.88 (3H, t, *J* = 7.0 Hz); ^{13}C NMR (75 MHz, $CDCl_3$) δ 167.3, 141.1, 134.5, 126.9, 124.7, 123.6, 122.7, 108.7, 52.0, 49.1, 29.5, 28.9, 22.2, 13.9; HRMS (ESI) *m/z*: [M+H]$^+$ Calcd for $C_{14}H_{19}N_2O_2$ 247.1441, found 247.1435 (– 2.4 ppm).

^{1}H NMR (300 MHz, $CDCl_3$) **61**

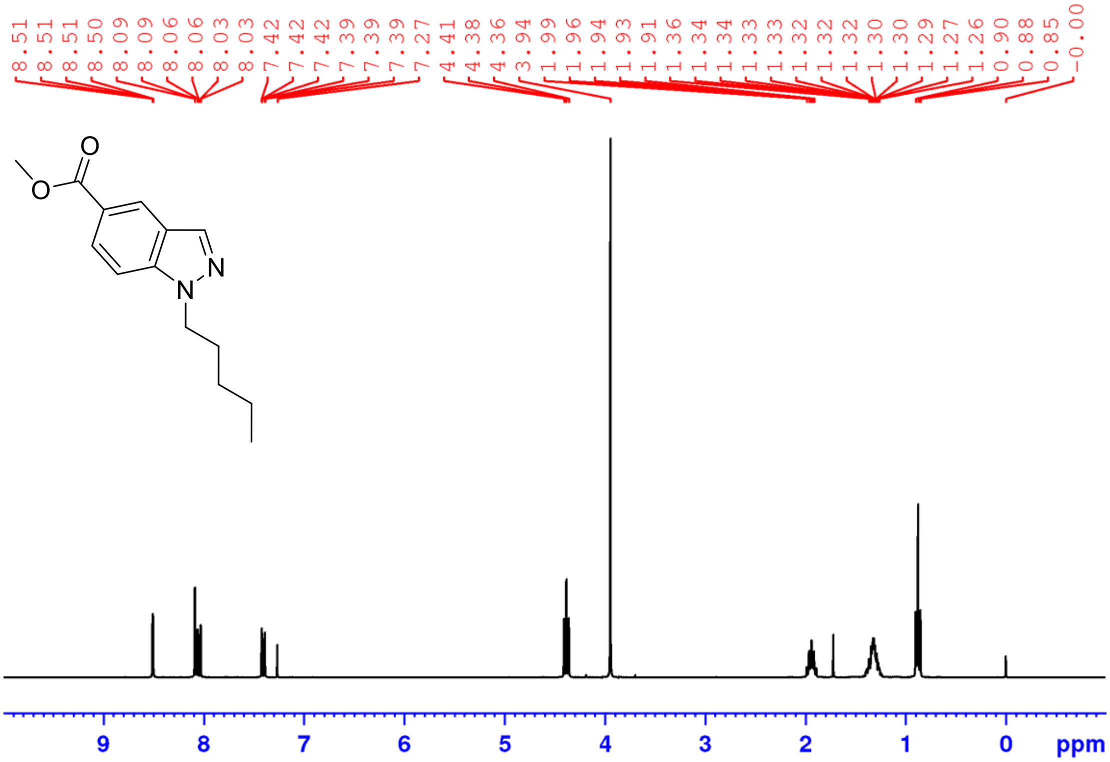

^{13}C NMR (75 MHz, $CDCl_3$) **61**

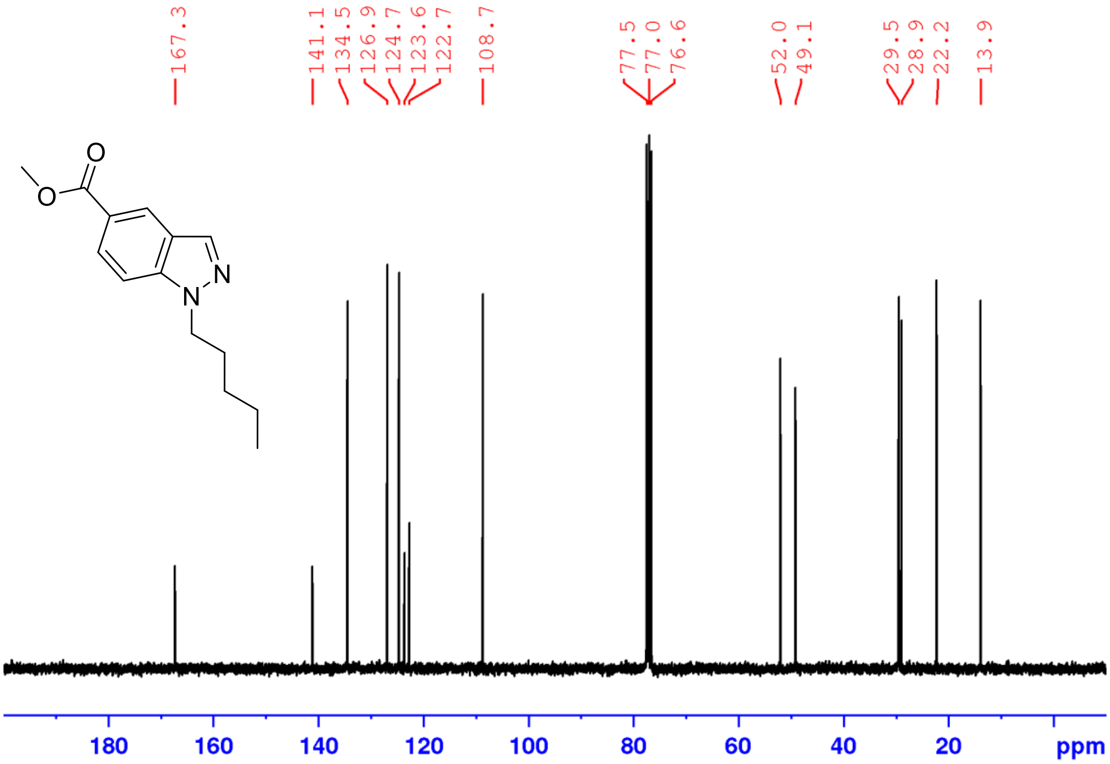

Methyl 2-*n*-pentyl-2*H*-indazole-5-carboxylate (62)

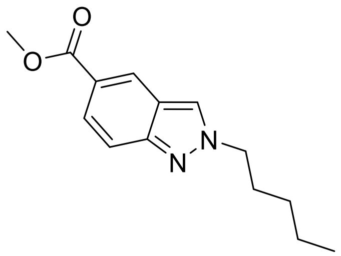

Following **General Procedure A** and/or **B**, wet flash column chromatography (EtOAc/hexane, 1:4) gave the title compound **62** (R_f = 0.23) as a yellow crystalline solid: m.p. 47–48 °C; IR (ATR, cm^{-1}) ν_{max} 2953, 2933, 2871, 1709, 1628, 1435, 1309, 1239, 1146, 1086, 769, 438; ^{1}H NMR (300 MHz, $CDCl_3$) δ 8.49 (1H, dd, J = 1.5. 0.9 Hz), 8.04 (1H, s), 7.90 (1H, dd, J = 9.1, 1.6 Hz), 7.70 (1H, ddd, J = 9.1, 0.9, 0.9 Hz), 4.41 (2H, t, J = 7.2 Hz), 3.93 (3H, s), 2.02 (2H, quint, J = 7.3 Hz), 1.43–1.25 (4H, m), 0.89 (3H, t, J = 6.9 Hz); ^{13}C NMR (75 MHz, $CDCl_3$) δ 167.4, 150.0, 125.5, 124.9, 124.7, 123.5, 121.0, 117.1, 54.0, 51.9, 30.1, 28.6, 22.1, 13.8; HRMS (ESI) *m/z*: $[M+H]^+$ Calcd for $C_{14}H_{19}N_2O_2$ 247.1441, found 247.1431 (– 4.0 ppm).

^{1}H NMR (300 MHz, $CDCl_3$) **62**

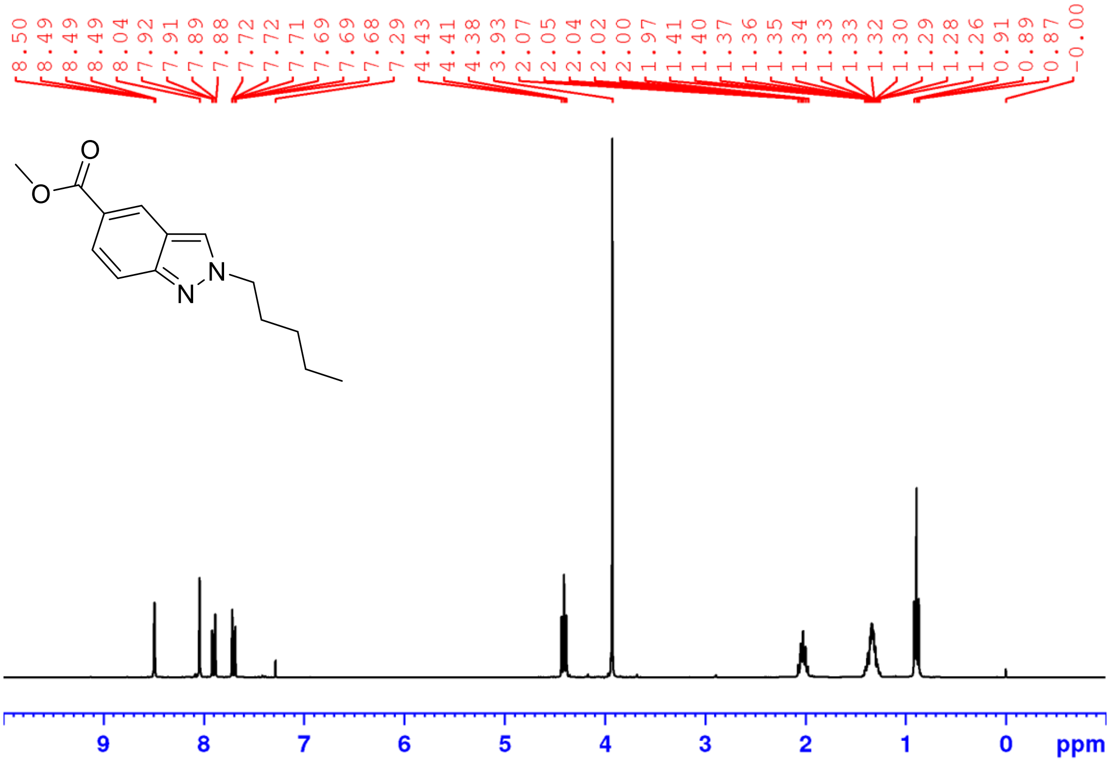

^{13}C NMR (75 MHz, $CDCl_3$) **62**

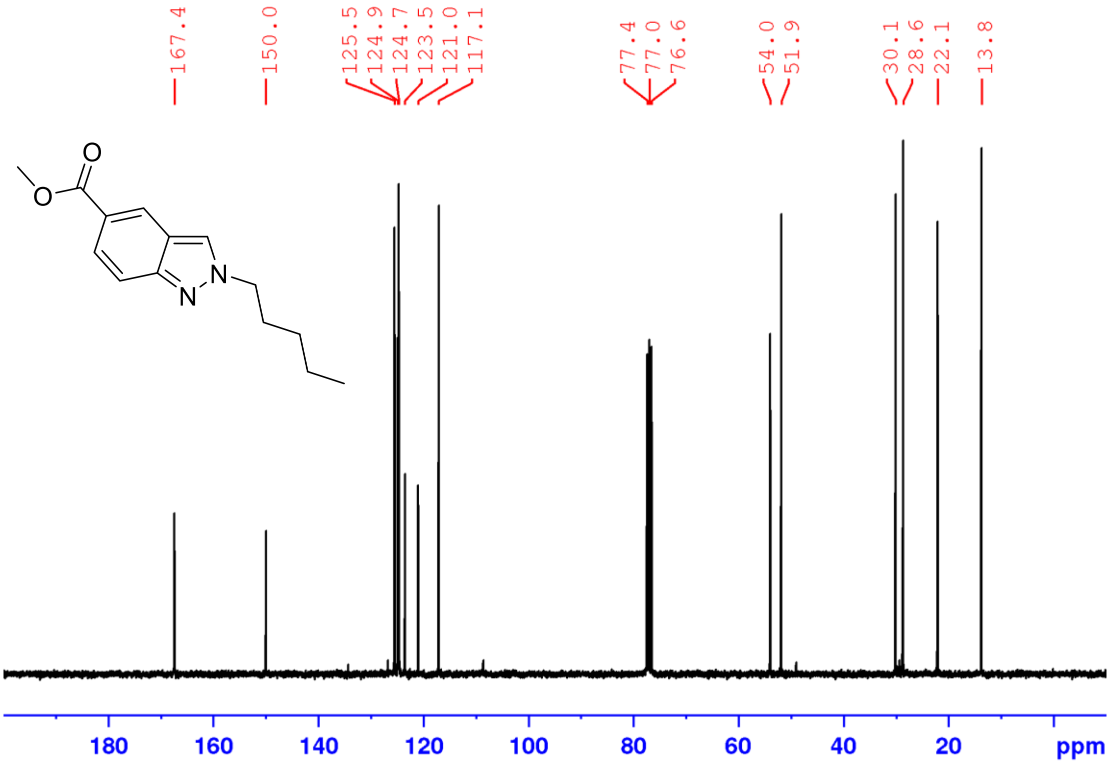

Methyl 1-*n*-pentyl-1*H*-indazole-4-carboxylate (63)

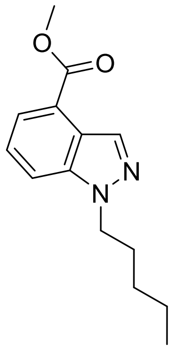

Following **General Procedure A** and/or **B**, wet flash column chromatography (EtOAc/hexane, 1:4) gave the title compound **63** (R_f = 0.50) as a pale yellow oil: IR (ATR, cm^{-1}) ν_{max} 2953, 2932, 1714, 1698, 1450, 1274, 1167, 1132, 925, 751; ^{1}H NMR (300 MHz, $CDCl_3$) δ 8.49 (1H, d, *J* = 0.8 Hz), 7.91 (1H, dd, *J* = 7.2, 0.7 Hz), 7.62 (1H, d, *J* = 8.4 Hz), 7.42 (1H, dd, *J* = 8.4, 7.2 Hz), 4.41 (2H, t, *J* = 7.1 Hz), 4.01 (3H, s), 1.93 (2H, quint, *J* = 7.3 Hz), 1.41–1.23 (4H, m), 0.87 (3H, t, *J* = 7.0 Hz); ^{13}C NMR (75 MHz, $CDCl_3$) δ 166.8, 139.8, 133.6, 125.3, 124.0, 123.0, 122.4, 113.9, 52.1, 49.1, 29.6, 29.0, 22.3, 13.9; HRMS (ESI) *m/z*: $[M+H]^+$ Calcd for $C_{14}H_{19}N_2O_2$ 247.1441, found 247.1436 (– 2.0 ppm).

^{1}H NMR (300 MHz, $CDCl_3$) **63**

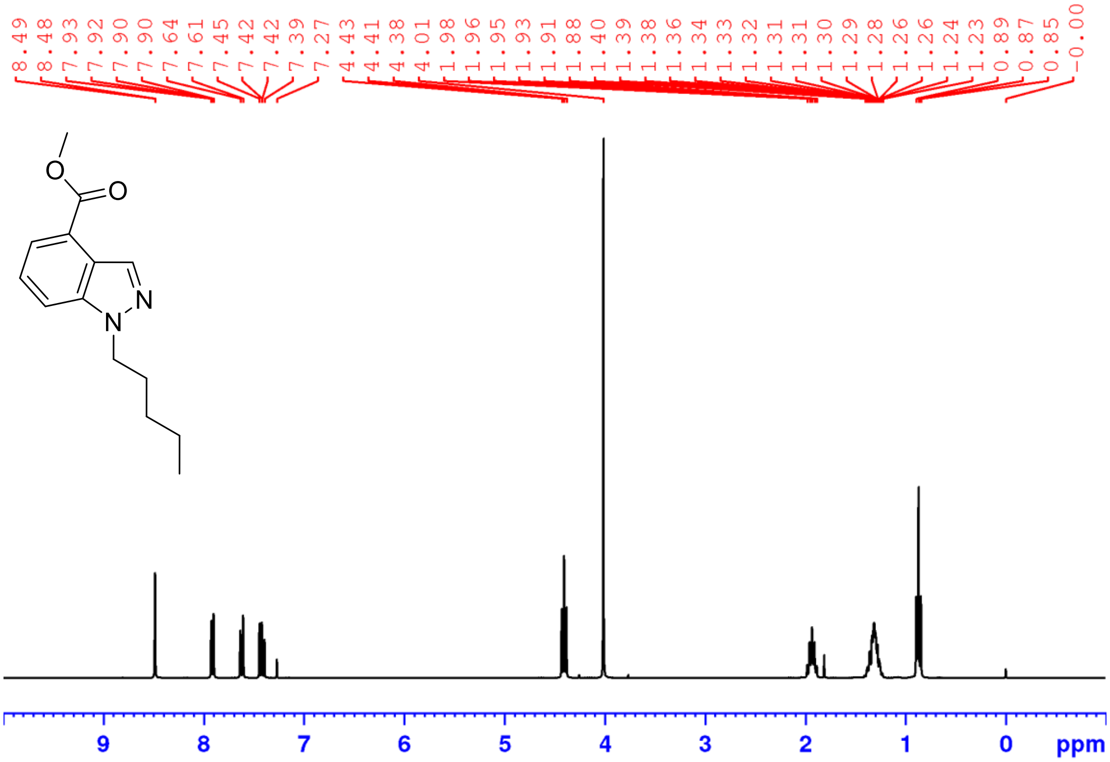

^{13}C NMR (75 MHz, $CDCl_3$) **63**

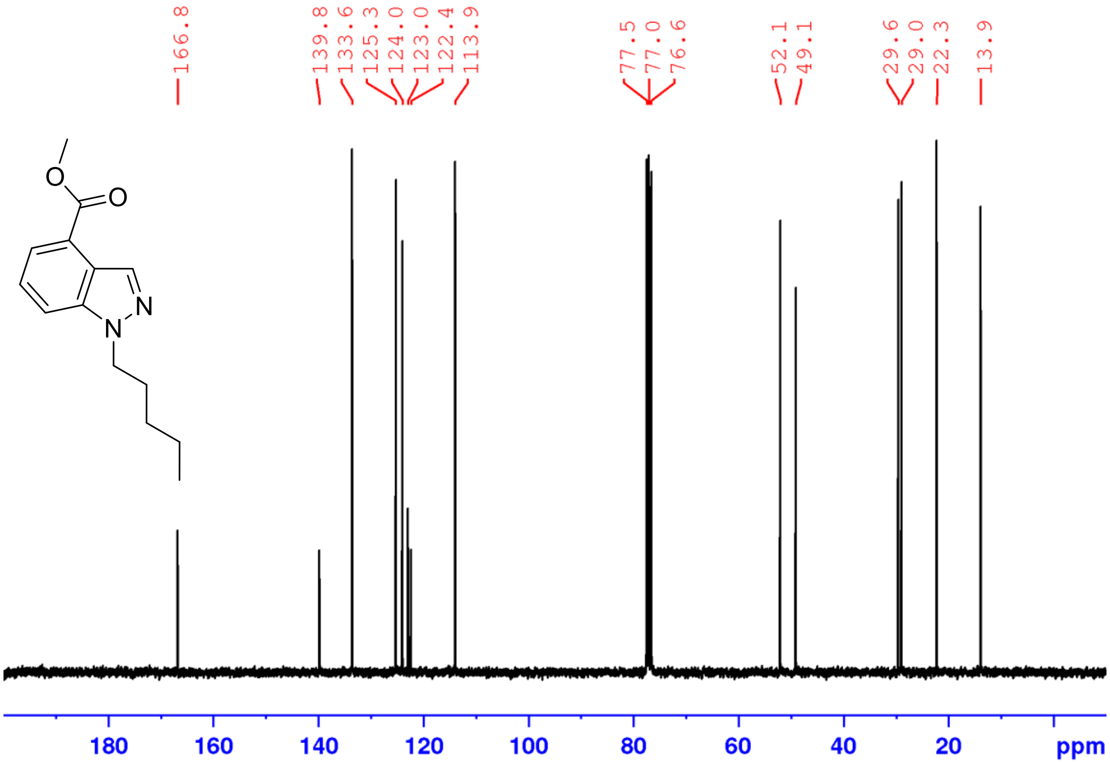

Methyl 2-*n*-pentyl-2*H*-indazole-4-carboxylate (64)

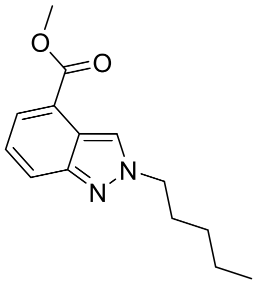

Following **General Procedure A** and/or **B**, wet flash column chromatography (EtOAc/hexane, 1:4) gave the title compound **64** (R_f = 0.33) as a colorless oil: IR (ATR, cm^{-1}) ν_{max} 2953, 2932, 2871, 2860, 1709, 1435, 1380, 1269, 1202, 1172, 1136, 1047, 785, 756; ^{1}H NMR (300 MHz, $CDCl_3$) δ 8.43 (1H, d, J = 0.7 Hz), 7.94 (1H, ddd, J = 8.6, 0.8, 0.8 Hz), 7.90 (1H, dd, J = 7.1, 0.8 Hz), 7.33 (1H, dd, J = 8.6, 7.1 Hz), 4.44 (2H, t, J = 7.2 Hz), 3.98 (3H, s), 2.04 (2H, quint, J = 7.3 Hz), 1.44–1.25 (4H, m), 0.90 (3H, t, J = 6.9 Hz); ^{13}C NMR (75 MHz, $CDCl_3$) δ 166.8, 149.0, 126.0, 124.7, 124.4, 123.2, 122.2, 120.0, 53.9, 51.9, 30.3, 28.7, 22.1, 13.8; HRMS (ESI) *m/z*: $[M+H]^+$ Calcd for $C_{14}H_{19}N_2O_2$ 247.1441, found 247.1436 (– 2.0 ppm).

^1H NMR (300 MHz, $CDCl_3$) **64**

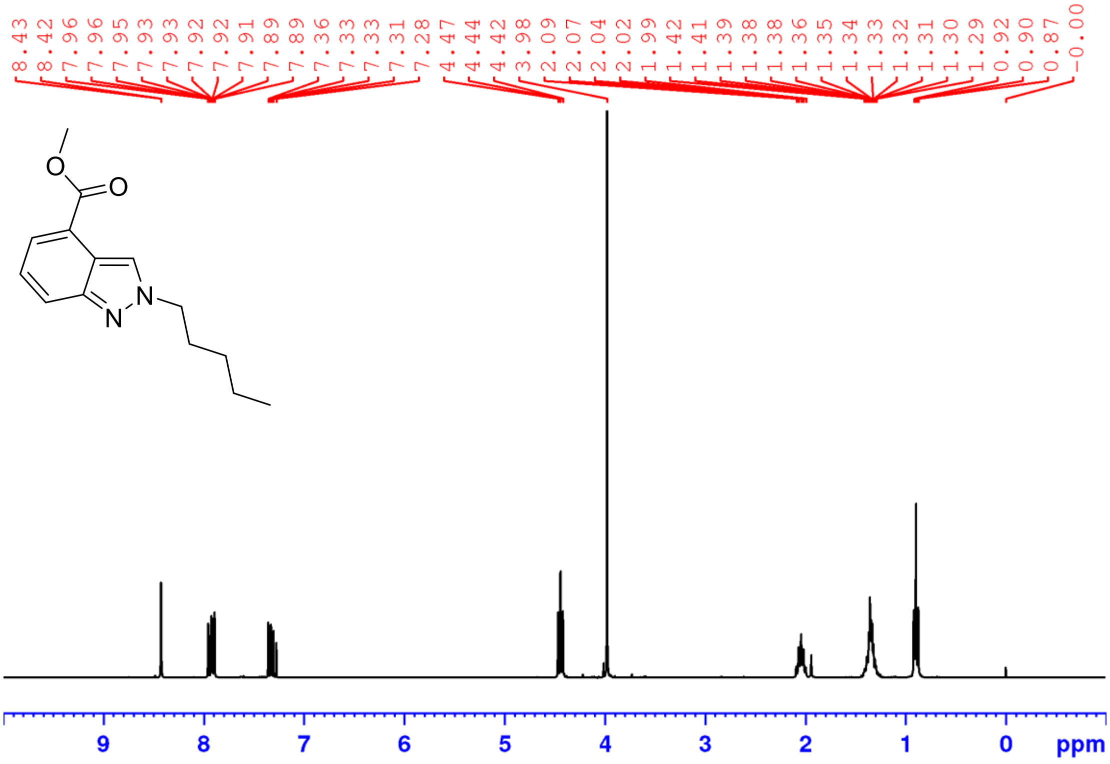

^{13}C NMR (75 MHz, $CDCl_3$) **64**

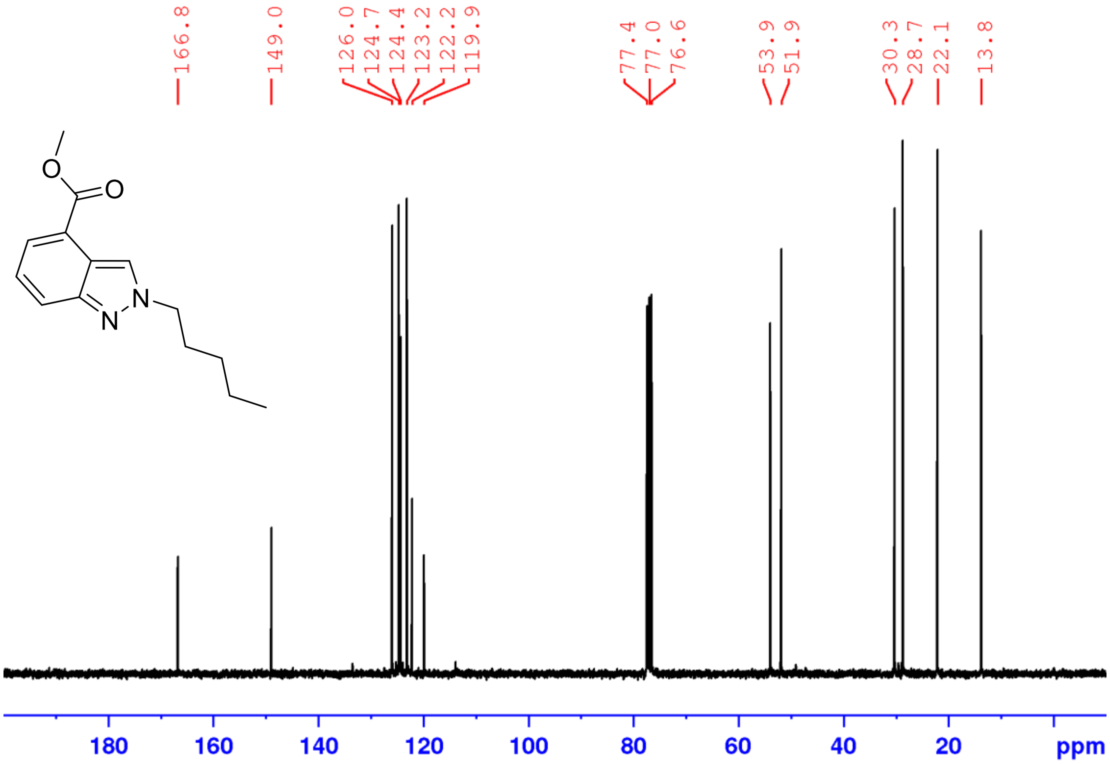

Methyl 1-benzyl-1*H*-indazole-3-carboxylate (69)

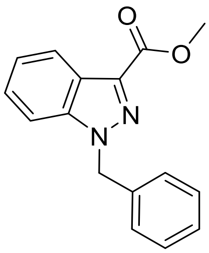

Following **General Procedure A** and/or **B**, wet flash column chromatography (EtOAc/hexane, 1:4) gave the title compound **69** (R_f = 0.36) as a yellow oil which solidified upon standing at room temperature: m.p. 79 °C (lit. m.p. 80–82 °C)[14]; IR (ATR, cm^{-1}) ν_{max} 3032, 2951, 1709, 1478, 1439, 1228, 1160, 1123, 729; ^1H NMR (300 MHz, $CDCl_3$) δ 8.24 (1H, ddd, J = 8.0, 1.2, 0.9 Hz), 7.40–7.19 (8H, m), 5.70 (2H, s), 4.05 (3H, s); ^{13}C NMR (75 MHz, $CDCl_3$) δ 163.1, 140.5, 135.6, 135.0, 128.8, 128.1, 127.2, 127.0, 124.1, 123.2, 122.2, 110.0, 54.1, 52.0; HRMS (ESI) *m/z*: $[M+H]^+$ Calcd for $C_{16}H_{15}N_2O_2$ 267.1128, found 267.1121 (– 2.6 ppm). Spectral data were in agreement with literature values [14].

^{1}H NMR (300 MHz, $CDCl_3$) **69**

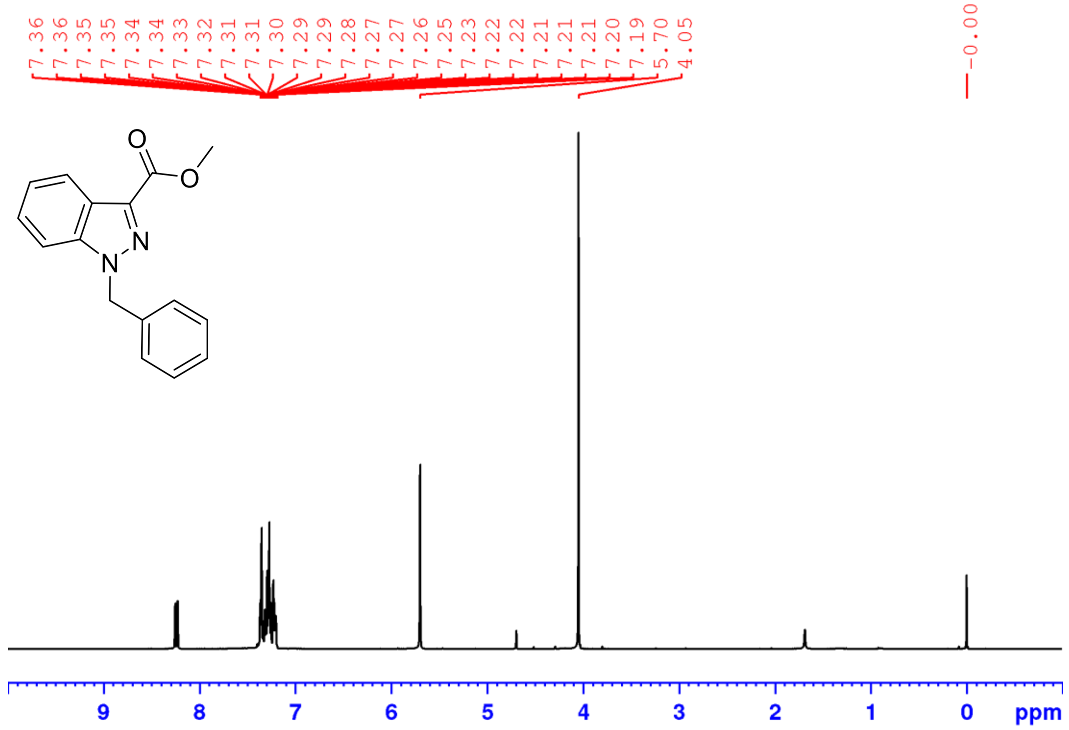

^{13}C NMR (75 MHz, $CDCl_3$) **69**

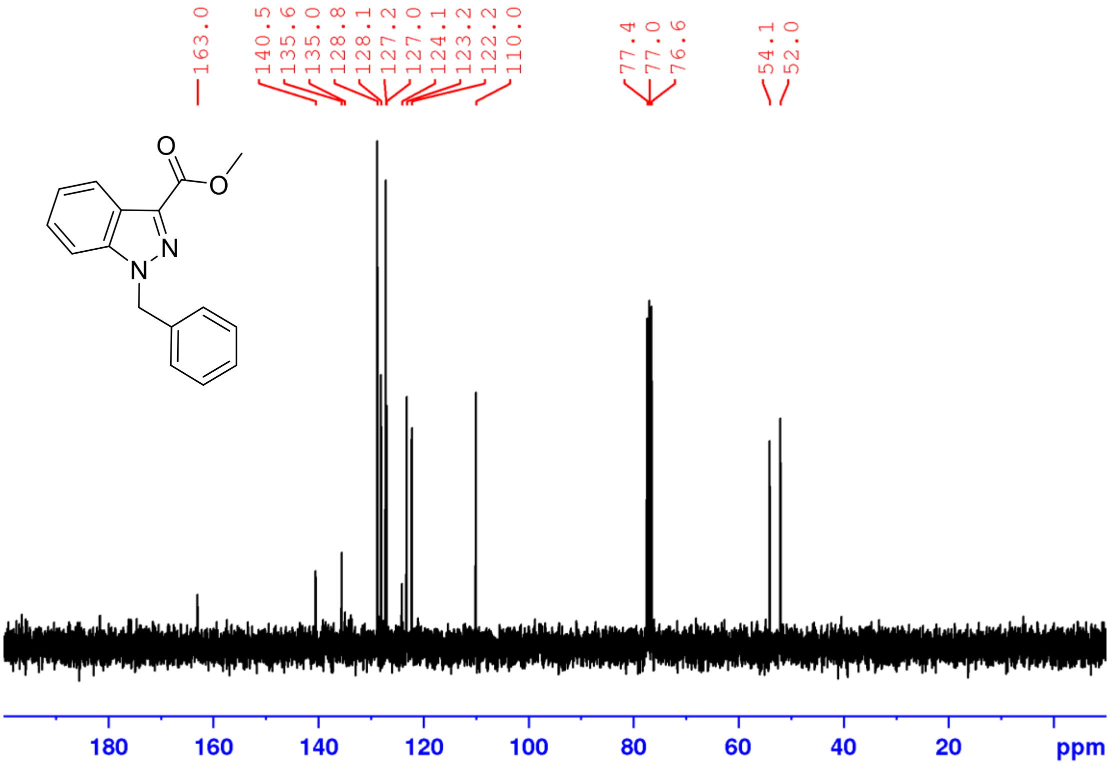

Methyl 2-benzyl-2*H*-indazole-3-carboxylate (70)

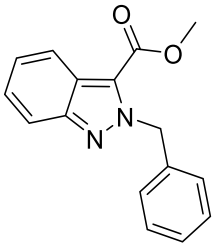

Following **General Procedure A** and/or **B**, wet flash column chromatography (EtOAc/hexane, 1:4) gave the title compound **70** (R_f = 0.60) as a colorless oil which solidified upon standing at room temperature: m.p. 74–76 °C; IR (ATR, cm^{-1}) ν_{max} 3065, 3034, 2954, 1713, 1466, 1278, 1210, 1088, 760, 707; ^1H NMR (300 MHz, $CDCl_3$) δ 8.02 (1H, d, J = 8.3 Hz), 7.82 (1H, dd, J = 8.5, 0.9 Hz), 7.38–7.21 (7H, m), 6.11 (2H, s), 3.99 (3H, s); ^{13}C NMR (75 MHz, $CDCl_3$) δ 160.6, 147.7, 136.4, 128.6, 128.0, 127.8, 126.5, 125.2, 123.7, 123.6, 121.5, 118.4, 56.6, 52.0; HRMS (ESI) *m/z*: $[M+H]^+$ Calcd for $C_{16}H_{15}N_2O_2$ 267.1128, found 267.1124 (– 1.5 ppm).

^{1}H NMR (300 MHz, $CDCl_3$) **70**

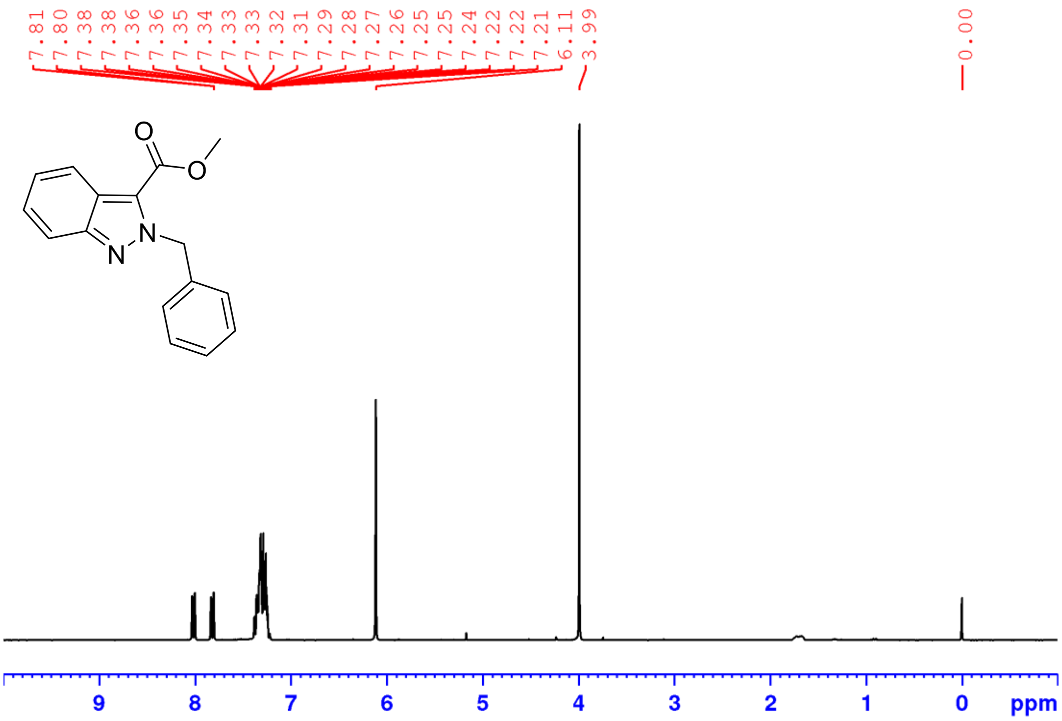

^{13}C NMR (75 MHz, $CDCl_3$) **70**

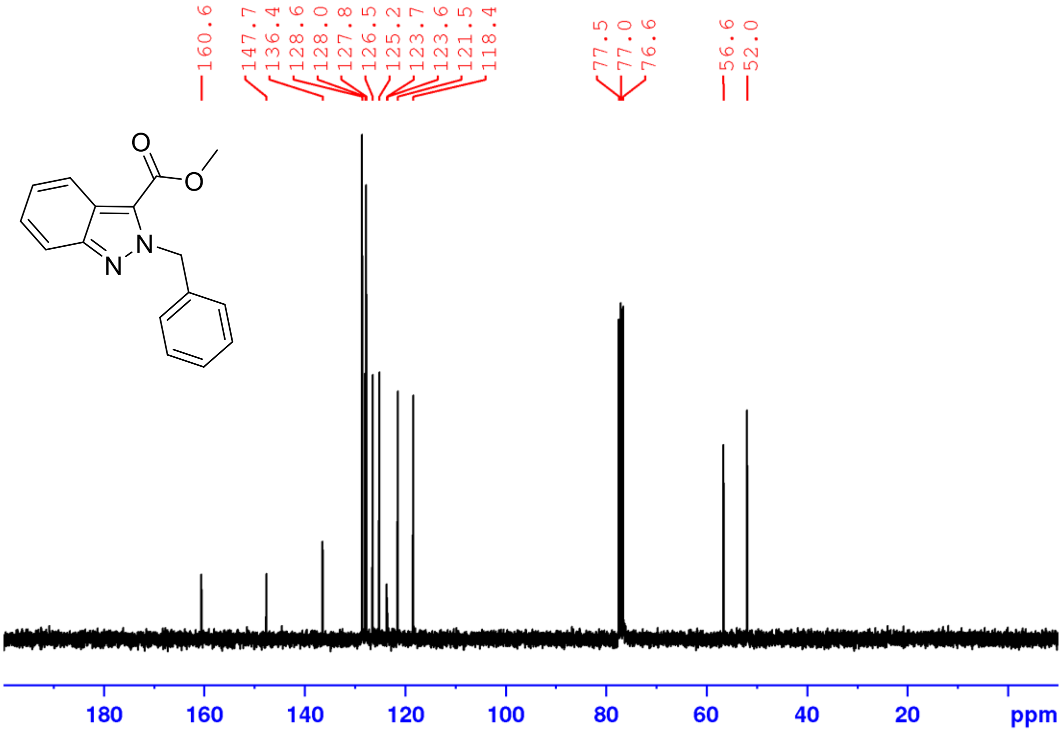

Methyl 1-(2-methylbenzyl)-1*H*-indazole-3-carboxylate (71)

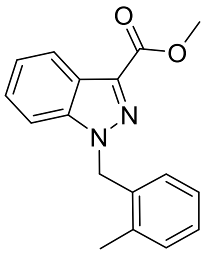

Following **General Procedure A** and/or **B**, wet flash column chromatography (EtOAc/hexane, 1:9) gave the title compound **71** (R_f = 0.17) as a colorless oil which solidified upon standing at room temperature: m.p. 85–86 °C; IR (ATR, cm^{-1}) ν_{max} 3013, 2953, 1713, 1479, 1232, 1160, 1125, 738, 431; ^{1}H NMR (300 MHz, $CDCl_3$) δ 8.27–8.24 (1H, m), 7.37–7.22 (3H, m), 7.20–7.15 (2H, m), 7.12–7.04 (1H, m), 6.78 (1H, d, *J* = 7.5 Hz), 5.71 (2H, s), 4.05 (3H, s), 2.35 (3H, s); ^{13}C NMR (75 MHz, $CDCl_3$) δ 160.1, 140.9, 135.8, 134.9, 133.6, 130.6, 128.1, 127.5, 127.0, 126.4, 124.1, 123.3, 122.3, 110.1, 52.5, 52.0, 19.3; HRMS (ESI) *m/z*: $[M+H]^+$ Calcd for $C_{17}H_{17}N_2O_2$ 281.1285, found 281.1283 (– 0.7 ppm).

^{1}H NMR (300 MHz, $CDCl_3$) **71**

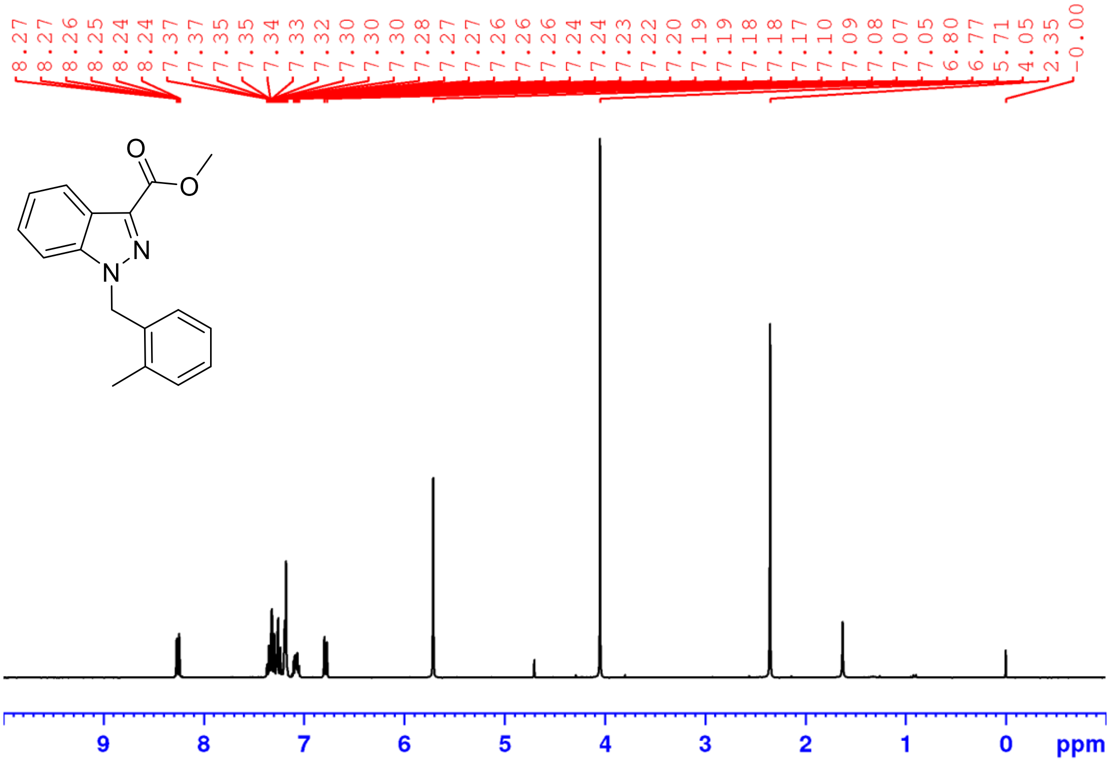

^{13}C NMR (75 MHz, $CDCl_3$) **71**

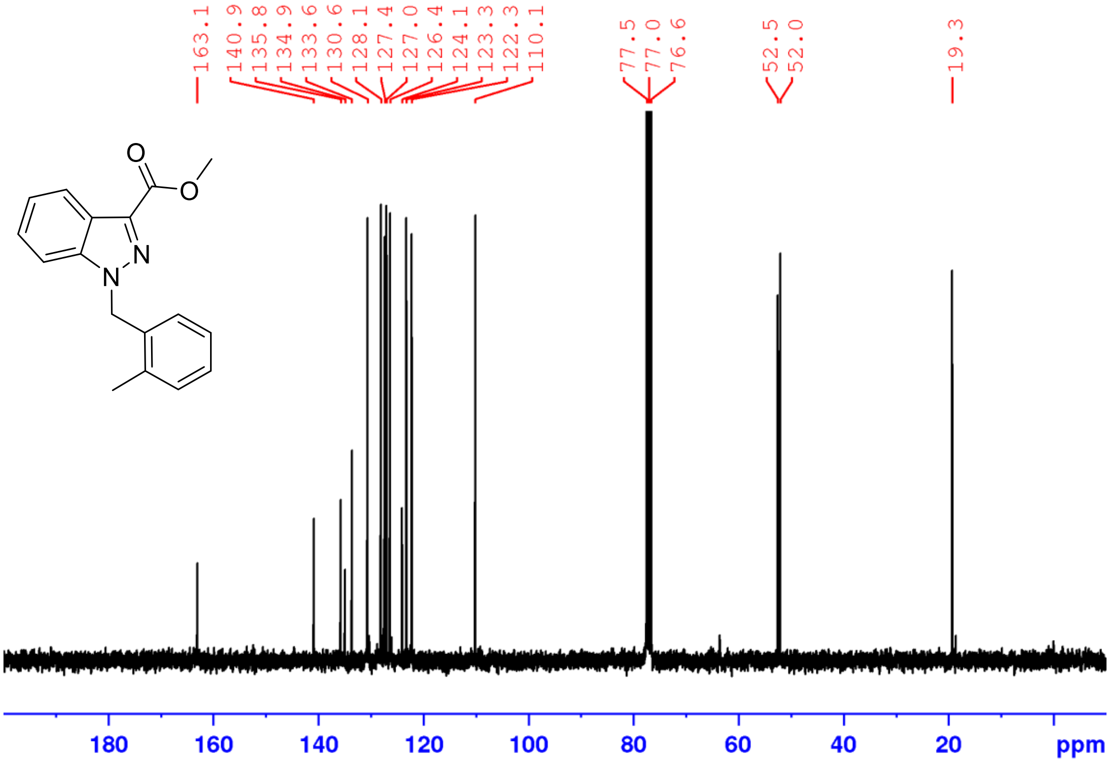

Methyl 2-(2-methylbenzyl)-2*H*-indazole-3-carboxylate (72)

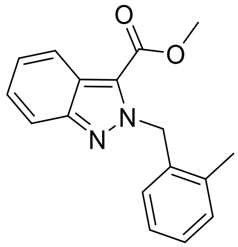

Following **General Procedure B**, wet flash column chromatography (EtOAc/hexane, 1:9) gave the title compound **72** (R_f = 0.37) as a colorless crystalline solid: m.p. 87 °C; IR (ATR, cm^{-1}) ν_{max} 3022, 2952, 1708, 1463, 1279, 1207, 1082, 757, 740, 725, 440; ^{1}H NMR (300 MHz, $CDCl_3$) δ 8.05 (1H, ddd, J = 8.2, 1.4, 1.1 Hz), 7.82 (1H, ddd, J = 8.4, 1.1, 0.8 Hz), 7.37 (1H, ddd, J = 8.1, 6.8, 1.3 Hz), 7.31 (1H, ddd, J = 8.3, 6.7, 1.3 Hz), 7.22–7.12 (2H, m), 7.03 (1H, dd, J = 7.4, 1.7 Hz), 6.55 (1H, d, J = 7.7 Hz), 6.13 (2H, s), 3.97 (3H, s), 2.45 (3H, s); ^{13}C NMR (75 MHz, $CDCl_3$) δ 160.6, 147.7, 135.3, 135.1, 130.2, 127.6, 126.4, 126.3, 126.2, 125.2, 124.1, 123.6, 121.5, 118.5, 54.5, 52.0, 19.3; HRMS (ESI) m/z: $[M+H]^+$ Calcd for $C_{17}H_{17}N_2O_2$ 281.1285, found 281.1284 (– 0.4 ppm).

^{1}H NMR (300 MHz, $CDCl_3$) **72**

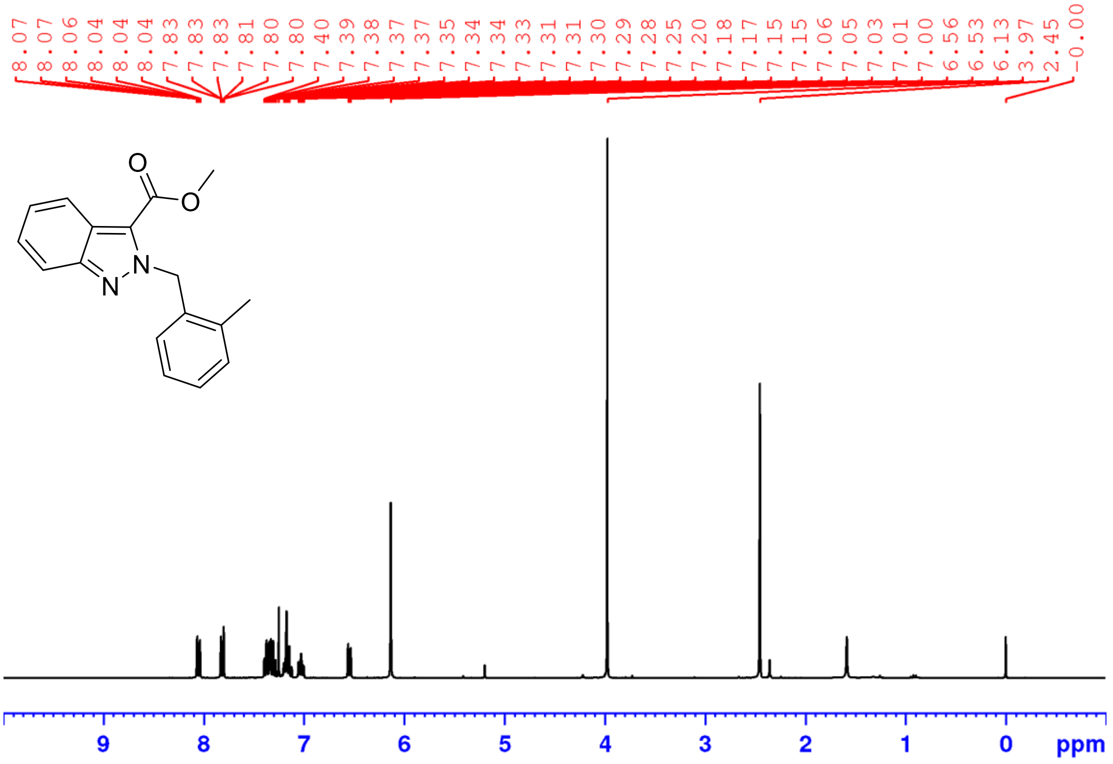

^{13}C NMR (75 MHz, $CDCl_3$) **72**

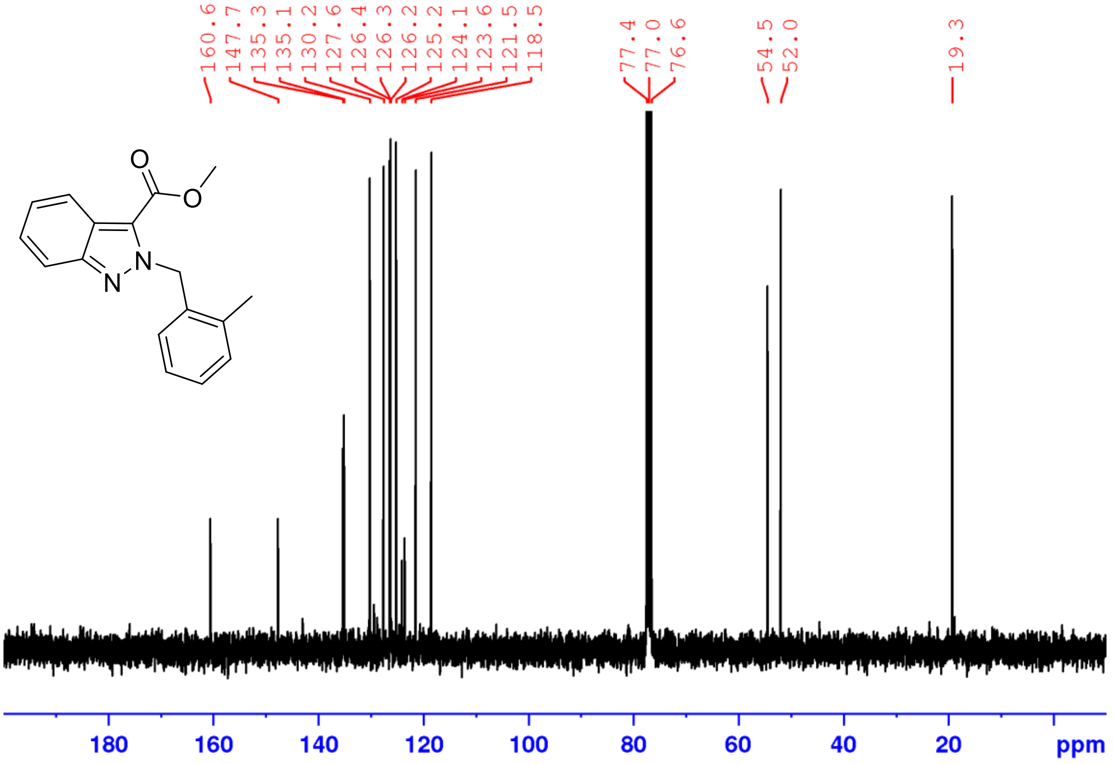

Methyl 1-(cyclohexylmethyl)-1*H*-indazole-3-carboxylate (73)

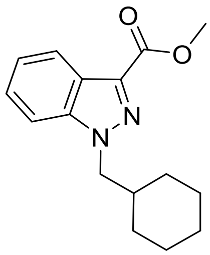

Following **General Procedure A** and/or **B**, wet flash column chromatography (EtOAc/hexane, 1:4) gave the title compound **73** (R_f = 0.47) as a yellow oil: IR (ATR, cm^{-1}) ν_{max} 2924, 2851, 1727, 1709, 1477, 1440, 1223, 1159, 1119, 790, 771, 750, 741; ^{1}H NMR (300 MHz, $CDCl_3$) δ 8.23 (1H, ddd, J = 8.2, 0.9, 0.8 Hz), 7.48–7.39 (2H, m), 7.30 (1H, ddd, J = 7.9, 6.2, 1.5 Hz), 4.28 (2H, d, J = 7.3 Hz), 4.04 (3H, s), 2.16–2.01 (1H , m), 1.72–1.55 (5H, m), 1.28–0.98 (5H, m); ^{13}C NMR (75 MHz, $CDCl_3$) δ 163.1, 141.1, 134.4, 126.6, 123.6, 122.9, 122.1, 109.8, 55.9, 51.9, 38.7, 30.8, 26.1, 25.5; HRMS (ESI) *m/z*: $[M+Na]^+$ Calcd for $C_{16}H_{20}N_2O_2Na$ 295.1417, found 295.1416 (– 0.3 ppm). Spectral data were in agreement with literature values [12].

^{1}H NMR (300 MHz, $CDCl_3$) **73**

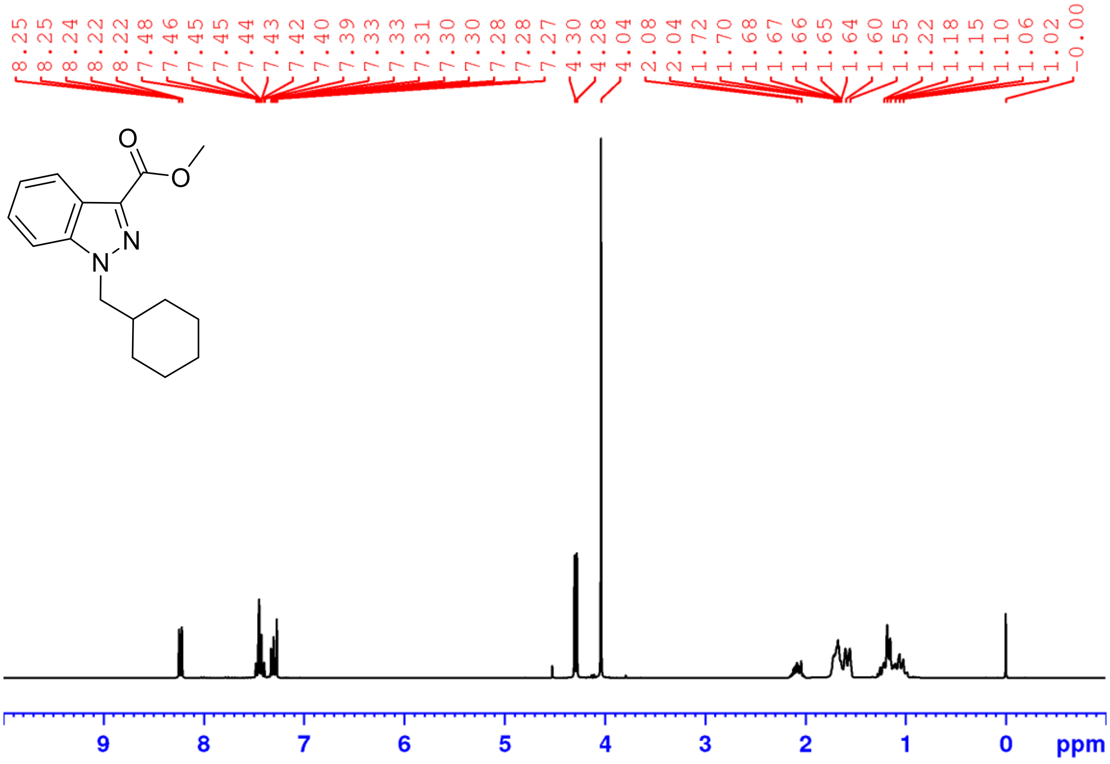

^{13}C NMR (75 MHz, $CDCl_3$) **73**

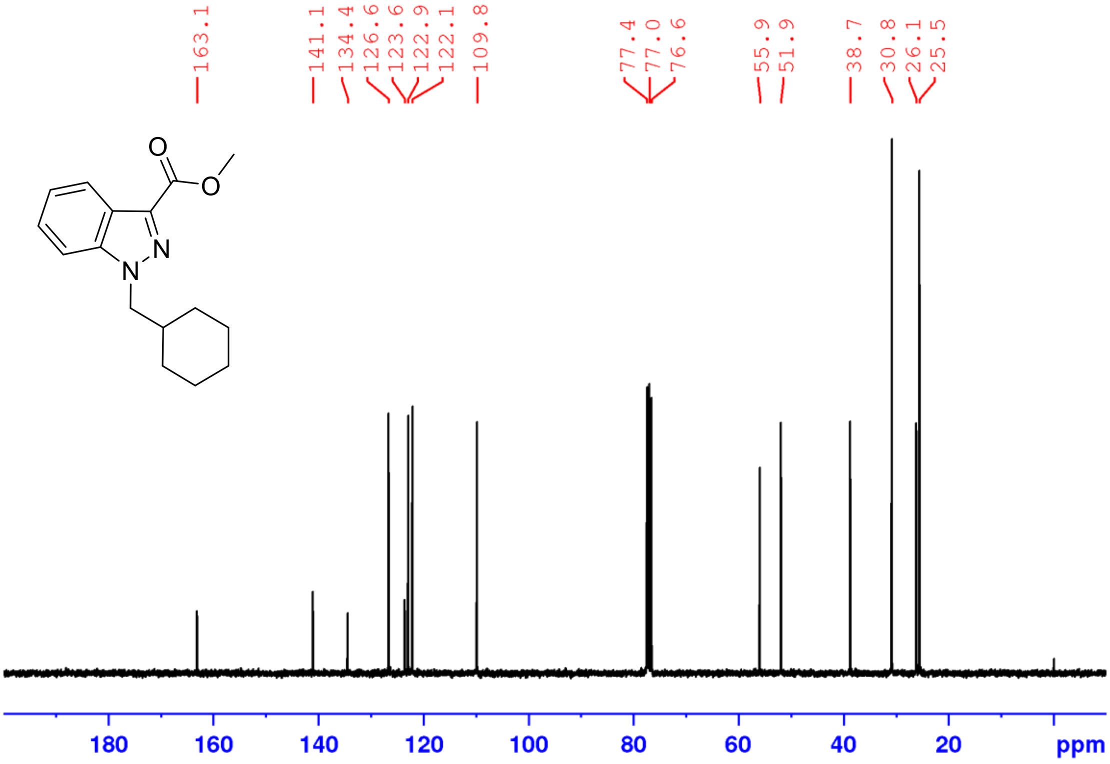

Methyl 2-(cyclohexylmethyl)-2*H*-indazole-3-carboxylate (74)

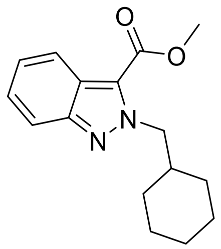

Following **General Procedure B**, wet flash column chromatography (EtOAc/hexane, 1:4) gave the title compound **74** (R_f = 0.67) as a yellow oil: IR (ATR, cm^{-1}) ν_{max} 2924, 2851, 1711, 1463, 1277, 1205, 1072, 779, 758, 739, 431; ^1H NMR (300 MHz, $CDCl_3$) δ 8.02 (1H, ddd, J = 8.3, 1.2, 1.0 Hz), 7.79 (1H, ddd, J = 8.4, 1.1, 1.0 Hz), 7.34 (1H, ddd, J = 8.0, 6.7, 1.2 Hz), 7.27 (1H, ddd, J = 8.3, 6.7, 1.2 Hz), 4.77 (2H, d, J = 7.3 Hz), 4.03 (3H, s), 2.13–2.01 (1H, m), 1.71–1.56 (5H, m), 1.32–1.04 (5H, m); ^{13}C NMR (75 MHz, $CDCl_3$) δ 160.8, 147.2, 126.2, 124.9, 123.8, 123.4, 121.4, 118.2, 59.2, 51.9, 39.5, 30.5, 26.3, 25.7; HRMS (ESI) m/z: $[M+H]^+$ Calcd for $C_{16}H_{21}N_2O_2$ 273.1598, found 273.1596 (– 0.7 ppm). Spectral data were in agreement with literature values [12].

^{1}H NMR (300 MHz, $CDCl_3$) **74**

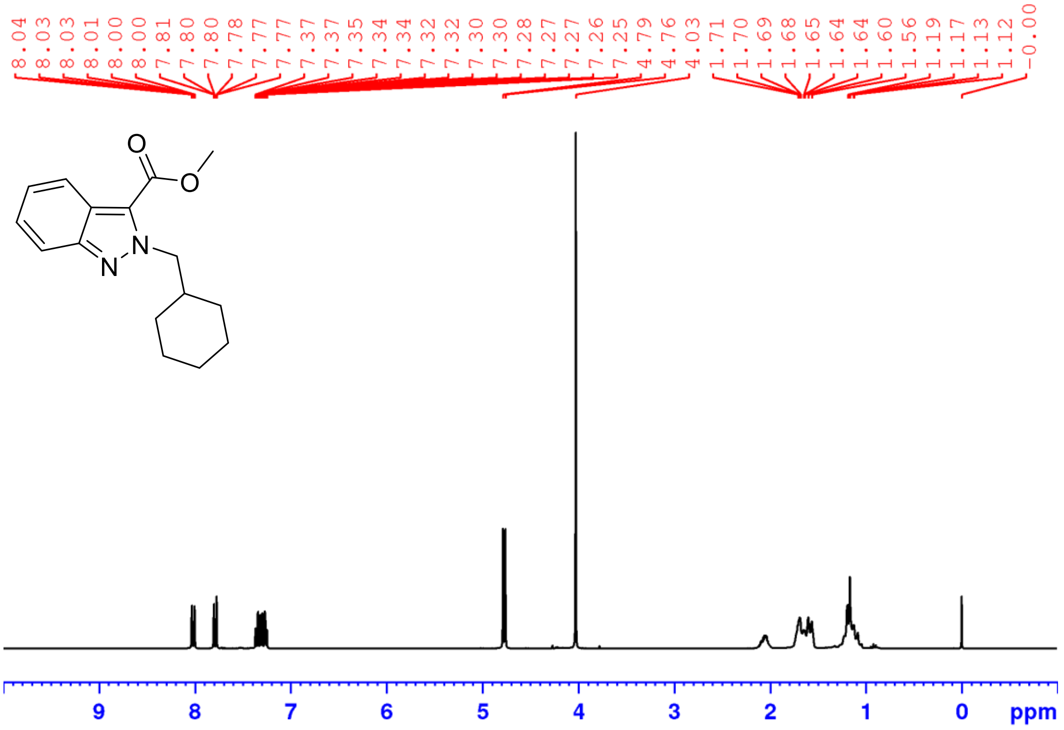

^{13}C NMR (75 MHz, $CDCl_3$) **74**

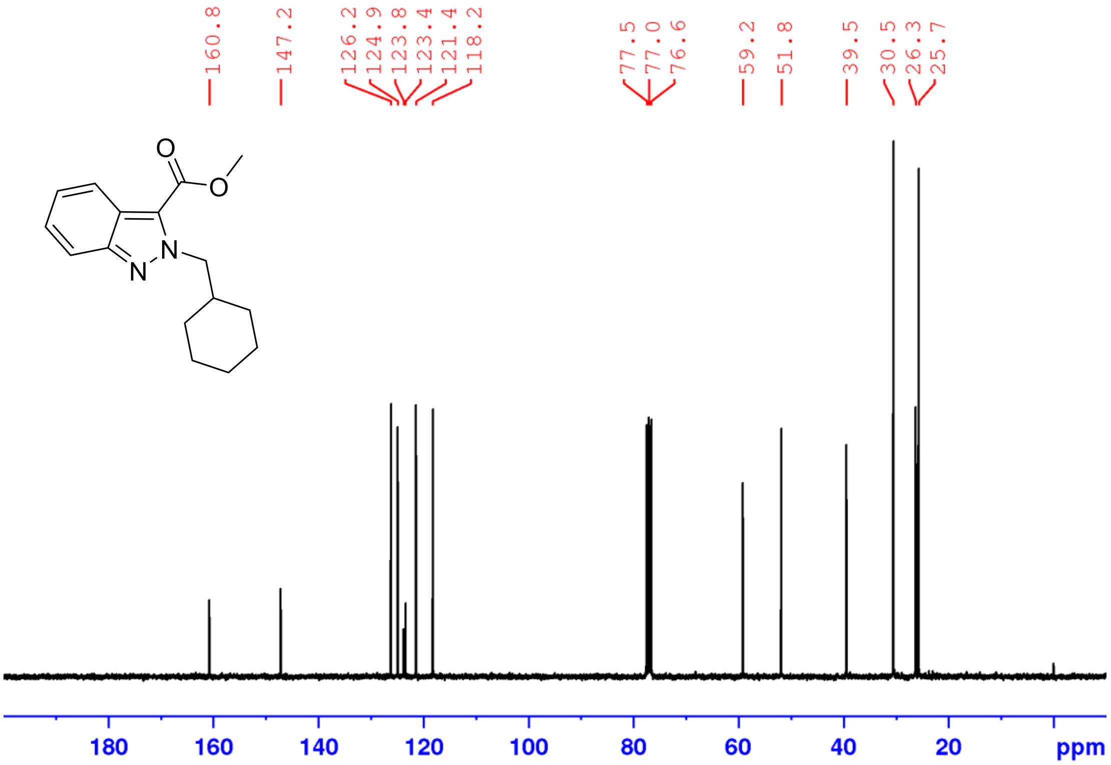

Methyl 1-(pentan-2-yl)-1*H*-indazole-3-carboxylate (75)

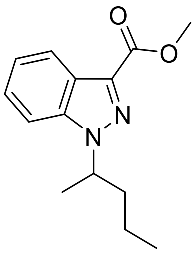

Following **General Procedure A** and/or **B**, wet flash column chromatography (EtOAc/hexane, 1:9) gave the title compound **75** (R_f = 0.27) as a colorless oil: IR (ATR, cm^{-1}) ν_{max} 3064, 2957, 2935, 2874, 1731, 1709, 1475, 1191, 1168, 1125, 752, 649, 433; ^{1}H NMR (300 MHz, $CDCl_3$) δ 8.24 (1H, d, J = 8.2 Hz), 7.51 (1H, d, J = 8.5 Hz), 7.41 (1H, ddd, J = 8.0, 6.8, 1.1 Hz), 7.30 (1H, ddd, J = 7.8, 6.8, 0.8 Hz), 4.78 (1H, tq, J = 8.6, 6.7 Hz), 4.40 (3H, s), 2.27–2.11 (1H, m), 1.95–1.82 (1H, m), 1.64 (3H, d, J = 6.9 Hz), 1.34–1.02 (2H, m), 0.87 (3H, t, J = 7.3 Hz); ^{13}C NMR (75 MHz, $CDCl_3$) δ 163.2, 140.2, 134.4, 126.4, 123.8, 122.9, 122.2, 109.7, 56.0, 51.8, 38.3, 20.5, 19.6, 13.6; HRMS (ESI) *m/z*: $[M+H]^+$ Calcd for $C_{14}H_{19}N_2O_2$ 247.1441, found 247.1453 (4.9 ppm).

^{1}H NMR (300 MHz, $CDCl_3$) **75**

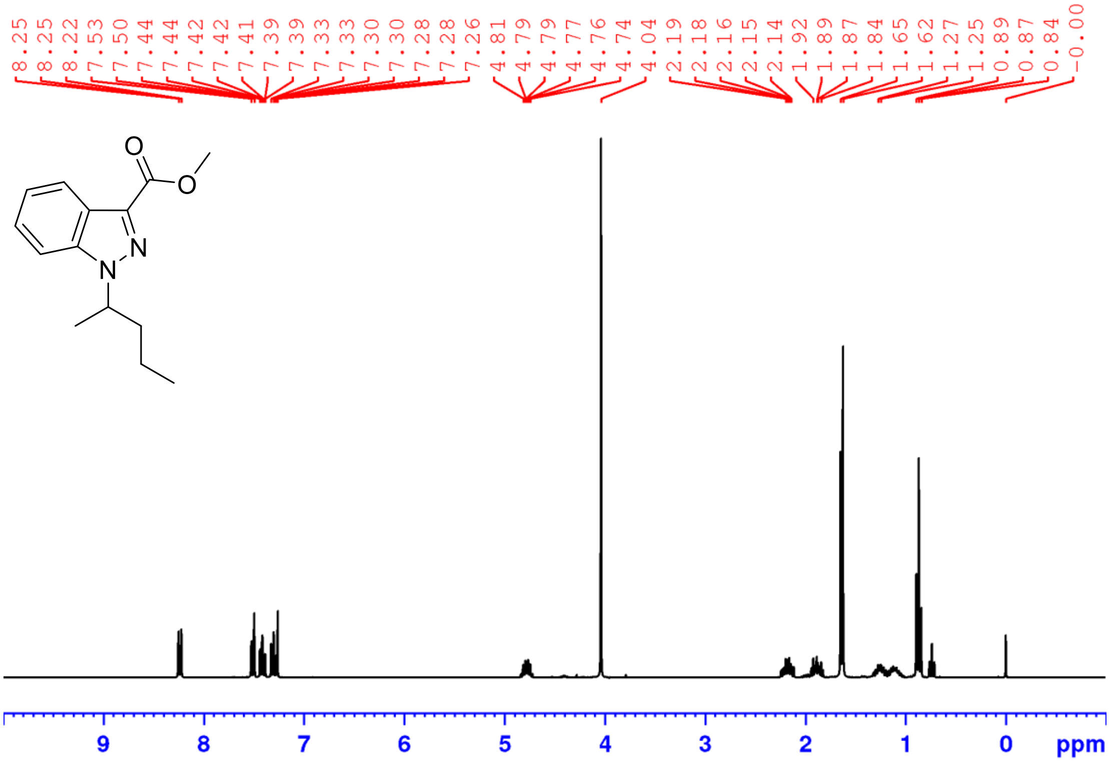

^{13}C NMR (75 MHz, $CDCl_3$) **75**

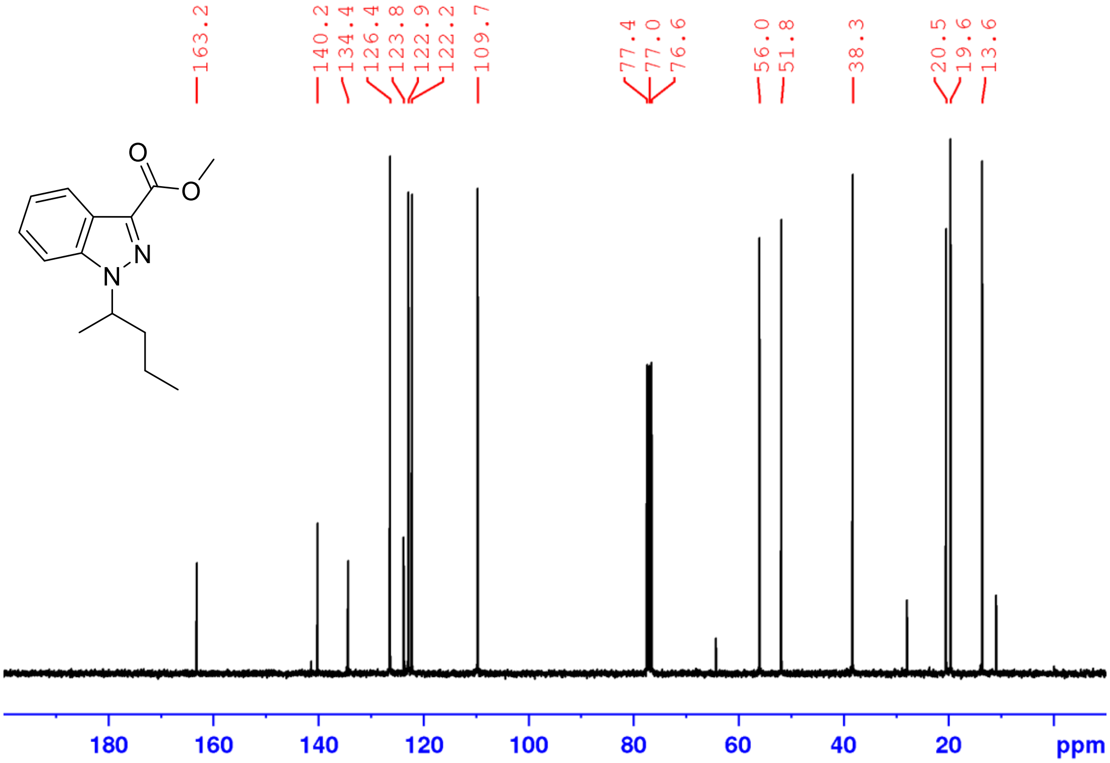

Methyl 2-(pentan-2-yl)-2*H*-indazole-3-carboxylate (76)

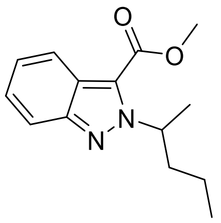

Following **General Procedure A** and/or **B**, wet flash column chromatography (EtOAc/hexane, 1:9) gave the title compound **76** (R_f = 0.50) as a colorless oil: IR (ATR, cm^{-1}) ν_{max} 2957, 2934, 2873, 1709, 1455, 1270, 1202, 1079, 759; ^{1}H NMR (300 MHz, $CDCl_3$) δ 8.02 (1H, ddd, *J* = 8.4, 1.3, 1.1 Hz), 7.82 (1H, ddd, *J* = 8.5, 1.2, 1.0 Hz), 7.33 (1H, ddd, *J* = 8.2, 6.7, 1.3 Hz), 7.26 (1H, ddd, *J* = 7.9, 6.7, 1.1 Hz), 5.91 (1H, tq, *J* = 8.5, 6.6 Hz), 4.02 (3H, s), 2.24–2.07 (1H, m), 1.90 – 1.80 (1H, m), 1.62 (3H, d, *J* = 6.6 Hz), 1.36–1.01 (2H, m), 0.88 (3H, t, *J* = 7.3 Hz); ^{13}C NMR (75 MHz, $CDCl_3$) δ 160.9, 147.4, 125.9, 124.7, 123.6, 123.2, 121.4, 118.3, 57.3, 51.7, 39.2, 21.5, 19.3, 13.7; HRMS (ESI) *m/z*: $[M+H]^+$ Calcd for $C_{14}H_{19}N_2O_2$ 247.1441, found 247.1440 (– 0.4 ppm).

^{1}H NMR (300 MHz, CDCl$_3$) **76**

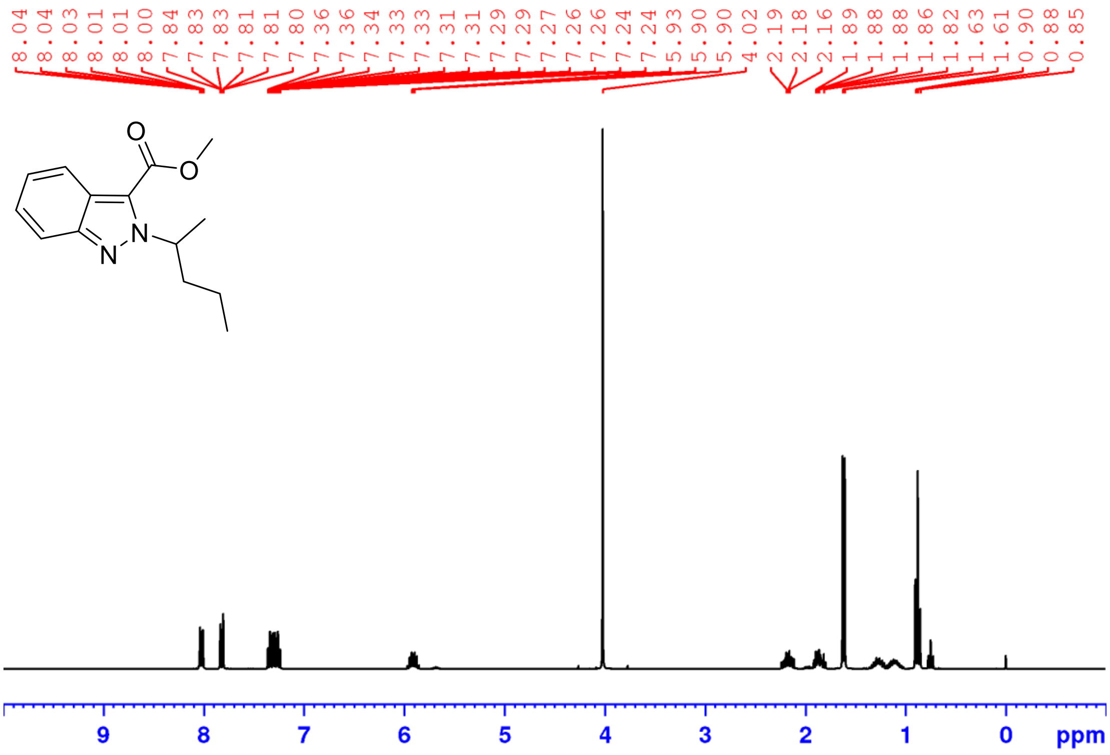

^{13}C NMR (75 MHz, CDCl$_3$) **76**

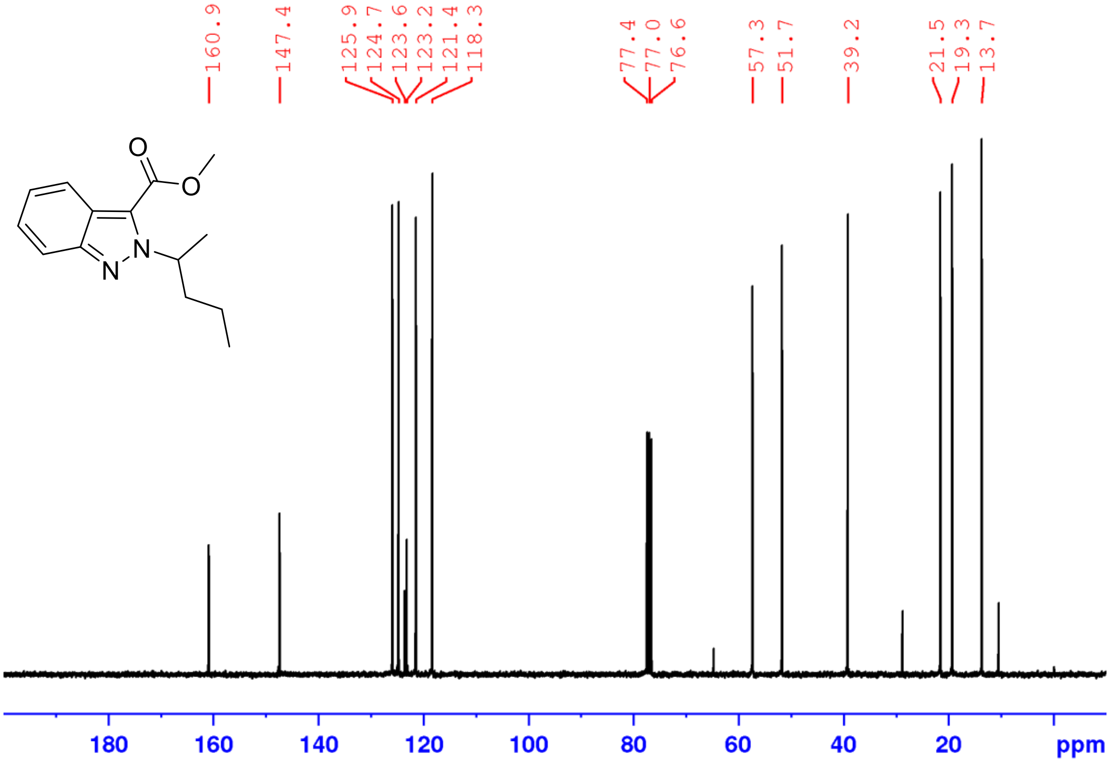

Methyl 1-(pentan-3-yl)-1*H*-indazole-3-carboxylate (77)

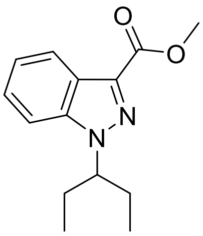

Following **General Procedure A** and/or **B**, wet flash column chromatography (EtOAc/hexane, 1:9) gave the title compound **77** (R_f = 0.27) as a colorless oil: IR (ATR, cm^{-1}) v_{max} 3058, 2967, 2934, 2877, 1710, 1475, 1439, 1407, 1244, 1191, 1165, 1125, 1084, 1008, 749, 432; ^{1}H NMR (300 MHz, $CDCl_3$) δ 8.24 (1H, ddd, *J* = 8.2, 1.0, 1.0 Hz), 7.51 (1H, d, *J* = 8.5 Hz), 7.41 (1H, ddd, *J* = 8.0, 6.8, 1.2 Hz), 7.30 (1H, ddd, *J* = 7.9, 6.8, 0.9 Hz), 4.41 (1H, sept., *J* = 4.8 Hz), 4.04 (3H, s), 2.27–2.12 (2H, m), 2.04–1.90 (2H, m), 0.74 (6H, t, *J* = 7.4 Hz); ^{13}C NMR (100 MHz, $CDCl_3$) δ 163.4, 141.5, 134.7, 126.5, 123.7, 122.9, 122.3, 109.8, 64.4, 52.0, 28.0, 11.0; HRMS (ESI) *m/z*: $[M+H]^+$ Calcd for $C_{14}H_{19}N_2O_2$ 247.1441, found 247.1444 (1.2 ppm).

^{1}H NMR (300 MHz, $CDCl_3$) **77**

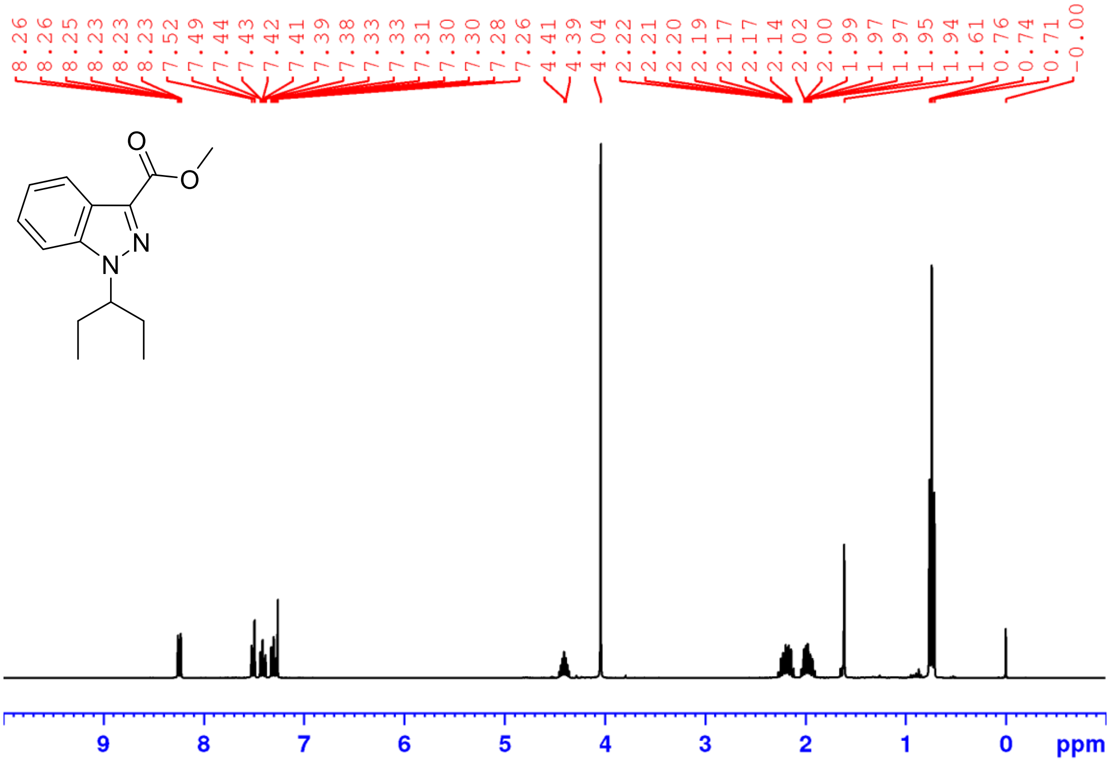

^{13}C NMR (75 MHz, $CDCl_3$) **77**

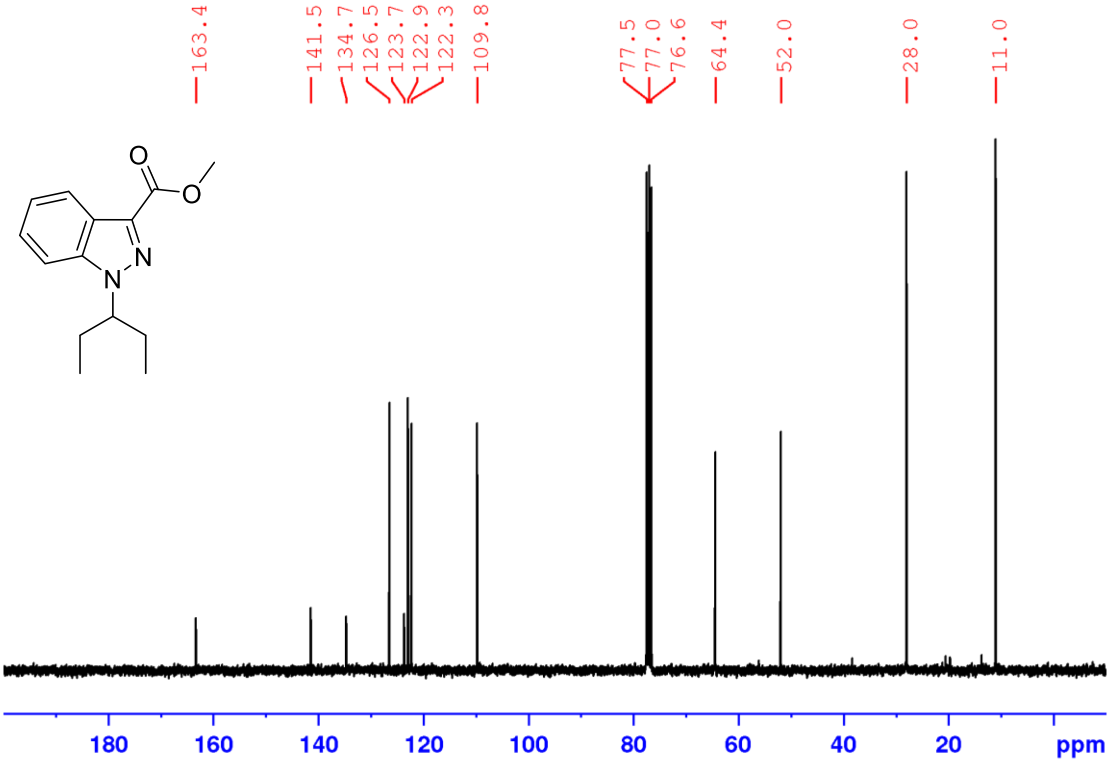

Methyl 2-(pentan-3-yl)-2*H*-indazole-3-carboxylate (78)

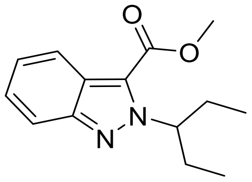

Following **General Procedure B**, wet flash column chromatography (EtOAc/hexane, 1:9) gave the title compound **78** (R_f = 0.50) as a colorless oil: IR (ATR, cm^{-1}) v_{max} 2967, 2929, 2876, 1712, 1455, 1361, 1267, 1202, 1080, 756, 743, 439; ^{1}H NMR (300 MHz, $CDCl_3$) δ 8.04 (1H, ddd, J = 8.3, 1.2, 1.0 Hz), 7.82 (1H, ddd, J = 8.5, 1.1, 0.9 Hz), 7.35 (1H, ddd, J = 8.0, 6.7, 1.3 Hz), 7.28 (1H, ddd, J = 7.8, 6.7, 1.1 Hz), 5.73–5.63 (1H, m), 4.03 (3H, s), 2.23–2.08 (2H, m), 2.03–1.89 (2H, m), 0.75 (6H, t, J = 7.4 Hz); ^{13}C NMR (75 MHz, $CDCl_3$) δ 161.1, 177.7, 126.0, 125.0, 124.8, 123.0, 121.6, 118.3, 64.8, 51.8, 28.8, 10.5; HRMS (ESI) *m/z*: $[M+H]^+$ Calcd for $C_{14}H_{19}N_2O_2$ 247.1441, found 247.1444 (1.2 ppm).

^{1}H NMR (300 MHz, $CDCl_3$) **78**

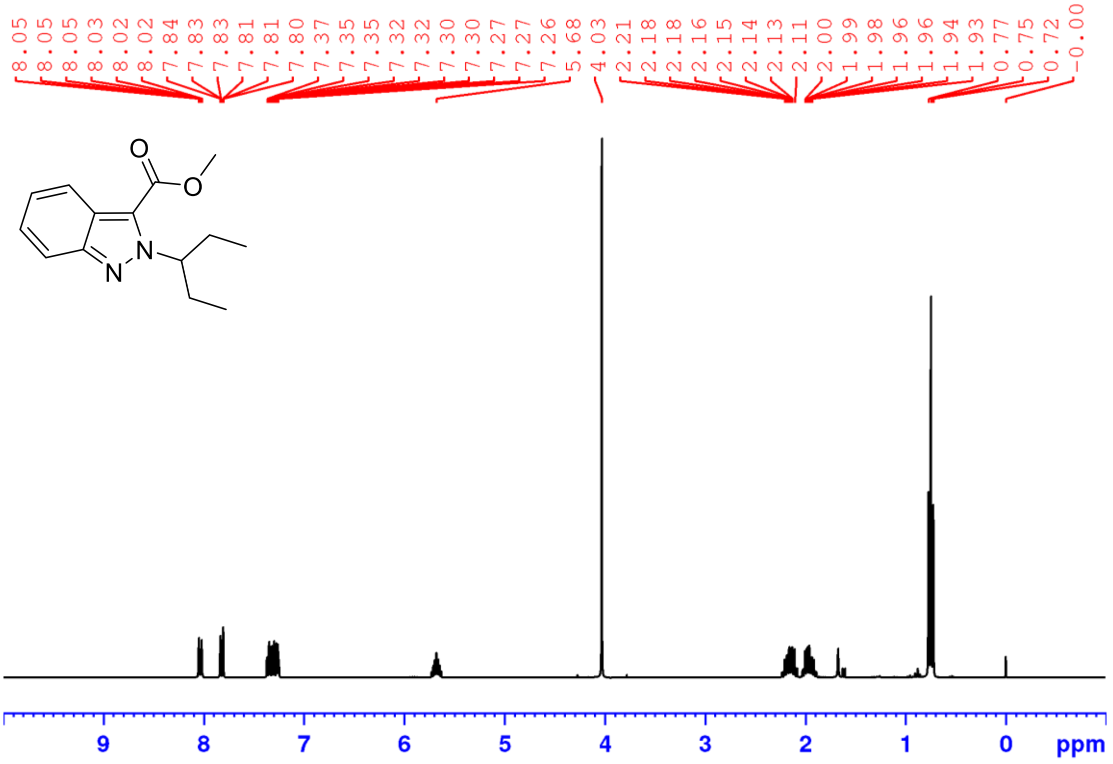

^{13}C NMR (75 MHz, $CDCl_3$) **78**

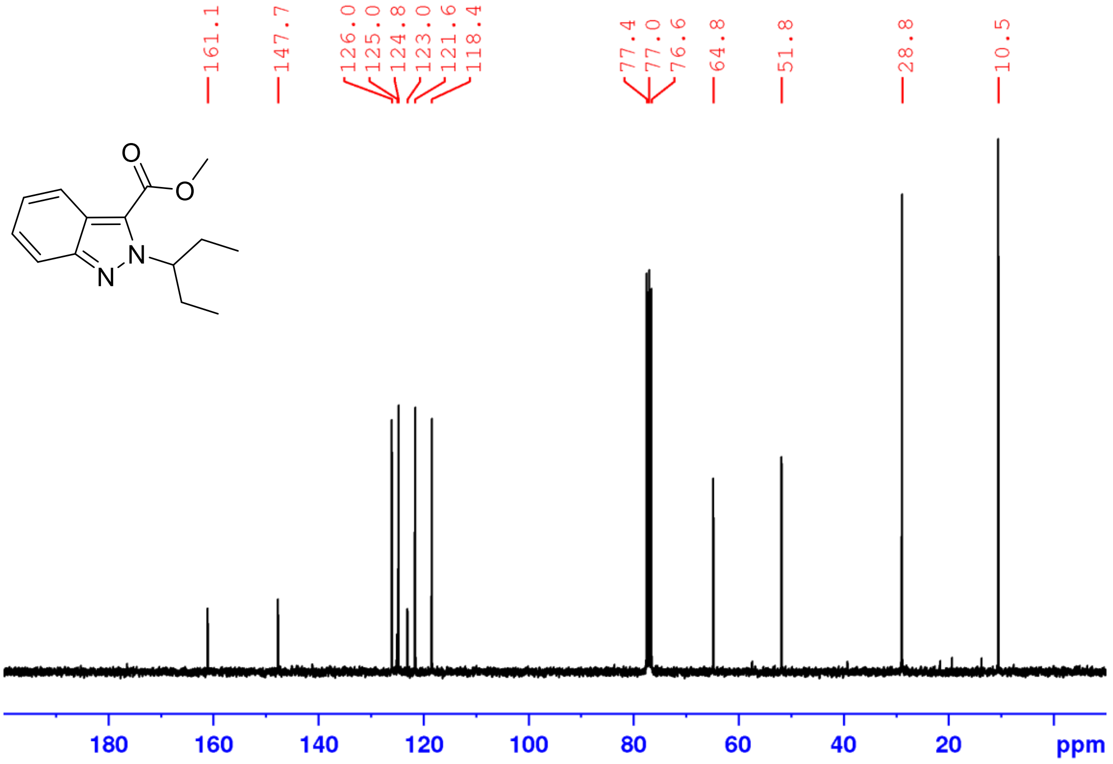

Synthesis of Tosylates

General Procedure (C):

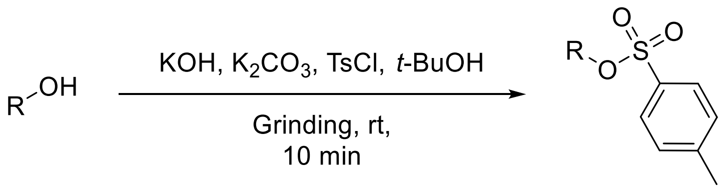

Following a previously reported method [15], to a glass mortar was added freshly ground KOH (5 equiv.) and alkyl alcohol (1 equiv). The mixture was vigorously ground for 5 min in a glass mortar and pestle (exposed to air) at room temperature. The resulting paste was then mixed with K_2CO_3 (3.6 equiv), TsCl (1.5 equiv), and ground for a further 3 min at room temperature. The reaction mixture was then treated with freshly ground KOH (5 equiv) and three drops of *t*-BuOH and ground for a further 2 min. The crude paste was then extracted with Et_2O and the resulting organic phase dried under reduced pressure to afford the crude tosylate. When required, further purification of the crude material using recrystallization or wet flash column chromatography gave the desired tosylate.

n-Pentyl 4-methylbenzenesulfonate

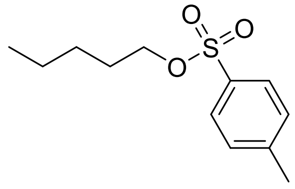

Following **General Procedure C** (employing *n*-pentanol as the alcohol), wet flash column chromatography (EtOAc/hexane, 1:4) gave the title compound (R_f = 0.67) as a colorless oil (885 mg, 73%): IR (ATR, cm^{-1}) ν_{max} 2958, 2932, 2872, 1598, 1356, 1188, 1174, 1097, 957, 910, 813, 662, 553; ^{1}H NMR (300 MHz, $CDCl_3$) δ 7.79 (2H, ddd, J = 8.3, 1.9, 1.9 Hz), 7.34 (2H, dd, J = 8.5, 0.6 Hz), 4.02 (2H, t, J = 6.5 Hz), 2.45 (3H, s), 1.64 (2H, quint, J = 6.9 Hz), 1.36–1.19 (4H, m), 0.85 (3H, t, J = 7.1 Hz); ^{13}C NMR (75 MHz, $CDCl_3$) δ 144.6, 133.3, 129.8, 127.9, 70.7, 28.5, 27.4, 22.0, 21.6, 13.8; HRMS (ESI) *m/z*: $[M+Na]^+$ Calcd for $C_{12}H_{18}O_3SNa$ 265.0869, found 265.0875 (2.3 ppm)

^{1}H NMR (300 MHz, $CDCl_3$)

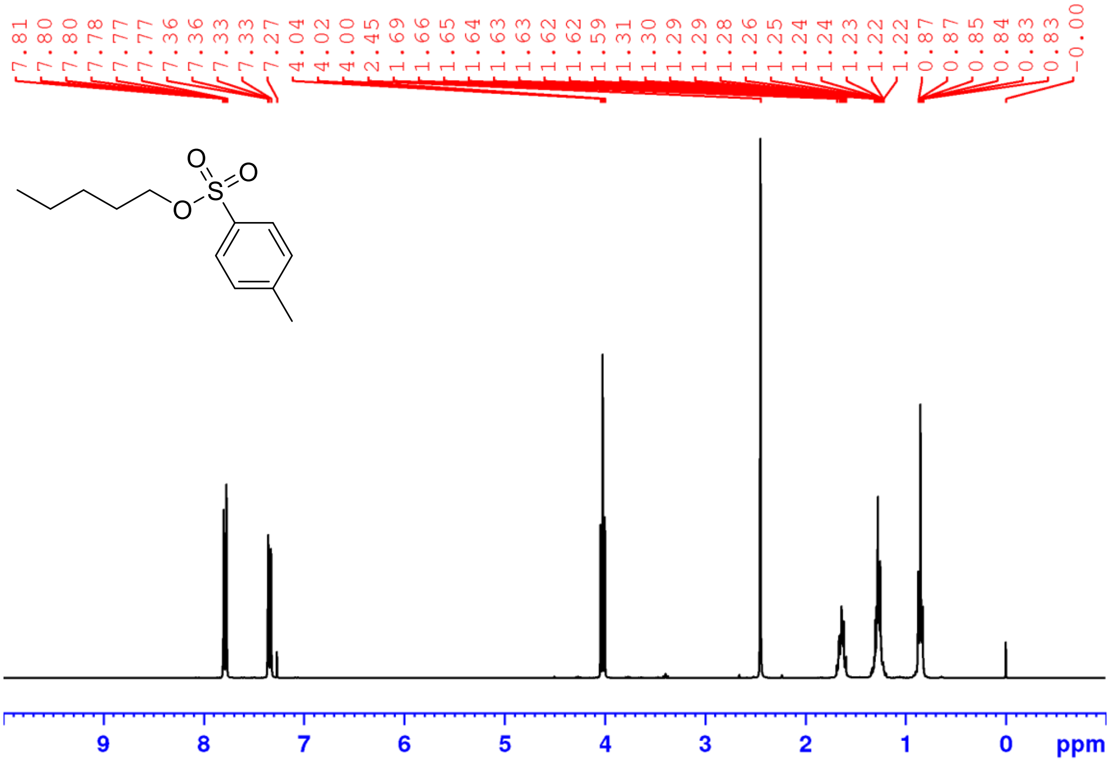

^{13}C NMR (75 MHz, $CDCl_3$)

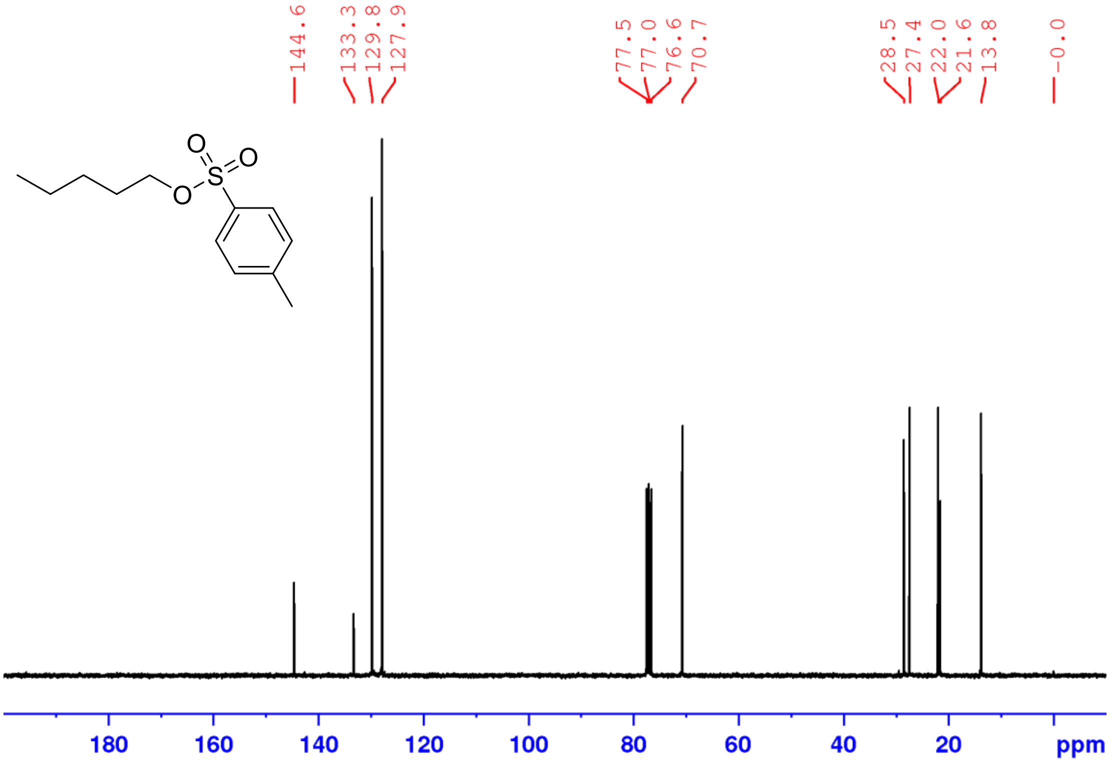

Benzyl 4-methylbenzenesulfonate

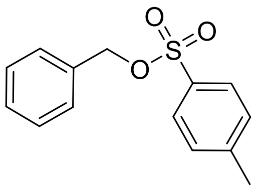

Following **General Procedure C** (employing benzyl alcohol as the alcohol), recrystallization from Et_2O/hexane (1:3) gave the title compound as colorless needles (1.471 g, 56%): m.p. 56–57 °C [Et_2O/hexane] (lit. m.p. 57–58 °C [Et_2O])[16]; IR (ATR, cm^{-1}) ν_{max} 3033, 2976, 1596, 1455, 1345, 1167, 908, 660, 553; ^1H NMR (300 MHz, $CDCl_3$) δ 7.79 (2H, ddd, J = 8.3, 1.7, 1.7 Hz), 7.34–7.29 (5H, m), 7.27–7.21 (2H, m), 5.05 (2H, s), 2.44 (3H, s); ^{13}C NMR (75 MHz, $CDCl_3$) δ 144.8, 133.3, 129.8, 129.0, 128.7, 128.5, 128.0, 71.9, 21.6; HRMS (ESI) m/z: $[M+Na]^+$ Calcd for $C_{14}H_{14}O_3SNa$ 285.0556, found 285.0551 (– 1.8 ppm). Spectral data were in agreement with literature values [16].

^{1}H NMR (300 MHz, $CDCl_3$)

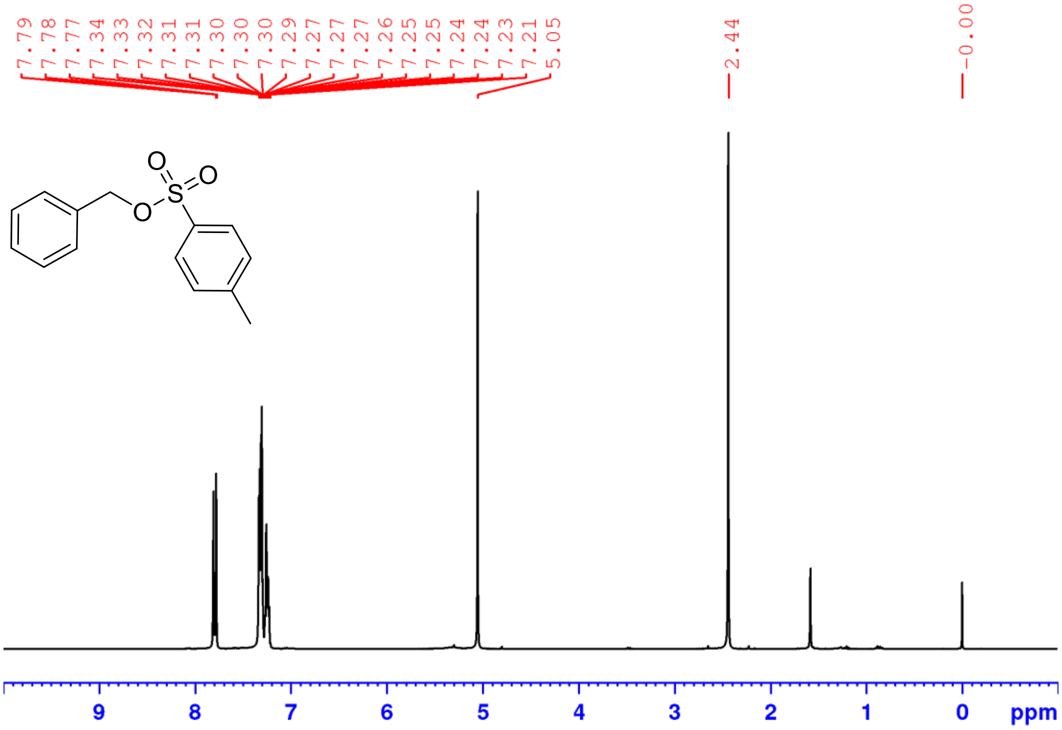

^{13}C NMR (75 MHz, $CDCl_3$) X116902

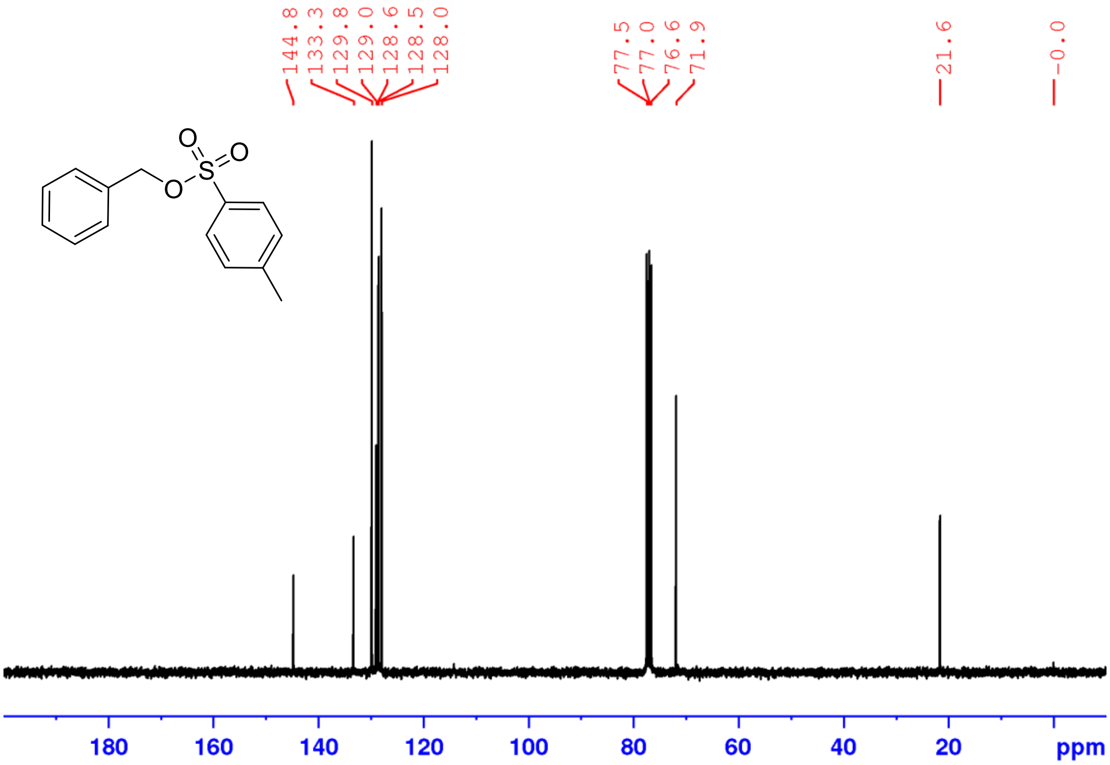

Cyclohexylmethyl 4-methylbenzenesulfonate

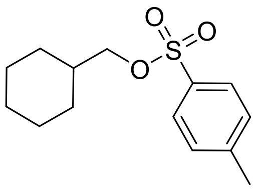

Following **General Procedure C** (employing cyclohexylmethanol as the alcohol), wet flash column chromatography (EtOAc/hexane, 1:4) gave the title compound (R_f = 0.67) as a colorless oil (1.224 g, 46%): IR (ATR, cm^{-1}) ν_{max} 2925, 2853, 1599, 1450, 1358, 1173, 1098, 971, 812, 664, 553; ^{1}H NMR (300 MHz, $CDCl_3$) δ 7.78 (2H, ddd, J = 8.4, 1.8, 1.8 Hz), 7.34 (2H, d, J = 8.0 Hz), 3.81 (2H, d, J = 6.1 Hz), 2.45 (3H, s), 1.70–1.59 (6H, m), 1.27–1.04 (3H, m), 0.95–0.84 (2H, m); ^{13}C NMR (75 MHz, $CDCl_3$) δ 144.6, 133.2, 129.8, 127.9, 75.4, 37.2, 29.1, 26.1, 25.4, 21.6; HRMS (ESI) m/z: $[M+Na]^+$ Calcd for $C_{14}H_{20}O_3SNa$ 291.1025, found 291.1025 (0.0 ppm). Spectral data were in agreement with literature values [17].

^{1}H NMR (300 MHz, $CDCl_3$)

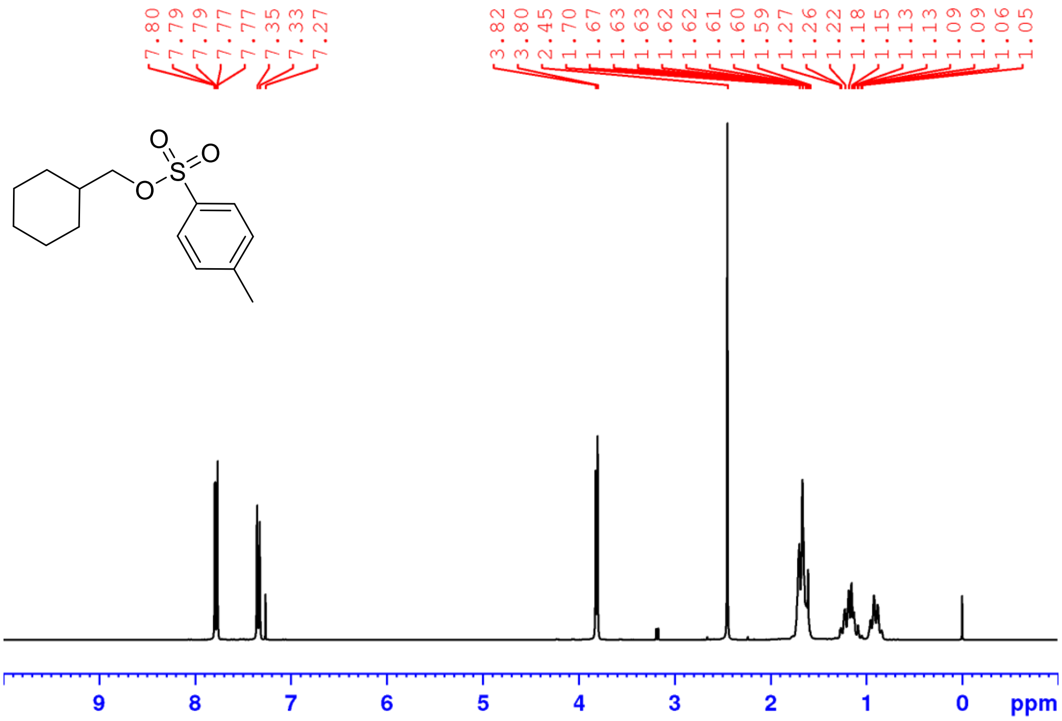

^{13}C NMR (75 MHz, $CDCl_3$)

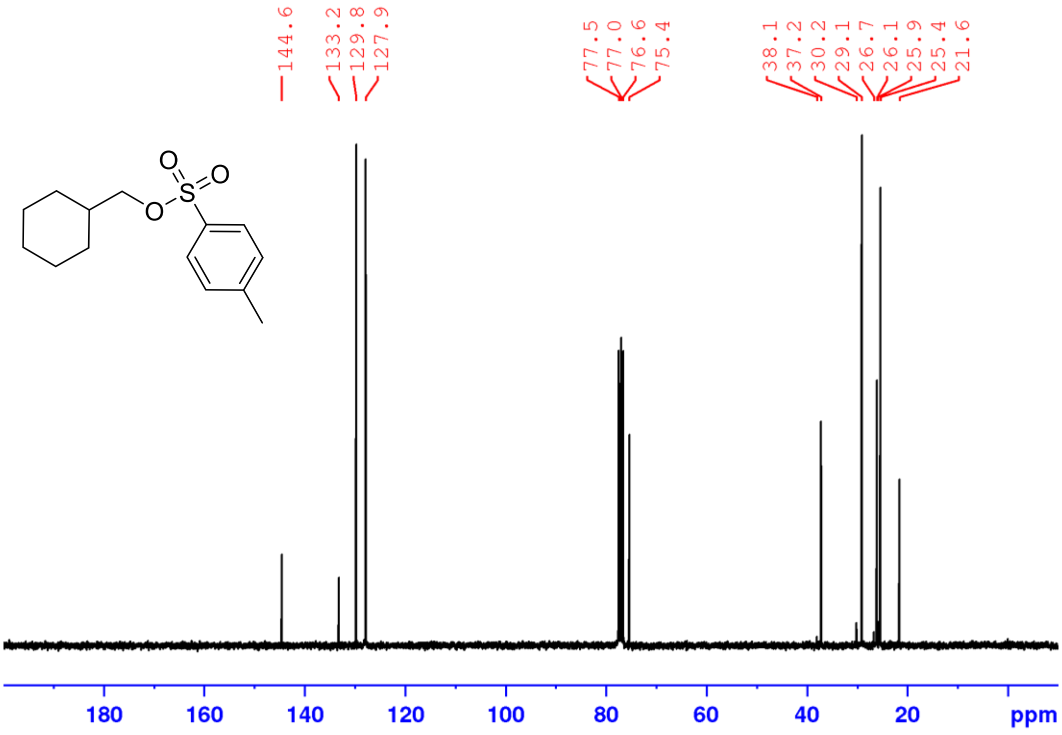

Pentan-2-yl 4-methylbenzenesulfonate

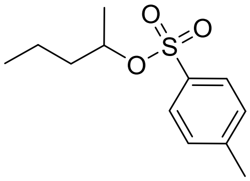

Following **General Procedure C** (employing pentan-2-ol as the alcohol), the title compound as obtained as a colorless oil (816 mg, 30%): IR (ATR, cm^{-1}) ν_{max} 2961, 2936, 2875, 1599, 1350, 1188, 1174, 1094, 894, 815, 774, 662, 575, 554; ^{1}H NMR (300 MHz, $CDCl_3$) δ 7.79 (2H, d, J = 8.2 Hz), 7.34–7.28 (2H, m), 4.63 (1H, appsext, J = 6.3 Hz), 2.44 (3H, s), 1.67–1.39 (2H, m), 1.36–1.13 (5H, m), 0.82 (3H, t, J = 7.3 Hz); ^{13}C NMR (75 MHz, $CDCl_3$) δ 144.3, 134.6, 129.6, 127.6, 80.3, 38.5, 21.5, 20.7, 18.1, 13.5; HRMS (ESI) m/z: $[M+Na]^+$ Calcd for $C_{12}H_{18}O_3SNa$ 265.0869, found 265.0867 (− 0.8 ppm). Spectral data were in agreement with literature values [18].

^{1}H NMR (300 MHz, $CDCl_3$)

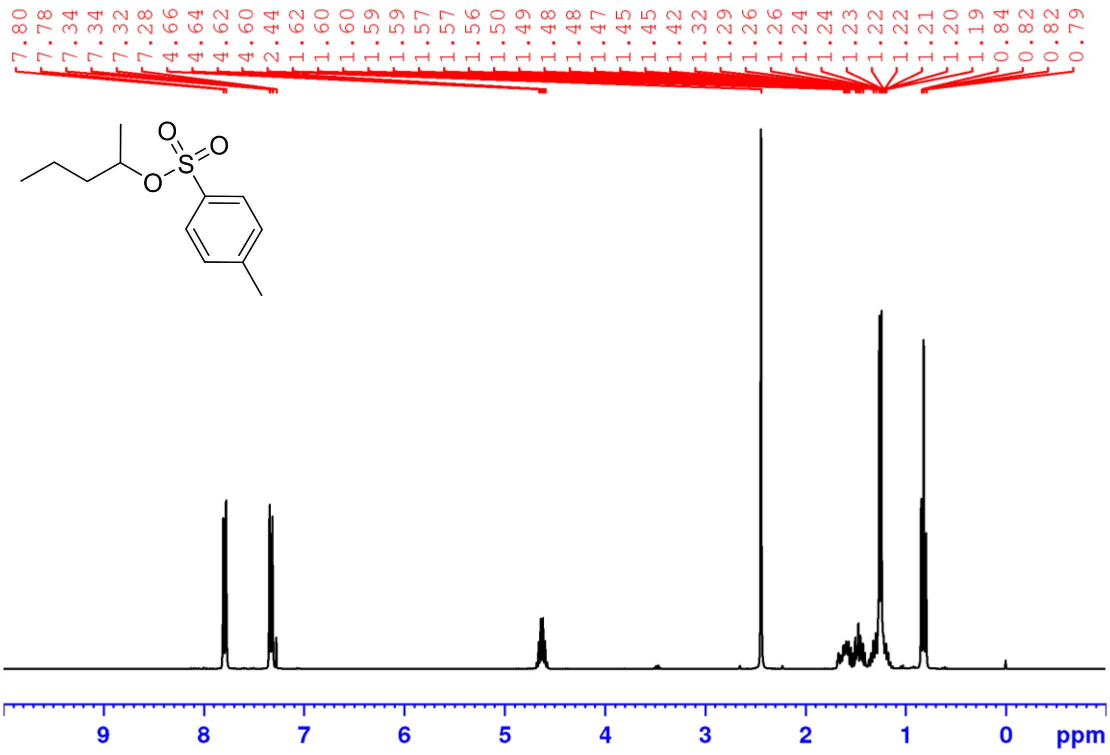

^{13}C NMR (75 MHz, $CDCl_3$)

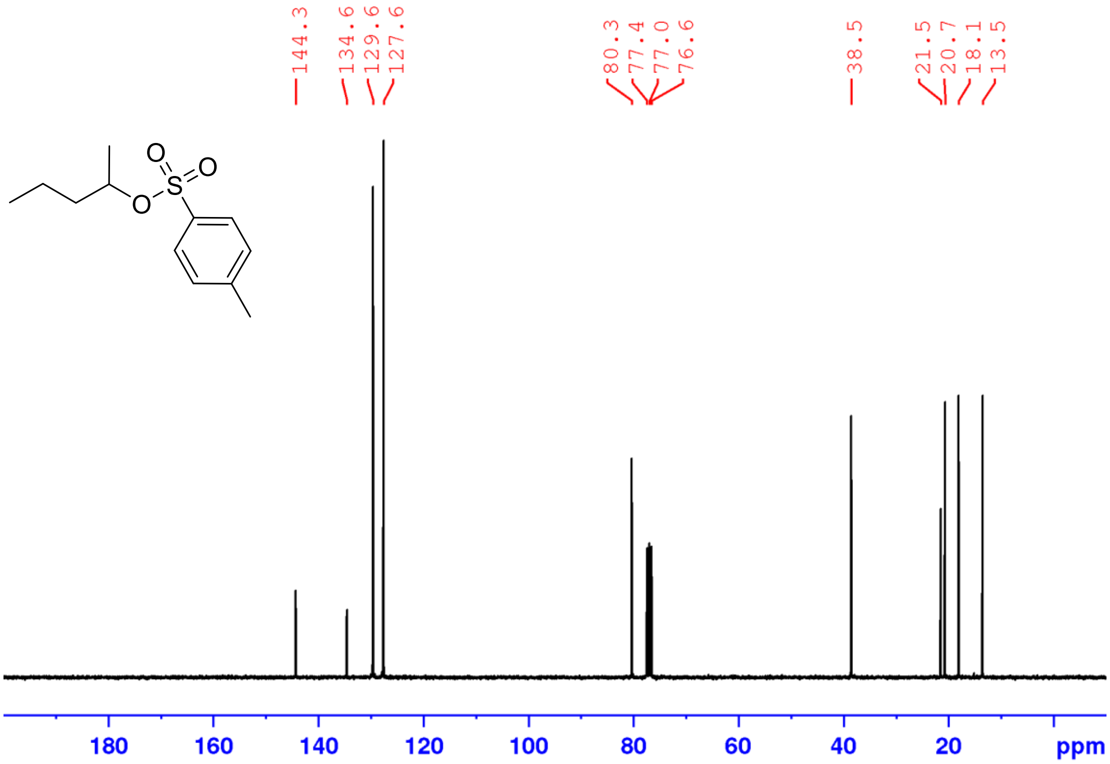

Regioisomeric distribution (*N*-1:*N*-2) determination; Crude ^{1}H NMR spectra

Table 1, Entry 3

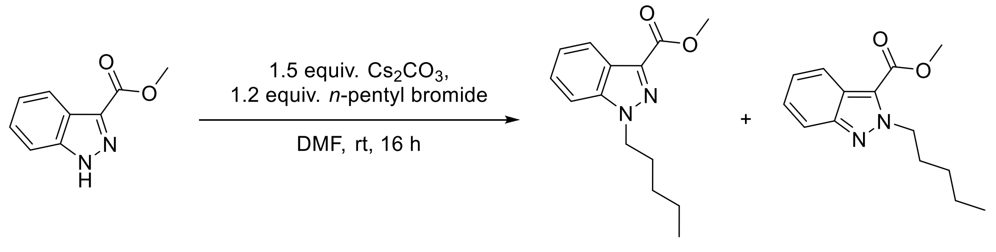

^{1}H NMR (400 MHz, $CDCl_3$)

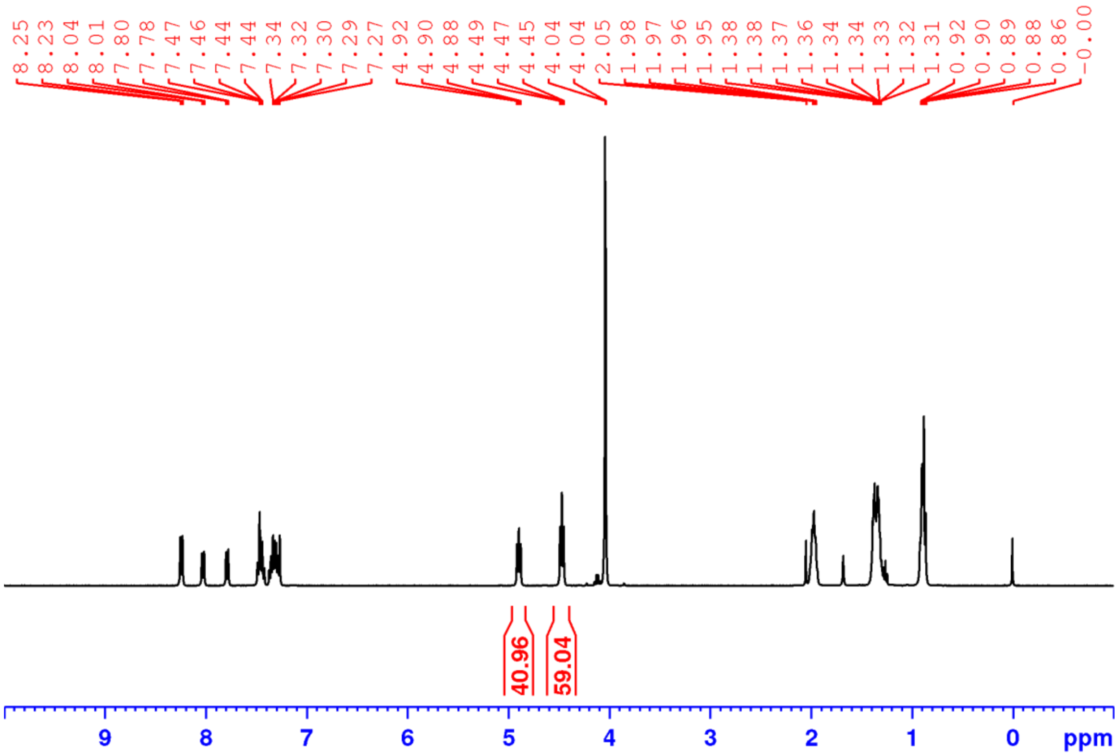

Table 1, Entry 18

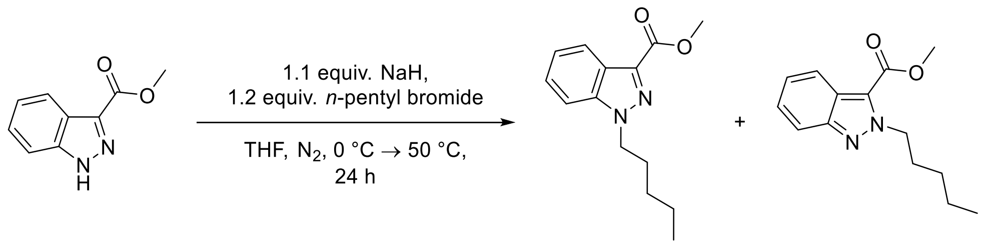

^{1}H NMR (400 MHz, $CDCl_3$)

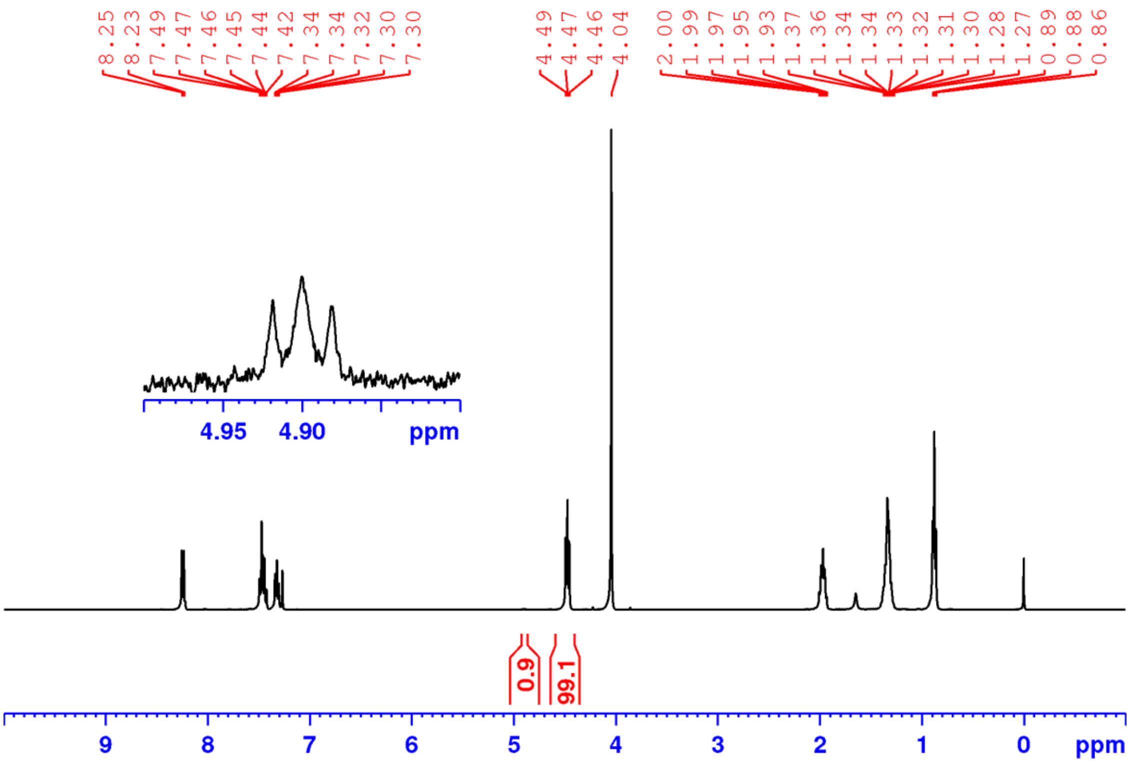

Scheme 1

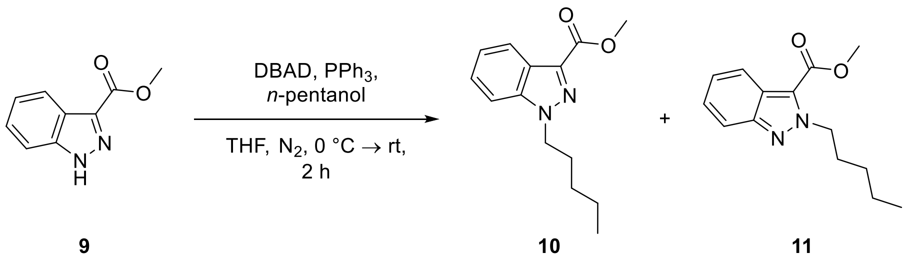

^{1}H NMR (300 MHz, $CDCl_3$)

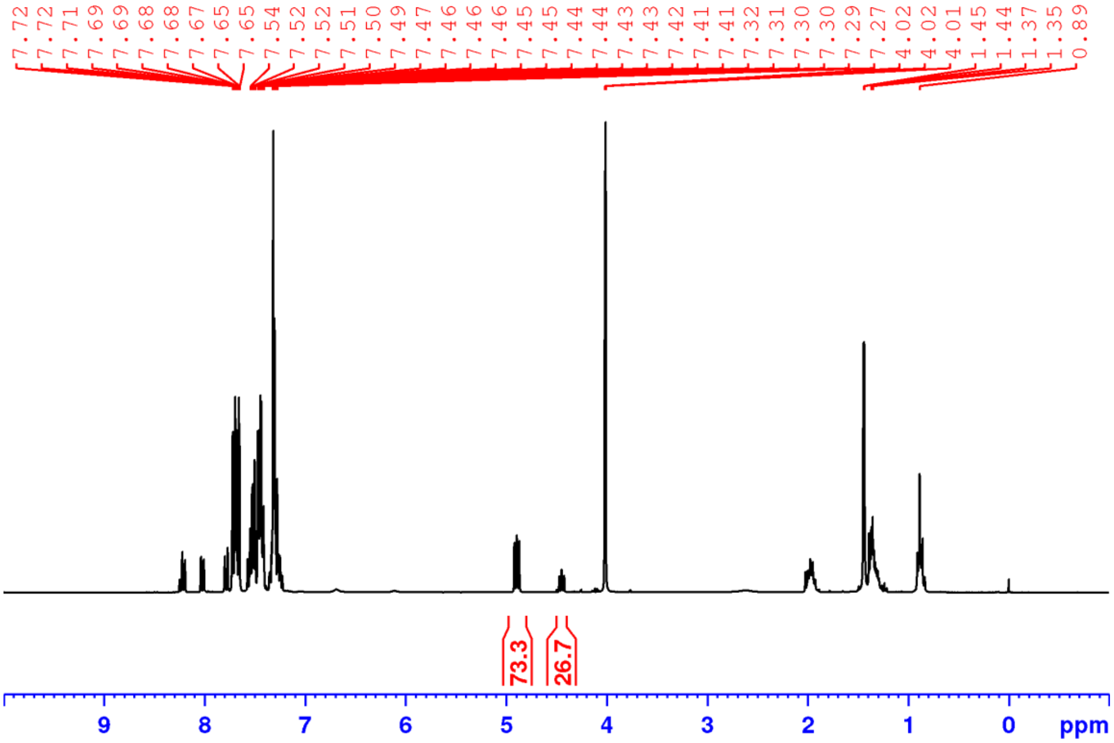

Table 2, Entry 1 (Conditions A)

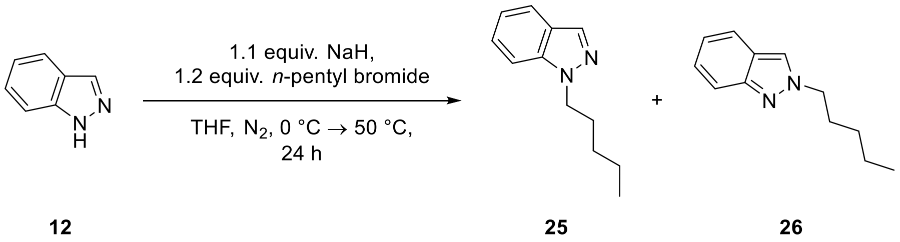

^{1}H NMR (400 MHz, $CDCl_3$)

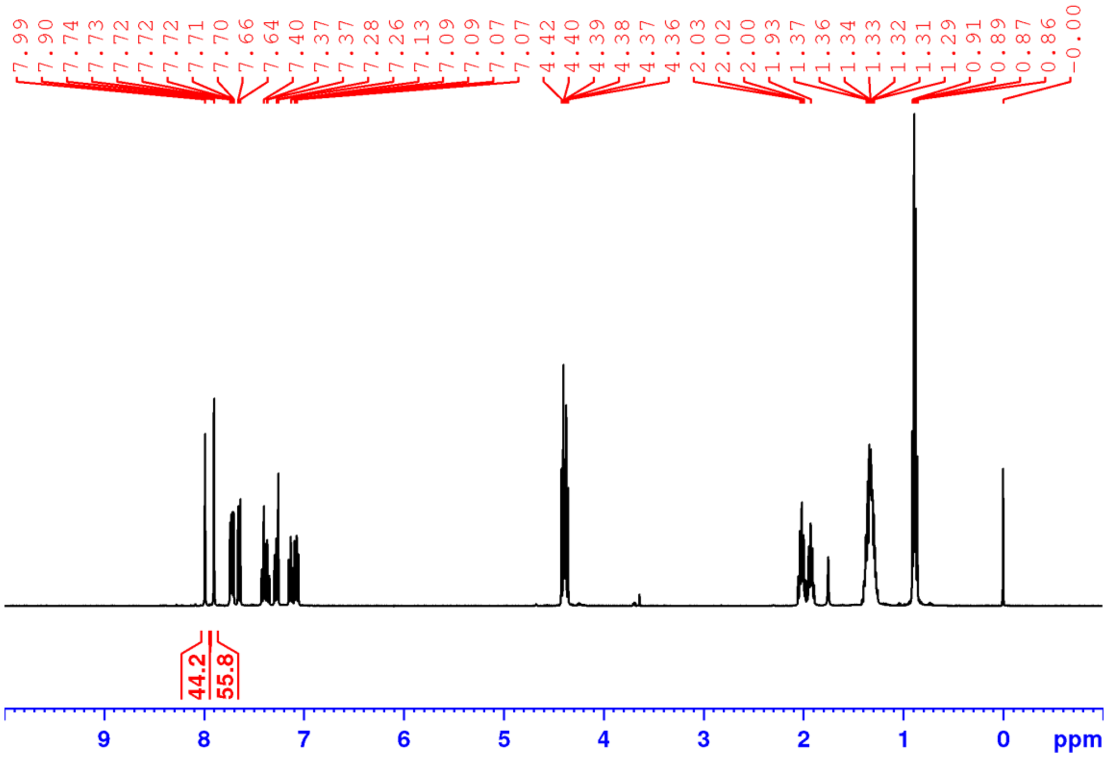

Table 2, Entry 1 (Conditions B)

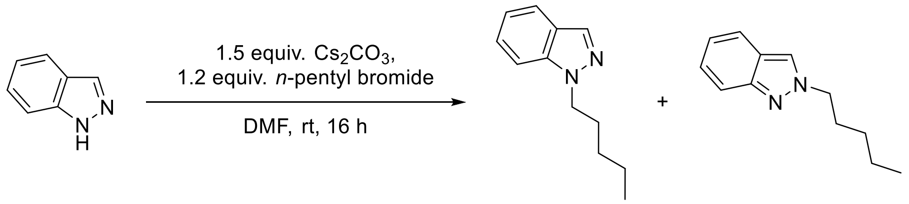

12 **25** **26**

^{1}H NMR (400 MHz, $CDCl_3$)

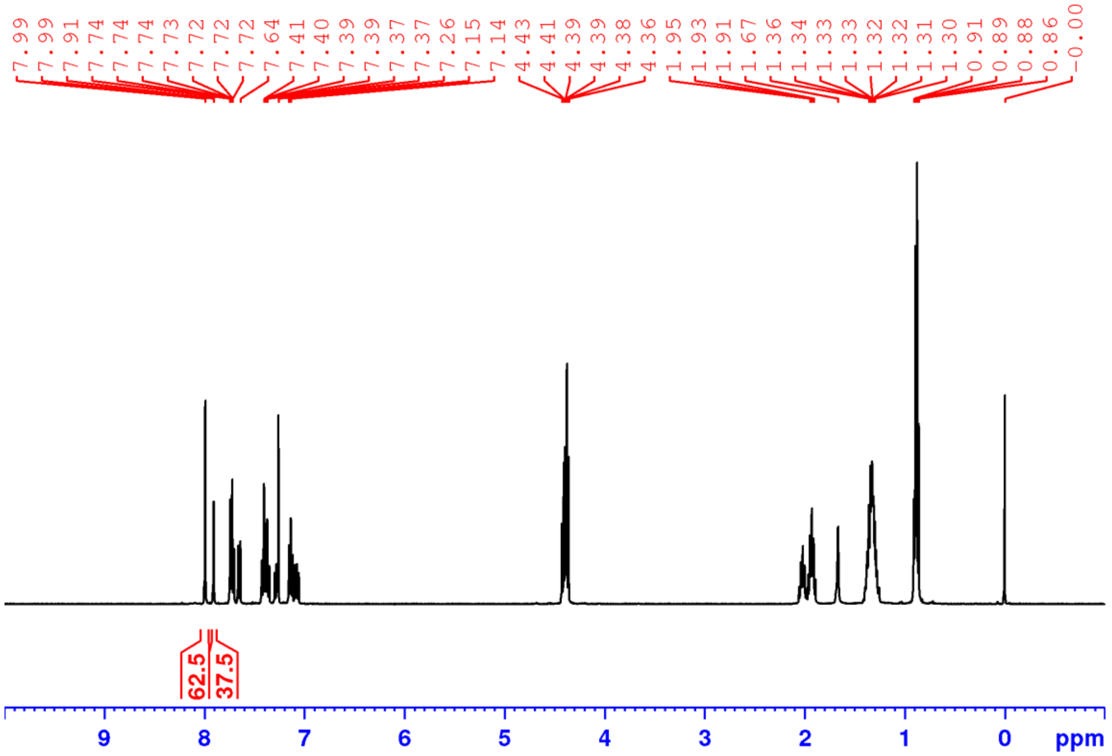

Table 2, Entry 2 (Conditions A)

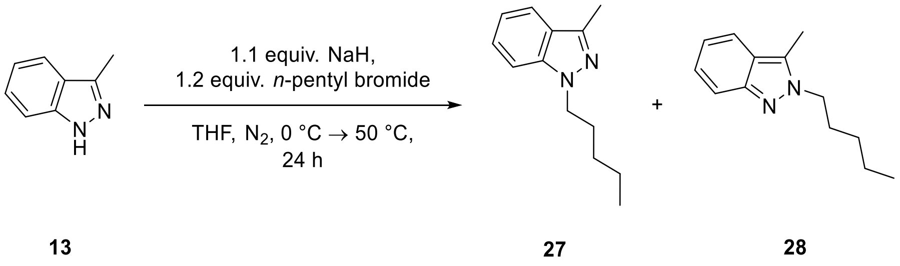

^{1}H NMR (400 MHz, $CDCl_3$)

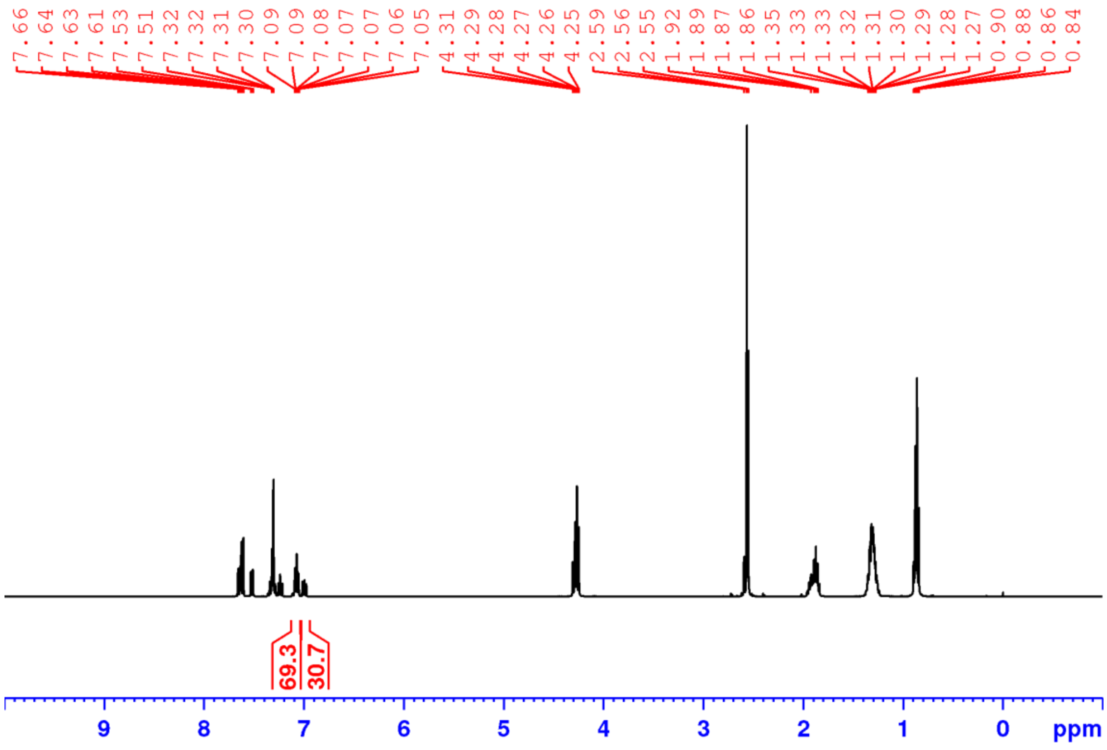

Table 2, Entry 2 (Conditions B)

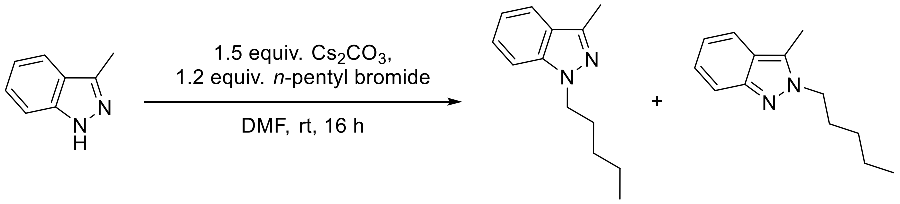

13 **27** **28**

^{1}H NMR (400 MHz, $CDCl_3$)

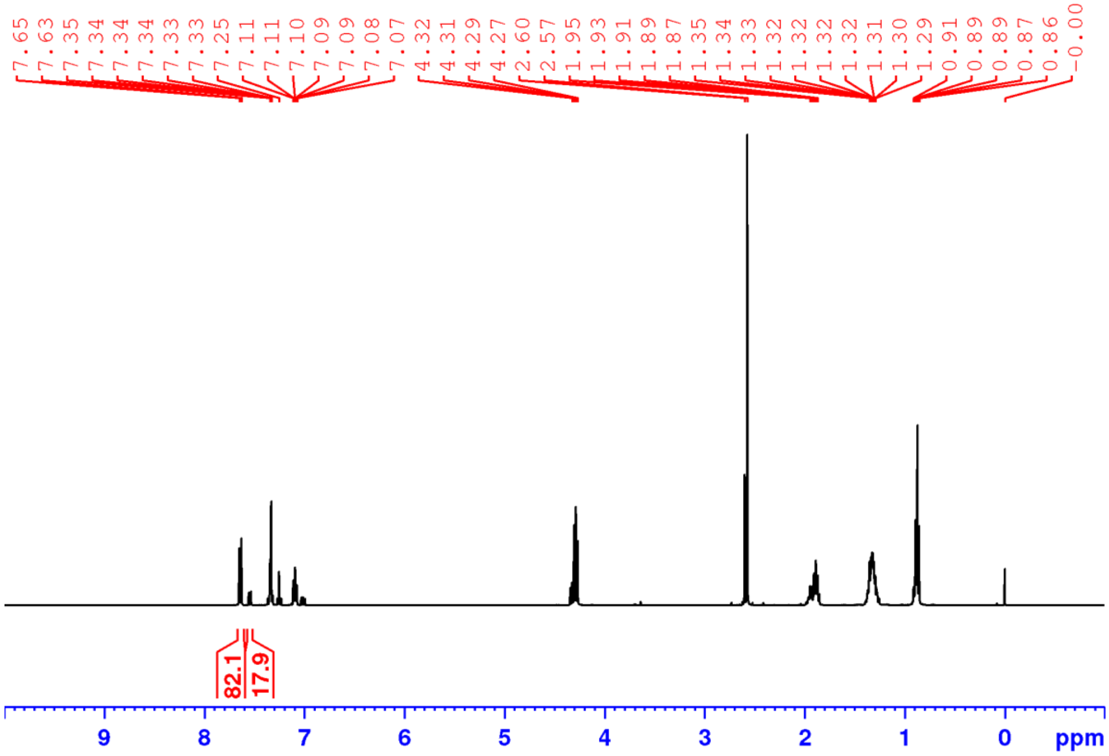

Table 2, Entry 3 (Conditions A)

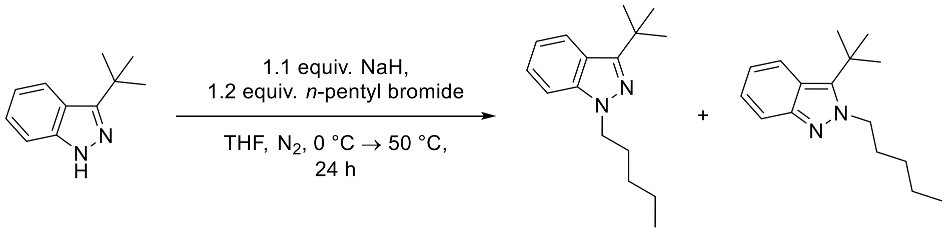

14 **29** **30**

^{1}H NMR (400 MHz, $CDCl_3$)

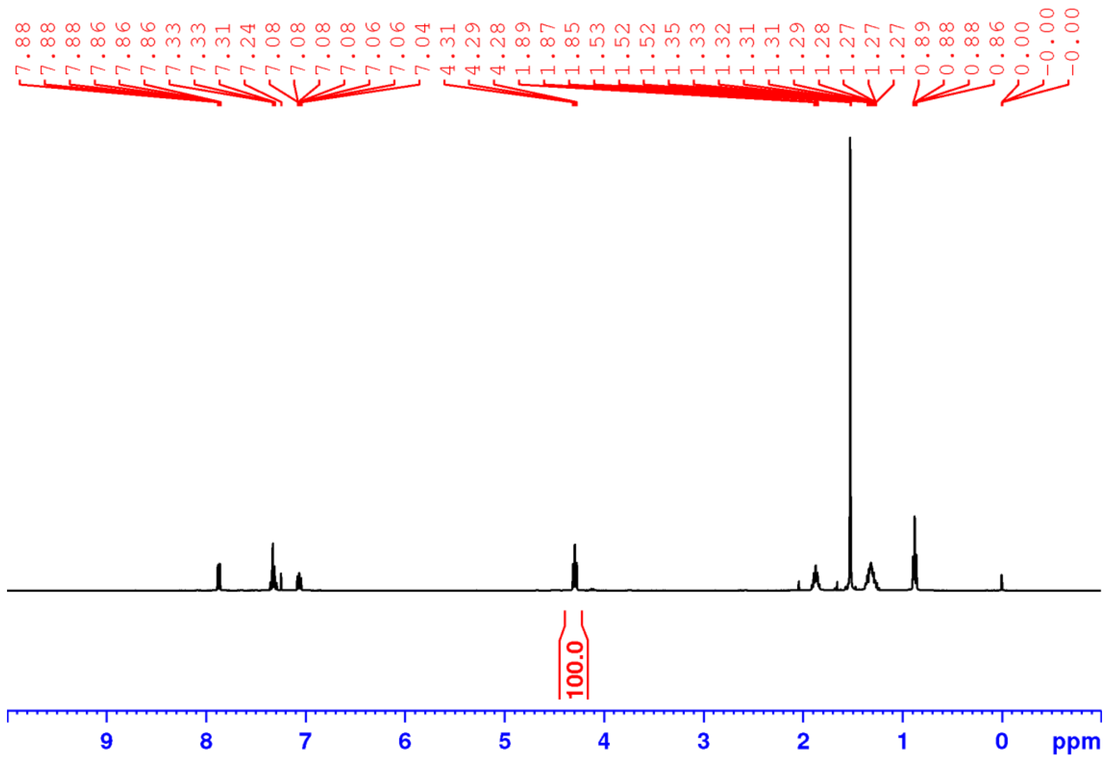

Table 2, Entry 3 (Conditions B)

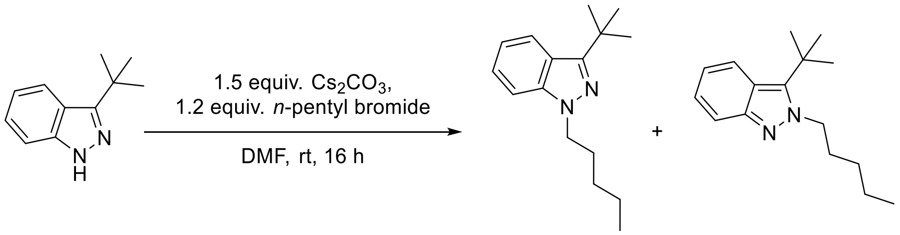

14 **29** **30**

^{1}H NMR (400 MHz, $CDCl_3$)

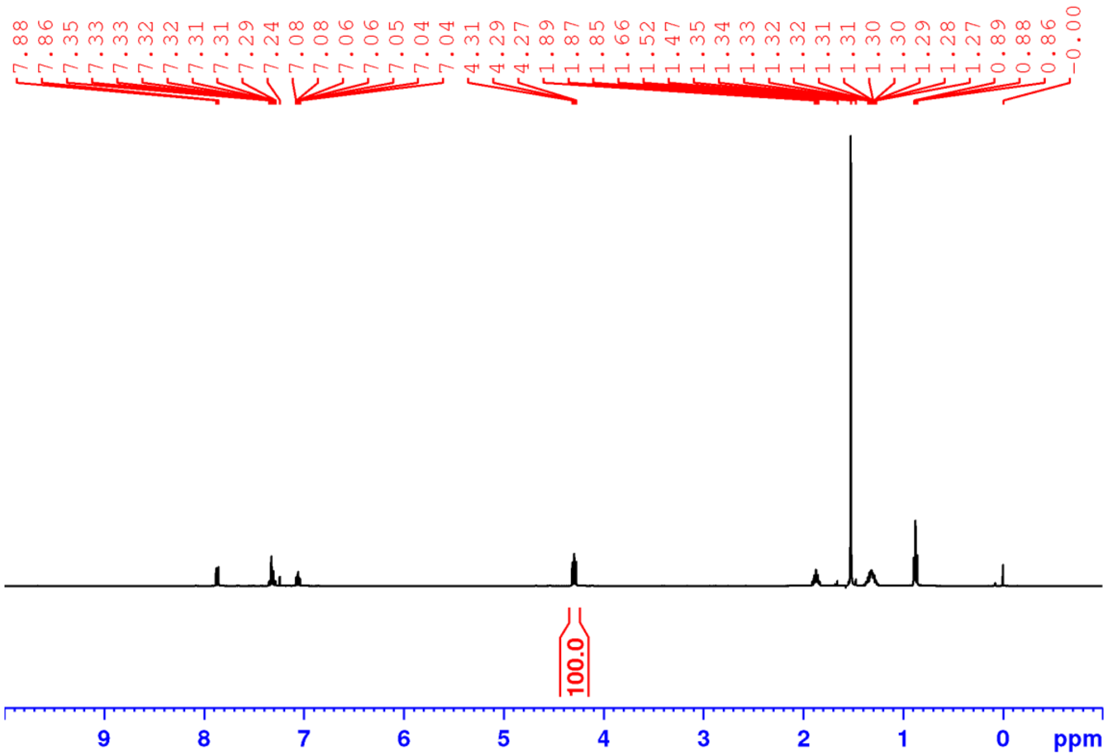

Table 2, Entry 4 (Conditions A)

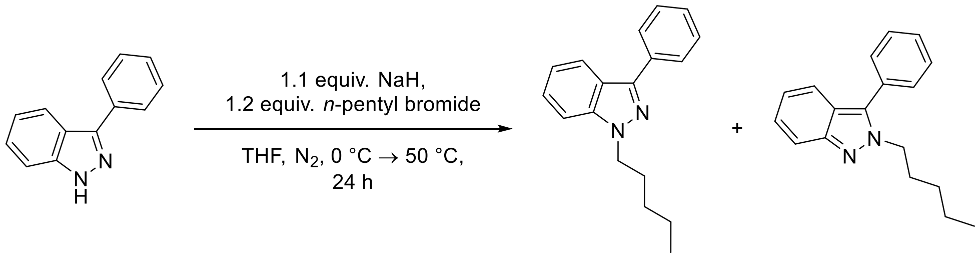

15 **31** **32**

^{1}H NMR (300 MHz, $CDCl_3$)

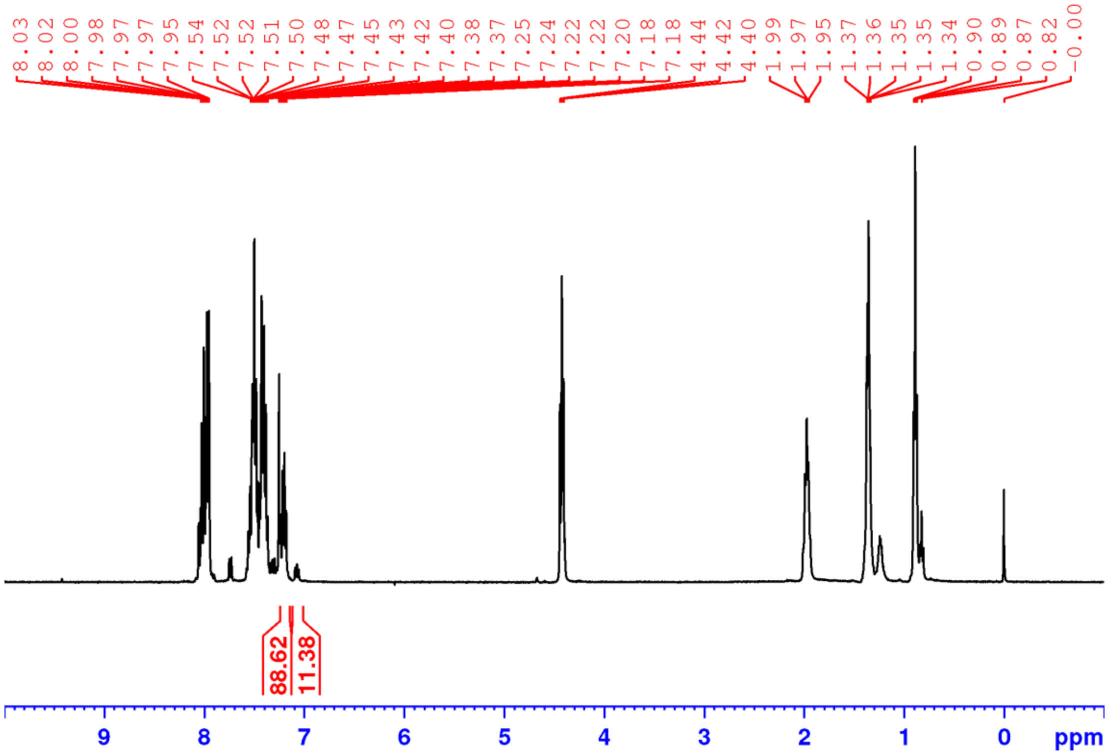

Table 2, Entry 4 (Conditions B)

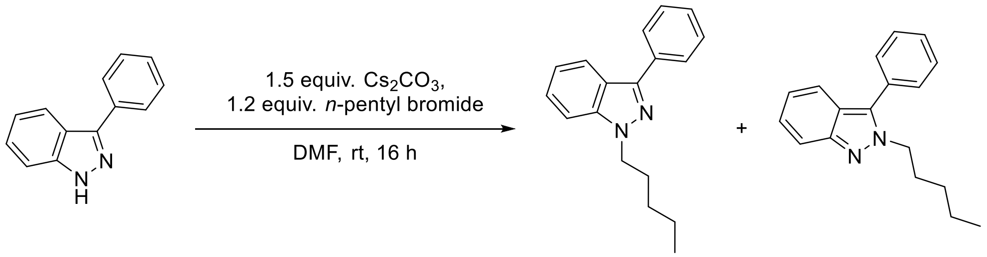

15 **31** **32**

^{1}H NMR (400 MHz, $CDCl_3$)

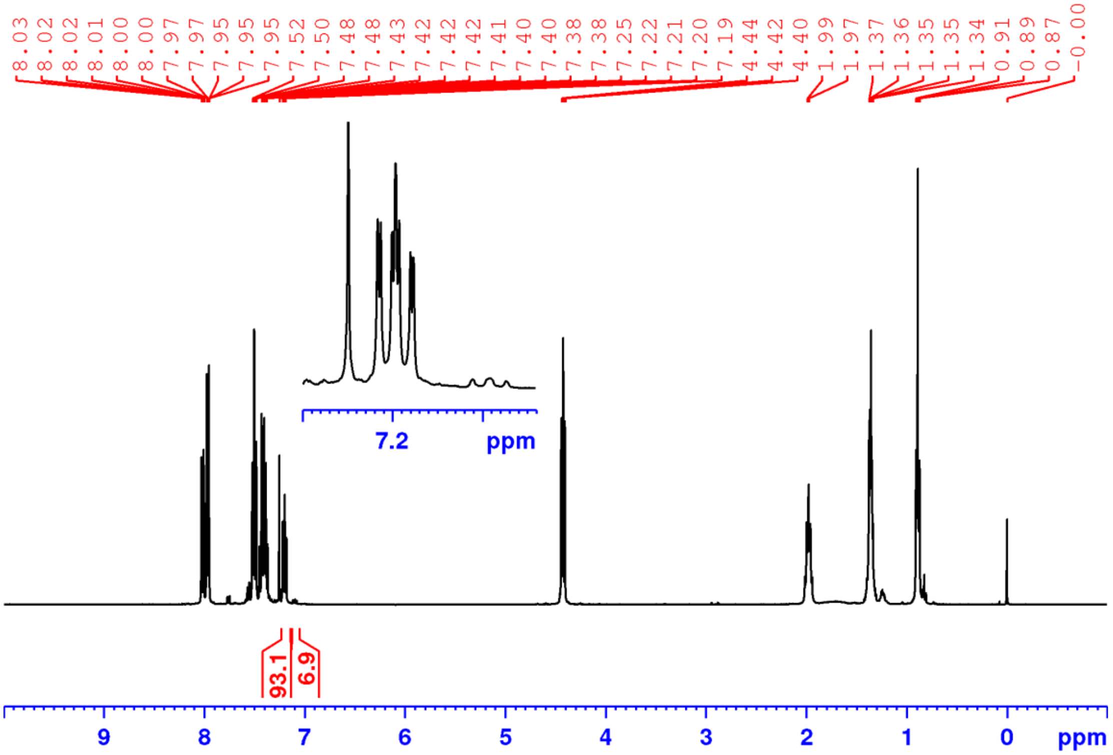

Table 2, Entry 5 (Conditions A)

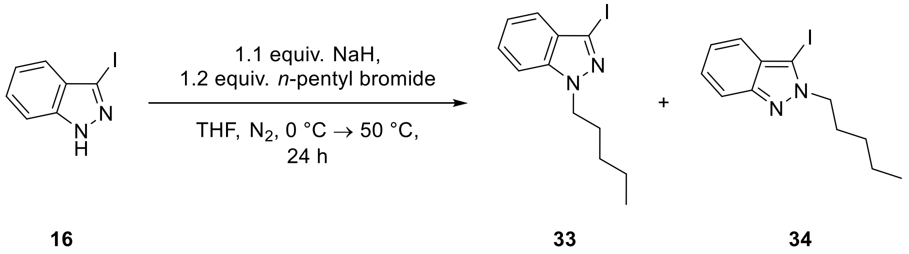

^{1}H NMR (300 MHz, $CDCl_3$)

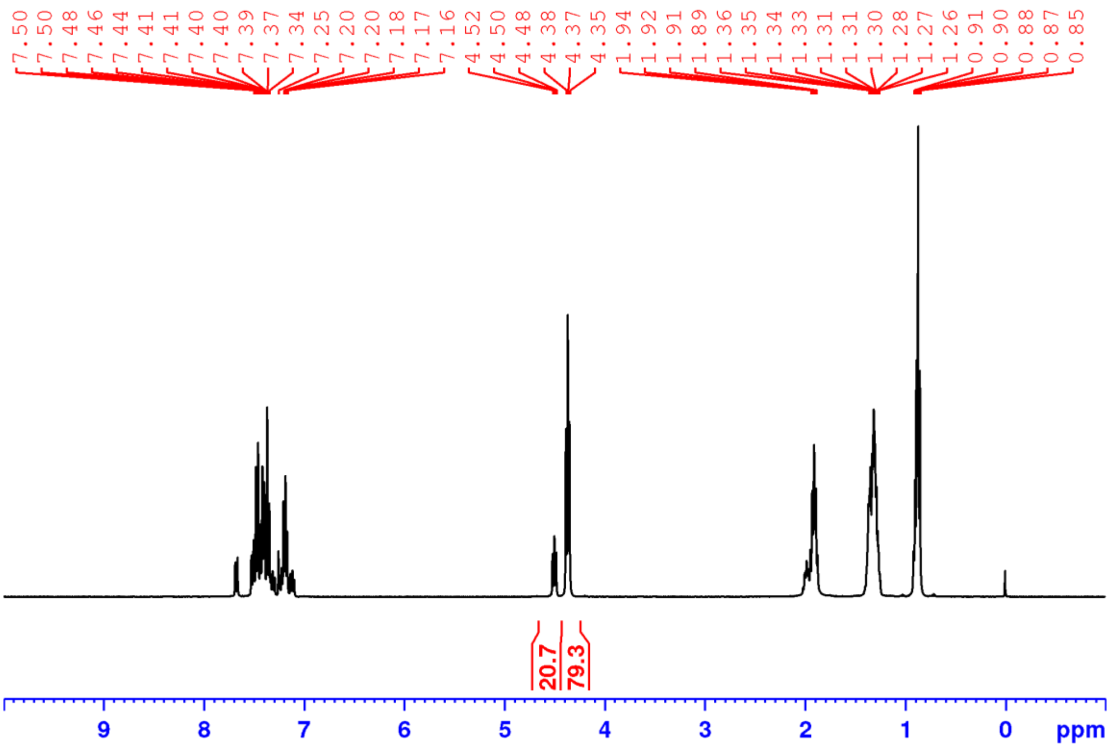

Table 2, Entry 5 (Conditions B)

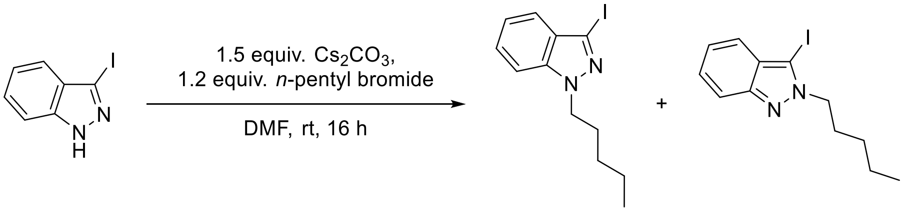

16 33 34

^{1}H NMR (400 MHz, $CDCl_3$)

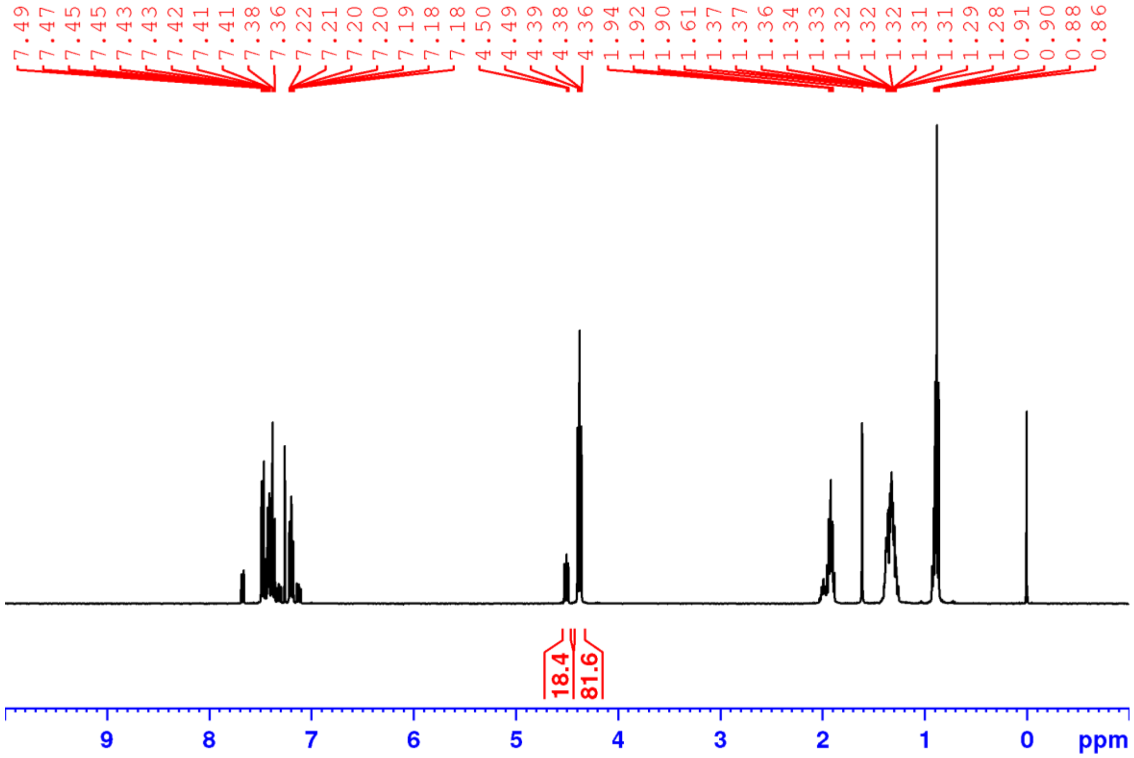

Table 2, Entry 6 (Conditions A)

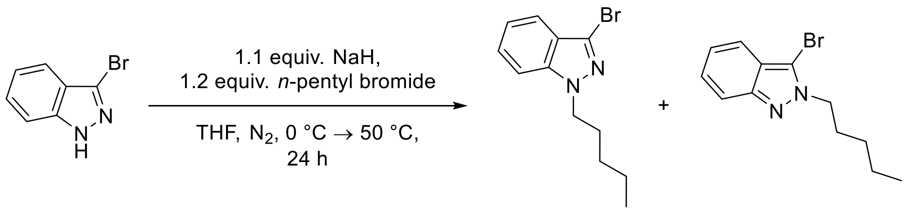

17 **35** **36**

^{1}H NMR (400 MHz, $CDCl_3$)

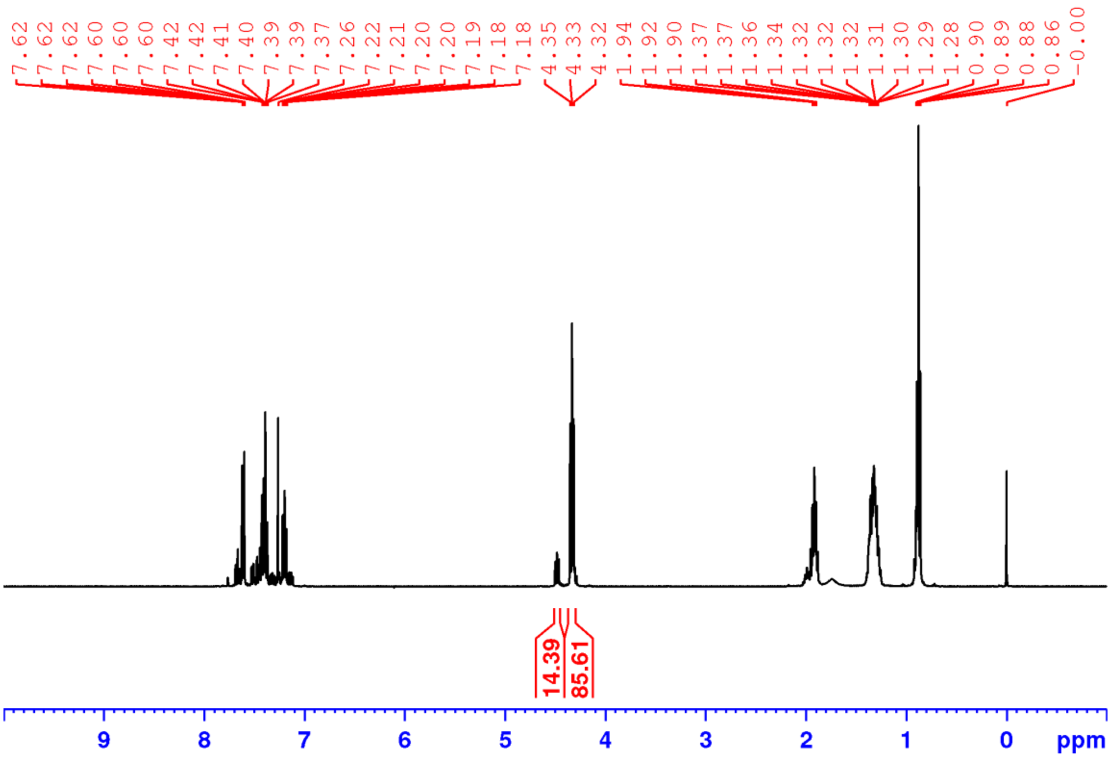

Table 2, Entry 6 (Conditions B)

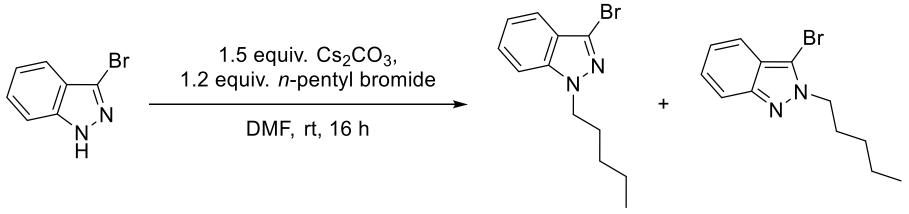

^{1}H NMR (400 MHz, $CDCl_3$)

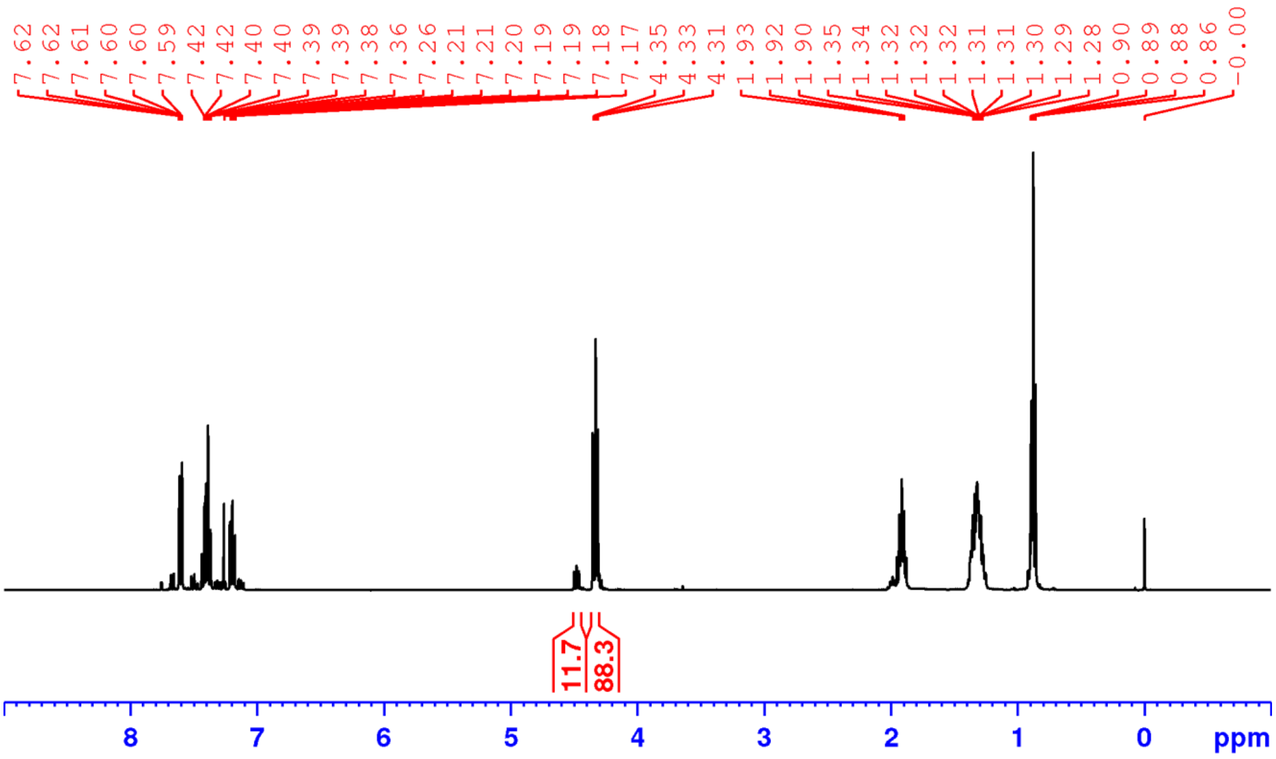

Table 2, Entry 7 (Conditions A)

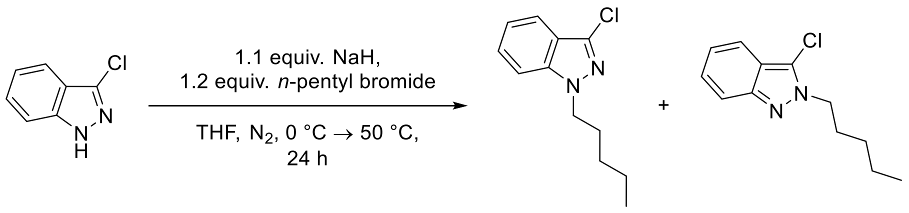

18 37 38

^{1}H NMR (300 MHz, $CDCl_3$)

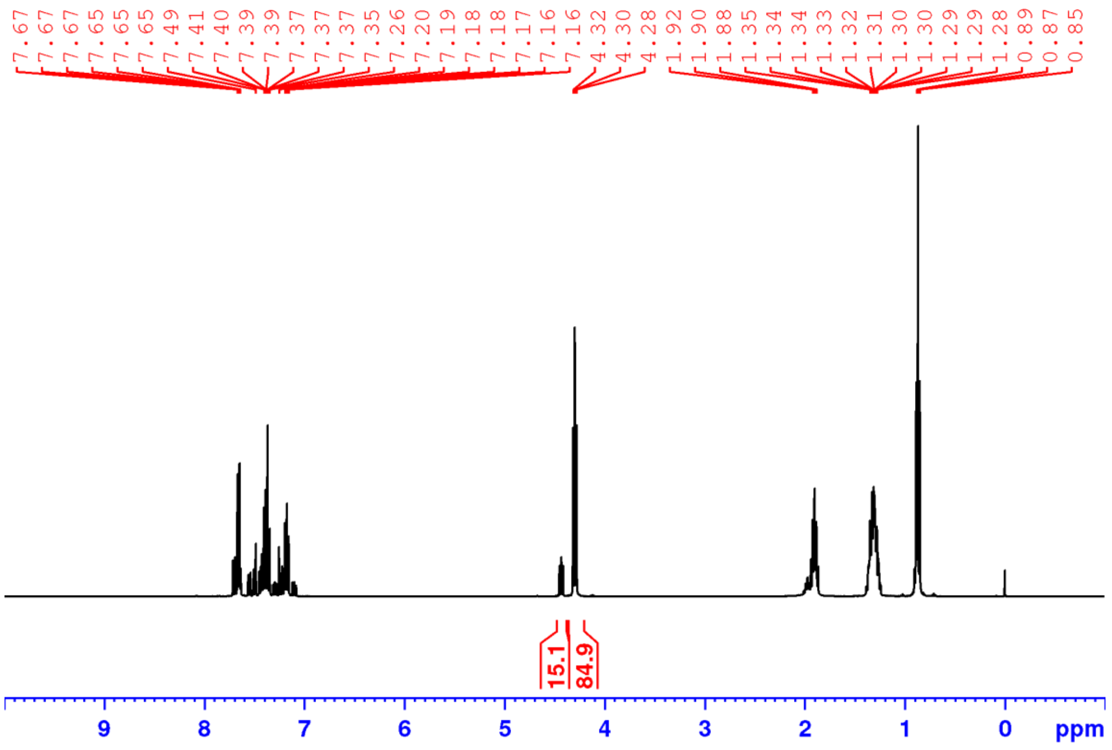

Table 2, Entry 7 (Conditions B)

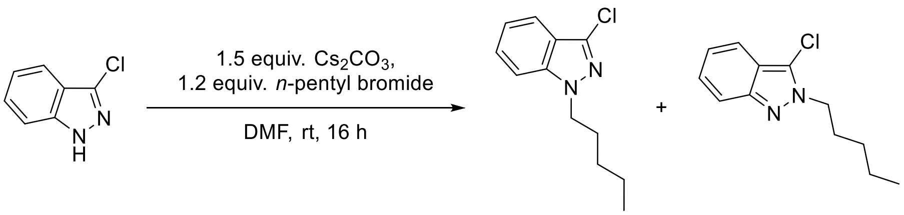

18 37 38

^{1}H NMR (400 MHz, $CDCl_3$)

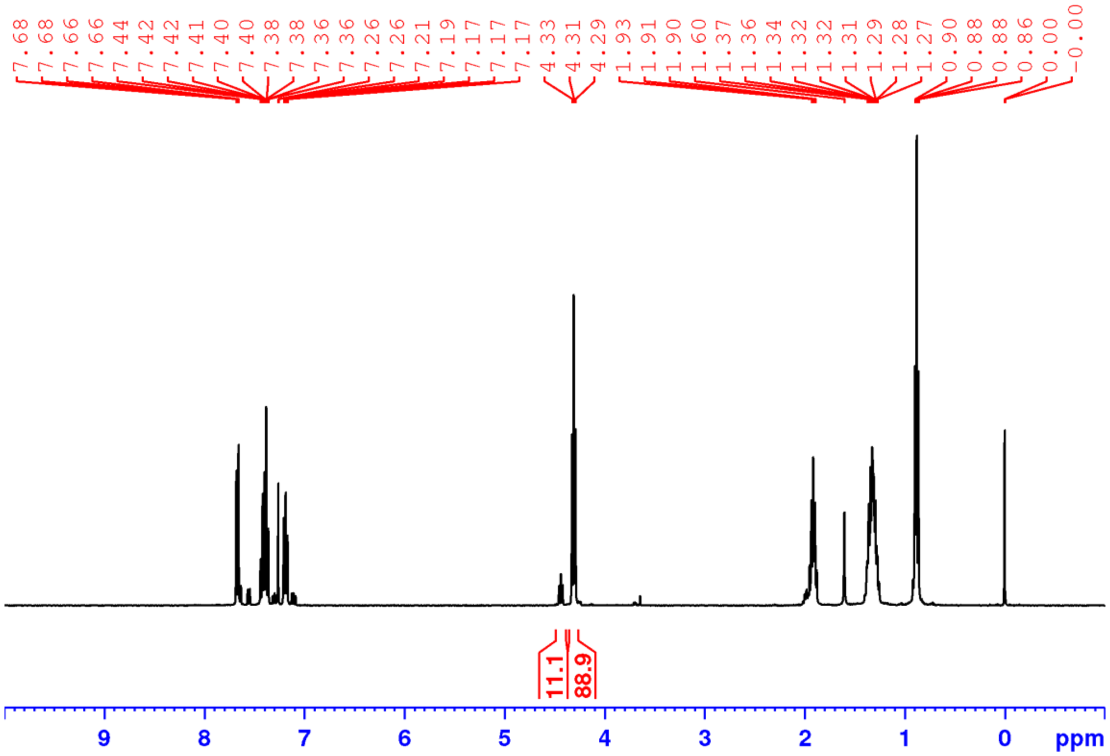

Table 2, Entry 8 (Conditions A)

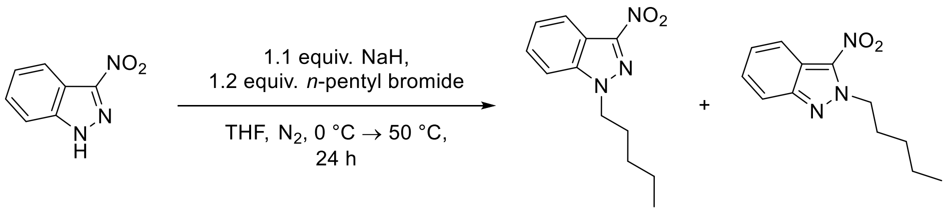

19 **39** **40**

^{1}H NMR (400 MHz, $CDCl_3$)

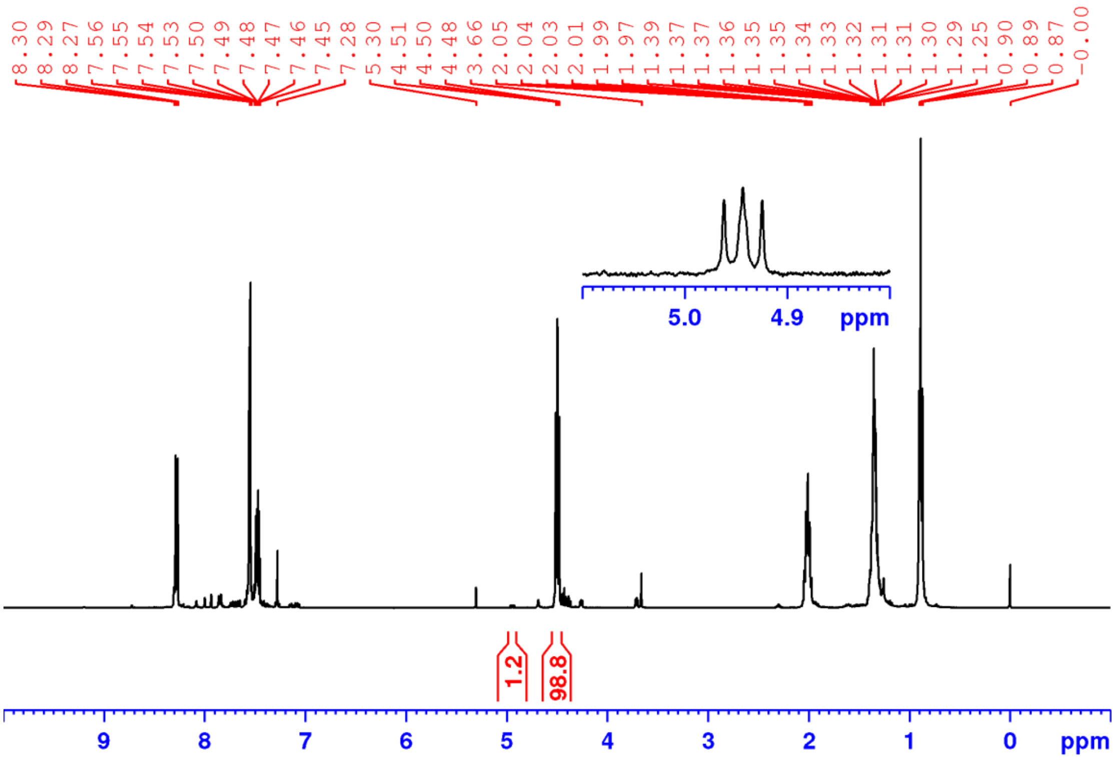

Table 2, Entry 8 (Conditions B)

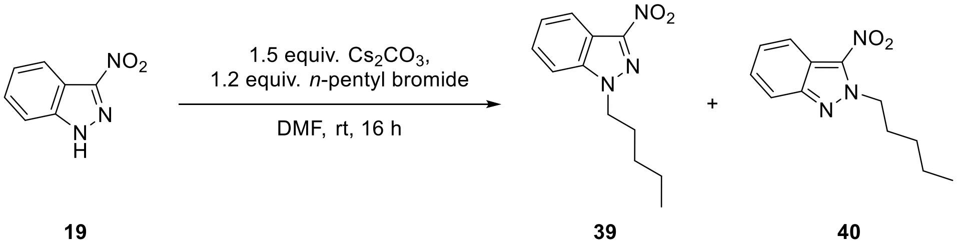

^{1}H NMR (400 MHz, $CDCl_3$)

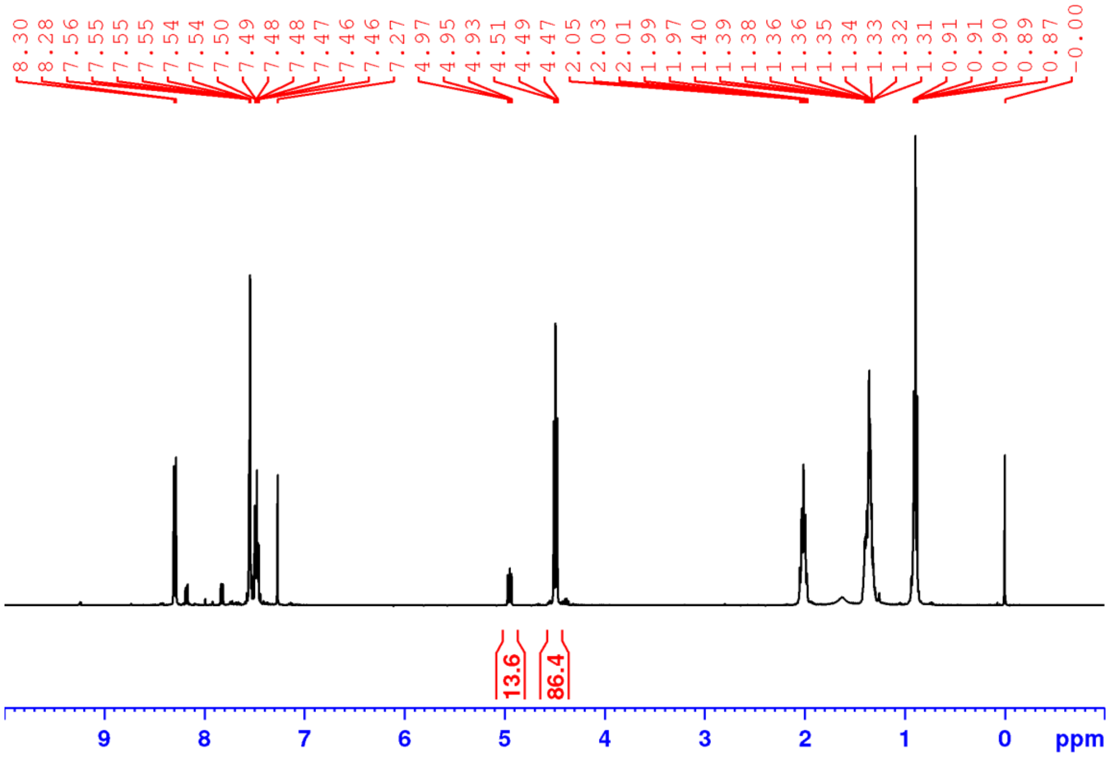

Table 2, Entry 9 (Conditions A)

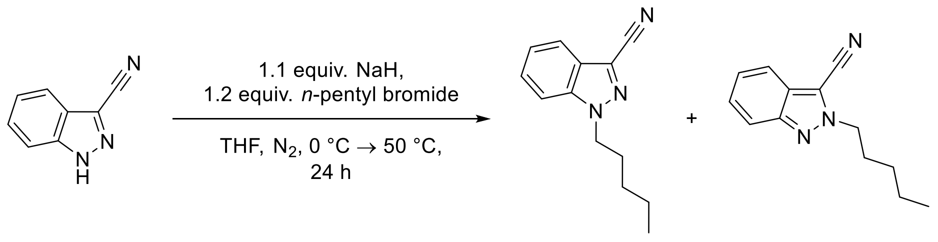

20 41 42

^{1}H NMR (400 MHz, $CDCl_3$)

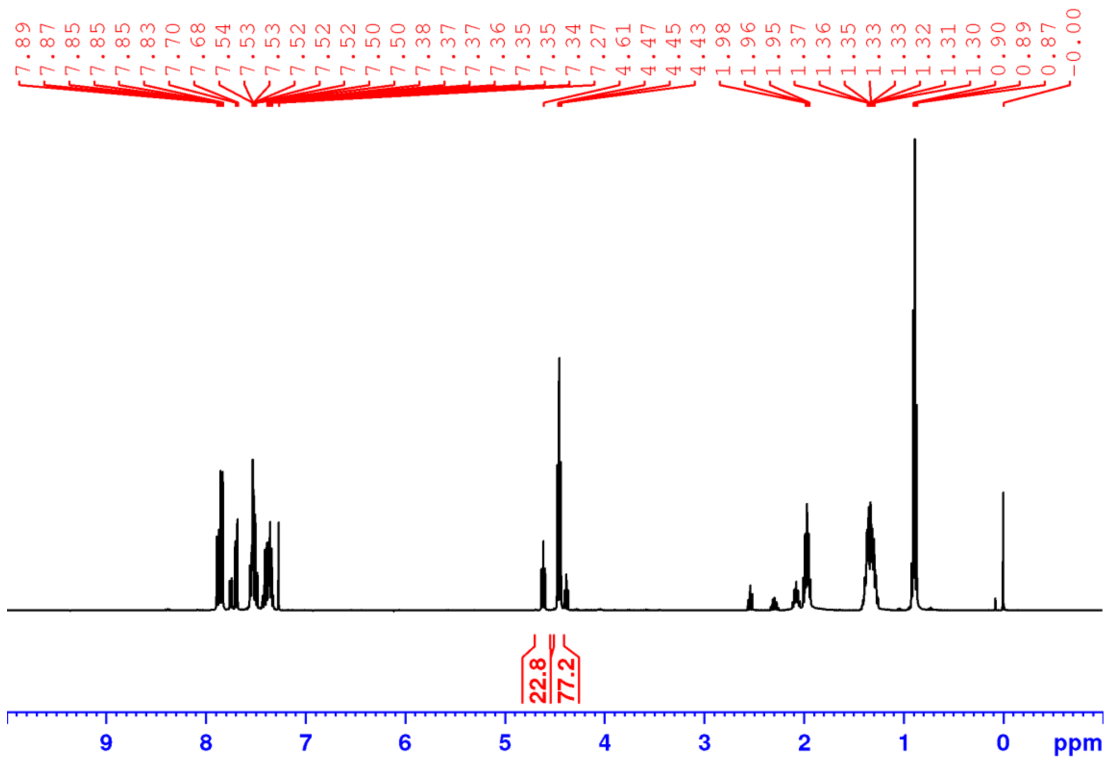

Table 2, Entry 9 (Conditions B)

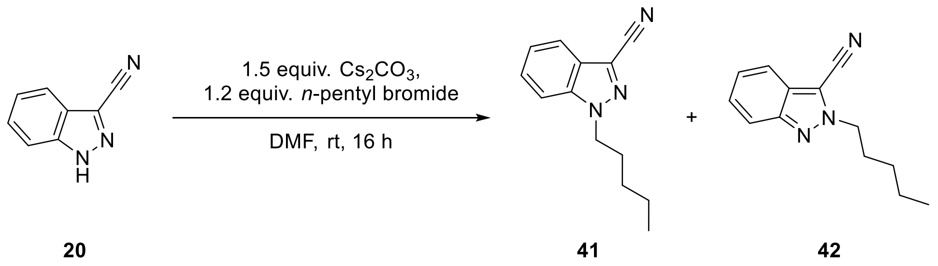

^{1}H NMR (400 MHz, $CDCl_3$)

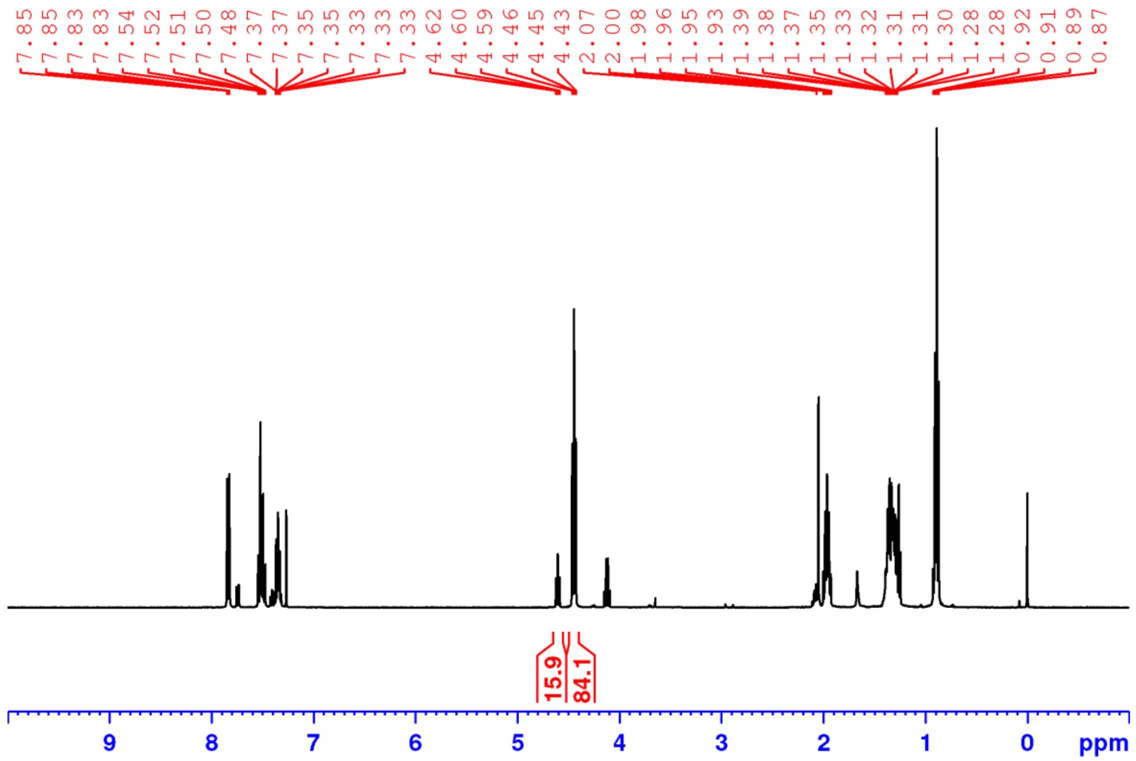

Table 2, Entry 10 (Conditions A)

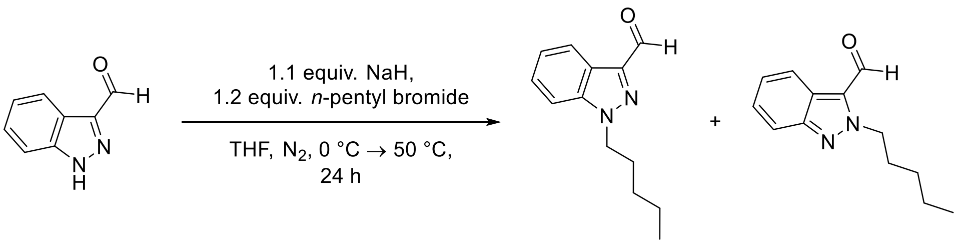

21 43 44

^{1}H NMR (400 MHz, $CDCl_3$)

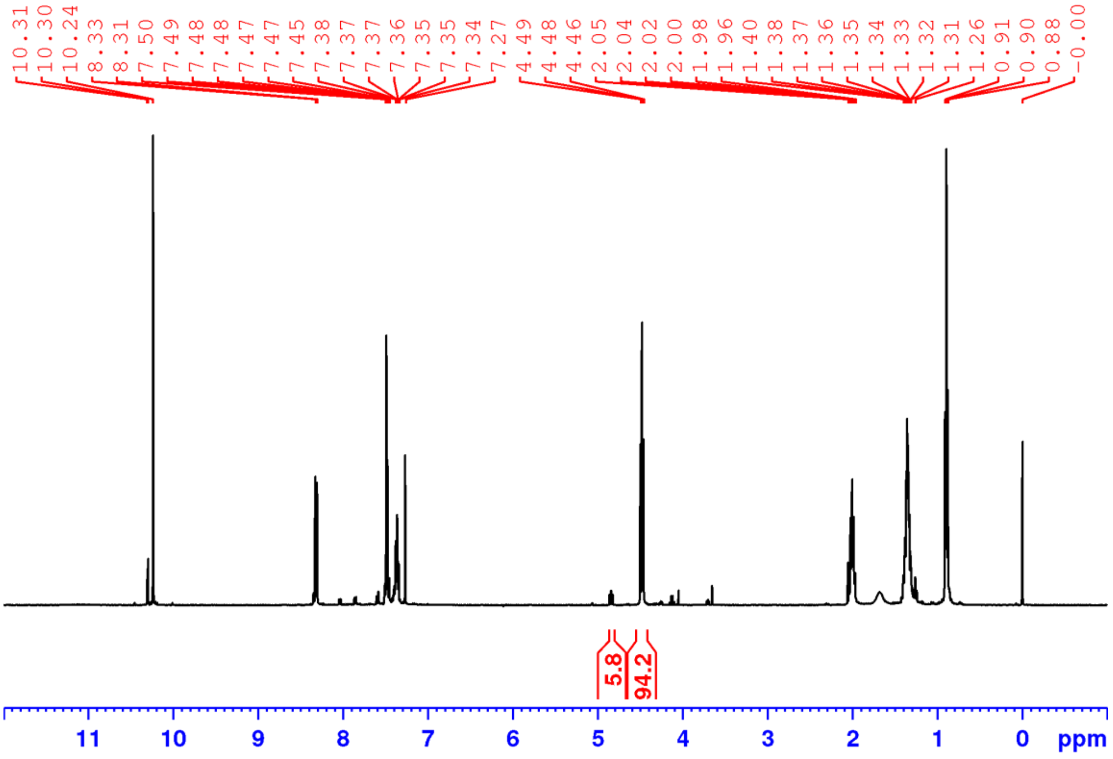

Table 2, Entry 10 (Conditions B)

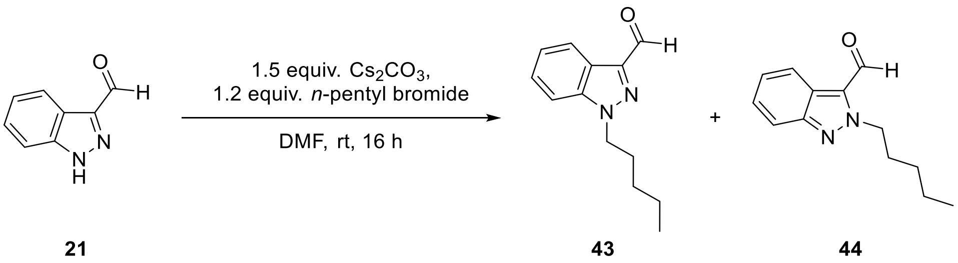

^{1}H NMR (400 MHz, $CDCl_3$)

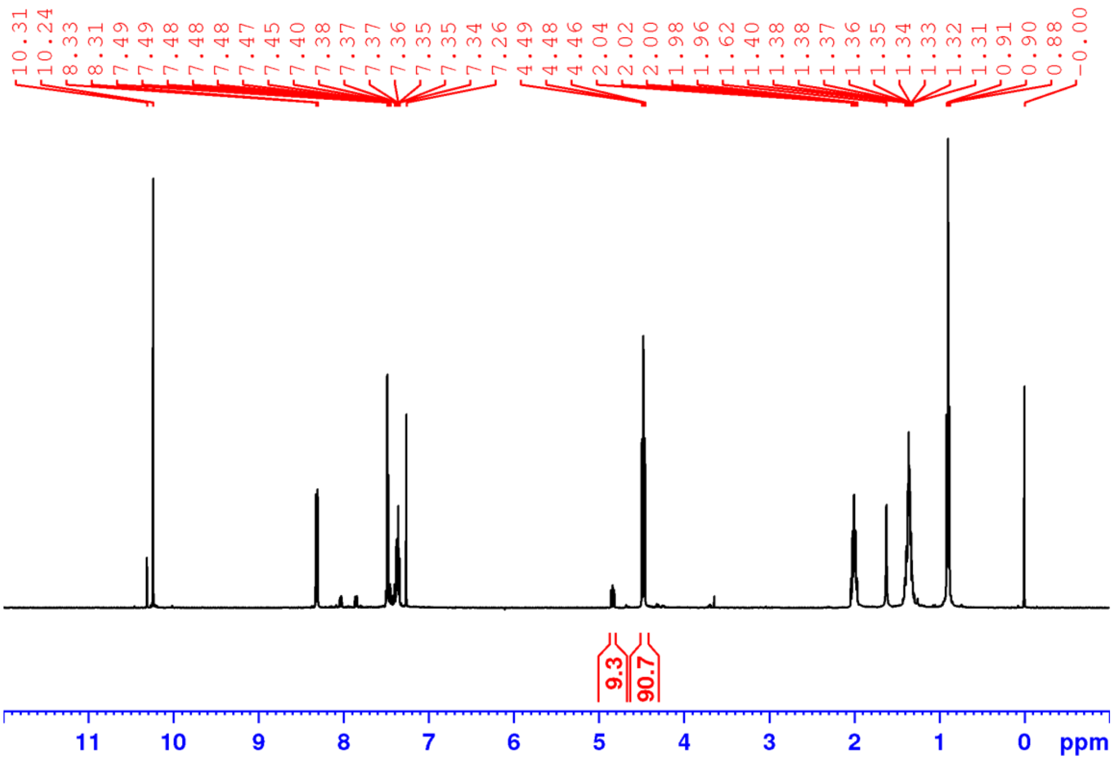

Table 2, Entry 11 (Conditions A)

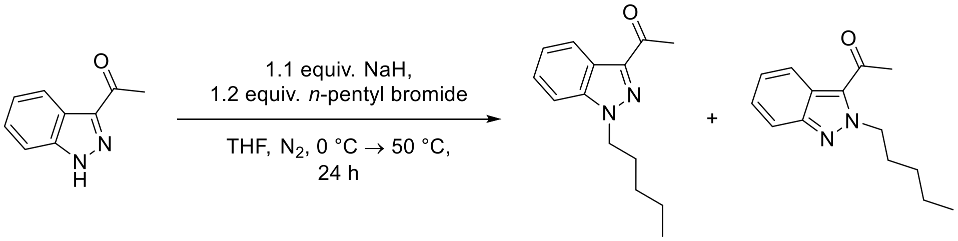

22 **45** **46**

^{1}H NMR (300 MHz, $CDCl_3$)

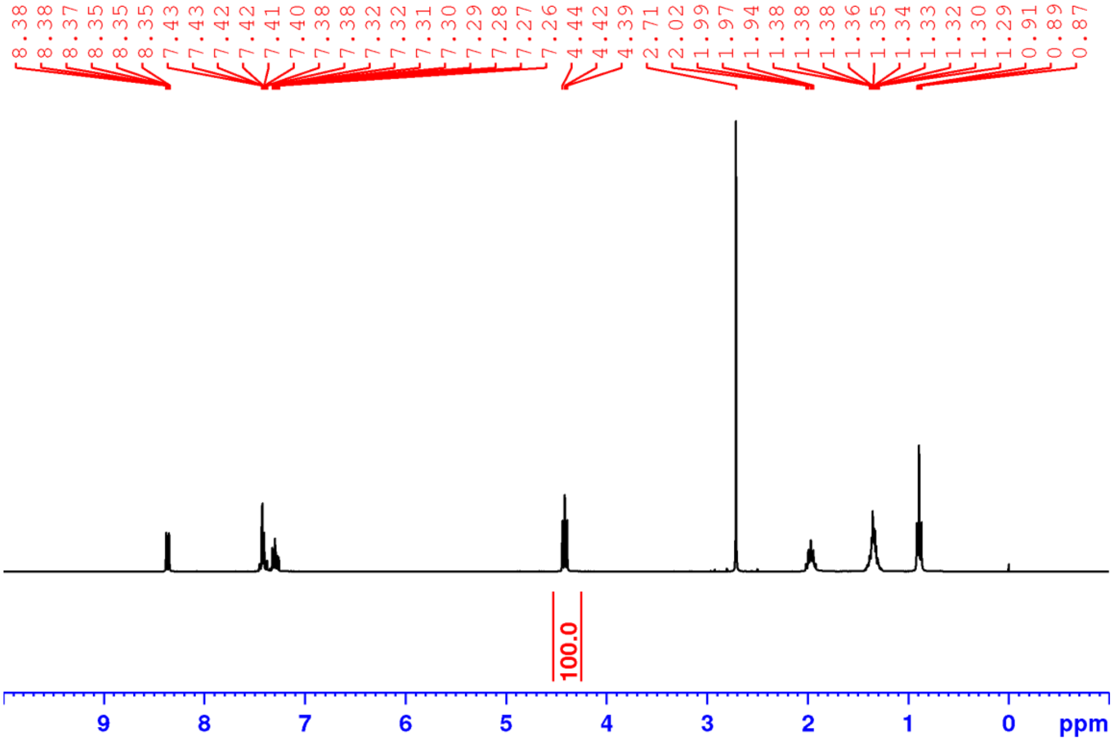

Table 2, Entry 11 (Conditions B)

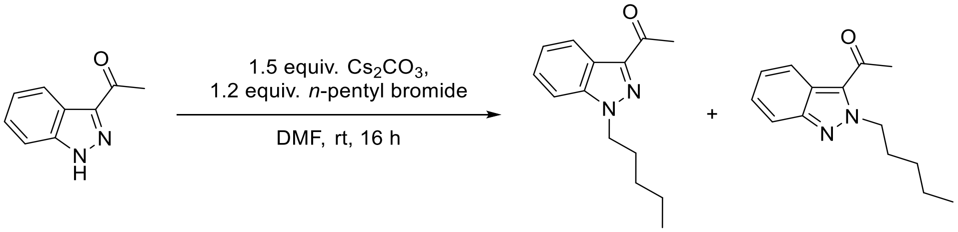

22 **45** **46**

^{1}H NMR (300 MHz, $CDCl_3$)

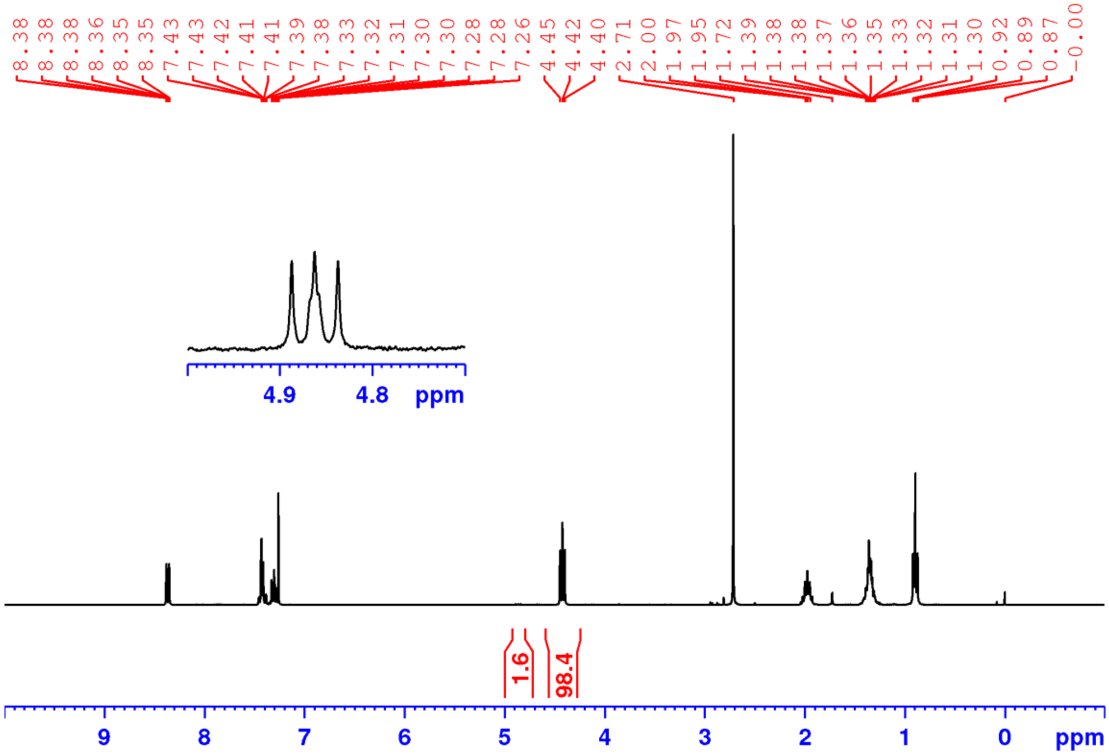

Table 2, Entry 12 (Conditions A)

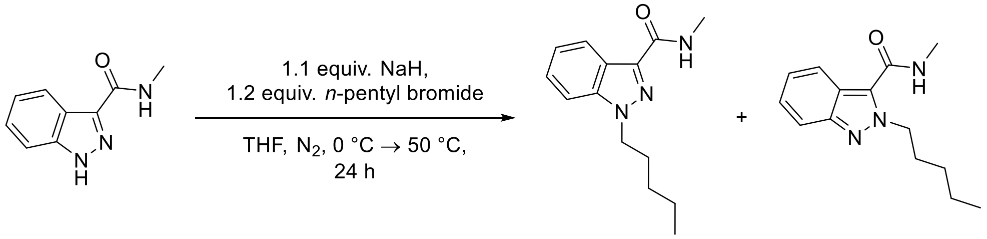

23 **47** **48**

^{1}H NMR (400 MHz, $CDCl_3$)

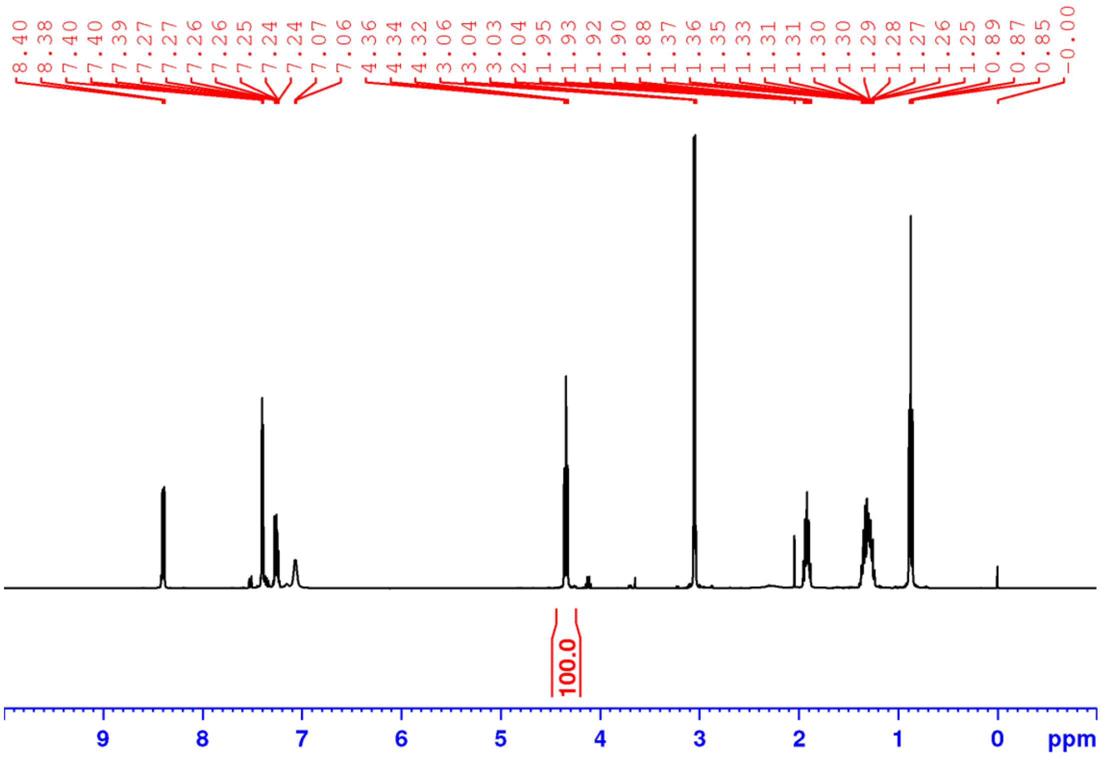

Table 2, Entry 12 (Conditions B)

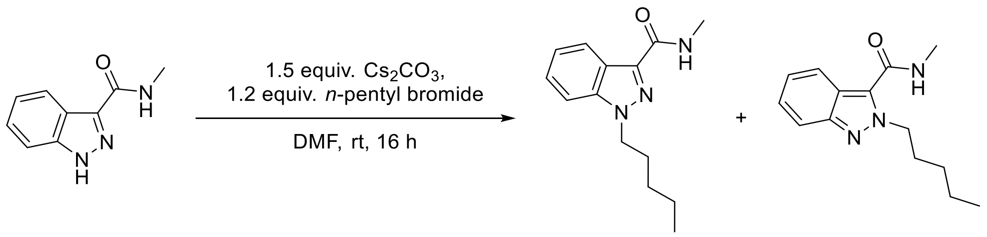

23 **47** **48**

^{1}H NMR (400 MHz, $CDCl_3$)

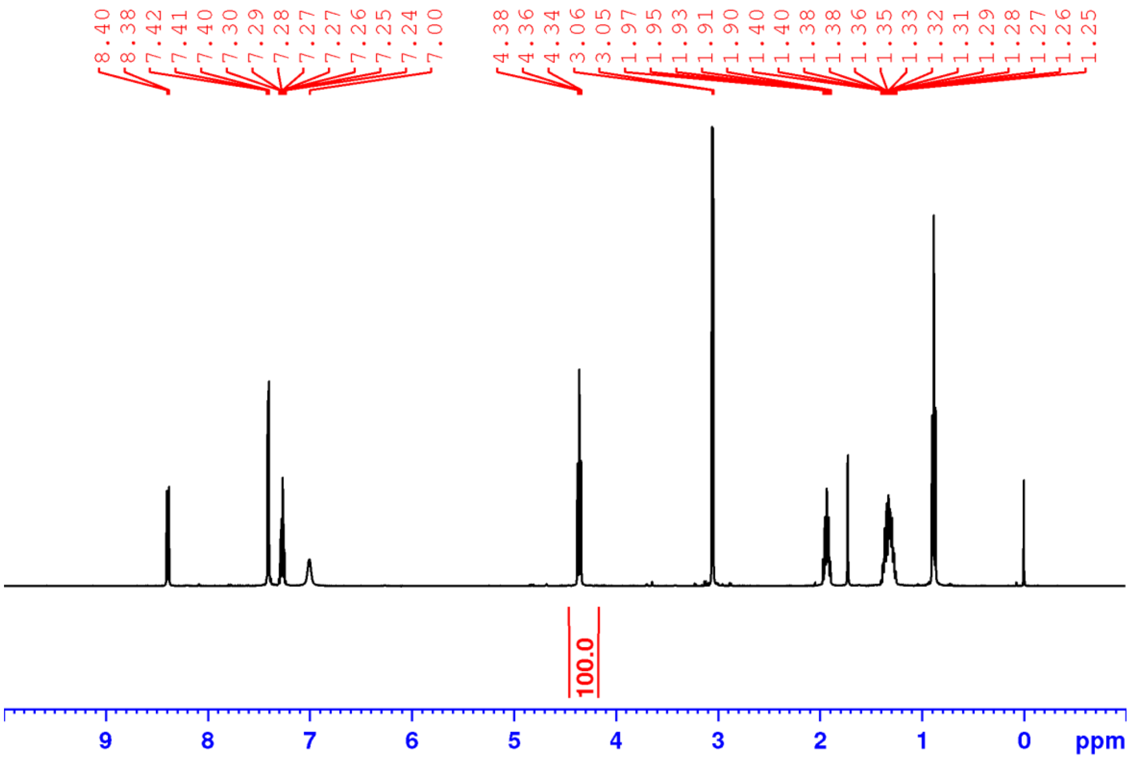

Table 2, Entry 13 (Conditions A)

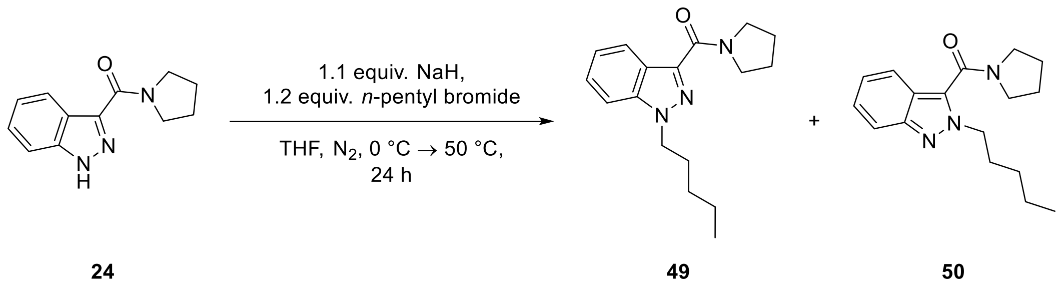

^{1}H NMR (300 MHz, $CDCl_3$)

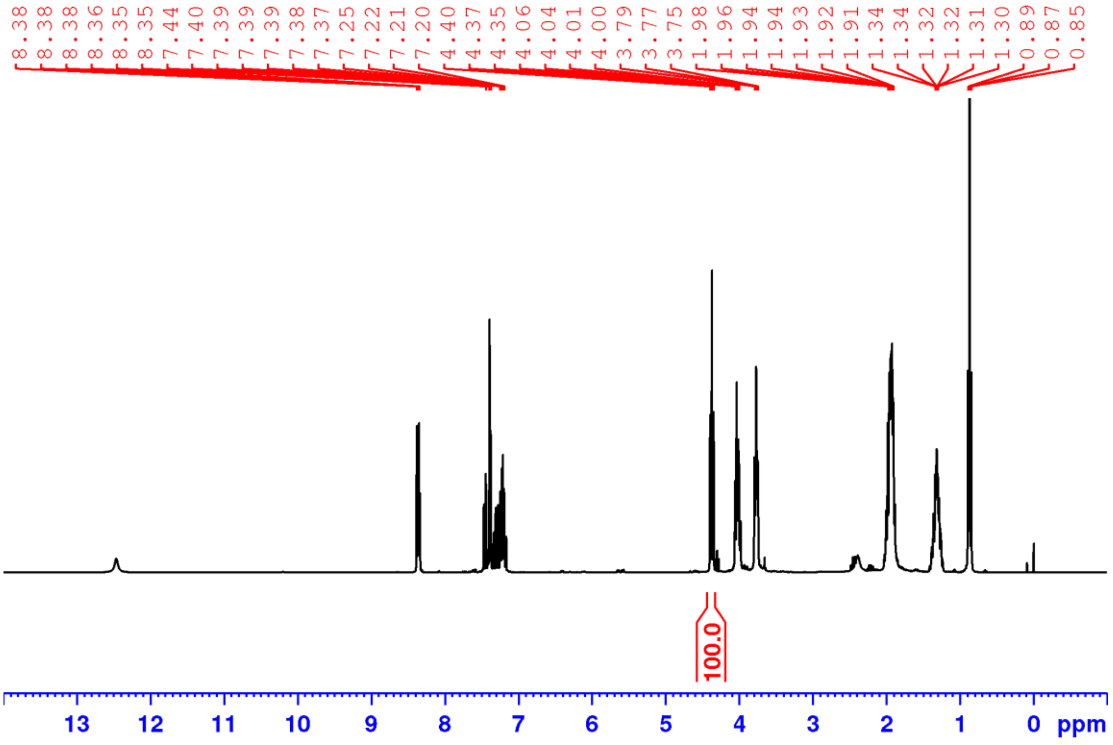

Table 2, Entry 13 (Conditions B)

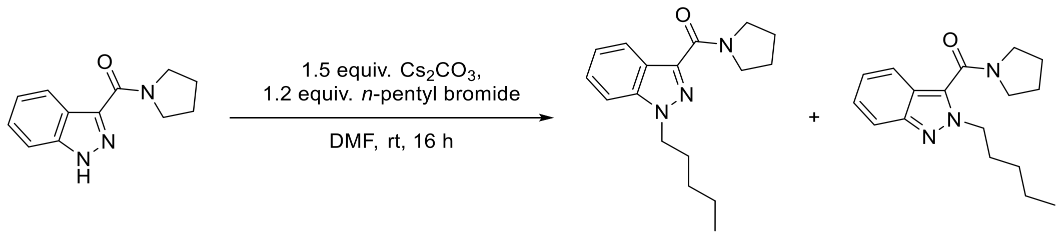

24 **49** **50**

^{1}H NMR (300 MHz, $CDCl_3$)

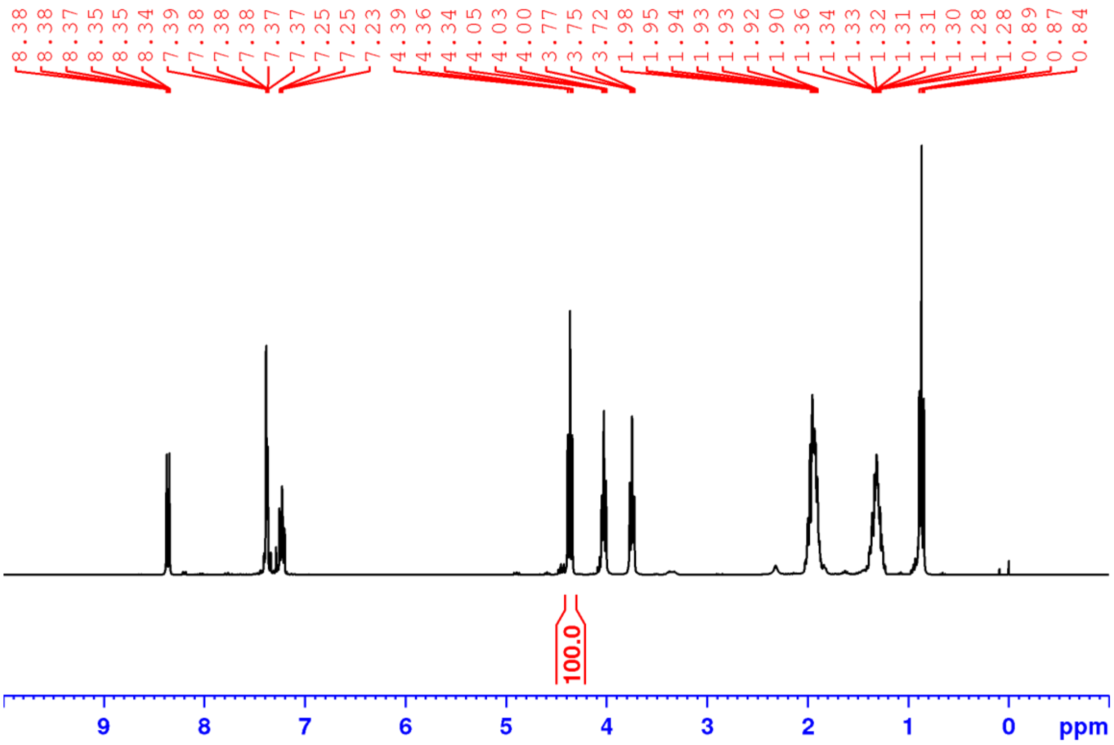

Table 3, Entry 1

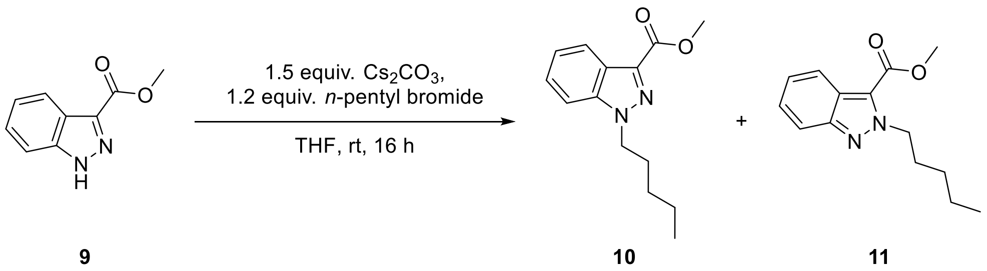

^1H NMR (300 MHz, $CDCl_3$)

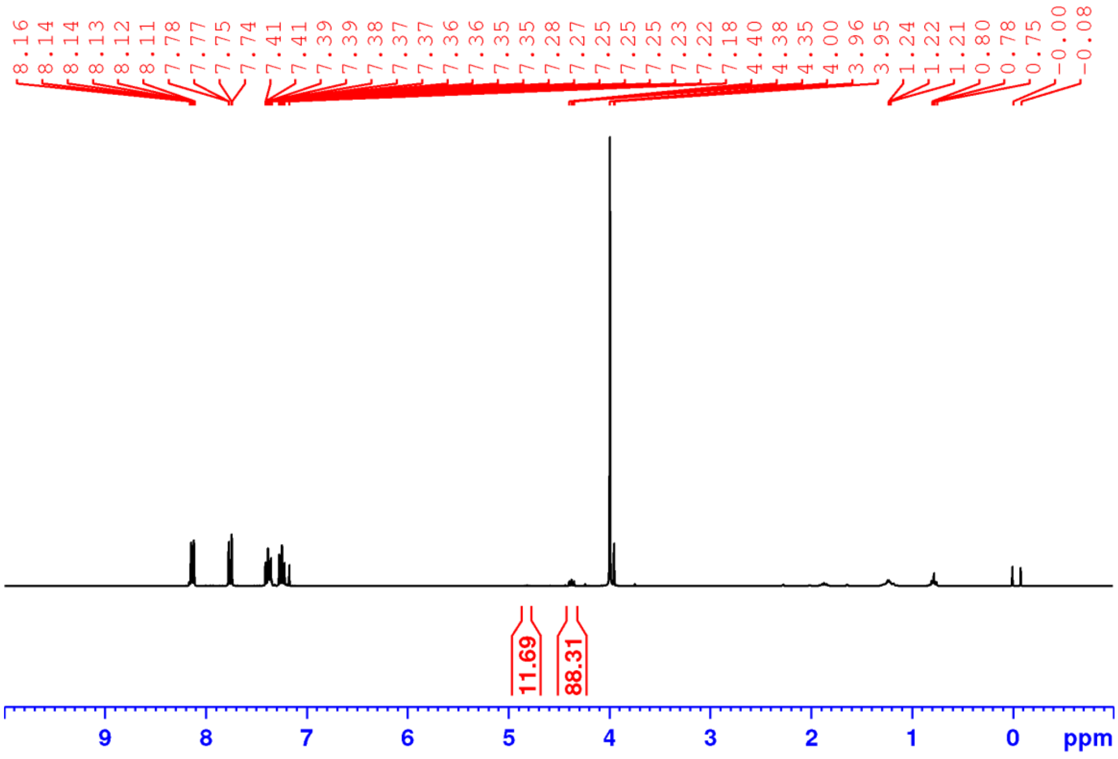

Table 3, Entry 2

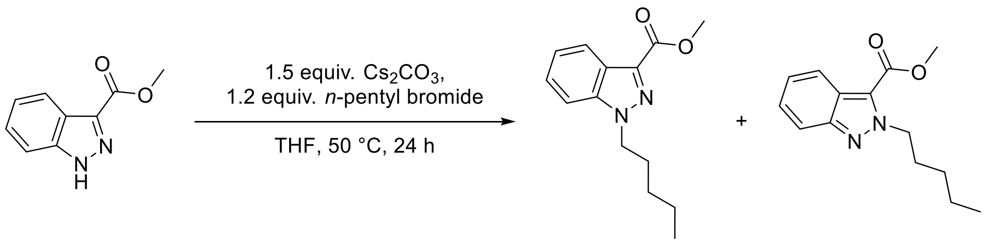

9 **10** **11**

^{1}H NMR (300 MHz, $CDCl_3$)

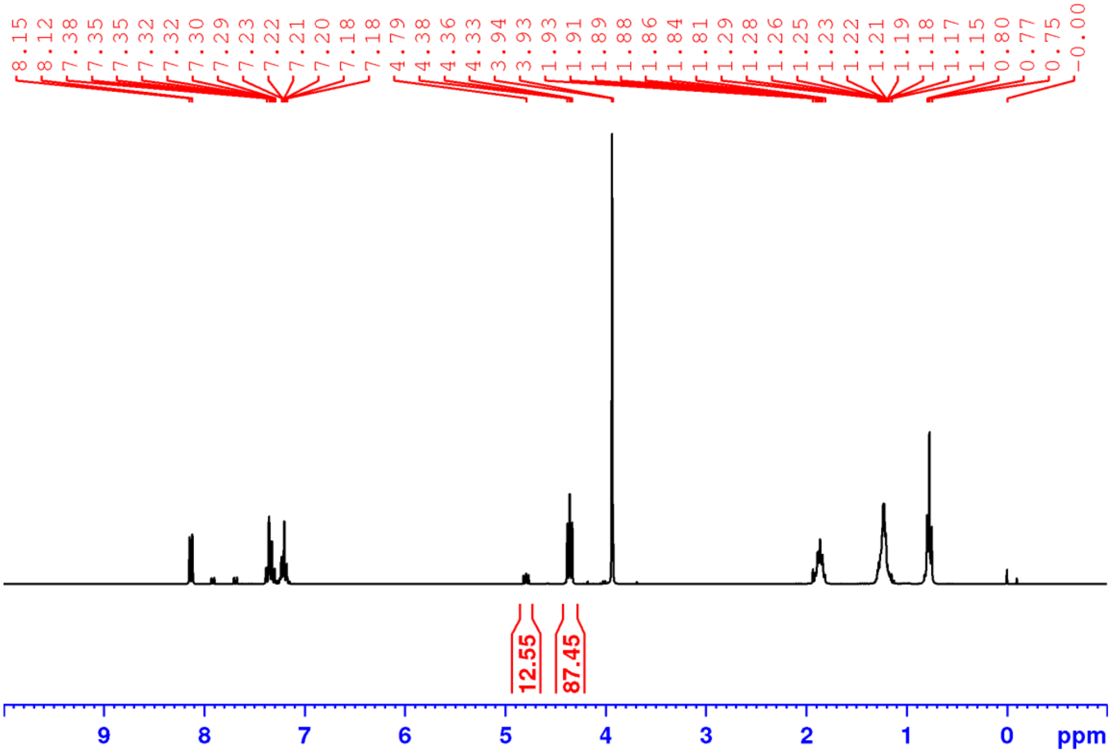

Table 3, Entry 3

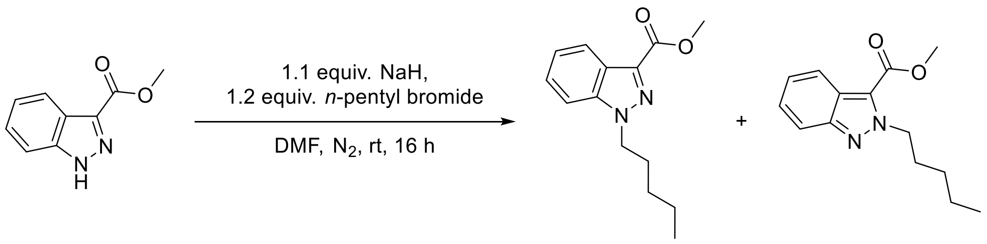

^{1}H NMR (300 MHz, $CDCl_3$)

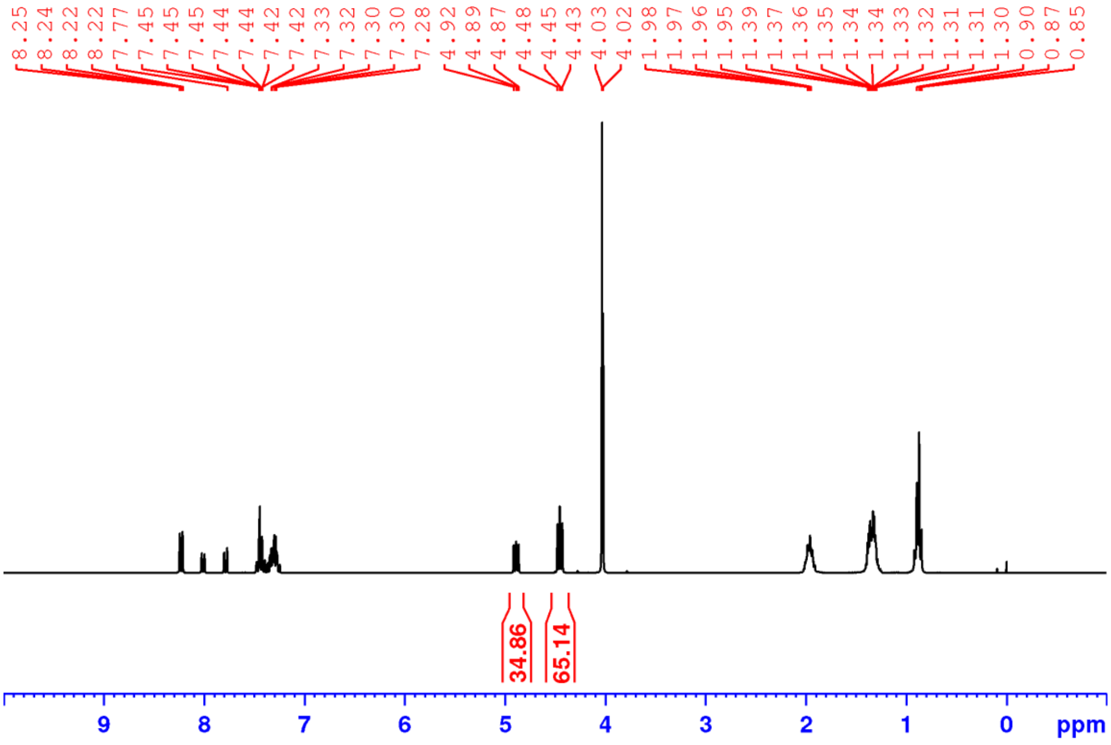

Table 3, Entry 4

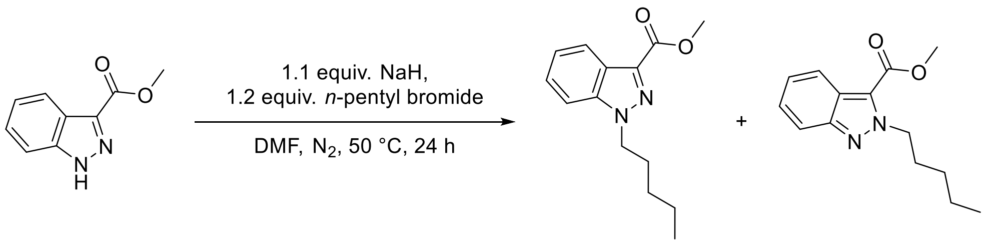

^{1}H NMR (300 MHz, $CDCl_3$)

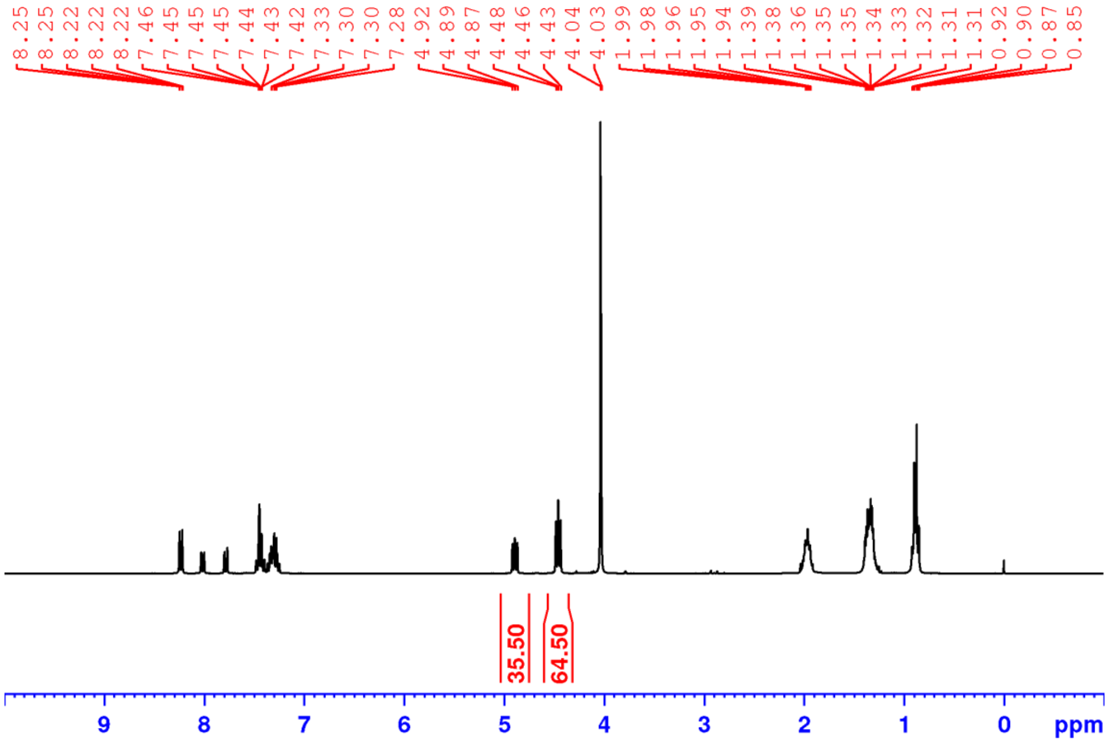

Table 3, Entry 5

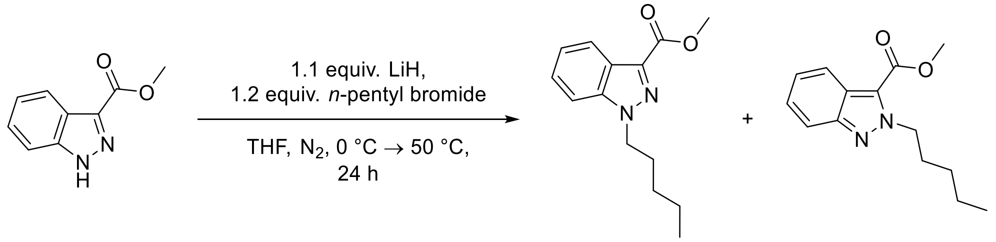

9 **10** **11**

^{1}H NMR (300 MHz, $CDCl_3$)

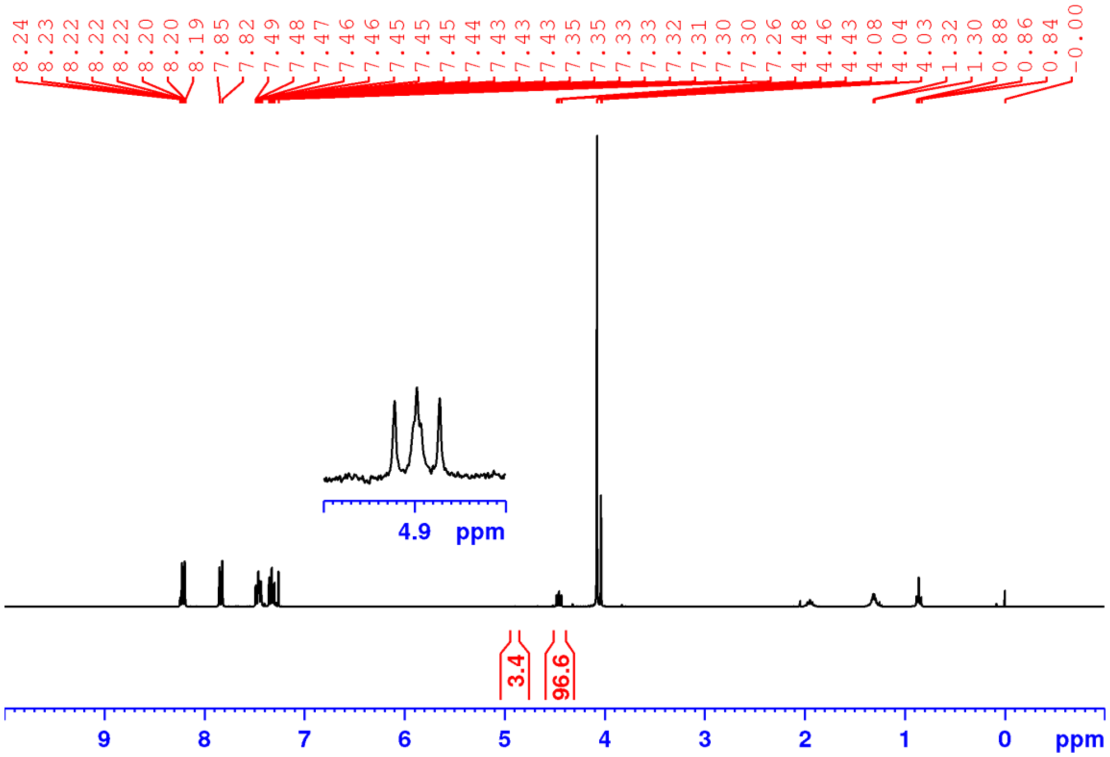

Table 3, Entry 6

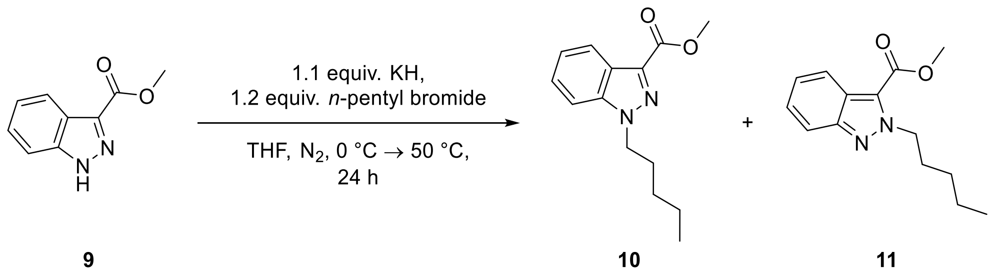

^{1}H NMR (300 MHz, $CDCl_3$)

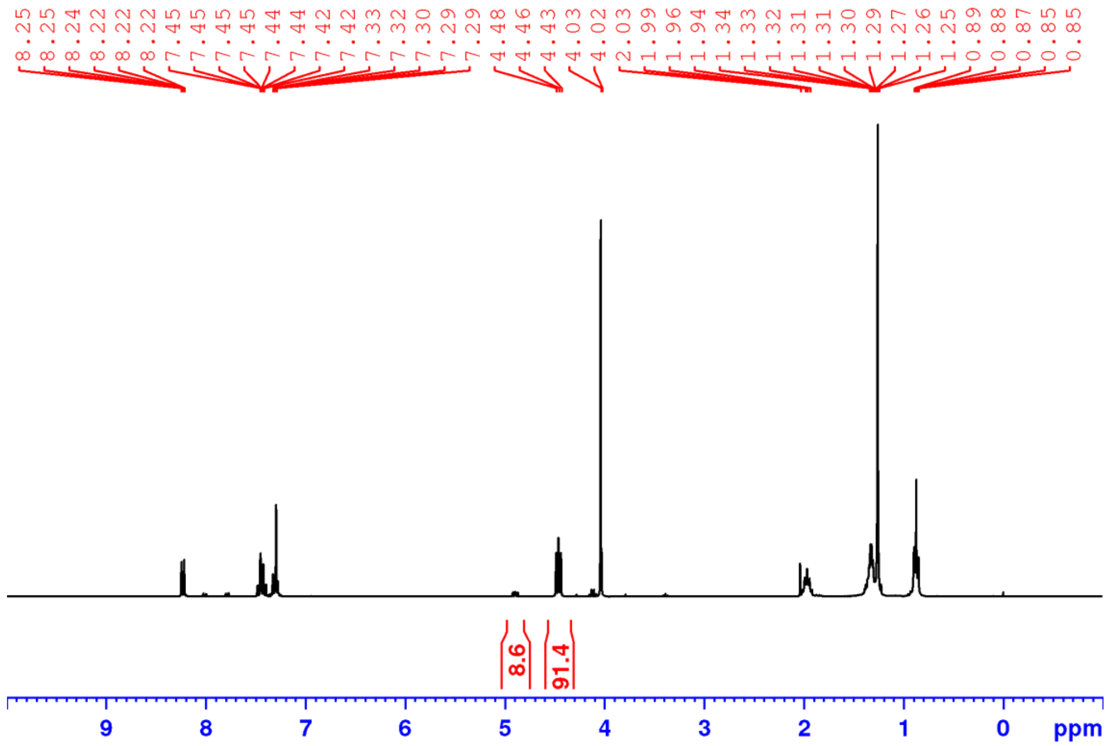

Table 3, Entry 7

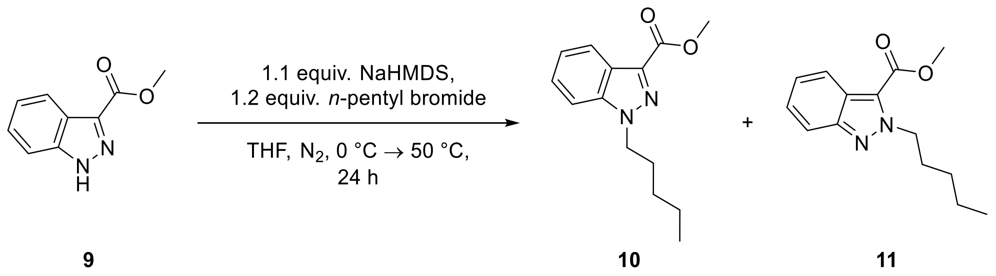

^{1}H NMR (300 MHz, $CDCl_3$)

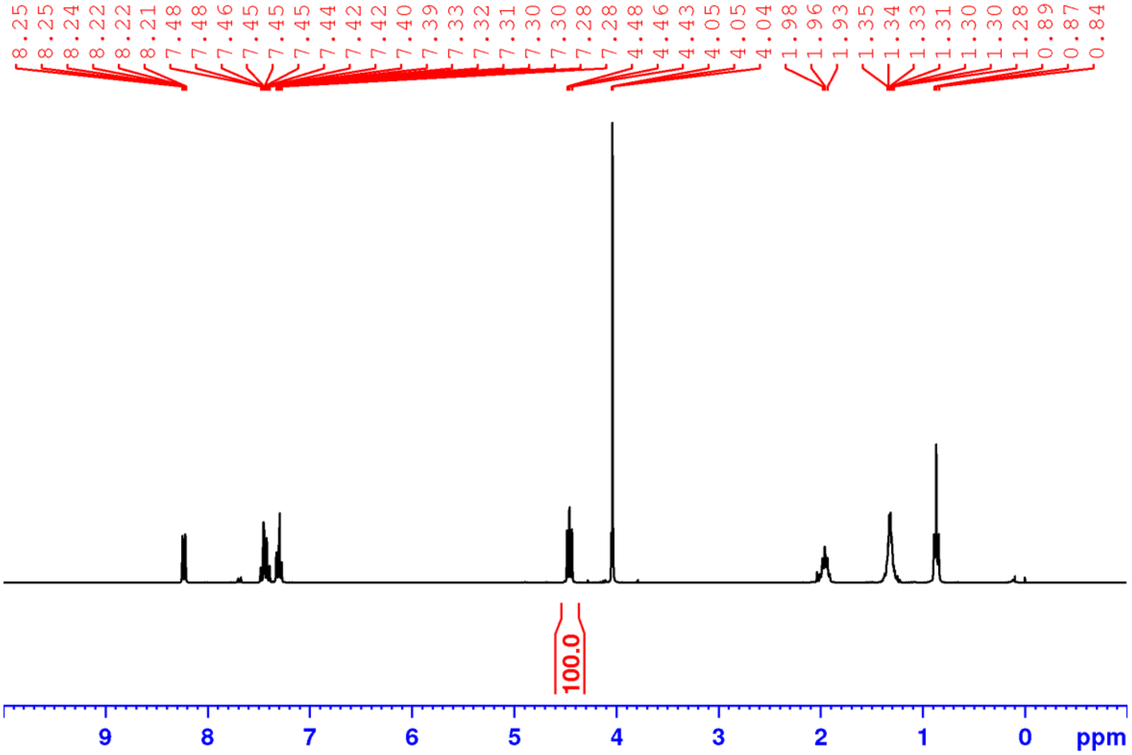

Table 3, Entry 8

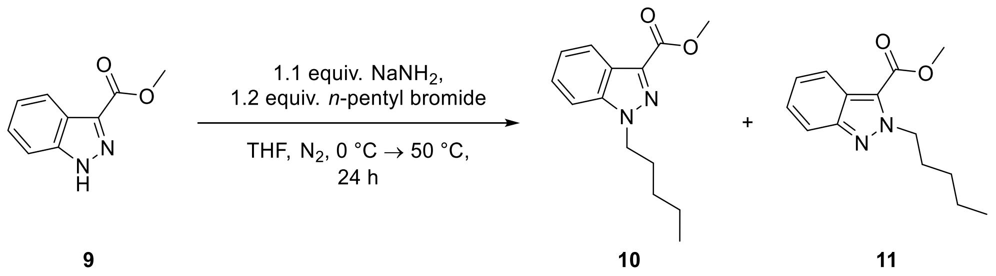

^{1}H NMR (300 MHz, $CDCl_3$)

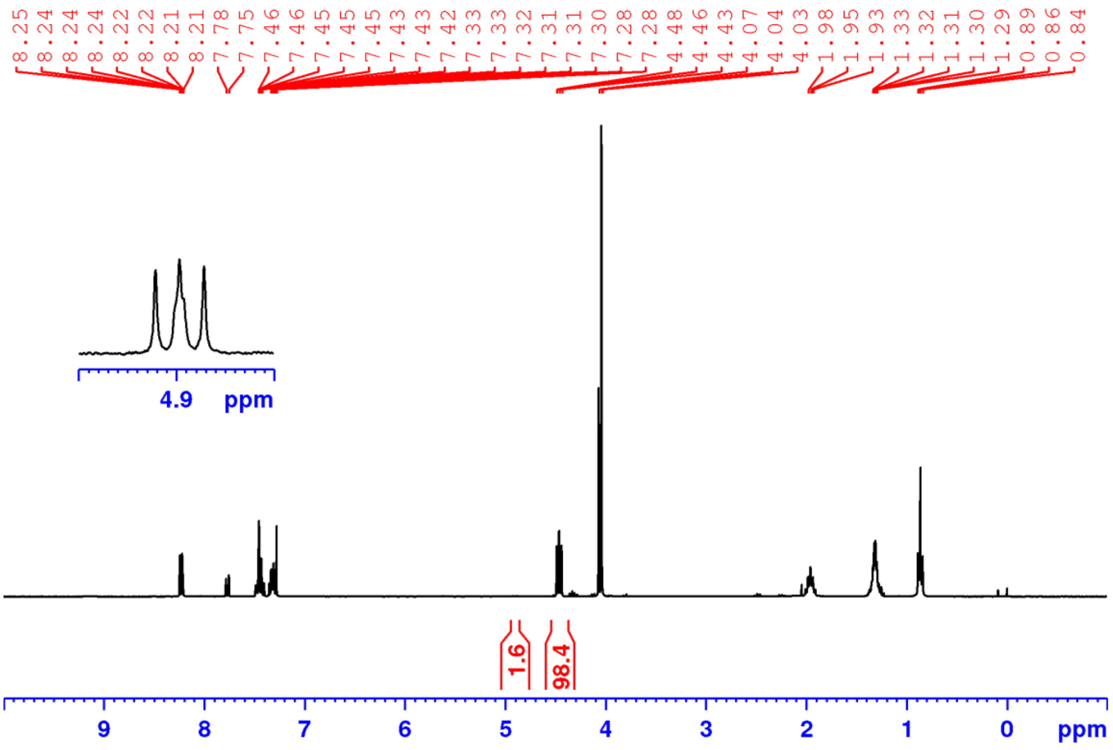

Table 3, Entry 9

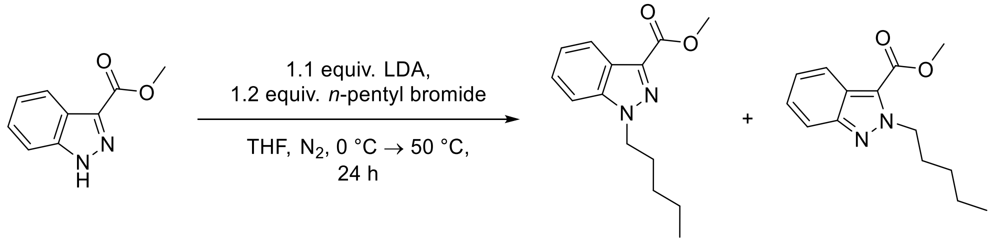

9 10 11

^{1}H NMR (300 MHz, $CDCl_3$)

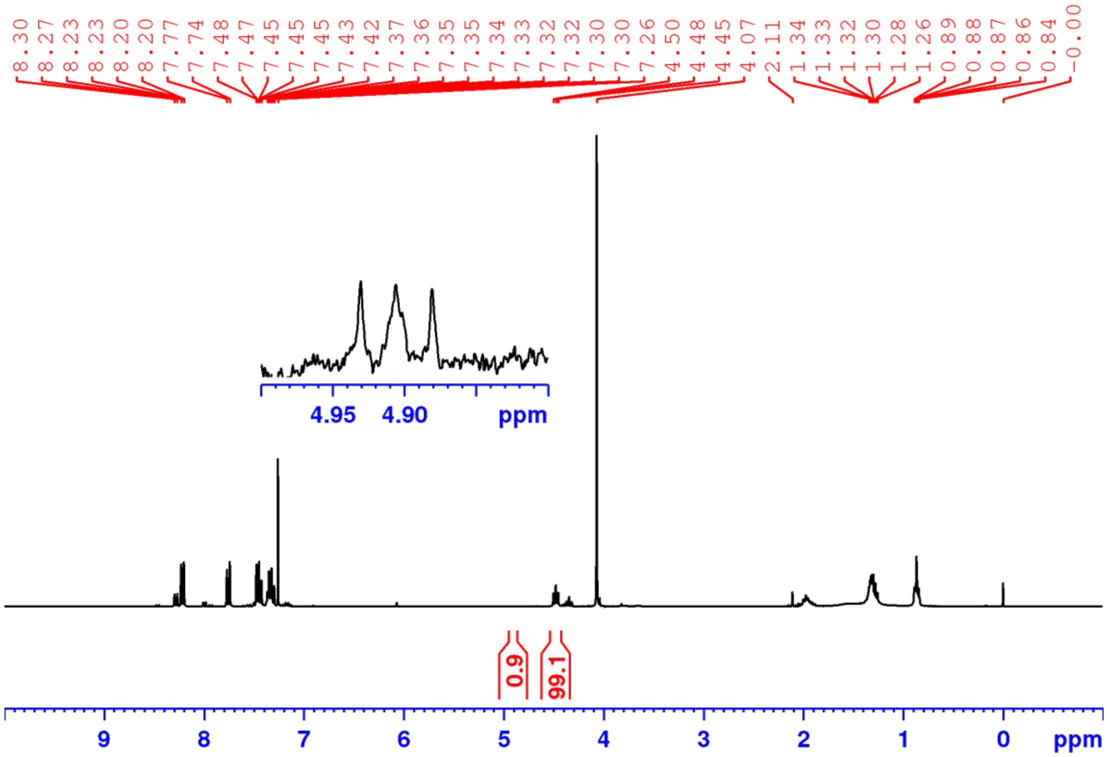

Table 4, Entry 1 (Conditions A)

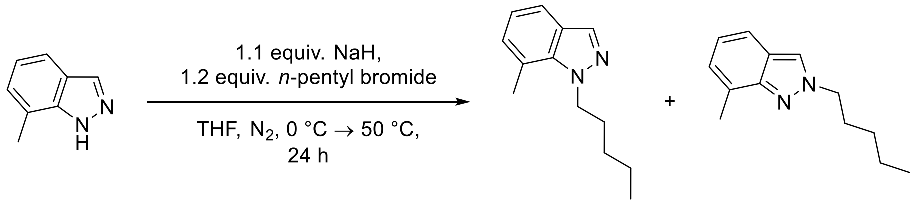

^1H NMR (300 MHz, $CDCl_3$)

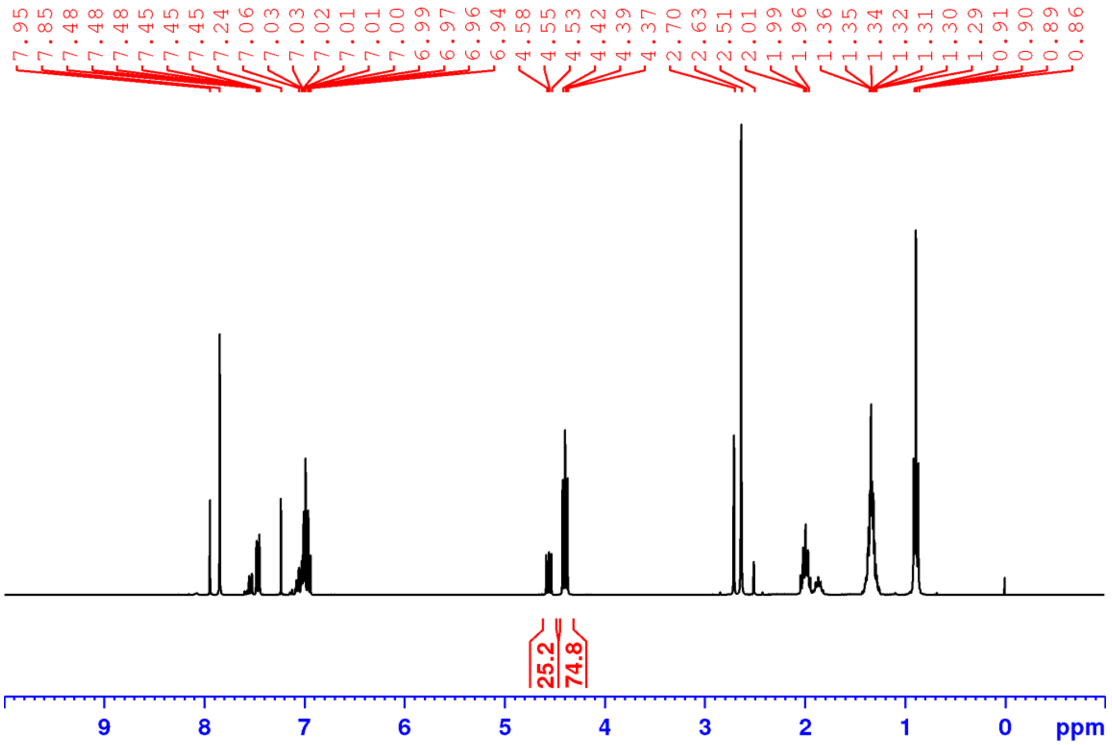

Table 4, Entry 1 (Conditions B)

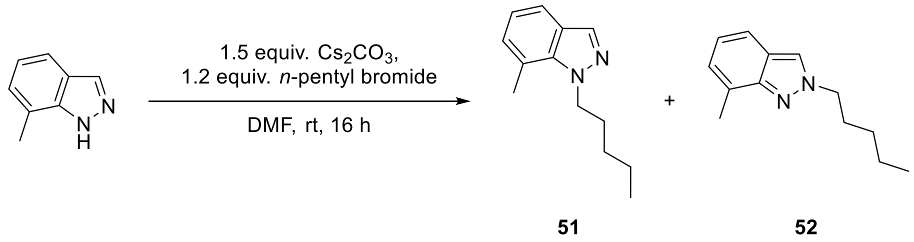

^1H NMR (300 MHz, $CDCl_3$)

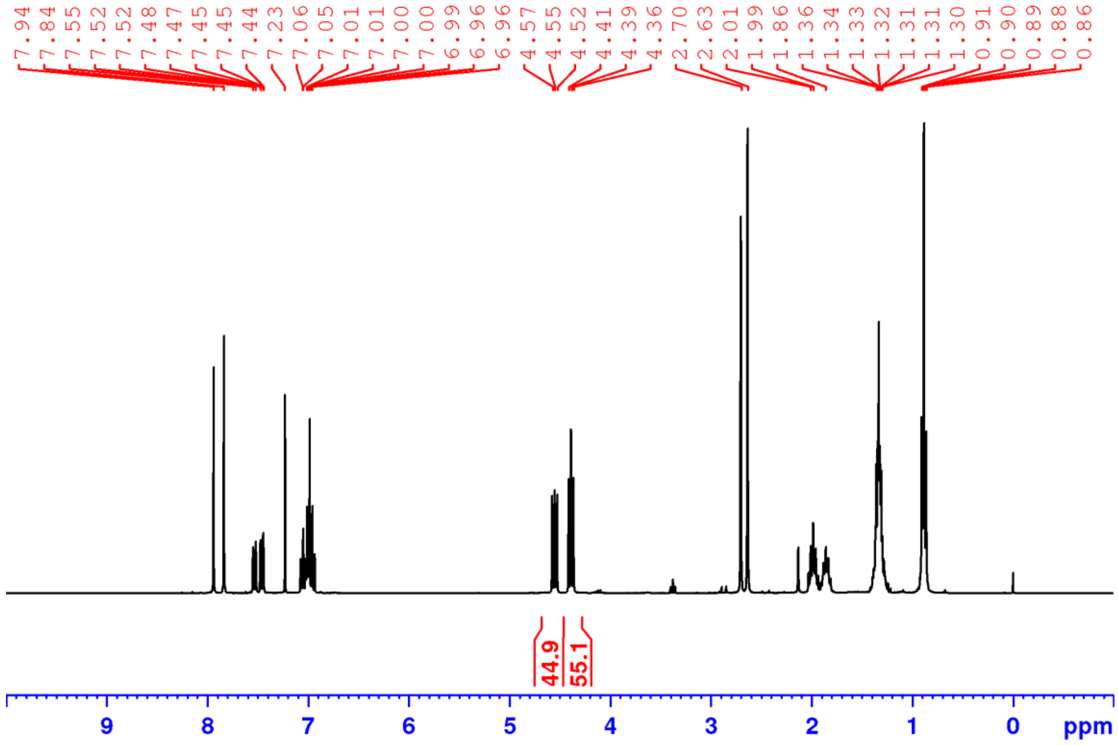

Table 4, Entry 2 (Conditions A)

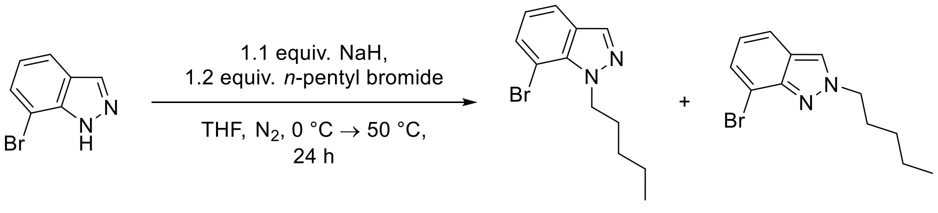

^{1}H NMR (300 MHz, $CDCl_3$)

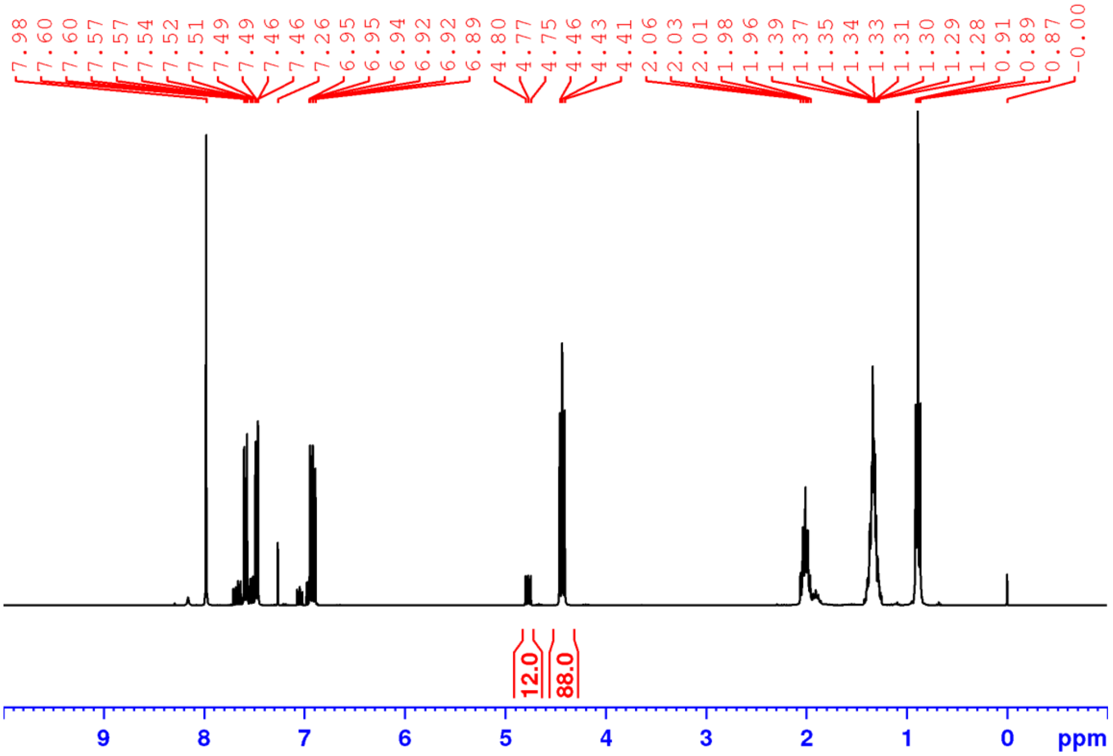

Table 4, Entry 2 (Conditions B)

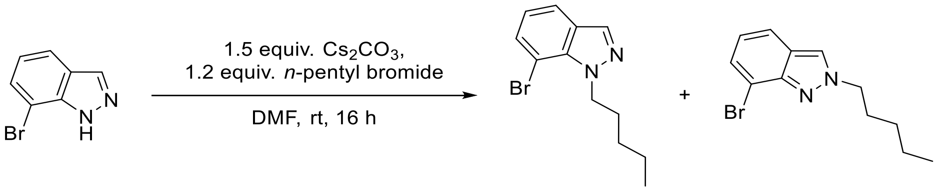

^{1}H NMR (300 MHz, $CDCl_3$)

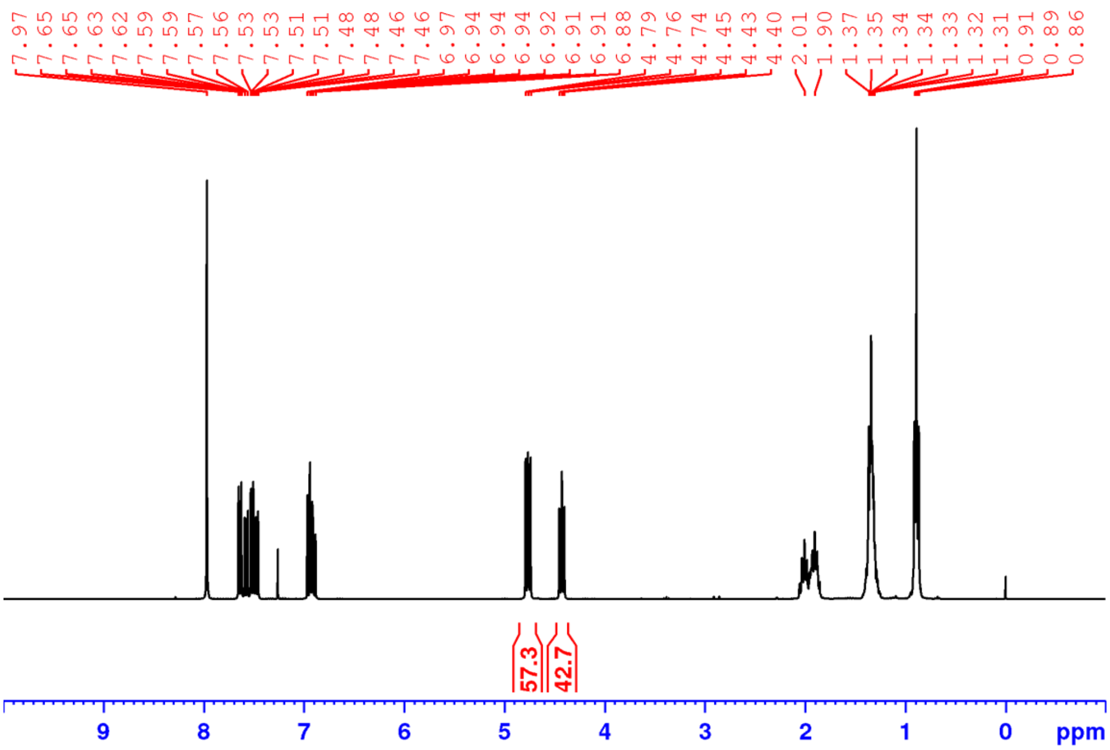

Table 4, Entry 3 (Conditions A)

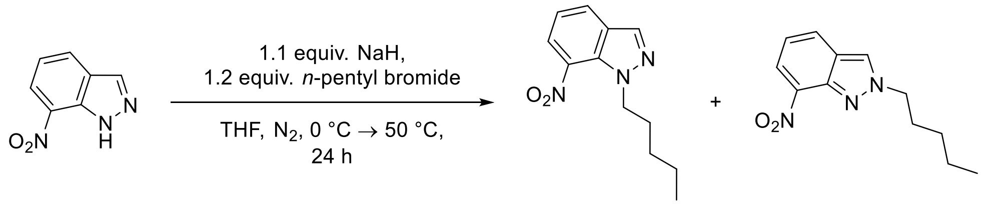

^{1}H NMR (300 MHz, $CDCl_3$)

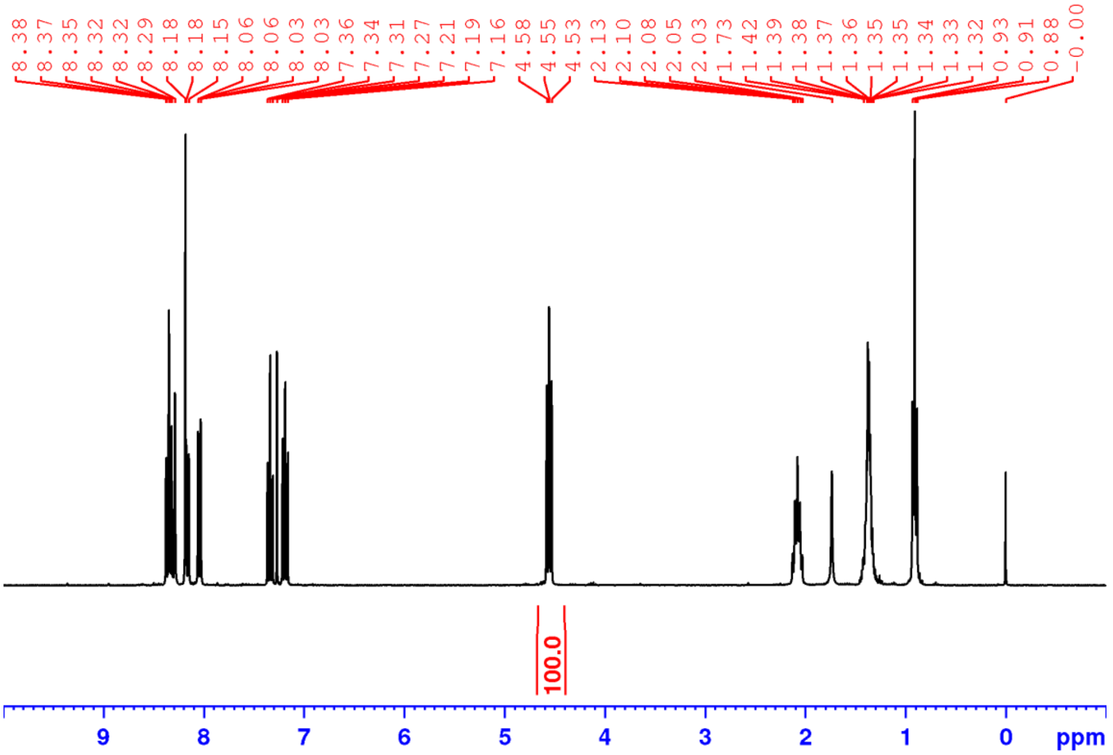

Table 4, Entry 3 (Conditions B)

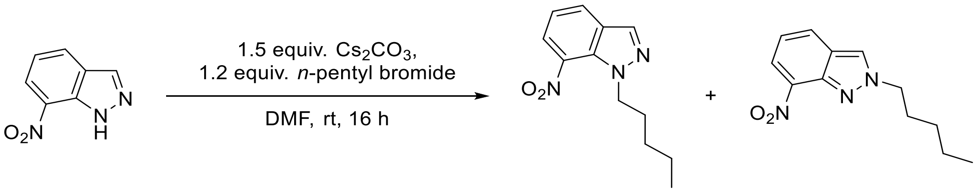

^{1}H NMR (300 MHz, $CDCl_3$)

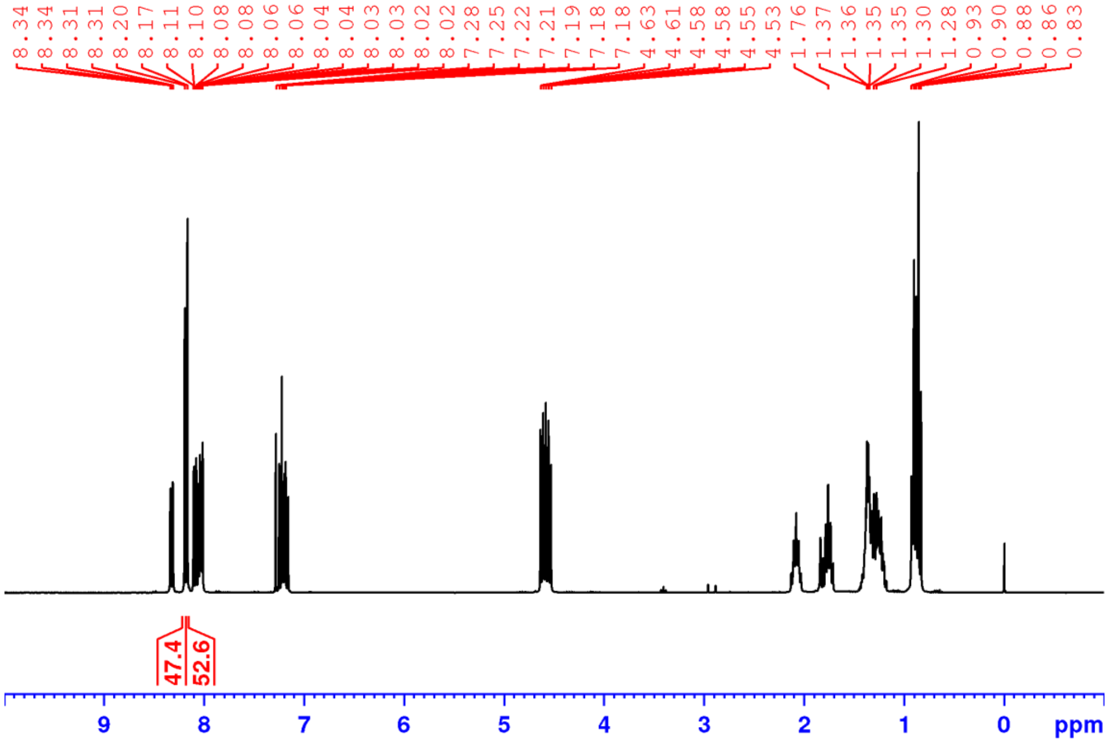

Table 4, Entry 4 (Conditions A)

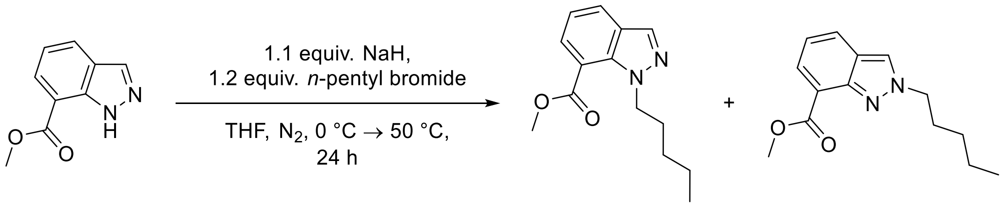

^{1}H NMR (300 MHz, $CDCl_3$)

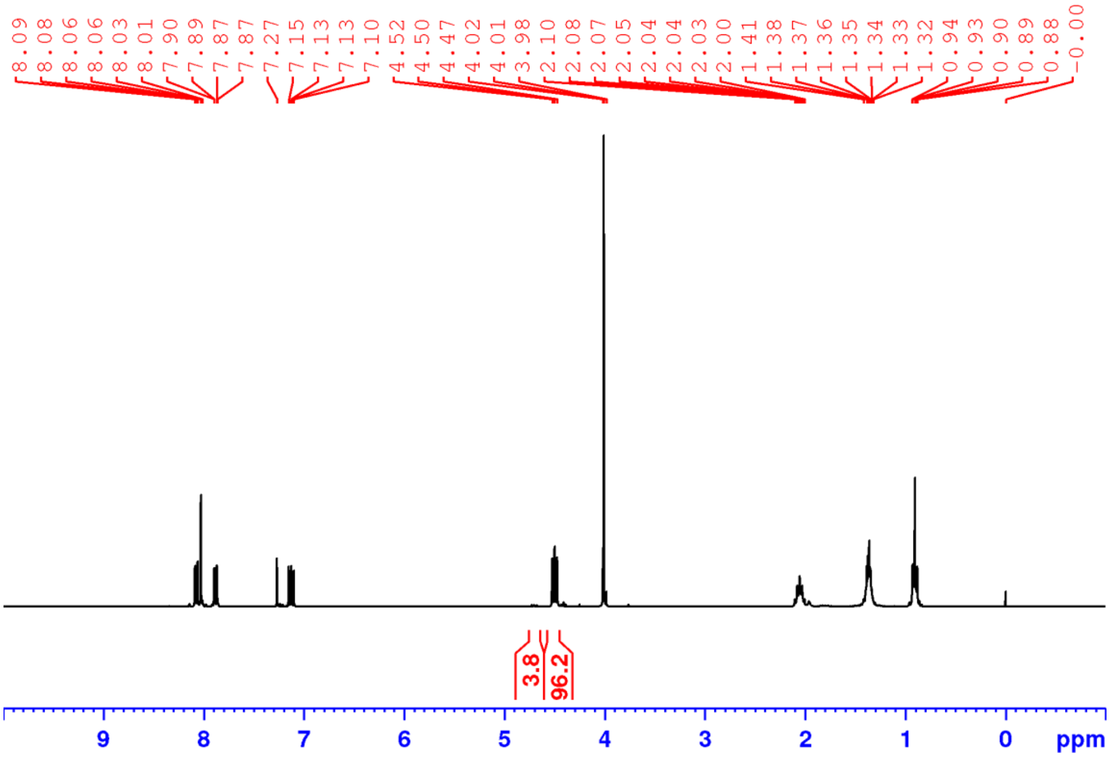

Table 4, Entry 4 (Conditions B)

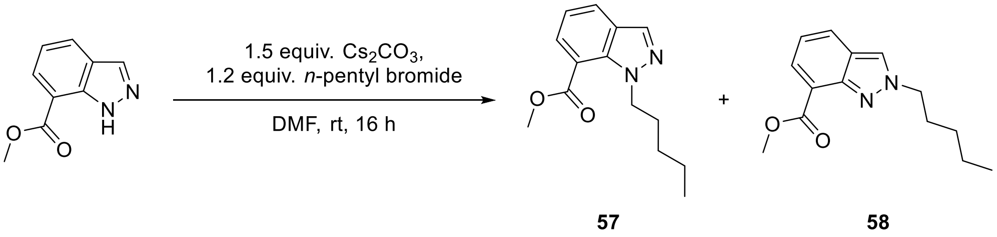

^{1}H NMR (300 MHz, $CDCl_3$)

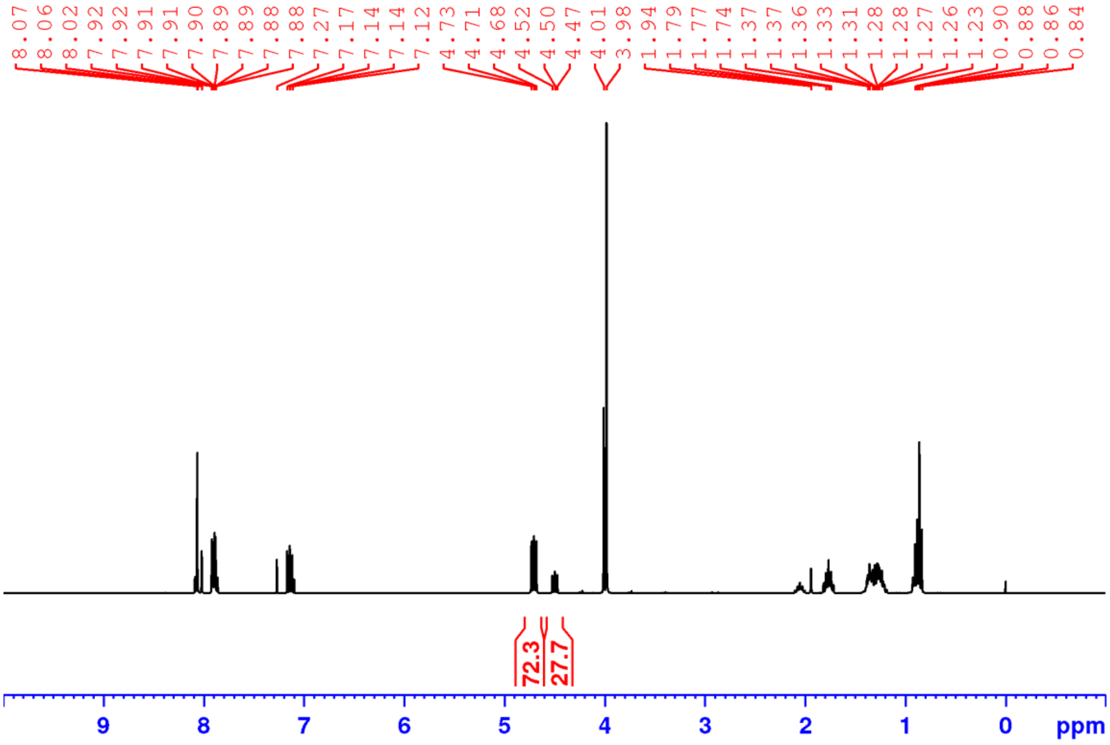

Table 4, Entry 5 (Conditions A)

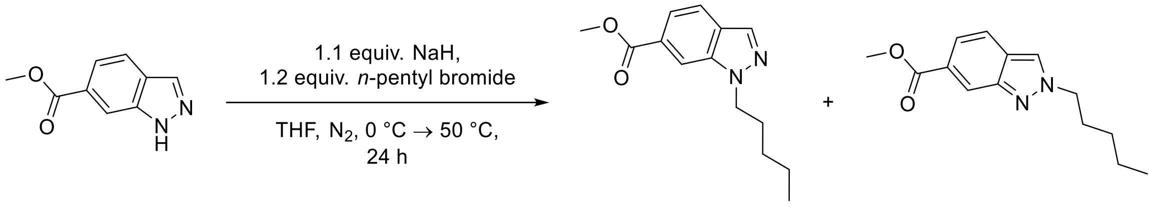

^{1}H NMR (300 MHz, $CDCl_3$)

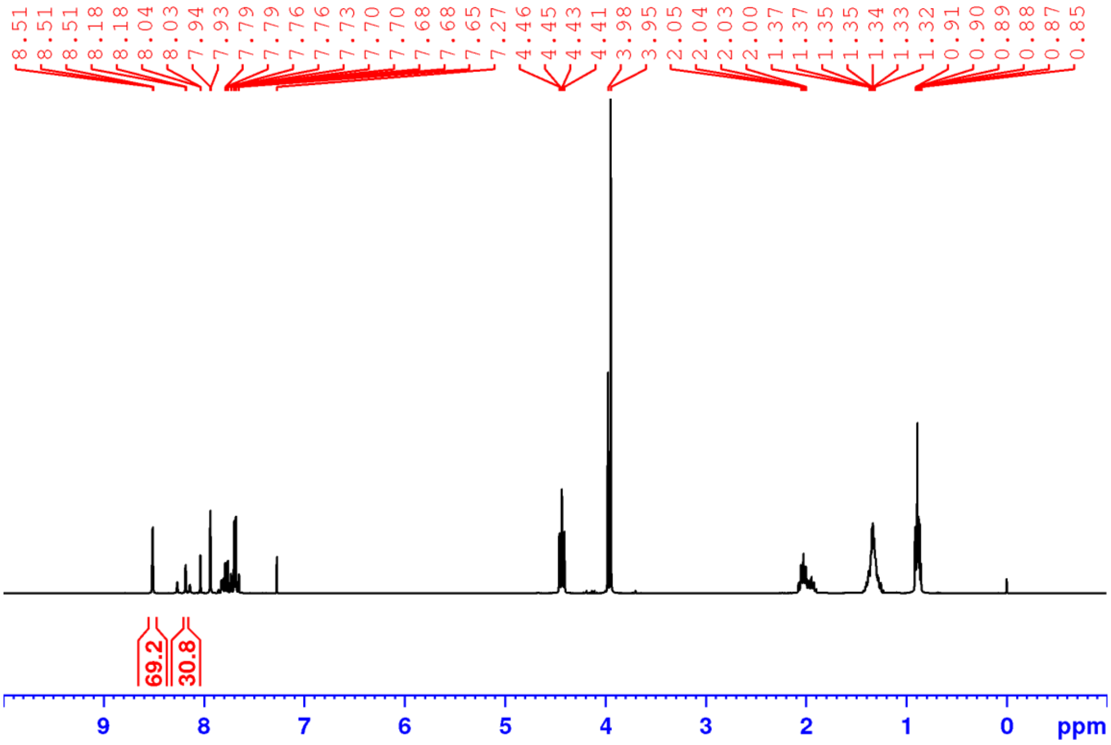

Table 4, Entry 5 (Conditions B)

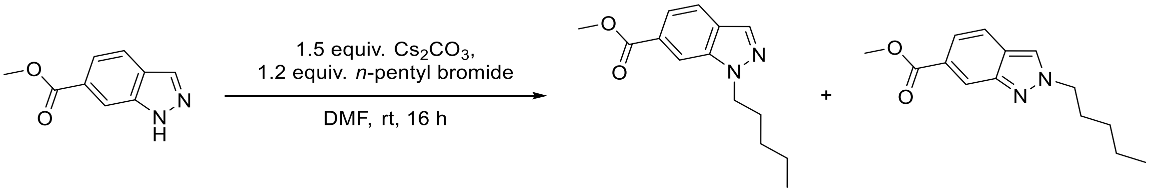

59 **60**

^{1}H NMR (300 MHz, $CDCl_3$)

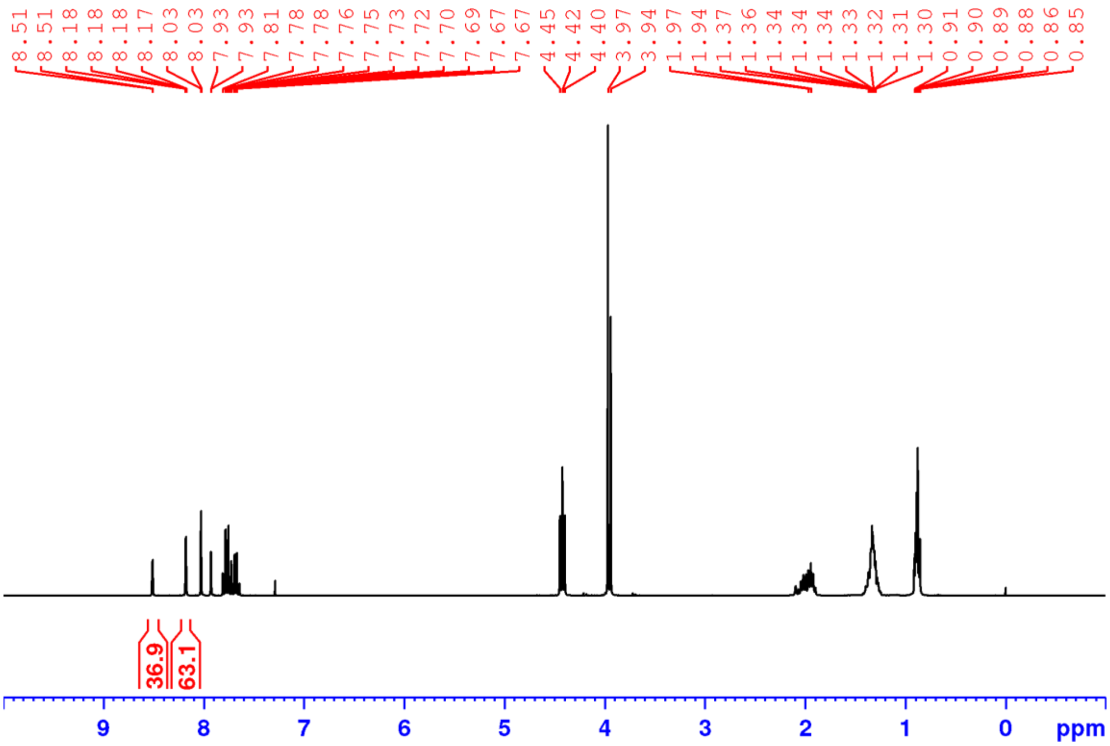

Table 4, Entry 6 (Conditions A)

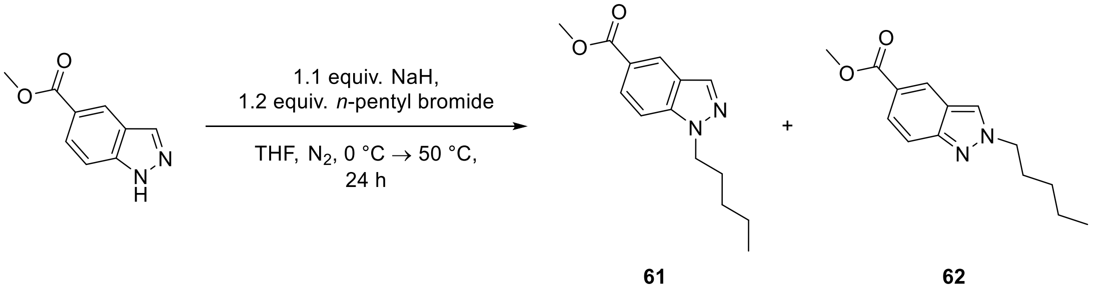

^{1}H NMR (300 MHz, $CDCl_3$)

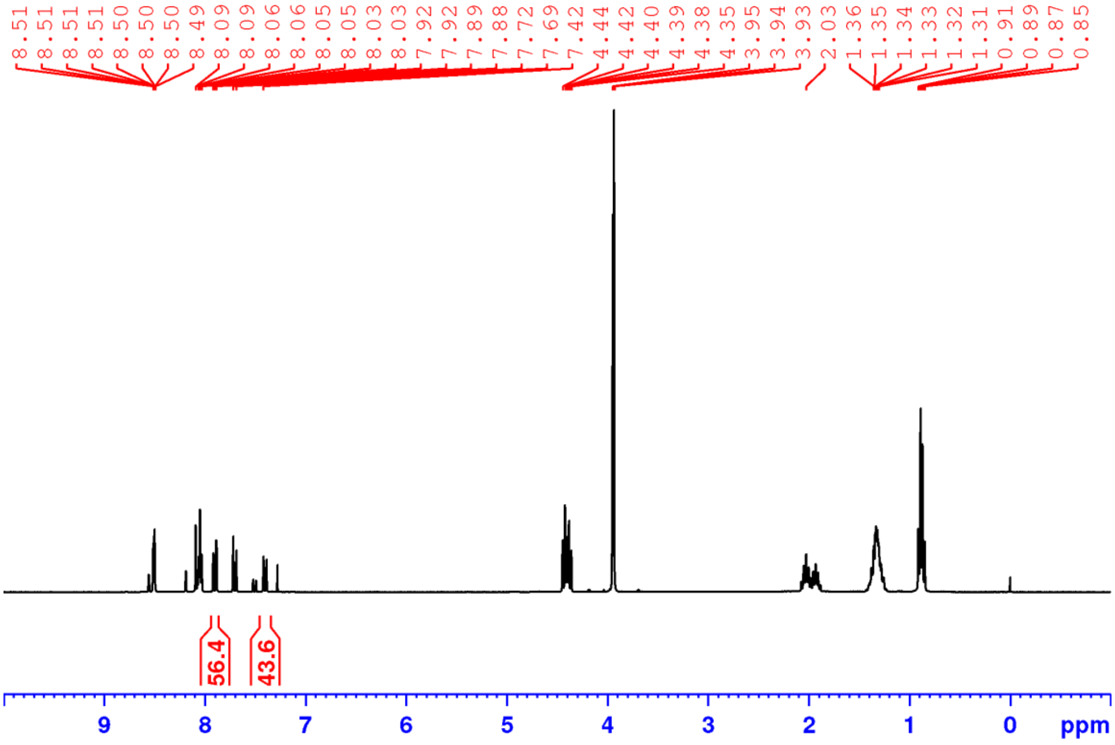

Table 4, Entry 6 (Conditions B)

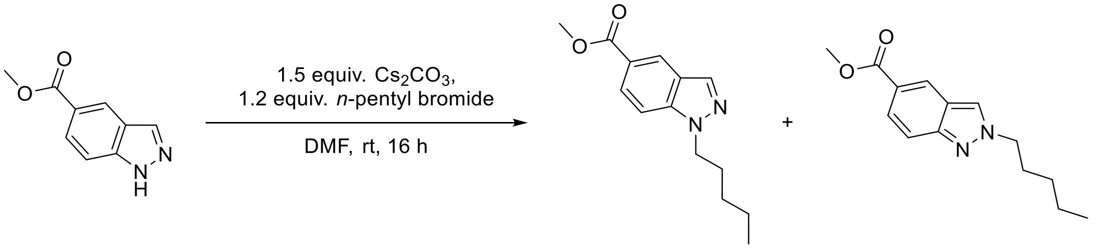

61 **62**

^{1}H NMR (300 MHz, $CDCl_3$)

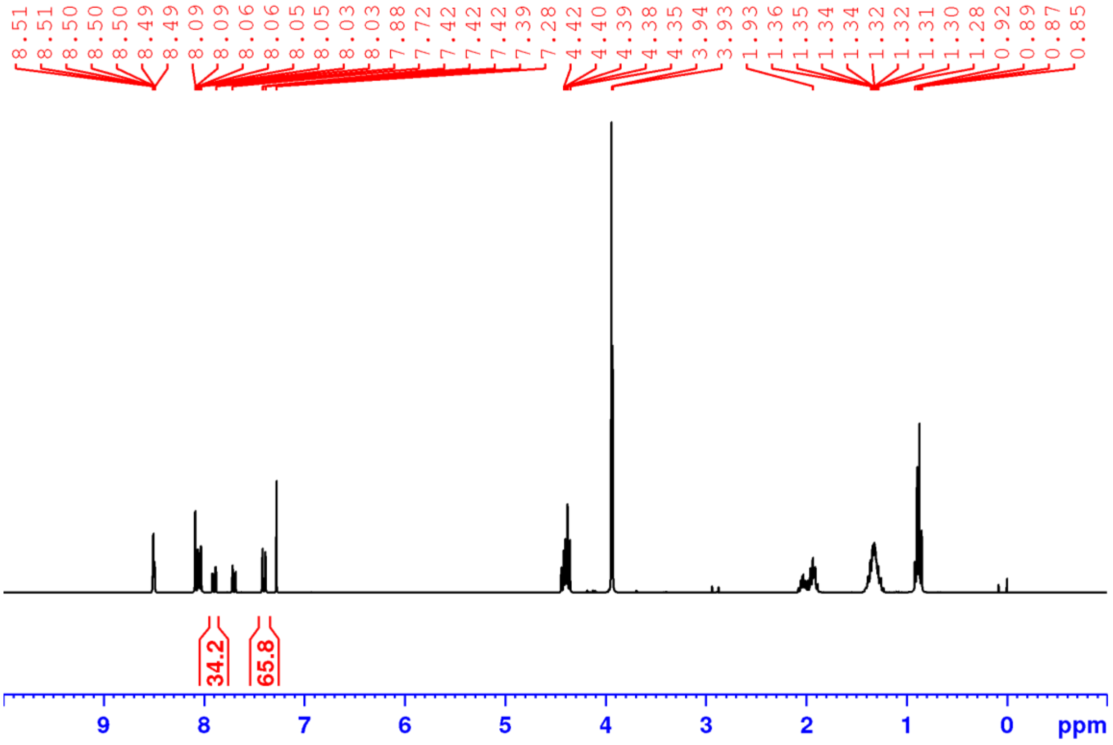

Table 4, Entry 7 (Conditions A)

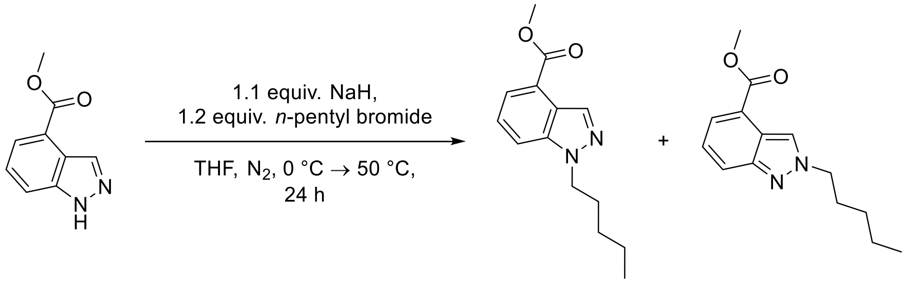

63

64

^{1}H NMR (300 MHz, $CDCl_3$)

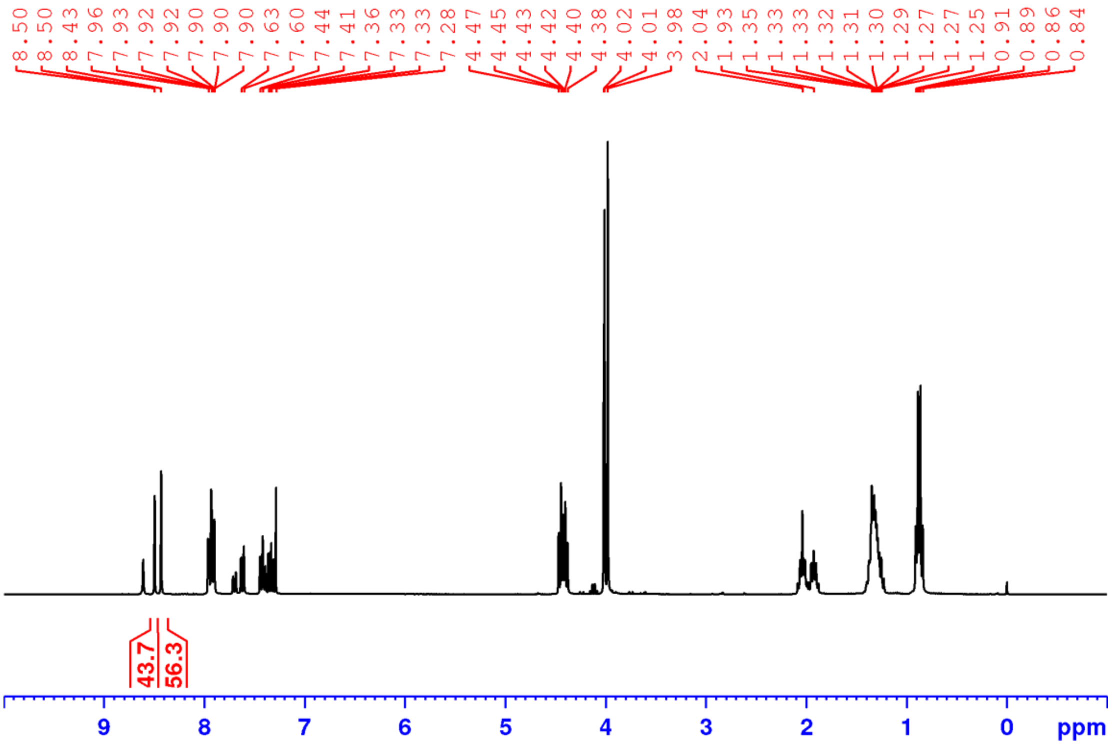

Table 4, Entry 7 (Conditions B)

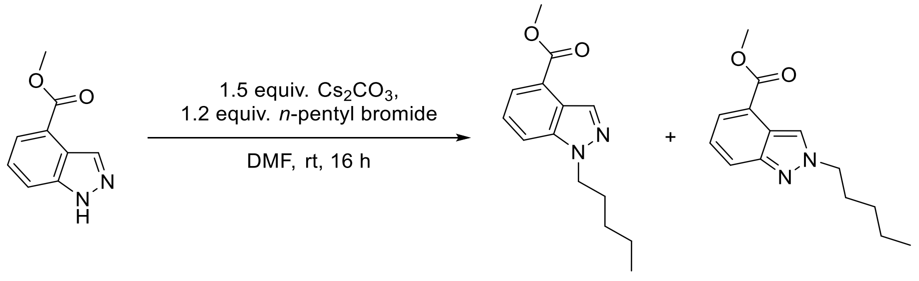

^{1}H NMR (300 MHz, $CDCl_3$)

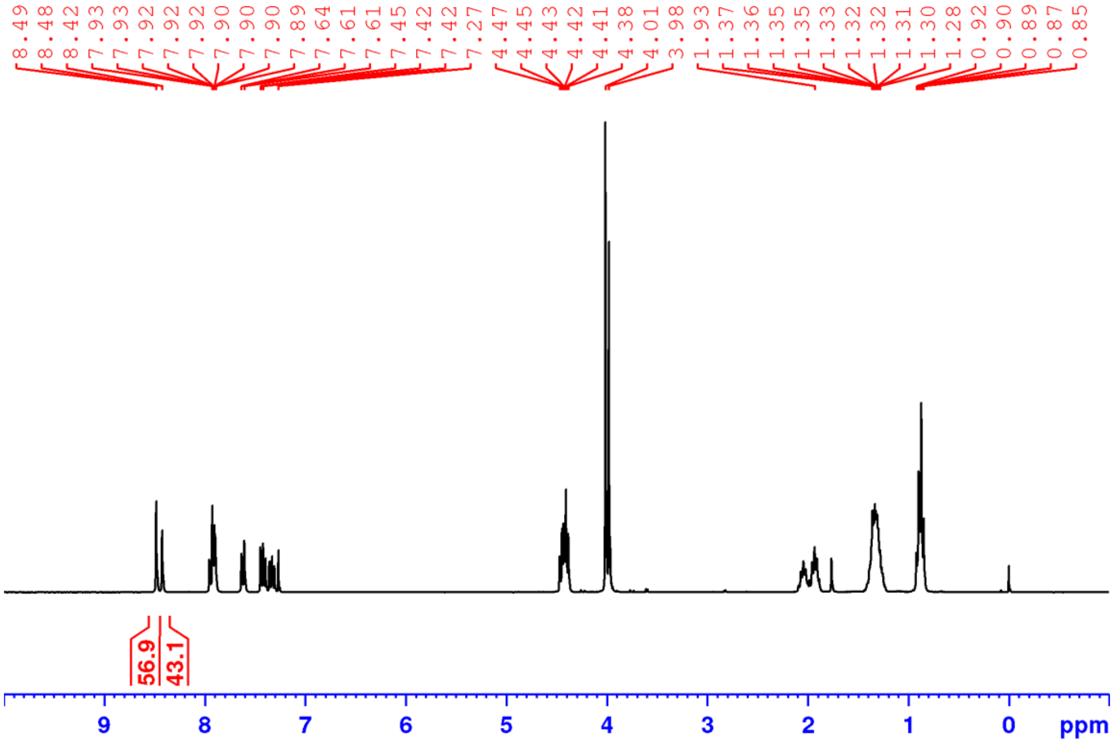

Table 5, Entry 1

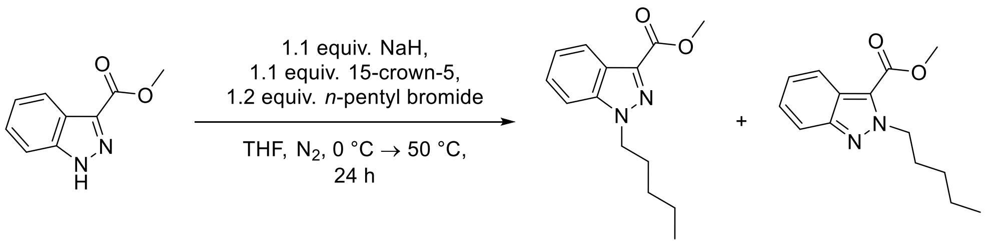

^{1}H NMR (300 MHz, $CDCl_3$)

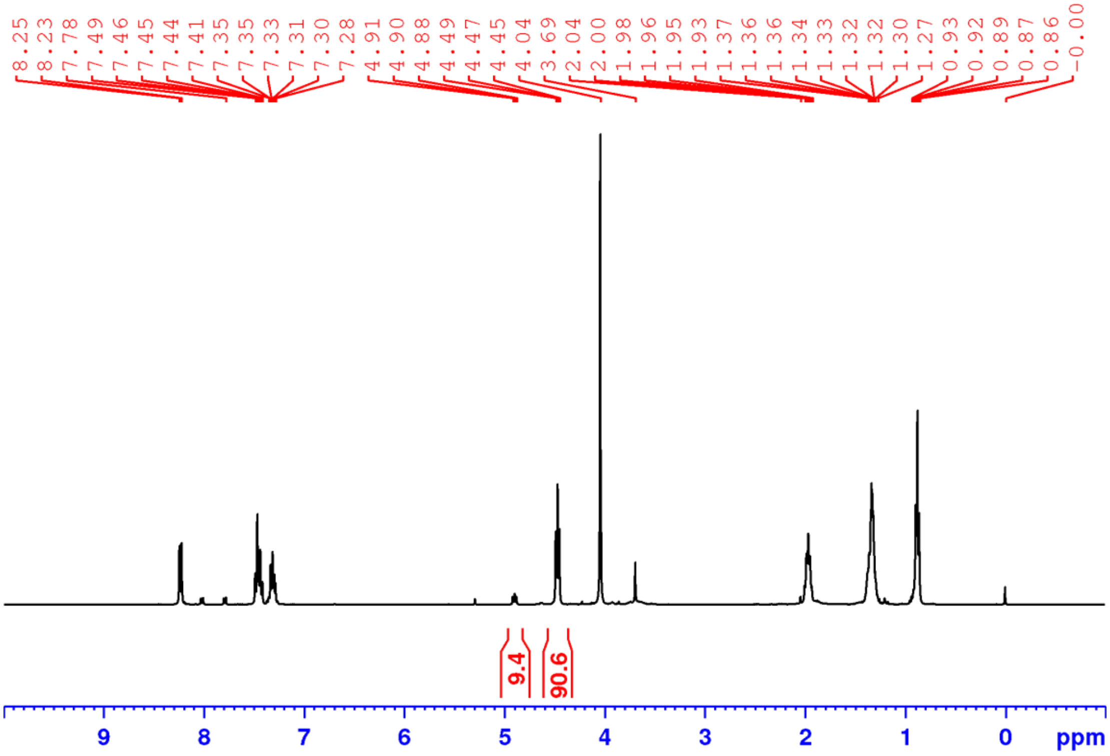

Table 5, Entry 2

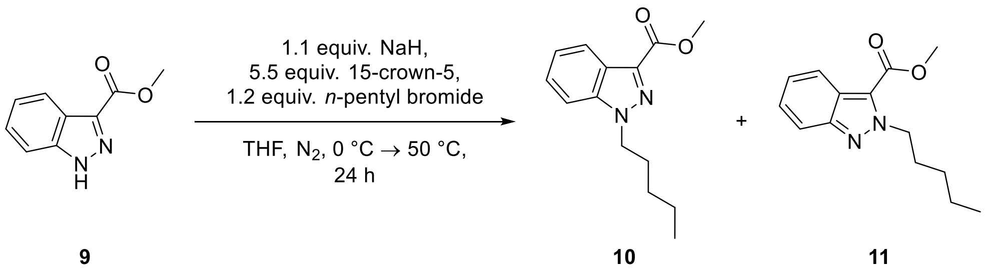

^{1}H NMR (300 MHz, $CDCl_3$)

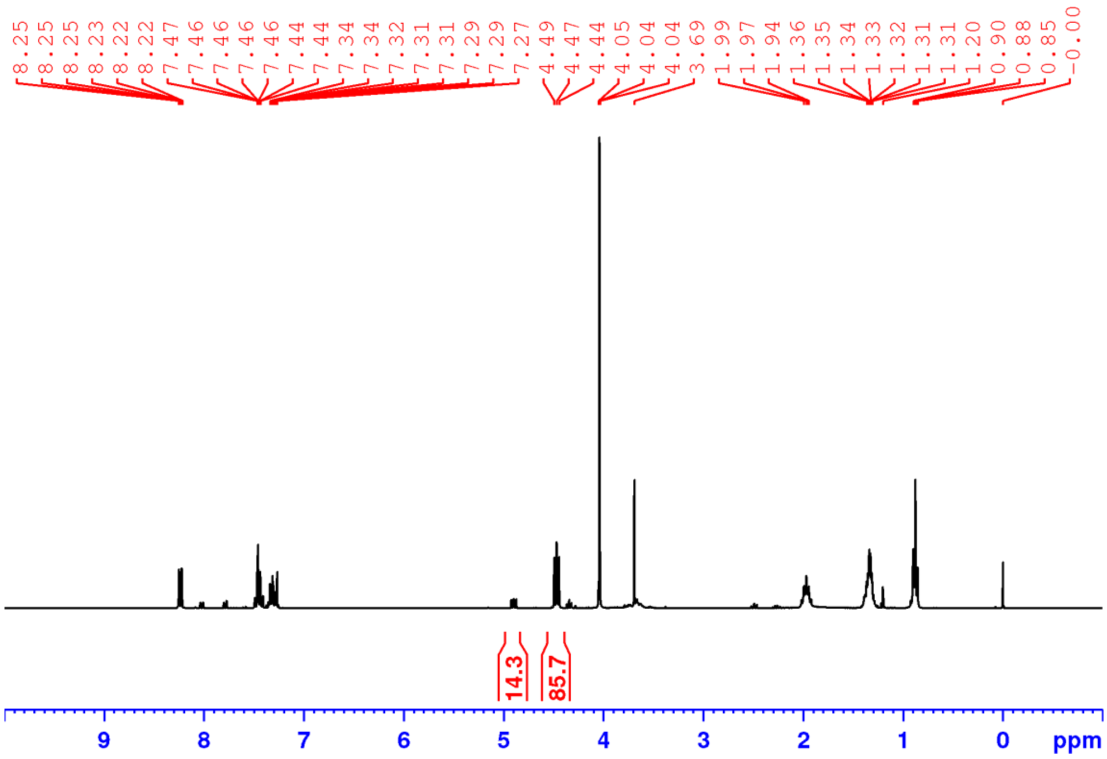

Table 6, Entry 1 (Conditions A)

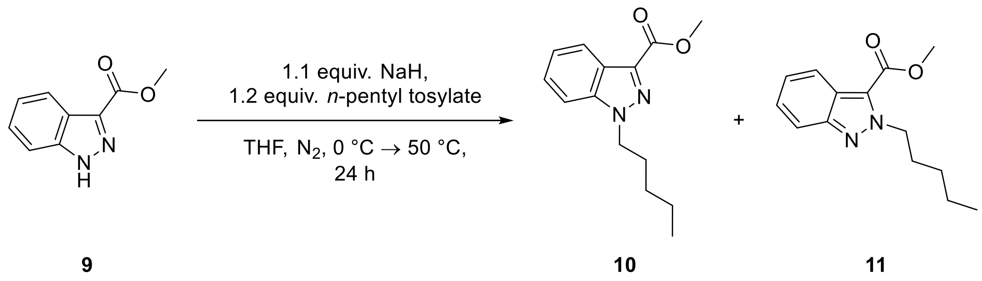

^{1}H NMR (400 MHz, $CDCl_3$)

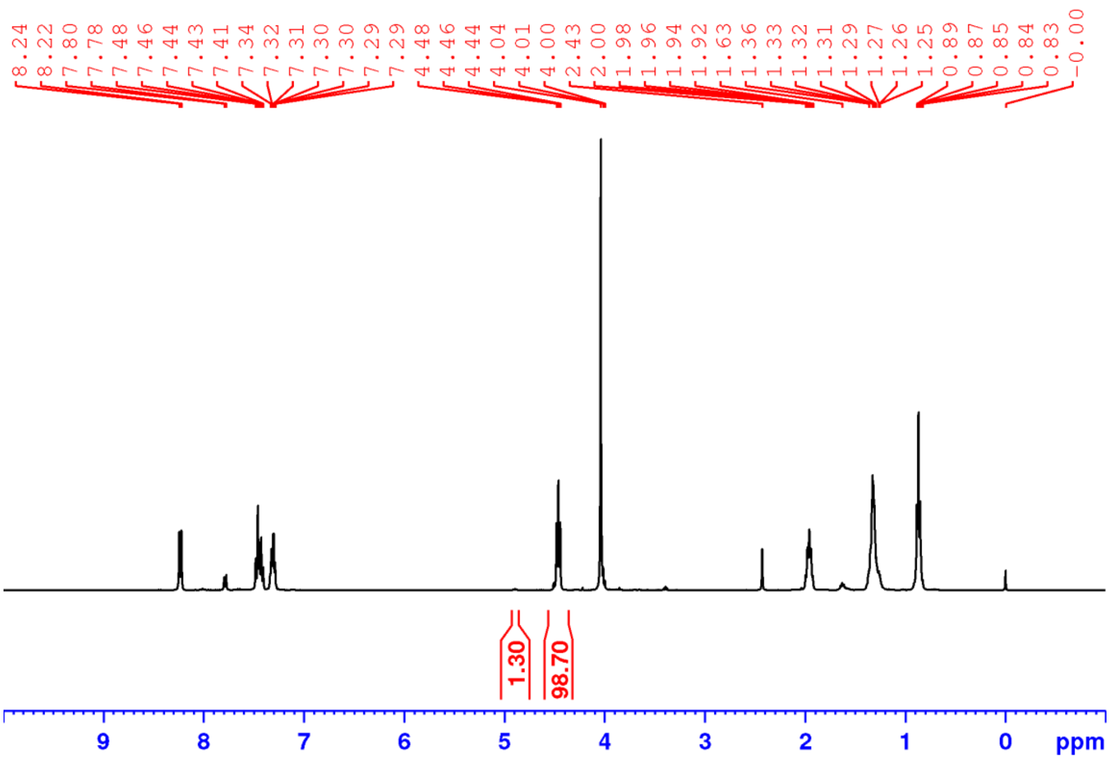

Table 6, Entry 1 (Conditions B)

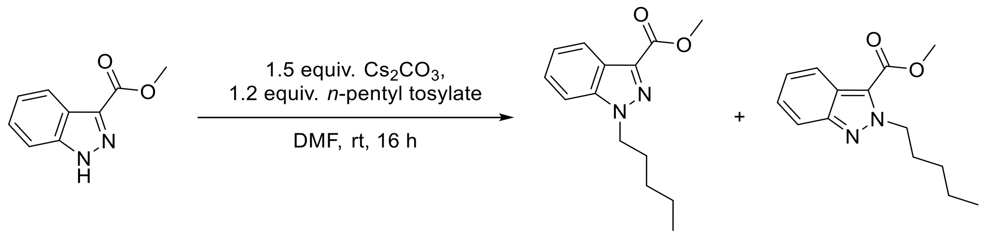

9 **10** **11**

^{1}H NMR (400 MHz, $CDCl_3$)

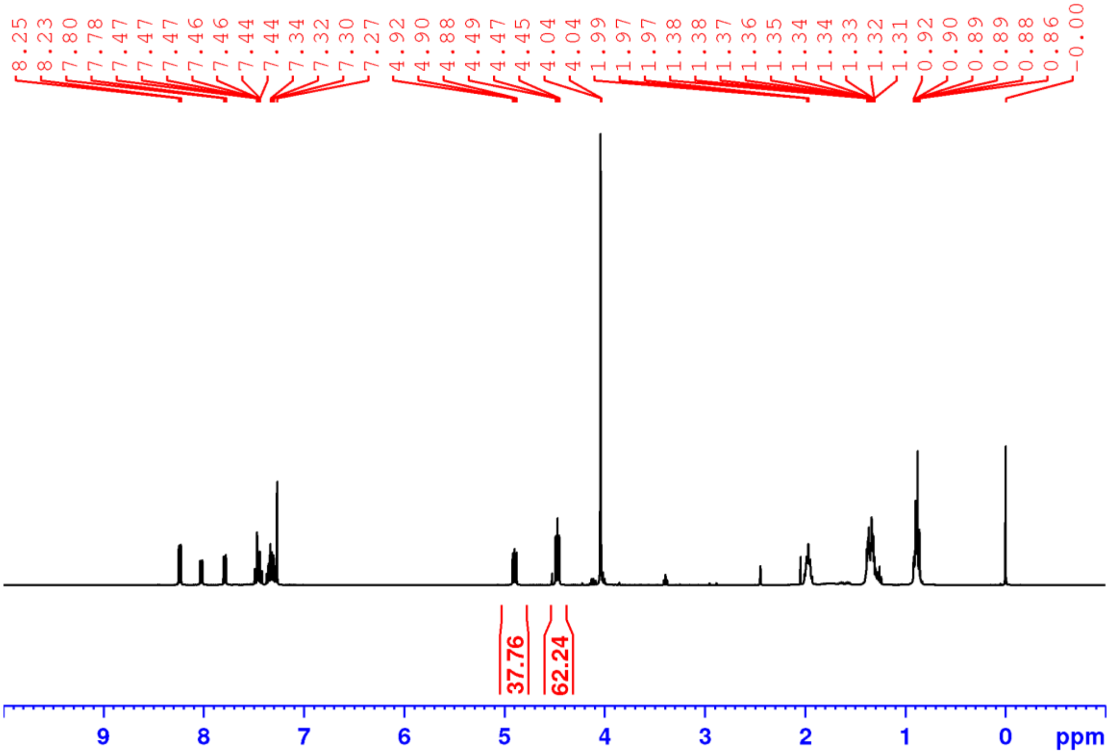

Table 6, Entry 2 (Conditions A)

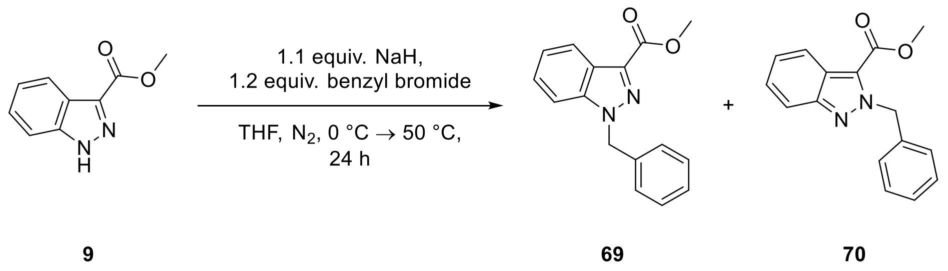

^{1}H NMR (400 MHz, $CDCl_3$)

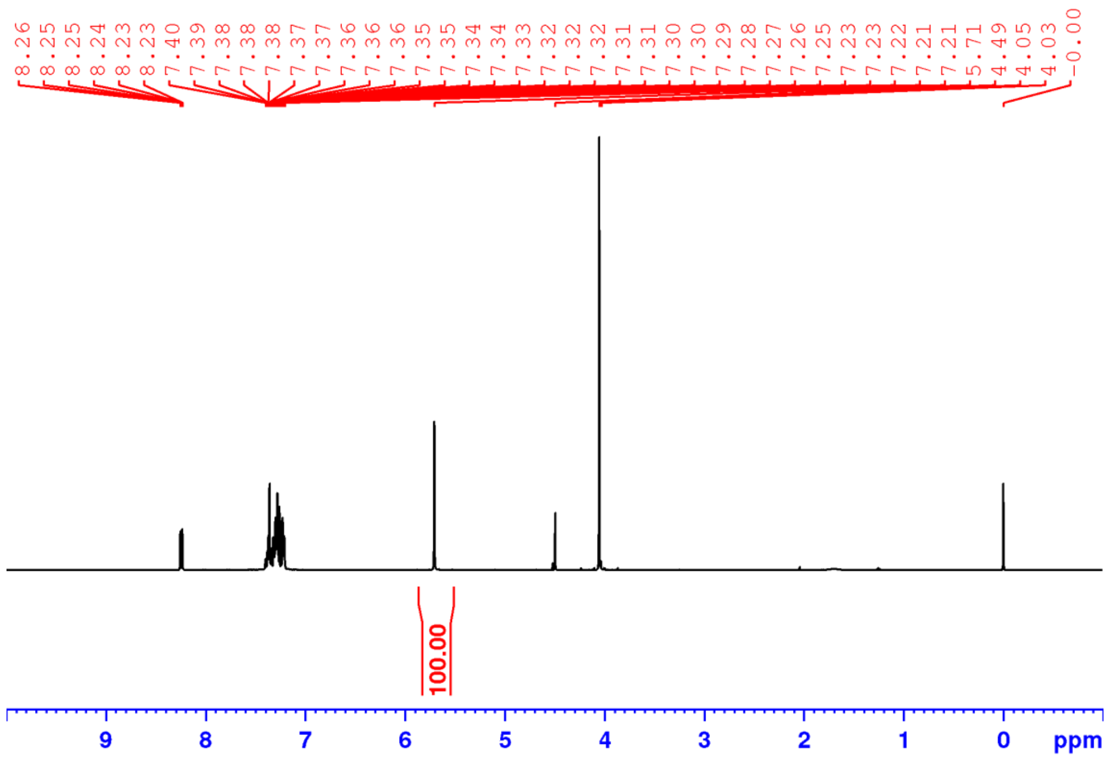

Table 6, Entry 2 (Conditions B)

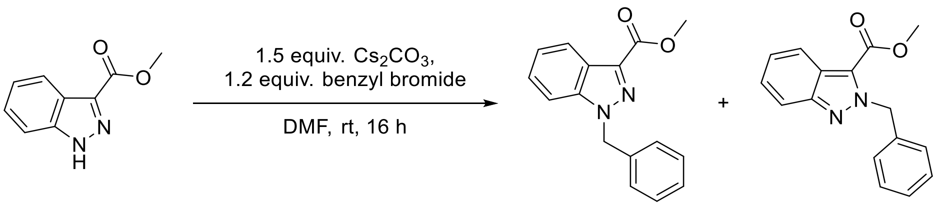

9 69 70

^{1}H NMR (400 MHz, $CDCl_3$)

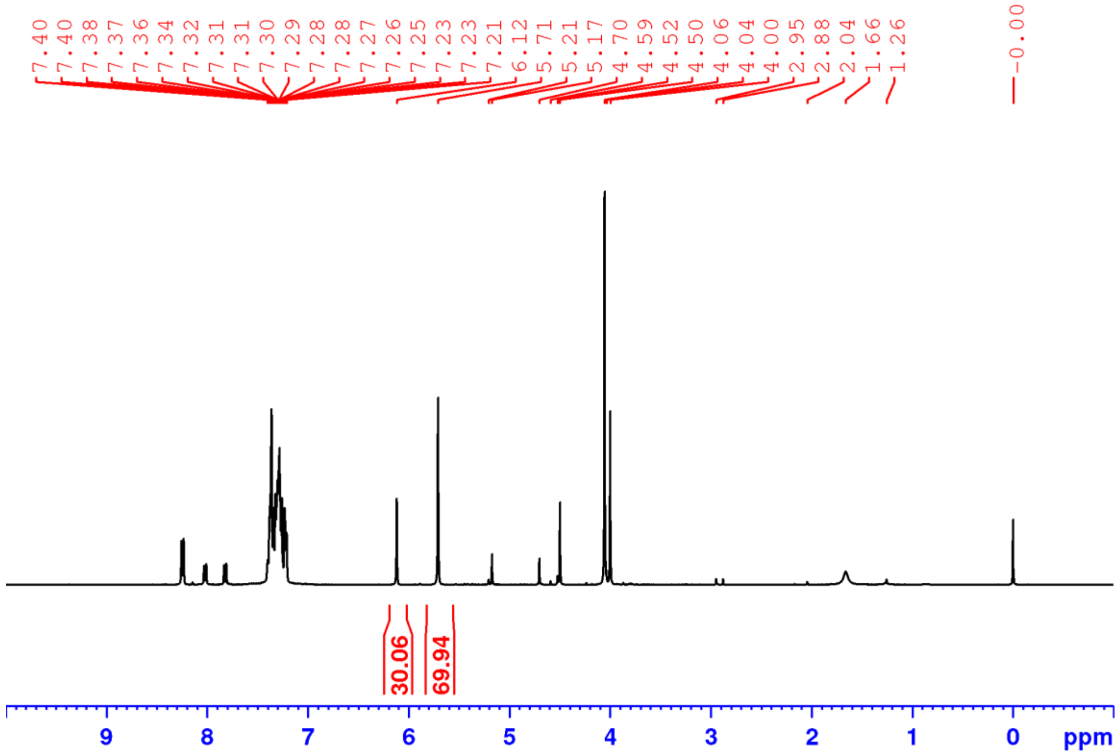

Table 6, Entry 3 (Conditions A)

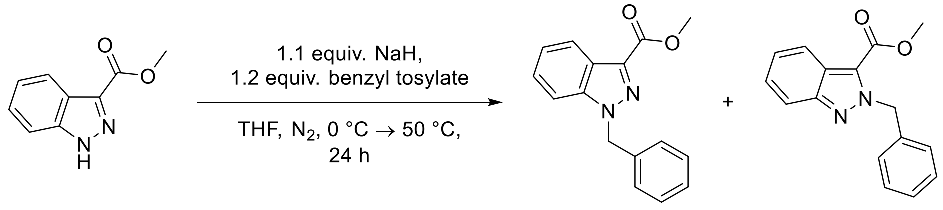

^{1}H NMR (400 MHz, $CDCl_3$)

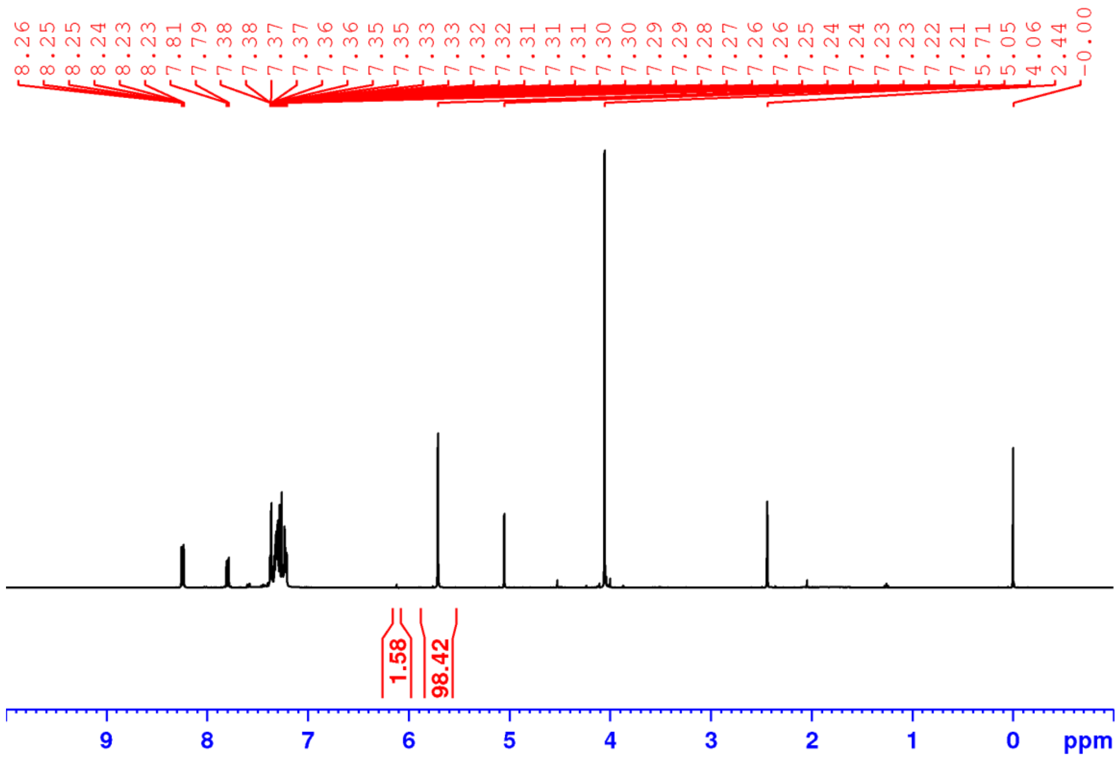

Table 6, Entry 3 (Conditions B)

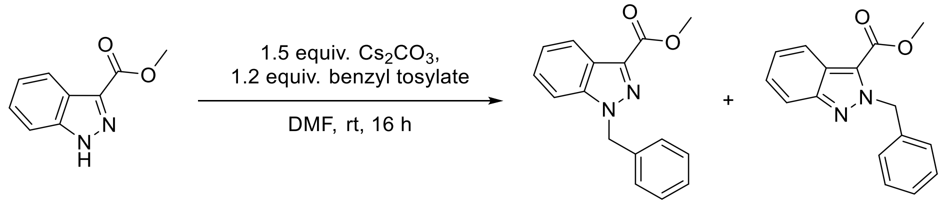

9 **69** **70**

^{1}H NMR (400 MHz, $CDCl_3$)

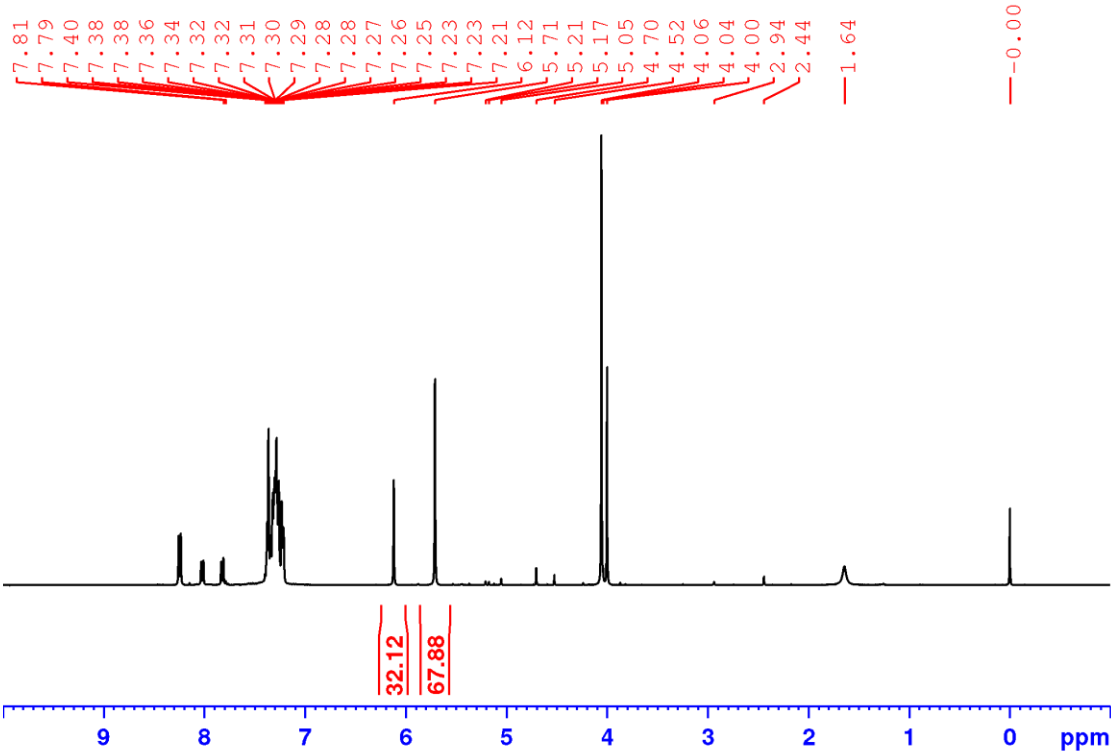

Table 6, Entry 4 (Conditions A)

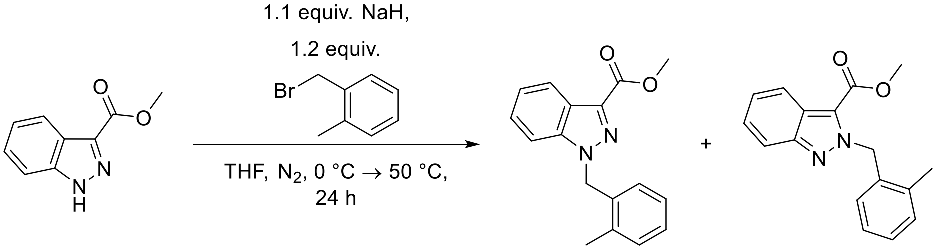

9 **71** **72**

^{1}H NMR (300 MHz, $CDCl_3$)

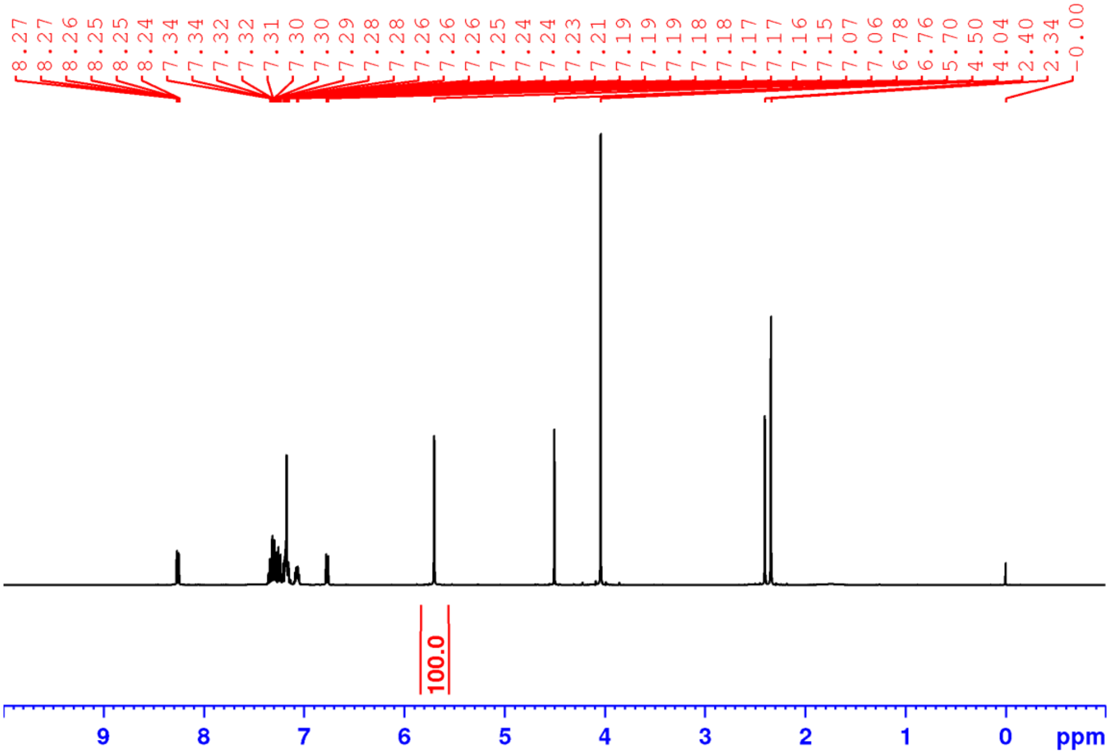

Table 6, Entry 4 (Conditions B)

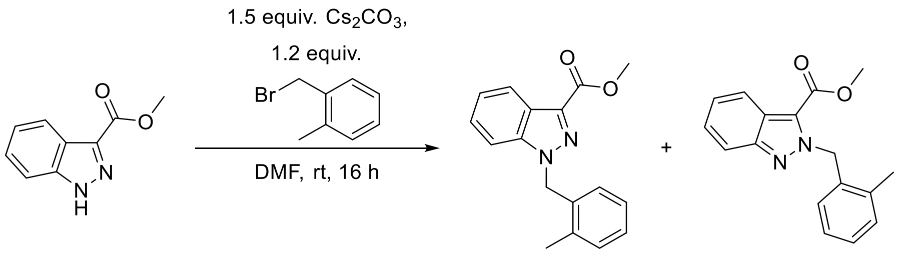

9 **71** **72**

^{1}H NMR (400 MHz, $CDCl_3$)

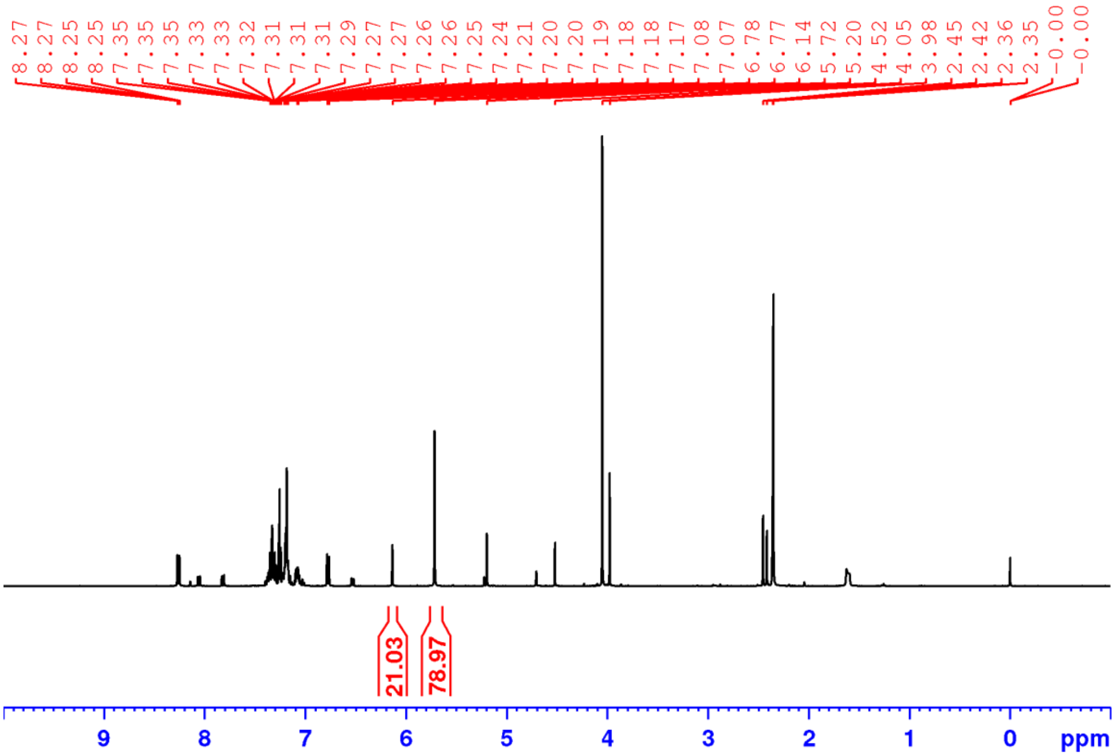

Table 6, Entry 5 (Conditions A)

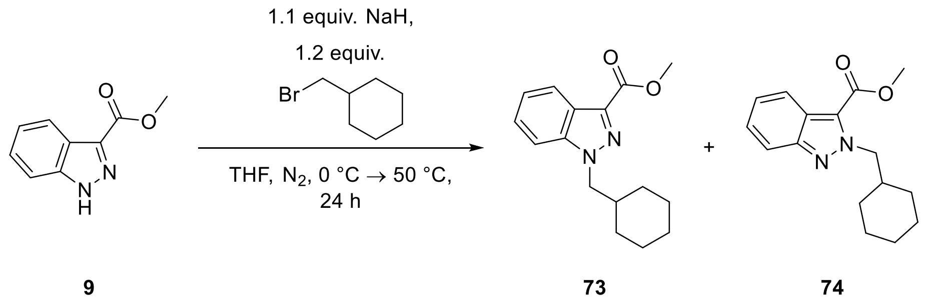

^{1}H NMR (300 MHz, $CDCl_3$)

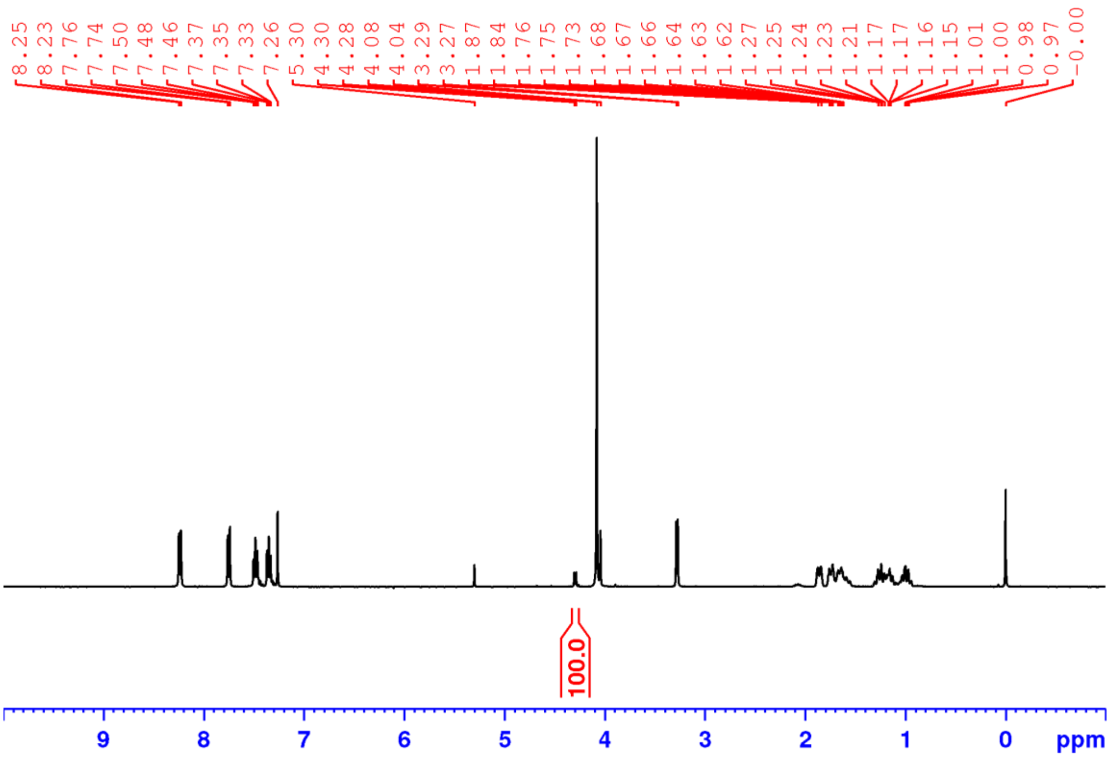

Table 6, Entry 5 (Conditions B)

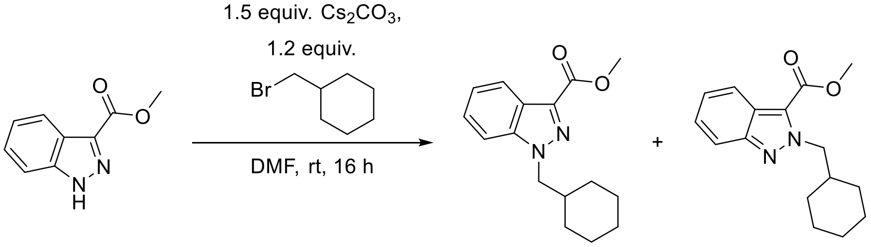

9 **73** **74**

^1H NMR (400 MHz, $CDCl_3$)

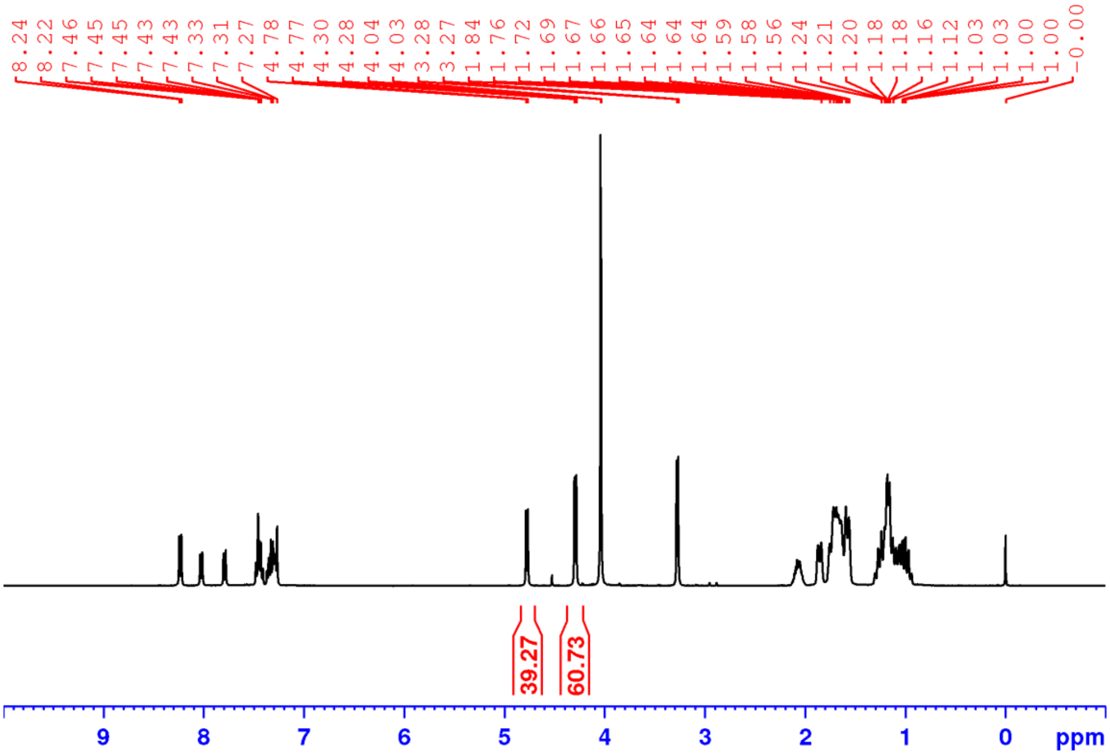

Table 6, Entry 6 (Conditions A)

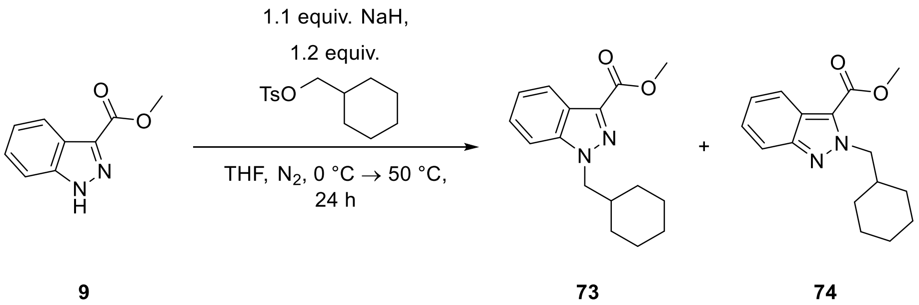

^{1}H NMR (300 MHz, $CDCl_3$)

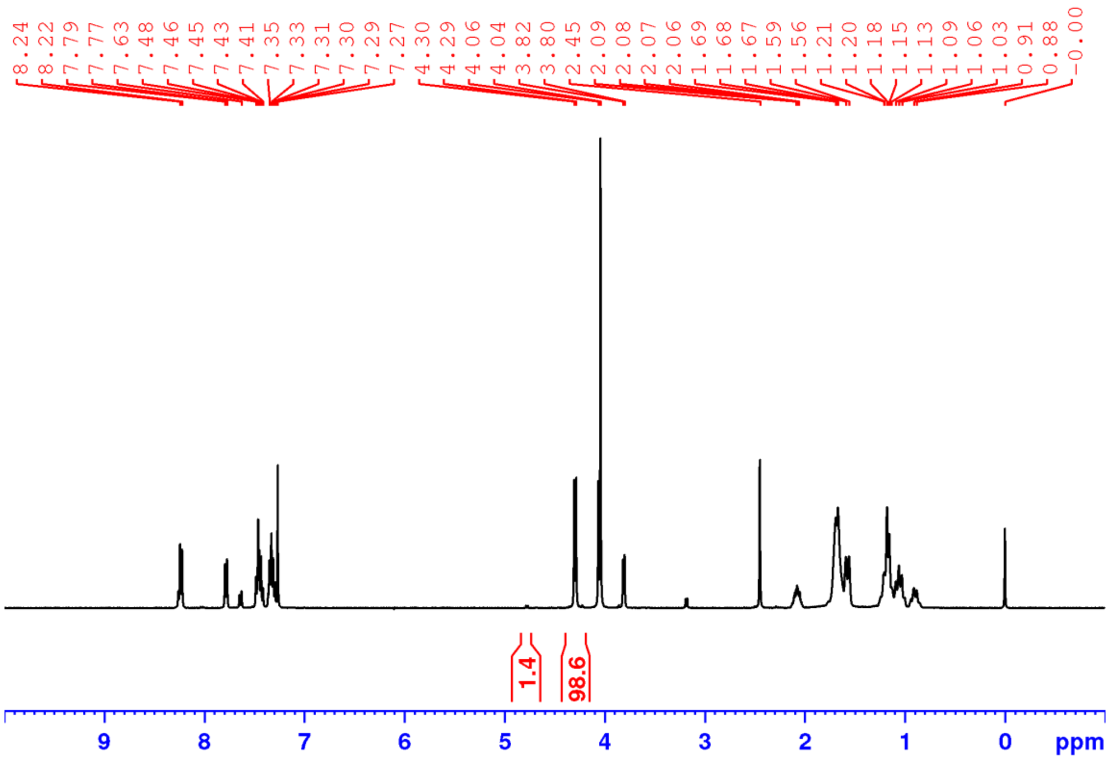

Table 6, Entry 6 (Conditions B)

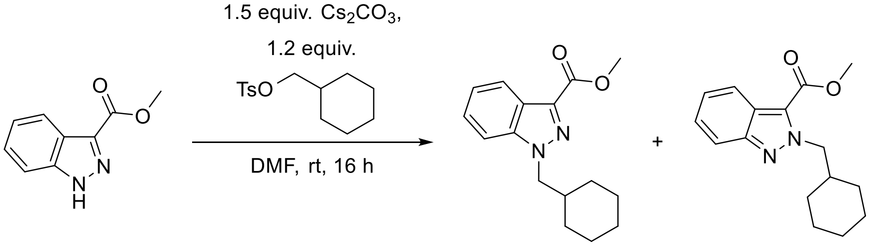

^{1}H NMR (400 MHz, $CDCl_3$)

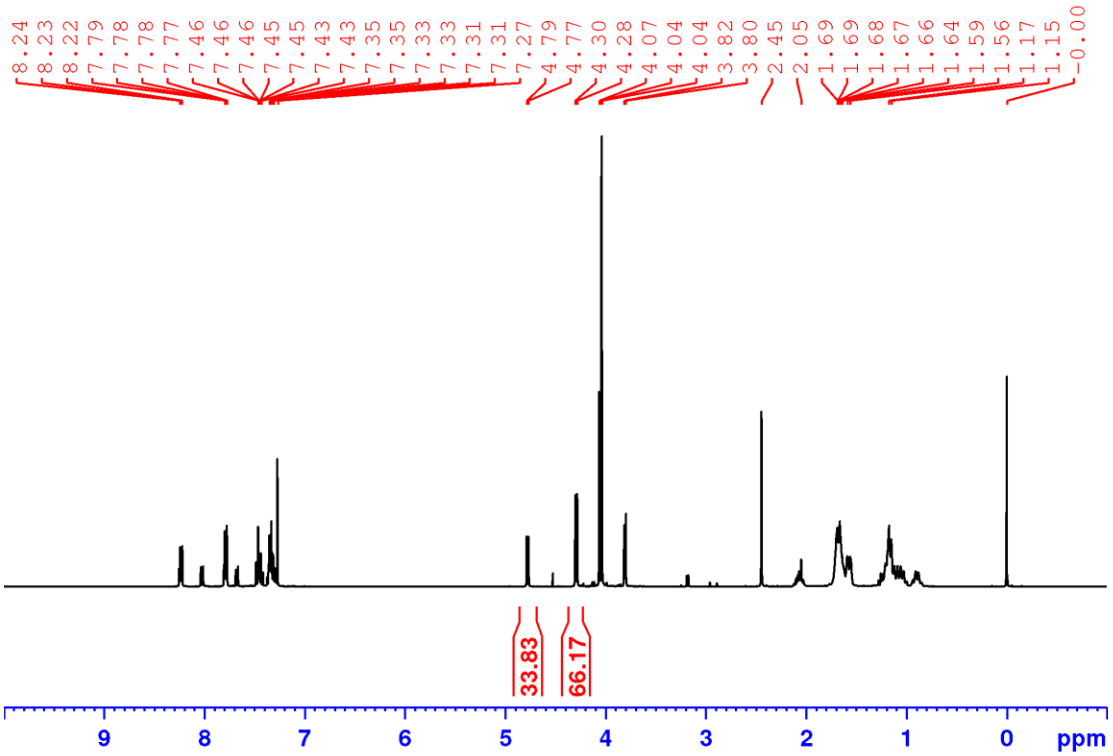

Table 6, Entry 7 (Conditions A)

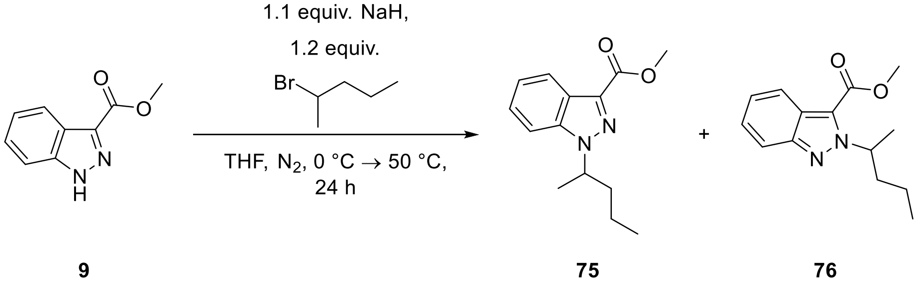

^{1}H NMR (300 MHz, $CDCl_3$)

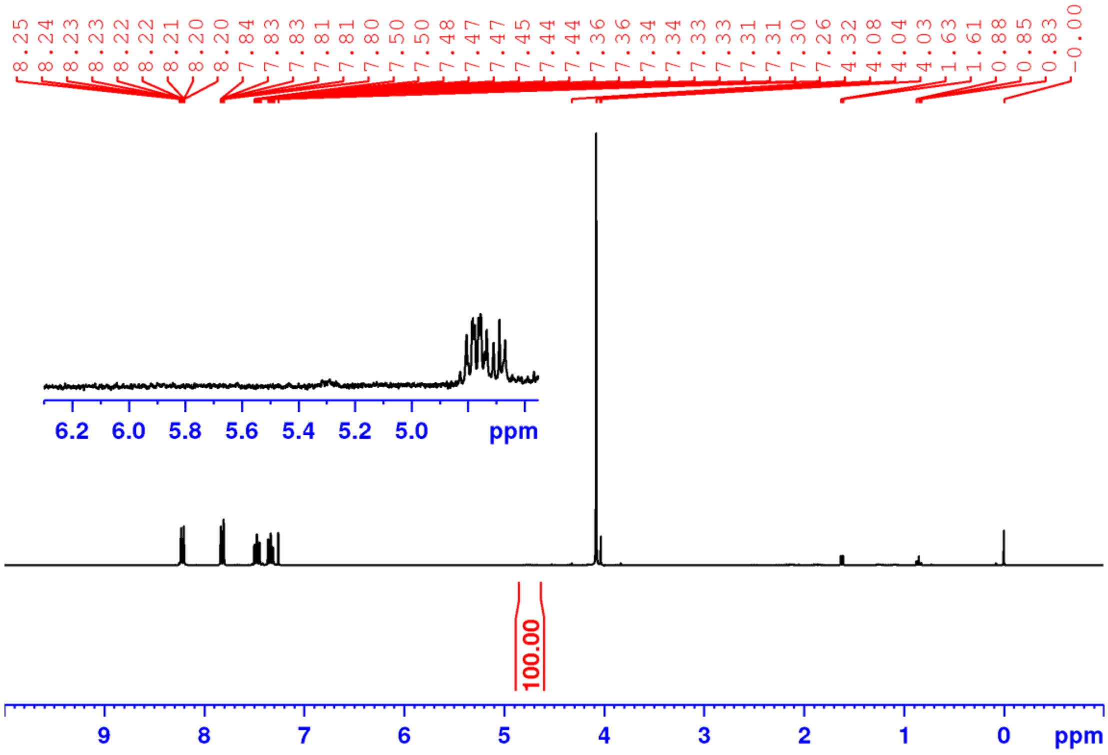

Table 6, Entry 7 (Conditions B)

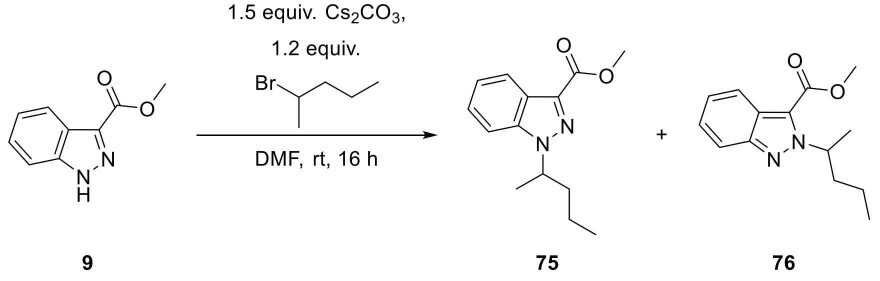

^1H NMR (400 MHz, $CDCl_3$)

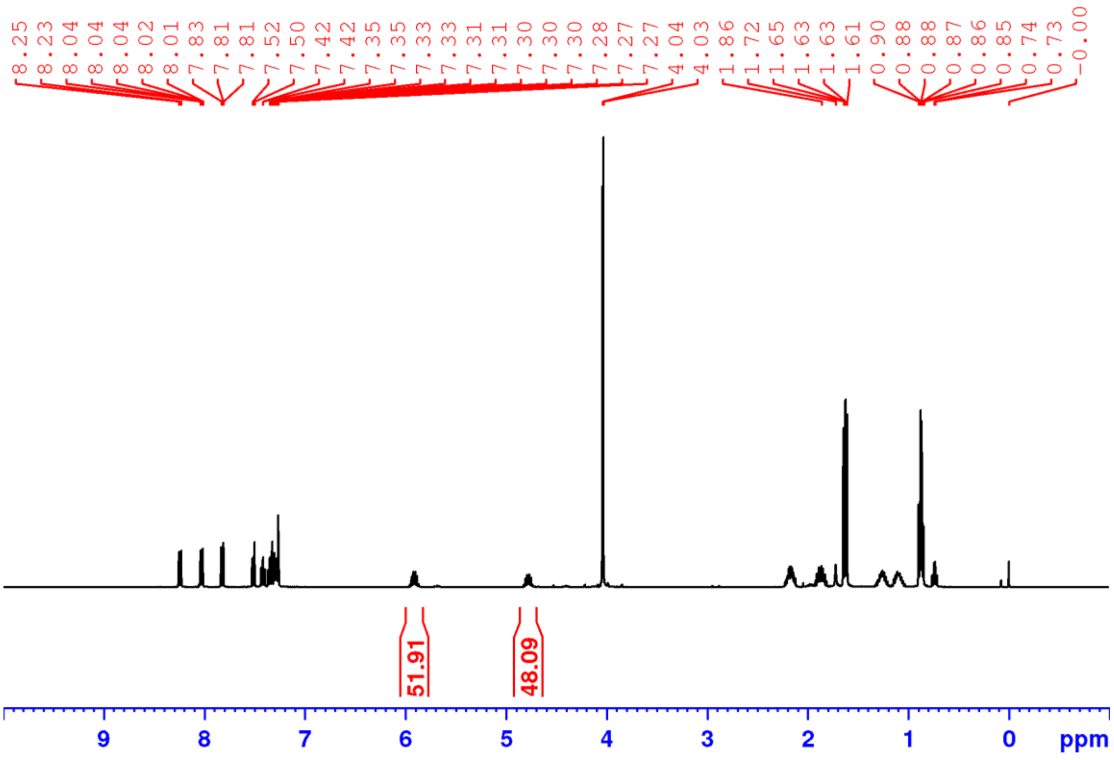

Table 6, Entry 8 (Conditions A)

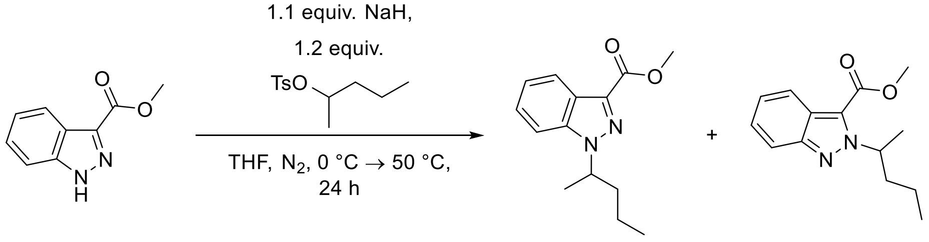

^1H NMR (300 MHz, $CDCl_3$)

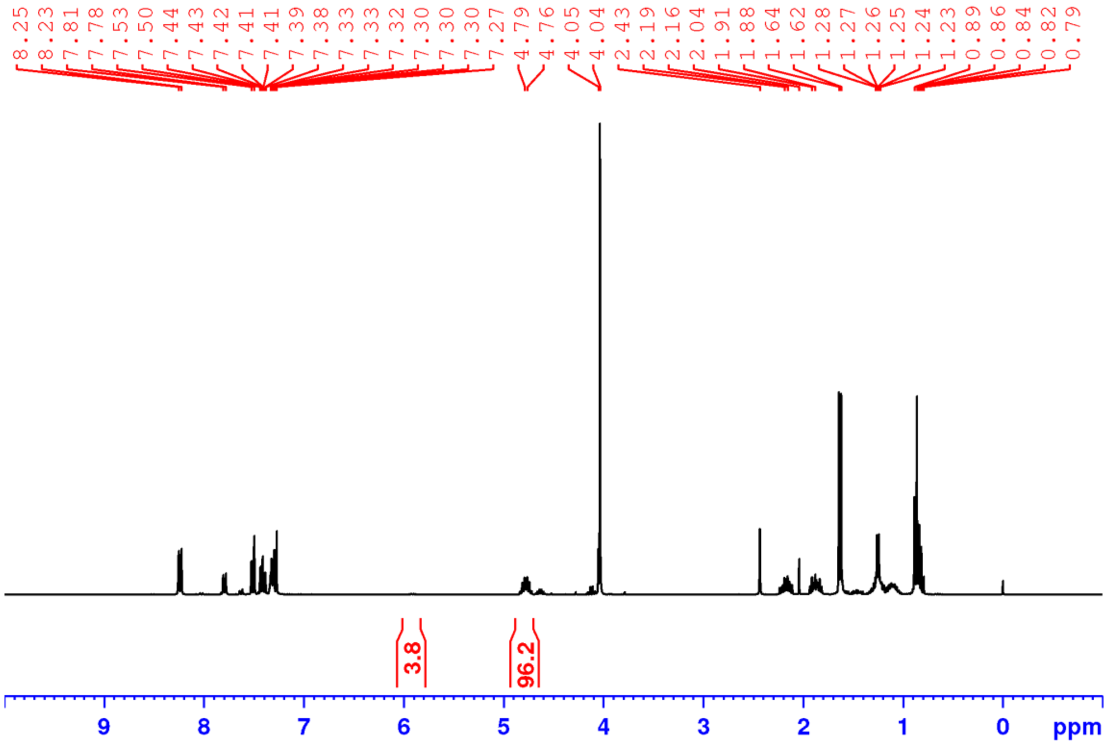

Table 6, Entry 8 (Conditions B)

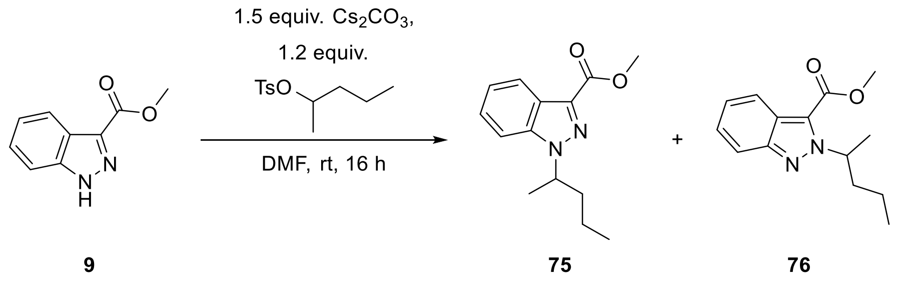

^{1}H NMR (300 MHz, $CDCl_3$)

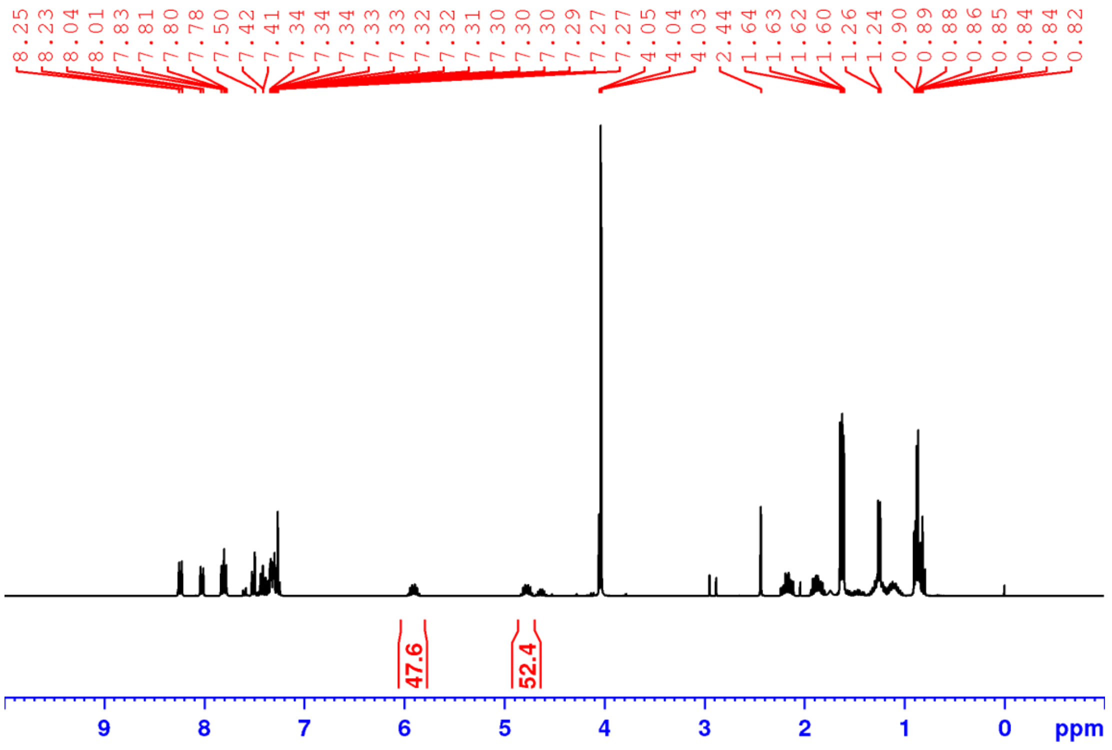

Table 6, Entry 9 (Conditions A)

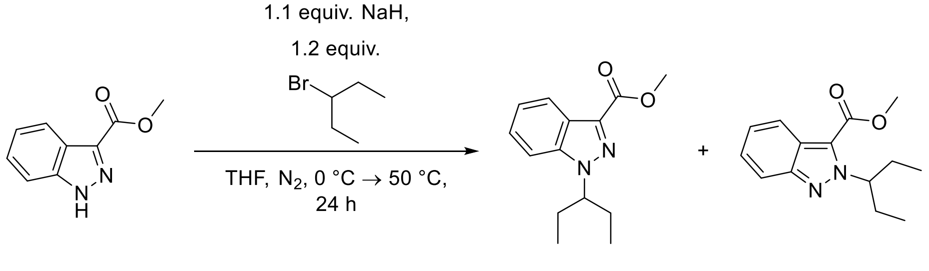

^{1}H NMR (300 MHz, $CDCl_3$)

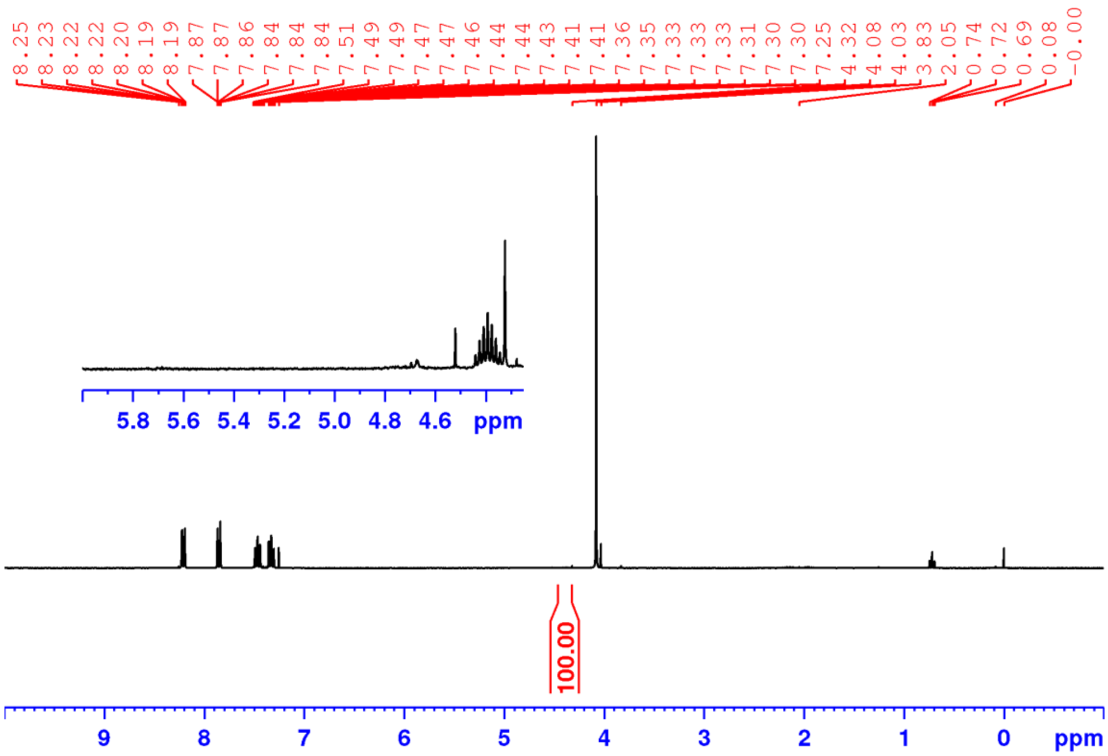

Table 6, Entry 9 (Conditions B)

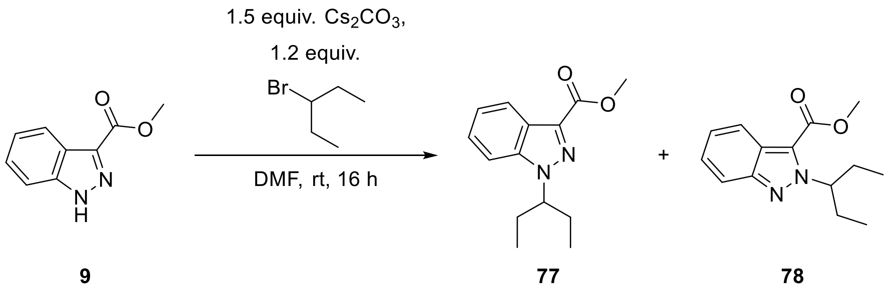

^{1}H NMR (400 MHz, $CDCl_3$)

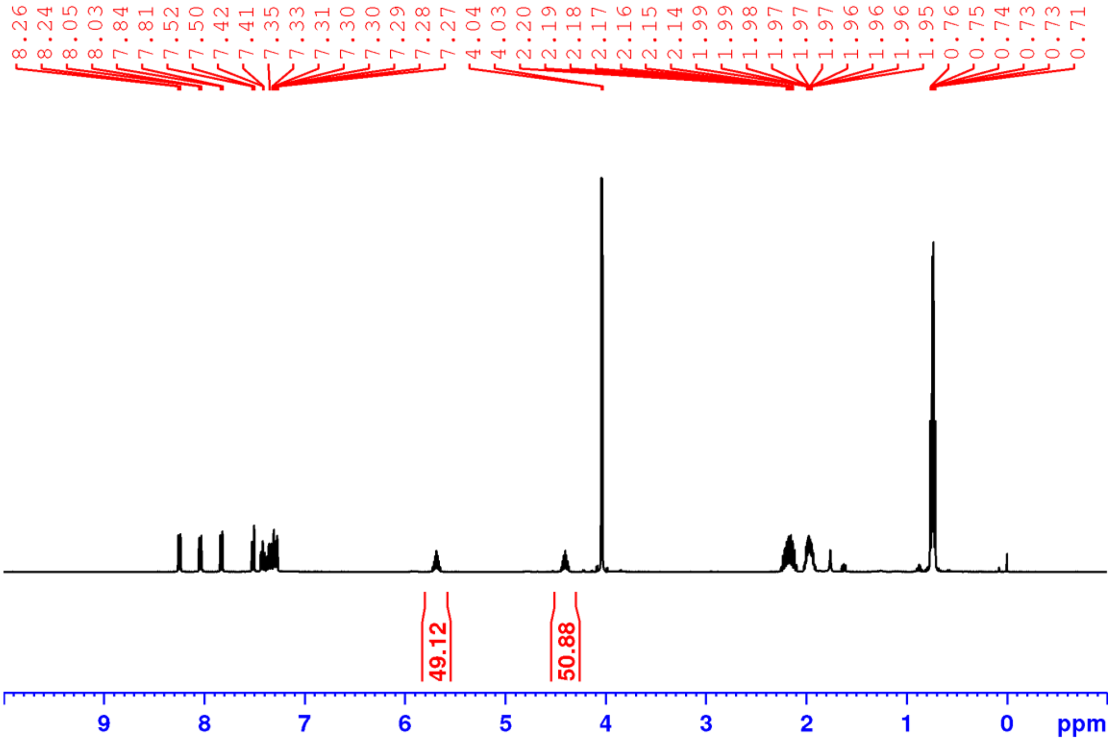

References

1. Ferrari, M.; Ripa, A.; Ripa, G.; Sisti, M. *J. Heterocycl. Chem.* **1989**, *26*, 531–532. DOI: 10.1002/jhet.5570260251
2. Tang, M.; Kong, Y.; Chu, B.; Feng, D. *Adv. Synth. Catal.* **2016**, *358*, 926 – 939. DOI: 10.1002/adsc.201500953
3. Banister, S.D.; Moir, M.; Stuart, J.; Kevin, R.C.; Wood, K.E.; Longworth, M.; Wilkinson, S.M.; Beinat, C.; Buchanan, A.S.; Glass, M.; Connor, M.; McGregor, I.S.; Kassiou, M. *ACS Chem. Neurosci.* **2015**, *6*, 1546–1559. DOI: 10.1021/acschemneuro.5b00112
4. Alaime, T.; Daniel, M.; Hiebel, M-A.; Pasquinet, E.; Suzenet, F.; Guillaumet, G. *Chem. Commun.* **2018**, *54*, 8411–8414. DOI: 10.1039/C8CC03612H
5. Bartsch R.A.; Yang I-W. *J. Het. Chem*. **1984**, *21*, 1063–1064. DOI: 10.1002/jhet.5570210428
6. Bamberger, E.; Goldberger, A.V. *Justus Liebigs Ann. Chem.* **1899**, *305*, 339–362. DOI: 10.1002/jlac.18993050306
7. Li, P.; Zhao, J.; Wu, C.; Larock, R.C.; Shi, F. *Org. Lett.* **2011**, *13*, 3340–3343. DOI: 10.1021/ol201086g
8. Chen, G.; Hu, M.; Peng, Y. *J. Org. Chem.* **2018**, *83*, 1591–1597. DOI: 10.1021/acs.joc.7b02857
9. Song, P.; Chen, M.; Ma, X.; Liu, T.; Zhou, Y.; Hu, Y. *Bioorg. Med. Chem.* **2015**, *23*, 1858–1868. DOI: 10.1016/j.bmc.2015.02.004
10. Tang, R-J.; Milcent, T.; Crousse, B. *J. Org. Chem.* **2018**, *83*, 930–938. DOI: 10.1021/acs.joc.7b02920
11. Chevalier, A.; Ouahrouch, A.; Arnaud, A.; Gallavardin, T.; Franck, X. *RSC Adv.* **2018**, *8*, 13121–13128. DOI: 10.1039/C8RA01546E
12. Longworth, M.; Banister, S.D.; Mack, J.B.C.; Glass, M.; Connor, M.; Kassiou, M. *Forensic Toxicol.* **2016**, *34*, 286–303. DOI: 10.1007/s11419-016-0316-y
13. Palit, S.; Bera, S.; Singh, M.; Mondal, D. *Synthesis*, **2015**, *47*, 3371–3384. DOI: 10.1055/s-0034-1381135
14. Palmieri, A.; Gabrielli, S.; Ballini, R. *Chem. Commun.* **2010**, *46*, 6165–6167. DOI: 10.1039/C0CC01097A
15. Kazemi, F.; Massah, A.R.; Javaherian, M. *Tetrahedron*, **2007**, *63*, 5083–5087. DOI: 10.1016/j.tet.2007.03.083
16. Maskill, H. *J. Chem. Soc., Perkin Trans. 2*, **1986**, 1241–1246. DOI: 10.1039/P29860001241

17. Weweler, J.; Younas, S.L.; Streuff, J. *Angew. Chem., Int. Ed.* **2019**, *58*, 17700–17703. DOI: 10.1002/anie.201908372
18. Deruer, E.; Hamel, V.; Blais, S.; Canesi, S. *Beilstein J. Org. Chem.* **2018**, *14*, 1203–1207. DOI: 10.3762/bjoc.14.101
